# Epithelial-mesenchymal transition, regulated by β-catenin and Twist, leads to esophageal wall remodeling in pediatric eosinophilic esophagitis

**DOI:** 10.1371/journal.pone.0264622

**Published:** 2022-03-03

**Authors:** Elizabeth Garcia, Zeenat Ladak, Takaaki Landry, Michael Wollin, Amit R. L. Persad, Consolato M. Sergi, Hien Q. Huynh, Rabindranath Persad, Sujata Persad

**Affiliations:** Department of Pediatrics, University of Alberta, Edmonton, Alberta, Canada; University of Colorado Boulder, UNITED STATES

## Abstract

Eosinophilic Esophagitis (EoE) is an antigen-triggered inflammatory condition of the esophageal lining characterized by eosinophilic infiltration. EoE is associated with significant remodeling, and although this remodeling is reversed by current treatment regimens, symptoms of EoE and associated remodeling reappear upon cessation of therapies. We hypothesized that structural remodeling of cell-cell adhesion is a key factor in the pathogenesis of EoE and that epithelial to mesenchymal transition (EMT) was a viable molecular process to lead to this remodeling. Endoscopically obtained biopsy samples from 18 EoE and 18 control pediatric patients were evaluated by transmission electron microscopy to measure intercellular spaces (IS) between cells. Biopsy samples from all groups were analyzed for cellular levels of cell-cell adhesion proteins: E-cadherin, zonula occludens associated protein-1 (ZO-1), and N-cadherin. We also analyzed for cellular levels and localization two of transcription factors, Twist1 and β-catenin, that are associated with promoting EMT. The IS was significantly increased in the EoE group compared to the control. We observed a significant decrease in E-cadherin and ZO-1 levels and a concomitant increase in N-cadherin levels in EoE samples compared to control. Further, while there was no significant change in cellular levels of β-catenin, we observed an altered localization of the protein from the cell membrane in control tissue to a nuclear/perinuclear localization in EoE. We observed higher levels of the transcription factor Twist1 in the EoE group compared to normal which was localized mainly at the nucleus. Our results suggest that the integrity of normally sealed esophageal epithelia is compromised in the EoE patients compared to control subjects, and this is due to alterations in the expression of cell adhesion molecules at the esophageal epithelium. Our data also suggest that EMT, potentially regulated by transcription factors β-catenin and Twist1, may be responsible for the molecular alteration which leads to the remodeling of esophageal epithelia in EoE.

## Introduction

Eosinophilic Esophagitis (EoE) is an antigen-triggered chronic inflammatory condition of the esophageal lining characterized by eosinophilic infiltration [1, 2]. Since the first clinical report by Attwood et al in 1993 [3], diagnostic criteria have evolved, with the most current guideline published in 2018 [2]. Inherent in the diagnosis is that EoE is distinct from GERD and other causes of esophageal eosinophilia [1, 2]. In the past 30 years there has been significant progress in delineating the pathogenesis of EoE with the predominant concept being sensitization by an allergen in a genetically susceptible host, generally with other types of hypersensitivity. This sensitization leads to an influx of eosinophils and the release of a variety of proinflammatory cytokines. The resultant tissue damage activates a series of signalling cascades potentiating the immune system [4, 5].

The epithelial layer of the esophagus is dependent on cell-cell adhesion to maintain its integrity, which includes tight maintenance of both barrier function and structure [6, 7]. The intercellular adhesions, which include the adherens junctions (AJ) and tight junctions (zonula occludens) (TJ), are mediated by cell adhesion proteins that include, E-Cadherin at the AJ, and Zonula Occludens-1 protein (ZO-1) at the TJ [6–10].

Epithelial-to-mesenchymal transition (EMT) is recognized as a normal physiologic response to tissue damage and has been shown to have a role in tissue differentiation in the developing embryo [10–14]. It is well described in cancer progression and has been increasingly recognized as a factor in tissue remodeling in chronic inflammatory diseases, including asthma, IBD and other pathophysiological conditions. It has been previously demonstrated that with chronic esophageal eosinophilic inflammation there is EMT associated with fibrosis in pediatric EoE [13–15]. This likely is reflected in increasing abnormality of esophageal structure and function and may culminate in stricture formation [15].

Dilated intercellular space (IS) has been previously described in patients with EoE [16, 17]^.^ The goal of our study was to determine the putative molecular mechanisms that may be involved in promoting the widening of IS. We evaluated alterations in adhesion molecules (E-cadherin & ZO-1) that maintain cell-cell interactions at cell adhesion sites and the putative role of EMT in promoting these alterations. We observed significantly lower levels of the E-cadherin and ZO-1 in in EoE compared to control tissue. In parallel, we observed higher levels and altered subcellular localization of the transcription factors β-catenin and Twist1, two well-known regulators of EMT, in EoE compared to control tissue. We propose that β-catenin and Twist1 may be instrumental in promoting EMT changes that culminates in significant alterations in epithelial cell adhesion molecules and the remodeling of esophageal epithelia in EoE.

## Material and methods

### Sample selection

This project was approved by the Health Research Ethics Board at the University of Alberta and Alberta Health Services, Edmonton (Pro00002336). This study was a retrospective study using formalin-fixed, paraffin embedded (FFPE) archival tissue for immunohistochemical and immunofluorescence analysis and parallel specimen from each patient which was fixed immediately in glutaraldehyde plus paraformaldehyde for transmission electron microscopy analysis as described below. All patient data were fully anonymized. Esophageal tissue samples from pediatric patients with EoE were selected from our EoE clinic pathologic database, who presented with the onset of EoE before 18 years of age. All patients met diagnostic criteria for the absence of pathologic acid reflux based on 24-hour pH probe criteria or failure to respond to anti-reflux therapy with proton pump inhibitor (PPI) 4 weeks before endoscopy. Samples were selected for inclusion when there was endoscopic evidence of EoE with histologic evidence of >15 Eosinophils/high-power field (Eos/HFP). This included both the macroscopic presence of linear furrows, white exudates, and mucosal edema. In addition, there was no anatomical division into the upper versus lower esophagus +/- mid esophagus. A board-certified pediatric pathologist reviewed all the biopsies, and eosinophils were manually quantified.

The EoE group was compared to biopsy samples from the control group. Control samples were selected from our endoscopic database of a patient population that underwent endoscopy for recurrent abdominal pain. All patients in this group exhibited a macroscopically normal-appearing esophagus. Less than 5 Eos/HPF were present in all control biopsy samples. Patients with other inflammatory conditions, including celiac disease, inflammatory bowel diseases, polyposis syndromes, or hypereosinophilic syndrome, were excluded. Random biopsy samples were obtained from 18 pediatric patients in the EoE clinic database and 18 control patients from the pediatric endoscopy database, meeting the above set of criteria and based on power calculation.

The biopsies were performed using 2.8-mm Single-Use Radial Jaw^TM^ biopsy forceps (Boston Scientific, USA).

### Sample size

All sample size/power calculations were computed using PASS 2002 and assuming a Bonferroni corrected significance level (α) of 0.007. Assuming a maximum within-group SD (S_max_) of 24, one-way analysis of variance (ANOVA) with 18 archived biopsies per group/location, achieving 95% power to detect differences in protein expressions. A sample size of 18 archived biopsies from each group and location combination were used.

For TEM a sample size of 18 fresh biopsies from each group and location combination was used for TEM analysis.

### Transmission electron microscopy (TEM)

Specimens from each patient were immediately fixed in 0.1 mol/L phosphate buffer, pH 7.2, containing 2% glutaraldehyde plus 2% paraformaldehyde and coded so that the category of the patient (EoE or control) was unknown to the morphologist. One specimen from each patient was selected for processing by transmission electron microscopy. The specimen was rinsed in phosphate buffer, post-fixed in 1% buffered osmium tetroxide, and dehydrated through a graded alcohol series. It was then infiltrated through propylene oxide and embedded in an epoxy resin. Blocks were trimmed, and ultrathin sections on copper grids were post-stained with uranyl acetate and lead citrate. Each specimen was examined and photographed in a JEOL 2100 transmission electron microscope at an accelerating voltage of 200kV. All images were taken at a 15K magnification (JEOL Ltd., Japan) using a digital camera with Gatan Digital micrograph software (Gatan, USA). For each sample, sequential images were taken starting from the surface and moving down to the basement membrane. Images were labelled accordingly. Using ImageJ software, the mean value of IS distance was computed for each patient by averaging 100 measurements of perpendicular space between adjacent cell membranes (ignoring cytoplasmic projections or microvilli).

### Immunofluorescence analysis

Formaldehyde fixed paraffin-embedded (FFPE) tissue sections (approximately 4 μm thick) were dewaxed: (45 min at 60°C), hydrated, and blocked for nonspecific binding with 5% goat serum or 10% bovine serum albumin. The presence of eosinophils in the esophageal tissue of each group was demonstrated in the same archived samples in our previous publication [18] by staining for major basic protein (MBP), the degranulation product of eosinophils that plays a role in the release of inflammatory mediators [19].

Tissue sections were first incubated with primary antibodies: anti-E-cadherin (Cell Signaling), anti-N-cadherin (Santa Cruz Biotechnology), anti-Twist1 (Cell Signaling), anti-Zonula Occludens-1-Alexa 488 (Invitrogen), and anti-β-catenin (Cell Signaling). Thereafter, primary antibody probed tissue sections were incubated with the appropriate Alexafluor-488 or Alexafluor-555 conjugated secondary fluorescent antibodies (Invitrogen, Carlsbad, CA). Negative controls included replacing the primary antibody with normal rabbit serum. Data were evaluated by computer-assisted quantification by two independent observers blinded to the clinical parameters of the samples. Results are expressed as the number of cells with positive staining/High Powered Field (HPF) Expression of the individual proteins were determined by microscopy, and staining intensity was quantified by Metamorph 7.6.3.0 (Molecular Devices, Sunnyvale, CA). IF with different antibodies was carried out in consecutive tissue sections from each FFPE tissue specimens. The area of tissue was consistent between stains and samples.

### Immunohistochemical analysis

Antibodies used for staining included anti-β-Catenin Clone 14/β-Catenin (Catalog no. 610154, BD Biosciences). For deparaffinization and rehydration, tissue slides were baked at 60° Celsius for 2 hours, immersed in xylene (2X) for 2 minutes followed by graded ethanol including 100% (2X), 95%, 85%, 75% and 50%, respectively, for 2 minutes each and then ddH2O for 5 minutes. Antigen presentation was carried out by immersing slides in boiling sodium citrate (10mM) for 30 minutes. The slides were washed 3X with wash solution (1X PBS with 0.05% Triton X-100) for 5 minutes after each step starting from antigen presentation until after ABC solution incubation. Thereafter, slides were blocked for 2 hours in blocking buffer (1X PBS, 5% goat serum, 0.2% Triton X-100, 0.1% BSA and ddH_2_O) followed by overnight incubation with primary antibody (1:200 dilution) at 4° Celsius. Following primary antibody incubation, slides were incubated with 0.3% H_2_O_2_ for 30 minutes and then incubated with HRP-labelled secondary antibody (Catalog no. NEF822001EA, Perkin Elmer) for 2 hours. For signal amplification, slides were treated with Tyramide Signal Amplification (Catalog no. NEL700A001KT, Perkin Elmer) reagent and Avidin-Biotin Complex solution (Catalog no. PK-6100, Vector Laboratories) for 7 and 30 minutes, respectively. After this, they were immersed in DAB chromogenic substrate (Catalog no. SK-4105, Vector Laboratories) for 1–10 minutes until a brown stain was detected and then washed under running tap water for 5 minutes. This was followed by hematoxylin (Catalog no. SH26-500D, Fisher Scientific) staining for 30 seconds, followed by incubation with Scott’s Topwater (3.5g sodium bicarbonate, 20g magnesium sulfate and ddH_2_O). After that, the slides were dehydrated in graded EtOH with increasing concentrations; 50%, 75%, 85%, 95%, and 100%, respectively, for two minutes each, followed by xylene for 2 minutes. Coverslips were then mounted on slides using Permount (Catalog no. SP15-100, Fisher Scientific).

### Statistical analysis

Prism graph software was used for statistical analysis including calculation of the mean and the standard error of EoE and control groups. T-test was used for the comparison of means. Probability values of <0.05 were considered statistically significant.

For TEM analysis means of measured distances were calculated for each sample. Data were analyzed using IBM SPSS software, ANOVA with LSD post Hoc test.

## Results

### Patient characteristic

The patient population for this study were the same patients that has been described/reported in our previous published study [18] (S1 Table). The mean age of patients with EoE (n = 18) and control (n = 18) was 12 and 9.9 years, respectively [18]. 78% of the children with EoE were male and 22% were female. In the control group, 56% of the children were male 44% were female. Baseline characteristics (patient characteristics and endoscopic characteristics) were presented in our previous study [18]. Briefly, all control samples exhibited either a macroscopically normal-appearing esophagus with no loss of vascular pattern and/or erosions in the lower esophagus. None of the patients with EoE had a normal endoscopy of the esophagus and exhibited endoscopic features that included loss of vascular pattern, vertical furrows, concentric rings, and white exudates [20]. All patients with EoE had a normal endoscopy for the stomach and small intestines. The mean number of esophageal eosinophils was 65 Eos/HPF (range 30–100) in patients with EoE compared with 3 Eos/HPF in our control group (range 0–5).

### Intercellular space is wider in EoE patient samples compared to control

**Fig 1:**
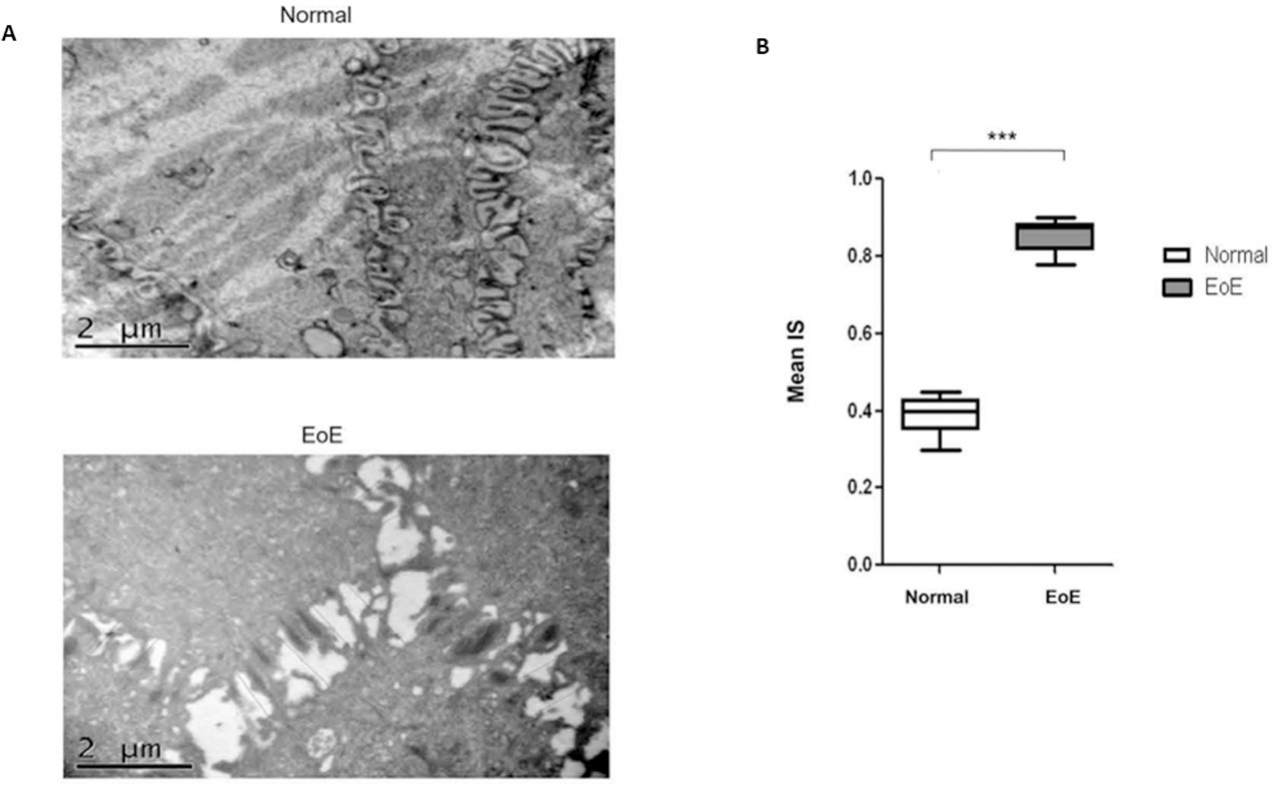
**A.** TEM images of intercellular space (IS) in esophageal biopsies of Normal (0–5 Eos/HPF) and EoE (>15 Eos/HPF) pediatric patients. Lines perpendicular to membranes indicate areas of measurement. **B.** Quantification (box plot) shows that IS in EoE is significantly dilated compared to normal (p<0.001). ***P<0.001.

Epithelial damage is known to contribute significantly to the pathogenesis of allergic disorders such as asthma by inducing injury of the barrier function and changes in the permeability of the mucosa [21, 22]. Using transmission electron microscopy (TEM), we quantified the IS between cells in the esophageal epithelial layers in EoE and normal patient biopsies. While tissues from normal patients showed intact tight junctions between cells in the epithelia (0.3896 μm +/-0.0211), there was significant gaps between adjacent cells in the EoE samples (0.8567 μm +/-0.0180) (Fig 1A), indicating a deterioration of cell-cell adhesions in EoE. Quantification of IS shows that the difference in the space in EoE compared to normal epithelia is significant (p<0.001) (Fig 1B).

### E-cadherin & Zonula Occludens-1 proteins are lower in EoE compared to normal

**Fig 2:**
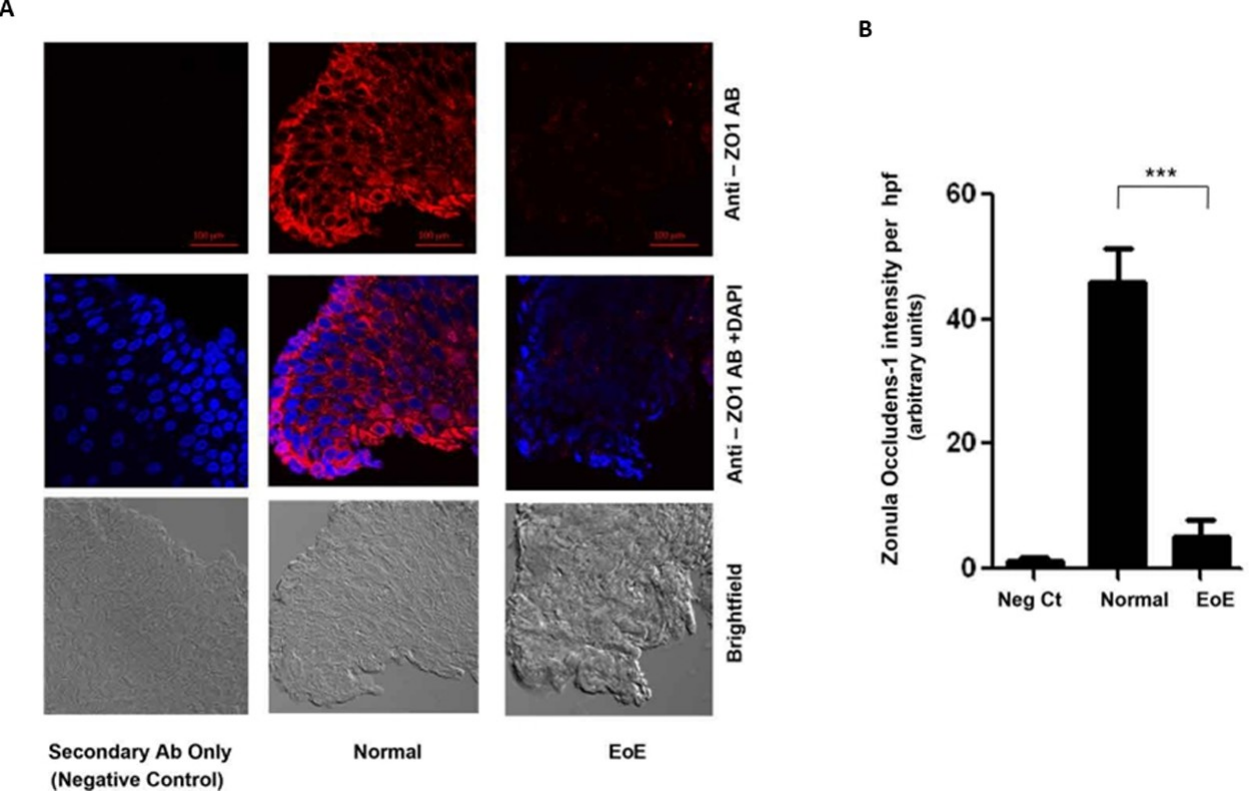
Representative photomicrograph and quantitation of IF analysis of control and EoE esophageal tissue: Photomicrograph of: **A.** Zonula Occludens-1 and **B.** E-cadherin. ***P<0.001.

**Fig 3:**
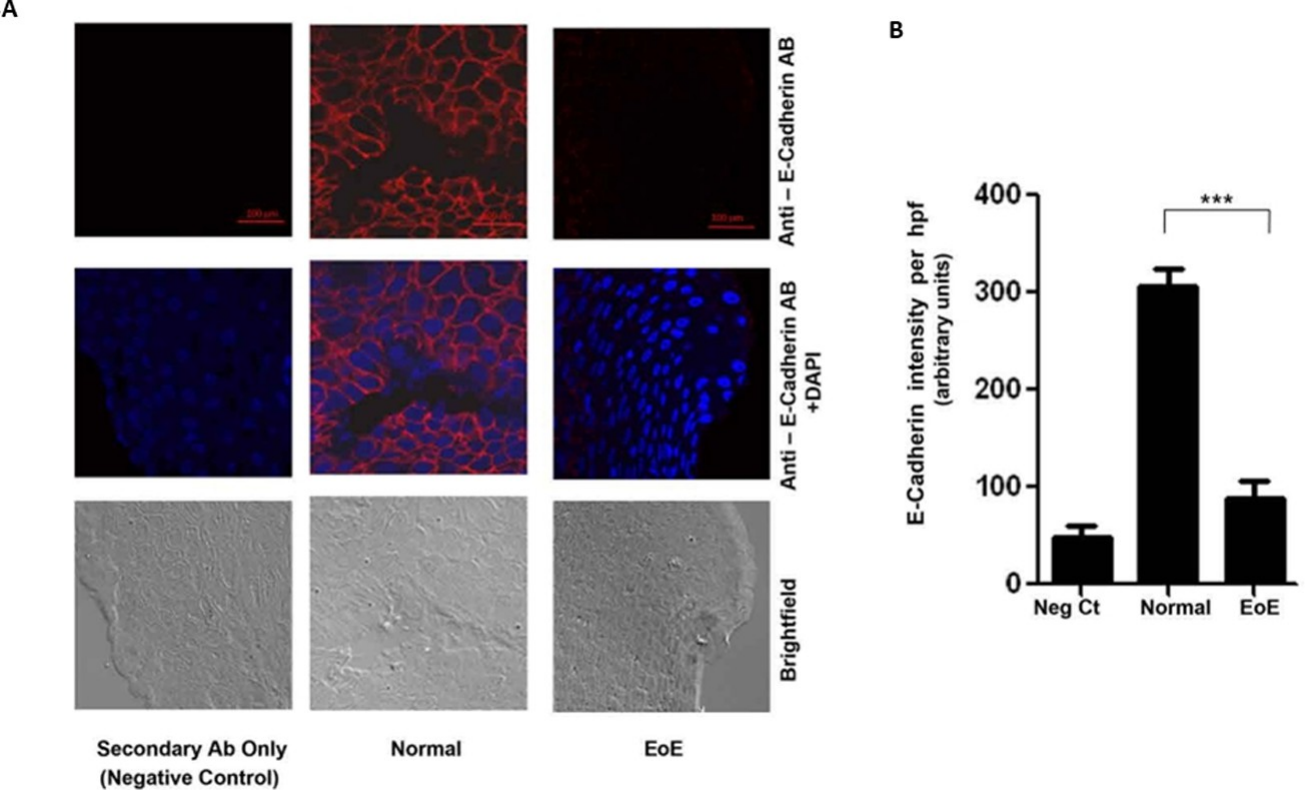
Densitometric analysis of: **A.** Zonula Occludens-1 and **B.** E-cadherin. Values are representative of the mean of a sample size of 18 control and 18 patients with EoE. ***P<0.001.

Intercellular adhesions, which include the adherens junctions (AJ) and tight junctions (zonula occludens) (TJ), are mediated by cell adhesion molecules, mainly E-Cadherin at the AJ, and Zonula Occludens-1 protein (ZO-1) at the TJ [6–9]. We compared cellular levels of E-Cadherin and ZO-1 in EoE and normal esophageal epithelia. Fig 2A is representative immunofluorescence (IF) analysis which shows that there were dramatically lower levels of ZO-1 in the EoE compared to the normal esophageal epithelia. Quantification of the levels and statistical analysis shows that this difference in ZO-1 levels is significant (p<0.001) (Fig 2B). Similarly, IF analysis showed that E-cadherin levels, which is prominently seen at the cell surface of the normal epithelia, were markedly decreased in EoE samples (Fig 3A). Quantification of the levels and statistical analysis shows that this difference in E-cadherin levels is significantly (p<0.001) attenuated in the EoE epithelia (Fig 3B).

### β-catenin localization is altered in EoE

**Fig 4:**
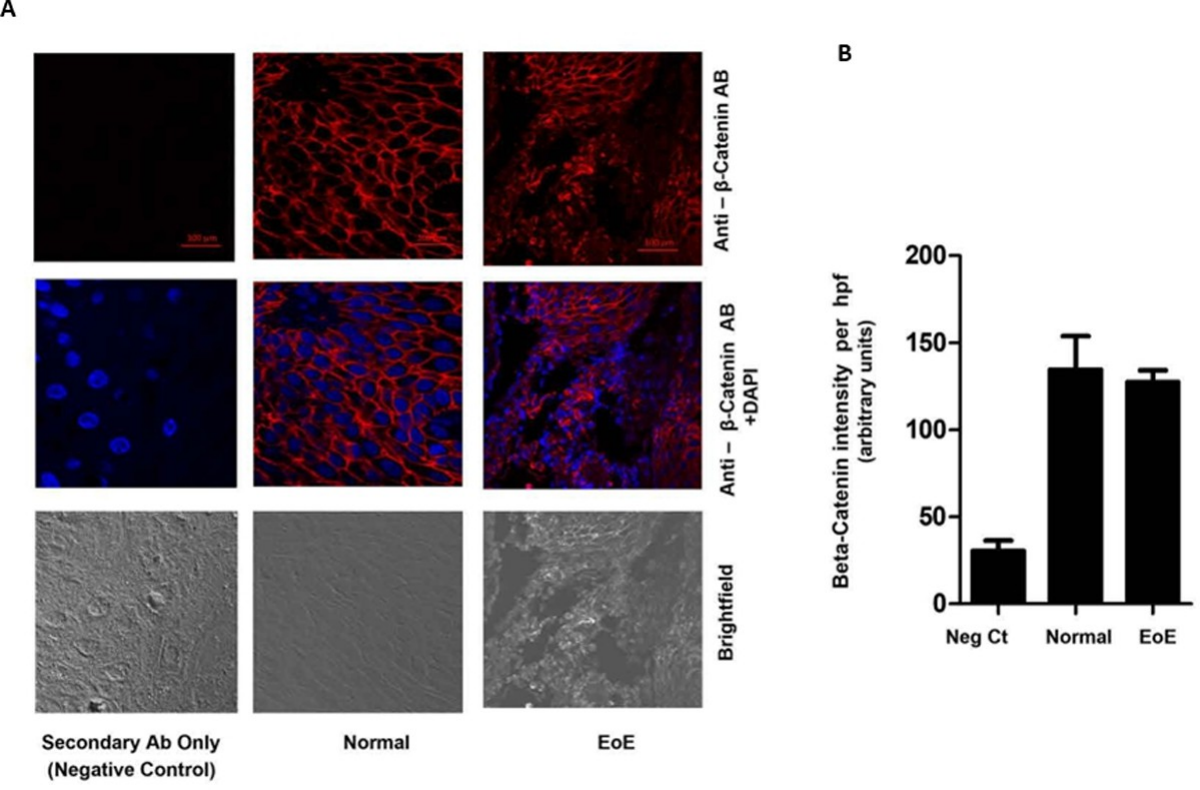
IF and IHC analysis of β-catenin in esophageal tissue from pediatric control (0–5 Eos/HPF) and EoE patients (>15 Eos/HPF). **A.** Representative photomicrograph of immunostaining (IF) with anti- β-catenin antibody. **B.** Densitometric analysis of β-catenin staining in EoE and control patient esophageal biopsy tissue.

**Fig 5:**
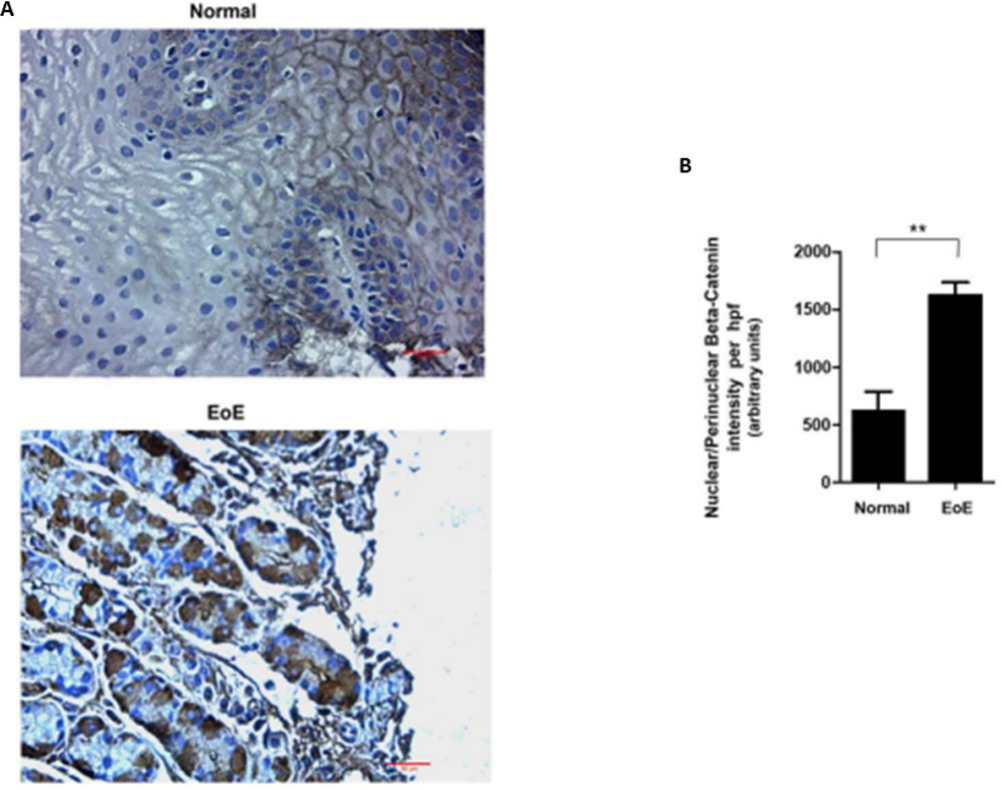
**A.** IHC analysis of β-catenin in esophageal tissue from pediatric control and EoE patients showing β-catenin localized mainly at nuclear/perinuclear space. **B.** Densitometric analysis of nuclear/perinuclear β-catenin in EoE and control patient esophageal biopsy tissue. Values are representative of the mean of a sample size of 18 control and 18 patients with EoE. **P<0.05.

Beta (β)-catenin is a multifunctional protein that serves a structural role at the adherens junctions, bound to E-cadherin [23] and a regulatory function as a transcriptional co-activator mediating Wnt/wingless canonical pathway signal transduction. Since E-cadherin levels decrease significantly in EoE compared to control esophageal tissue, we evaluated levels and localization of β-catenin. IF analysis of β-catenin shows that while β-catenin is exclusively localized at the cell membrane in normal esophageal tissue, the protein becomes more diffusely localized at the nuclear, peri-nuclear, and cytoplasmic compartments in EoE (Fig 4A). Densitometry analysis of the β-catenin staining shows that there is no significant increase in β-catenin levels in EoE compared to normal esophageal tissue (p-value<0.05) (Fig 4B). Our findings, concerning the subcellular relocalization of β-catenin, were confirmed by evaluating tissue levels and localization of β-catenin using immunohistochemical analysis (IHC) (Fig 5A). Fig 5B shows that while β-catenin is discreetly localized at the cell membrane in normal esophageal tissue, the localization of the protein is prominently more nuclear/peri-nuclear in EoE tissue.

### N-cadherin and Twist1 levels are higher in EoE compared to normal

**Fig 6:**
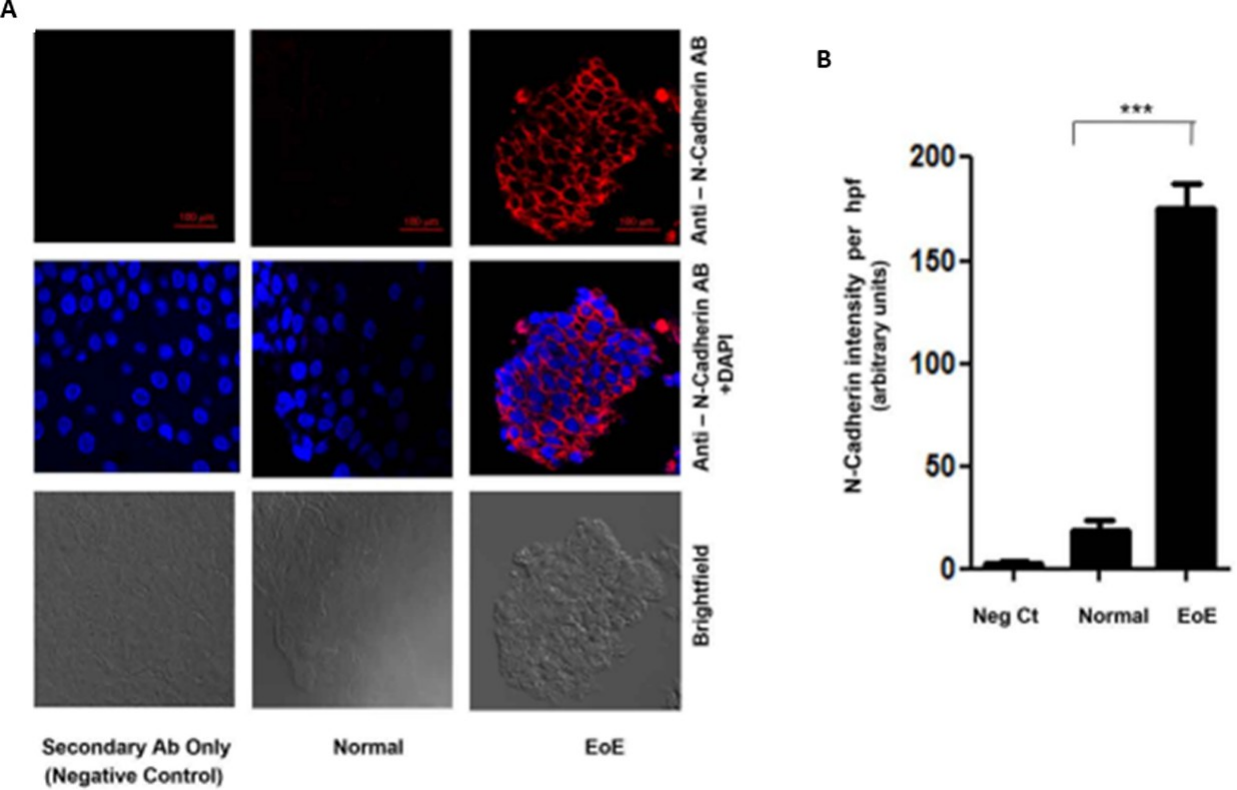
IF analysis of N-cadherin in esophageal tissue from pediatric control (0–5 Eos/HPF) and EoE patients (>15 Eos/HPF). **A.** Representative photomicrograph of immunostaining with anti-N-cadherin antibody. **B**. Densitometric analysis of N-Cadherin staining in EoE and control patient esophageal biopsy tissue. Values are representative of the mean of a sample size of 18 control and 18 patients with EoE. ***P<0.001.

We questioned whether, mechanistically, an epithelial to mesenchymal transition (EMT) type process was activated in EoE to induce the esophageal wall remodeling associated with EoE. Accordingly, we evaluated the presence of the mesenchymal cadherin, N-cadherin. IF analysis showed that while esophageal control tissue did not show any identifiable levels of N-cadherin, there was an abundance of N-cadherin present in EoE tissue (Fig 6A). Quantification of the levels and statistical analysis shows that the difference in N-cadherin levels was significant (p<0.001) (Fig 6B).

**Fig 7:**
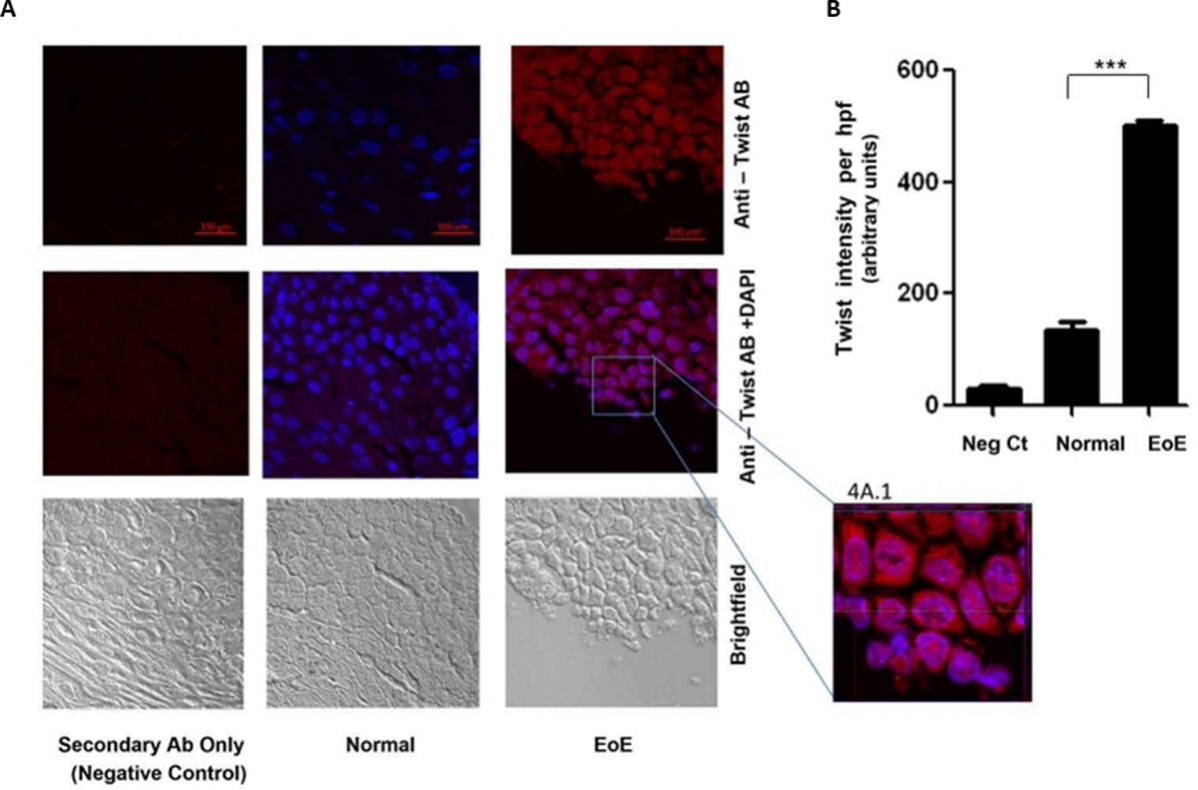
IF analysis of TWIST1 in esophageal tissue from pediatric control (0–5 Eos/HPF) and EoE patients (>15 Eos/HPF). **A.** Representative photomicrograph of immunostaining with anti-TWIST1 antibody; **A.1.** 60X photomicrograph showing Twist present exclusively in nuclei. **B.** Densitometric analysis of TWIST1 staining in EoE and control patient esophageal biopsy tissue. Values are representative of the mean of a sample size of 18 control and 18 patients with EoE. ***P<0.001.

We also examined tissue levels and nuclear localization of the transcription factor Twist1. Twist1 is an EMT inducer that is well documented to regulate EMT-related gene expression. IF analysis shows that while there were non-identifiable levels of Twist1 in normal tissue, there was a dramatic appearance of Twist1 in EoE tissue (Fig 7A). Further, Twist1 protein was present almost exclusively within the nucleus (Fig 7A.1). Quantification of the levels and statistical analysis shows that the difference in Twist1 levels between EoE and control was statistically significant (p<0.001) (Fig 7B).

## Discussion

EoE is a chronic inflammatory condition with an immunoallergic pathogenesis, related to food allergens involving an allergen-initiated, TH2-cytokine-dependent, IL-5-mediated infiltration of eosinophils to the esophageal mucosa [10–12, 14, 24–26]. Accordingly, dietary therapy induces and maintains remission in a significant proportions of patients [27, 28]. EoE is associated with significant remodeling, and it is evident that while this remodeling is reversed by the current treatment regimens, symptoms of EoE and associated remodeling of esophageal wall resumes upon cessation of therapies. Further, it is of interest that the hypersensitivity reaction is limited to the esophagus. Unlike the columnar epithelium of the distal GI tract, the esophagus has a stratified squamous epithelium reliant on adherens and tight junctions for fidelity [12, 14]. In this manuscript, we show significant decreases in two key cell-cell adhesion molecules that maintain the integrity of the barrier function of the esophageal epithelia, E-cadherin and ZO-1, in EoE tissue compared to normal. In addition, we report higher levels and subcellular relocalization of the transcription factors β-catenin and Twist1, in EoE tissue compared to control. We propose that the higher cellular levels of these transcription factors may be instrumental in transcriptionally altering the expressions of epithelial cell adhesion molecules resulting in compromise to the fidelity of the adherens and tight junctions leading to structural remodeling of the esophageal wall, a key factor in the pathogenesis of EoE.

We compared the intercellular distance in the esophageal epithelia of EoE samples to normal esophageal tissue. Our results show significantly greater gaps between epithelial cells in EoE than normal esophagi, where the gaps were non-identifiable. Intracellular adhesions are maintained by cell-cell adhesion molecules such as cadherins (adherens junctions) and ZO-1 (zonula occludens/tight junctions), among others. Classical cadherins, such as E-cadherin, are the major transmembrane protein of the adherens junction and mediate intercellular contacts through trans-pairing between cadherins on opposing cells [10, 29]. E-cadherin is the core transmembrane protein of the adherens junction and is required for binding and localization of several essential cytoplasmic proteins termed catenins that connect the cadherin complex to the actin cytoskeleton and several intracellular signaling pathways that control gene transcription [14, 30]. Important in the context of this study, the formation of the adherens junction is critical to the assembly of the tight junction, although E-cadherin is subsequently not required to maintain a tight junction organization once formed [31, 32]. Thus, the breakdown of the adherens junctions due to loss of E-cadherin can effectively impact the formation of tight junctions as well [14]. Our results show significant decreases in cellular expressions of both adhesion molecules, E-cadherin and ZO-1, which can effectively impact the integrity of cell adhesion by compromising both the adherens and tight junctions. Therefore, the initial loss of E-cadherin, which results in compromise of the adherens junctions, may also impact the subsequent assembly of the tight junction.

Loss of E-cadherin also results in the release of β-catenin from adherens junctions, where it links E-cadherin to the actin cytoskeleton [33, 34]. β-catenin is a dual-function protein that links E-cadherin to the cytoskeleton and serves as a co-transcriptional activator together with T-cell factor (TCF/LEF) [33, 34]. We determined levels and localization of β-catenin and found that while there was no significant decrease in total cellular levels of β-catenin in EoE patients compared to normal patients, β-catenin’s subcellular distribution was significantly different in EoE compared to normal tissue. Furthermore, while β-catenin is localized mainly at the plasma membrane in the control tissue, it exhibited a more diffuse and distinctly nuclear/perinuclear distribution in EoE samples. This suggests that the β-catenin, released into the cytoplasm upon loss of E-cadherin, subsequently migrates from the cytoplasm to the nucleus, where it necessarily needs to be in order to execute its transcriptional activity. To this end, β-catenin is well established to support increased transcription of factors that promote EMT [35, 36] such as Twist, as well as proliferation, migration, maturation, and angiogenesis.

A hallmark of EMT is a switch in cadherin phenotype, E-cadherin to N-cadherin [10, 33]. We observed an increase in cellular N-cadherin levels in association with the decrease in E-cadherin levels in EoE [14]. The transcription factor Twist1, and others such as SNAIL and zinc-finger E-box-binding protein, are major regulators of EMT that suppresses epithelial gene expression (such as E-cadherin) and induces mesenchymal gene expression (such as N-cadherin) [10, 14, 36, 37]. Many factors upregulate Twist1, a prominent factor being nuclear factor (NF)-κB. Our previous report showed that the pro-inflammatory TNF-α-NFκB pathway was activated in the EoE tissue promoted by the increased nuclear localization of the NFκB p50-p65 heterodimer [18]. Other studies have also indicated that TNFα induces EMT through AKT/GSK or NF-κB-mediated expression of Snail and Twist1 [10, 12, 13, 38–40]. Our present study shows that Twist1 was dramatically induced in EoE tissue. We did not observe any detectable levels of cellular/nuclear Twist1 in normal tissue.

Taken together, this study suggests EMT as a critical pathophysiologic step for the chronic histologic changes noted with EoE development. Based on our observations, we propose the following putative series of events in EoE tissue remodelling: the EMT phenomenon may be induced by chronic inflammation brought about by the EoE disease state, likely secondary to hypersensitivity reaction leading to activation of the aforementioned inflammatory response via the NF-κB or TGF-β pathways [12, 13]. This inflammatory microenvironment may lead to upregulation of transcription factors such as Twist1, which may regulate molecular subprocesses such as the cadherin switch culminating in the obliteration of the adherens and, subsequently, the tight junctions. The release of β-catenin upon the loss of E-cadherin can effectively further potentiate EMT-related remodeling by increasing cellular/nuclear levels of transcription of factors, such as Twist 1, that promote EMT. While the involvement of EMT in the remodeling associated with EoE has been previously described, to the best of our knowledge, this is the first report that shows that the β-catenin/Twist1 network may likely be involved in promoting EMT that is central to the remodeling process. To this end, β-catenin/Twist1/SNAIL network has been previously implicated in EMT associated with airway remodeling and fibrosis [34, 36, 41].

In conclusion, this study strengthens the prior demonstrations by others of the involvement of EMT in supporting and promoting an ongoing epithelial remodeling process in EoE. However, our study shows a novel potential involvement of the transcription factors β-catenin/Twist1 in promoting disease progression. We speculate a putative mechanistic process by which increased expressions and activity of β-catenin/Twist1 resulting from an inflammatory environment (TNF-α/ NFκB) promote the EMT phenotype that disrupts the esophageal epithelial wall and subsequent epithelial remodeling. This relationship gives our findings clinical and potentially translational relevance. While it is unclear from the present literature whether EMT induction is an early step or a later step in disease progression, it seems likely that, following reversible chronic inflammatory changes, induction of EMT is a late-occurring, irreversible pathophysiologic step resulting in tissue remodeling. This would be similar to disease progression in severe asthma. Further research is required to elucidate this detail. If that is the case, EMT markers, such as β-catenin/Twist1 could be useful predictive tools to determine the degree of disease progression and may be useful in furthering our understanding of this condition.

## Supporting information

S1 Table Patient characteristics in EoE and control groups. (PDF)

S1 File Original data for Fig 1B, intercellular space (IS) in esophageal biopsies of normal (0–5 Eos/HPF) and EoE (>15 Eos/HPF) form 20 different patients. (PDF)
