# Supplementary material for: Epithelial-mesenchymal transition, regulated by β-catenin and Twist, leads to esophageal wall remodeling in pediatric eosinophilic esophagitis
Source: PLoS One. 2022 Mar 3;17(3):e0264622. doi: 10.1371/journal.pone.0264622 (PMC8893662; doi:10.1371/journal.pone.0264622)
Supplement: S1 Table — (PDF) [file pone.0264622.s001.pdf]

Supplementary Table 1: Patient characteristics in EoE and control groups.

	**EoE**	**Control**
N value	18	18
Mean age, y	12	9.9
Sex (%)		
Male	14 (78)	11 (61)
Female	4 (22)	7 (39)
Symptoms (%)		
Dysphagia	89	11
Food Impaction	83	5
Heartburn	22	33
Abdominal pain	28	61
Regurgitation	39	62
Atopy	78	5
Endoscopy findings, %		
Loss of vascular pattern	72	22
Vertical furrows	56	0
Concentric ring	17	0
White exudate	30	0
Minor erosive changes	0	27

EoE – eosinophilic esophagitis
