# Supplementary material for: Epithelial-mesenchymal transition, regulated by β-catenin and Twist, leads to esophageal wall remodeling in pediatric eosinophilic esophagitis
Source: PLoS One. 2022 Mar 3;17(3):e0264622. doi: 10.1371/journal.pone.0264622 (PMC8893662; doi:10.1371/journal.pone.0264622)
Supplement: S1 File — (PDF) [file pone.0264622.s002.pdf]

Original data for Figure 1B, intercellular space (IS) in esophageal biopsies of Normal (0-5 Eos/HPF) and EoE (>15 Eos/HPF) form 20 different patients.

Original data for Figure 1B, intercellular space (IS) in esophageal biopsies of Normal (0-5 Eos/HPF) and EoE (>15 Eos/HPF) form 20 different patients.

Normal (0-5 Eos/HPF)

2uM=286.007pixels

Samples:

	angle	length (Pixels)
1	22.932	56.462
2	26.565	44.721
3	26.565	44.721
4	45	53.74
5	15.945	58.241
6	51.34	51.225
7	41.634	48.166
8	4.399	52.154
9	8.531	40.447
10	45	42.426
Average column size:		49.2303

(IS) Average in Eos
(0-5 Eos/HPF)
0.408346

Formula of Average: (Average X 2)/286:
0.354268 **μM**

	angle	length
1	25.974	66.764
2	2.936	48.102
3	-3.731	62.195
4	7.595	60.796
5	15.945	70
6	47.564	64.847
7	-7.431	62.779
8	-8.326	52.873
9	0	74
10	0	80
Average column size:		64.2356

Formula of Average: (Average X 2)/286:
0.4492 **μM**

Original data for Figure 1B, intercellular space (IS) in esophageal biopsies of Normal (0-5 Eos/HPF) and EoE (>15 Eos/HPF) form 20 different patients.

	angle	length		
1	-32.619	59.363		
2	39.806	46.861		
3	2.386	48.042		
4	45	56.569		
5	45	53.74		
6	45	50.912		
7	-29.055	61.774		
8	-23.429	65.391		
9	-15.422	60.166		
10	38.367	51.221	Formula of Average: (Average X 2)/286:	
Average column size:		55.4039	0.38744	**μM**

	angle	length		
1	-31.701	69.925		
2	-31.218	57.175		
3	-33.69	69.322		
4	-33.69	64.9		
5	-29.358	53.43		
6	-88.315	68.029		
7	109.799	53.141		
8	90	52.09		
9	-36.384	64.403		
10	-33.311	63.762	Formula of Average: (Average X 2)/286:	
Average column size:		61.6177	0.430893	**μM**

	angle	length		
1	40.101	59.358		
2	35.256	62.056		
3	-14.036	40.1		
4	4.83	42.506		
5	4.97	72.2		
6	-37.278	68.404		
7	75.964	68.17		
8	-90	61.52		
9	-50.659	57.747		
10	-53.409	100	Formula of Average: (Average X 2)/286:	
Average column size:		63.2061	0.442001	**μM**

Original data for Figure 1B, intercellular space (IS) in esophageal biopsies of Normal (0-5 Eos/HPF) and EoE (>15 Eos/HPF) form 20 different patients.

	angle	length		
1	56.56	30.028		
2	-38.83	75.262		
3	11.725	48.259		
4	1.614	82.056		
5	7.001	64.856		
6	3.652	74.191		
7	61.46	72.769		
8	55.146	76.484		
9	21.297	43.649		
10	-54.866	40.06	Formula of Average: (Average X 2)/286:	
Average column size:		55.23764	0.386277	**μM**

Original data for Figure 1B, intercellular space (IS) in esophageal biopsies of Normal (0-5 Eos/HPF) and EoE (>15 Eos/HPF) form 20 different patients.

EOE (>15 Eos/HPF)

2uM=286.007pixels

(IS) Average in Eos (>15 Eos/HPF)
**0.851434**

Samples:		angle	length
	1	137.603	124.579
	2	149.621	134.462
	3	167.005	133.417
	4	167.989	192.208
	5	53.673	84.404
	6	-33.341	90.973
	7	-26.075	104.652
	8	79.695	89.443
	9	-26.03	95.708
	10	-28.025	140.471
Average column size:			119.0317

Formula of Average: (Average X 2)/286:
0.83239 **µM**

		angle	length
	1	-32.6	104.167
	2	-34.439	119.753
	3	-44.204	103.666
	4	-34.939	128.101
	5	-39.56	118.997
	6	-27.979	124.941
	7	-37.983	175.235
	8	-29.445	142.394
	9	-36.747	187.2
	10	-38.83	110.523
Average column size:			131.4977

Formula of Average: (Average X 2)/286:
0.919564 **µM**

Original data for Figure 1B, intercellular space (IS) in esophageal biopsies of Normal (0-5 Eos/HPF) and EoE (>15 Eos/HPF) form 20 different patients.

		angle	length		
	1	53.616	141.605		
	2	137.911	93.546		
	3	-45	98.995		
	4	-62.354	94.826		
	5	-58.465	103.247		
	6	76.159	142.127		
	7	-55.713	106.508		
	8	-47.49	107.673		
	9	-46.591	101.863		
	10	-33.389	105.394	Formula of Average: (Average X 2)/286:	
Average column size:			109.5784	0.766283	**µM**

		angle	length		
	1	-33.389	105.394		
	2	-35.838	108.814		
	3	-41.82	101.98		
	4	-61.477	104.709		
	5	-53.807	121.607		
	6	-41.285	109.124		
	7	-39.56	119.331		
	8	-48.715	109.124		
	9	-47.663	121.754		
	10	106.504	112.641	Formula of Average: (Average X 2)/286:	
Average column size:			111.4478	0.779355	**µM**

		angle	length		
	1	81.314	145.671		
	2	94.83	142.506		
	3	98.427	109.179		
	4	74.476	149.452		
	5	85.03	138.521		
	6	87.647	146.123		
	7	38.774	116.486		
	8	32.692	139.223		
	9	77.705	119.662		
	10	36.491	100	Formula of Average: (Average X 2)/286:	
Average column size:			130.6823	0.913862	**µM**

Original data for Figure 1B, intercellular space (IS) in esophageal biopsies of Normal (0-5 Eos/HPF) and EoE (>15 Eos/HPF) form 20 different patients.

		angle	length		
	1	-46.102	117.105		
	2	-54.744	102.056		
	3	-46.146	141.45		
	4	-37.03	142.801		
	5	-46.245	130.138		
	6	-35.727	120.428		
	7	-34.796	143.694		
	8	-41.583	119.842		
	9	-51.203	130.874		
	10	-41.987	134.536	Formula of Average: (Average X 2)/286:	
Average column size:			128.2924	0.89715	**μM**

Original data for Figure 1B, intercellular space (IS) in esophageal biopsies of Normal (0-5 Eos/HPF) and EoE (>15 Eos/HPF) form 20 different patients.

Statistics

Column A/	Normal/ EOE
Unpaired t test	
P value	< 0.0001
P value sur	***
Are means	Yes
One- or twc	Two-tailed
t, df	t=13.87 df=10
How big is the difference?	
Mean ± SE	0.4083 ± 0.01545 N=6
Mean ± SE	0.8514 ± 0.02795 N=6
Difference	-0.4431 ± 0.03194
95% confid	-0.5142 to -0.3719
R squared	0.9506
F test to compare variances	
F,DFn, Dfd	3.274, 5, 5
P value	0.219
P value sur	ns
Are varianc	No
F test to compare variances	
F,DFn, Dfd	1.367, 5, 5
P value	0.7402
P value sur	ns
Are varianc	No

Original data for Figure 1D, Quantification of Zonula Occludens-1 analysis of control and eoE esopageal biopsy tissue.

Original data for Figure 1D, Quantification of Zonula Occludens-1 analysis of control and eoE esopageal biopsy tissue.

Values area representative of mean of a samples size n=18 each group. ***p,0.001

High Content Analysis (HCA)

Average Cell Intensity/hpf

Negatice Control	Control	EoE (>15 Eos)
0	0	0
0	0	0
0	0	0
0.008866	0	110.1268
0.011897	0	0
0	0	0
0.007712	0	0
0	0	0
0.01255	0	0
0.009825	0	0
0.015485	0	0
0.017976	0	0
0	0	0
0	0	0
0	0	0
0	0	0
0	0	0
0	0	0
0.009252	0	0
0	0	0
0	0	0
0	0	0
0	0	0
0.006929	0	0
0	0	0
0	0	0
0	0	0
0	0	0
0	0	0
0.01635	0	0
0	0	0
0	0	0
0	0	0
0.006581	0	0
0.008493	0	85.02041
0	0	0
0	0	0
0.005884	0	0
0	0	0
0	0	0
0	0	0
0.019462	0	0
0.013113	0	0
0.013742	0	0
0	0	0
0.015775	0	0
0.013011	0	0
0	0	0
0	0	0

Original data for Figure 1D, Quantification of Zonula Occludens-1 analysis of control and eoE esopageal biopsy tissue.

Values area representative of mean of a samples size n=18 each group. ***p,0.001

0.010463	0	0
0.017525	0	0
0	0	0
0	0	0
0.016919	0	0
0.008896	0	101.4528
0.006114	0	0
0.009186	0	0
109.4507	0	
97.95	0	
0	0	0
0	0	0
0.013436	0	0
0.011127	0	0
0.027495	0	0
0.008398	0	0
0.012679	0	0
0	0	0
0.00602	0	0
0.013633	0	0
0.007618	0	0
0.010508	0	0
0	0	0
0.009394	0	0
0	0	0
0	0	0
0.008866	0	0
0.011897	0	0
0	0	0
0.007712	0	0
0	0	0
0.01255	0	0
0.009825	0	0
0.015485	0	0
0.017976	0	0
0	0	0
0	0	0
0	0	0
0	0	0
0	0	0
0	0	0
0.009252	0	0
0	0	0
0	0	0
0	0	0
0	0	0
0.006929	0	0
0	0	0
0	0	0
0	0	0
0	0	0
0	0	0
0.01635	0	0
0	0	0
0	0	0
0	0	0
0.006581	0	0

Original data for Figure 1D, Quantification of Zonula Occludens-1 analysis of control and eoE esopageal biopsy tissue.

Values area representative of mean of a samples size n=18 each group. ***p,0.001

0.008493	0	0
0	0	0
0	0	0
0.005884	0	0
0	0	0
0	0	0
0	0	0
0.019462	0	0
0.013113	0	0
0.013742	0	0
0	0	0
0.015775	0	0
0.013011	0	0
0	0	0
0	0	0
0.010463	0	0
0.017525	0	0
0	0	0
0	0	0
0.016919	0	0
0.008896	0	0
0.006114	0	0
0.009186	0	0
0.01891	0	0
0.038567	0	0
0	0	0
0.023058	0	0
0.013436	0	0
0.011127	0	0
0.027495	0	0
0.008398	0	0
0.012679	0	0
0	0	0
0.00602	0	0
0.013633	0	0
0.007618	0	0
0.010508	0	0
0	0	0
0	0	0
0	0	0
0	0	0
0	0	0
0	0	0
0	0	0
0	0	0
0	0	0
0	0	0
0	0	0
0	0	0
0	0	0
0	0	0
0	0	0
0	0	0
0	0	0
0	0	0
0	0	0
0	0	0

Original data for Figure 1D, Quantification of Zonula Occludens-1 analysis of control and eoE esopageal biopsy tissue.

Values area representative of mean of a samples size n=18 each group. ***p,0.001

0	0	0
0	0	0
0	0	0
0	0	0
0	0	0
0	0	0
0	0	0
0	0	0
0	0	0
0	0	0
0	0	0
0	0	0
0	0	0
0	0	0
0.010508	0	0
0	0	0
0	0	0
0	0	0
0	0	0
0	0	0
0	0	
0	0	0
0	0	0
0	0	0
0	0	0
0	0	0
0	0	0
0	99.55056	0
0	0	0
0	0	0
0	0	0
0	0	0
0	0	0
0	0	0
0	0	0
0	89.6375	0
0	0	0
0	0	0
0	0	0
0	0	0
0	114.4038	0
0	0	0
0	0	0
0	0	0
0	0	0
0	0	0
0	90.40606	0
0	0	0
0	0	0
0	126.4	0
0	96.5	0
0	0	0
0	0	0
0	107.9412	0
0	97.60317	0
0	89.8913	0
0	0	0

Original data for Figure 1D, Quantification of Zonula Occludens-1 analysis of control and eoE esopageal biopsy tissue.

Values area representative of mean of a samples size n=18 each group. ***p,0.001

0	0	0
0	0	0
0	0	0
0	0	0
0	0	0
0	0	0
0	0	0
0	99.54779	0
0	0	0
0	0	0
0	0	0
0	0	0
0	0	0
0	0	0
0	0	0
0	0	0
0	0	0
0	0	0
0	0	0
0	0	0
0	107.4717	0
0	0	0
0	91.59375	0
0	0	0
0	0	0
0	86.20618	0
0	0	0
0	0	0
0	0	0
0	0	0
0	0	0
0	0	0
0	0	0
0	0	0
0	0	0
0	0	0
0	0	0
0	0	0
0	0	0
0	0	0
0	0	0
0	103.2464	0
0	0	0
0	0	0
0	0	0
0	0	0
0	0	0
0	0	0
0	0	0
0	0	0
0	0	0
0	102.6818	0
0	0	0
0	0	0
0	0	0
0	0	0
0	0	0

Original data for Figure 1D, Quantification of Zonula Occludens-1 analysis of control and eoE esopageal biopsy tissue.

Values area representative of mean of a samples size n=18 each group. ***p,0.001

0	0	0
0	92.66315	0
0	0	0
0	0	0
0	352.2348	0
0	371.7109	0
0	397.119	0
0	132.7293	0
0	351.319	0
0	294.1057	0
0	197.0412	0
0	218.5017	0
0	309.8467	0
0	261.1048	0
0	248.2342	0
0	286.6948	0
0	129.2992	0
0	280.8991	0
0	188.7395	0
0	352.235	0
0	371.711	0
0	397.119	0
0	132.729	0
0	351.319	0
0	294.106	0
0	197.041	0
0	218.502	0
0	309.847	0
0	261.105	0
0	248.234	0
0	286.695	0
0	129.299	0
0	280.899	0
0	188.74	0
0	311.4055	0
0	158.0183	0
0	242.2001	0
0	254.7823	0
0	214.4044	0
0	171.9398	0
0	250.9007	0
0	286.6884	0
0	306.7091	0
0	136.2956	0
0	299.0658	0
0	266.3929	0
0	295.363	0
0	121.2987	0
0	288.0075	0
0	211.0362	0
0	279.136	0
0	314.0647	0
0	195.8567	0
0	150.8945	0
0	281.7386	0
0	222.7844	0
0	245.4654	0

Original data for Figure 1D, Quantification of Zonula Occludens-1 analysis of control and eoE esopageal biopsy tissue.

Values area representative of mean of a samples size n=18 each group, ***p 0.001

0 225.7428 0

Original data for Figure 1D, Quantification of Zonula Occludens-1 analysis of control and eoE esopageal biopsy tissue.

Values area representative of mean of a samples size n=18 each group. ***p,0.001

Statistics:

Table Analyzed	
Column B	0
vs	vs
Column C	>15 EOE
Unpaired t test	
P value	0.001
P value sur	***
Are means	Yes
One- or twc	Two-tailed
t, df	t=3.043 df=562
How big is the difference?	
Mean ± SE	45.87 ± 5.389 N=335
Mean ± SE	24.53 ± 3.123 N=229
Difference	21.34 ± 7.012
95% confid	7.596 to 35.08
R squared	0.01621
F test to compare variances	
F,DFn, Dfd	4.356, 334, 228
P value	P<0.0001
P value sur	***
Are varianc	Yes

Original data for Figure 1F. Quantification of E-Cadherine IF analysis of control and EoE esophageal biopsy tissue.

Original data for Figure 1F. Quantification of E-Cadherine IF analysis of control and EoE esophageal biopsy tissue.

Values are representative of mean of a sample size n=18 for each group.*p<00.1**

High Content Analysis (HCA)

Average Cell Intensity/hpf

Negatice Control	Control	EoE (>15 Eos)
0.009394	260.89	124.3884
0	169.12	199.2963
0	275.213	109.8899
0.008866	215.465	388.1668
0.011897	339.589	180.2577
0	394.222	245.9418
0.007712	202.894	505.0249
0	151.95	174.245
0.01255	358.556	202.7924
0.009825	144.319	269.7285
0.015485	186.252	305.1081
0.017976	129.029	151.3441
0	426.812	844.4361
0	287.572	171.348
0	301.373	218.2052
0	345.952	154.6426
0	285.33	159.6415
0	229.458	297.6749
0.009252	384.332	296.9835
0	371.024	366.0871
0	195.083	524.4441
0	134.08	499.762
0	201.451	124.388
0.006929	414.702	199.296
0	845.765	109.89
0	505.659	388.167
0	294.66	180.258
0	244.011	245.942
0	602.968	505.025
0.01635	184.177	174.245
0	278.053	202.792
0	132.029	269.728
0	298.977	305.108
0.006581	167.057	151.344
0.008493	230.445	844.436
0	217.2613	171.348
0	513.3583	218.205
0.005884	323.2155	154.643
0	290.0894	159.642
0	486.2803	297.675
0	257.3629	296.984
0.019462	503.4935	366.087
0.013113	193.4753	0
0.013742	331.135	0
0	317.6642	0
0.015775	267.4504	0
0.013011	229.4091	0
0	607.0822	0

Original data for Figure 1F. Quantification of E-Cadherine IF analysis of control and EoE esophageal biopsy tissue.
Values are representative of mean of a sample size n=18 for each group.*p<00.1**

0	135	0
0.010463	168.0635	0
0.017525	315.948	0
0	156.809	0
0	465.0683	0
0.016919	217.261	0
0.008896	513.358	0
0.006114	323.215	0
0.009186	290.089	0
0.01891	486.28	0
0.038567	257.363	0
0	503.494	0
0.023058	193.475	0
0.013436	331.135	0
0.011127	317.664	0
0.027495	267.45	0
0.008398	229.409	0
0.012679	607.082	0
0	135	0
0.00602	168.063	0
0.013633	315.948	0
0.007618	156.809	0
0.010508	465.068	0
0		

Original data for Figure 1F. Quantification of E-Cadherine IF analysis of control and EoE esophageal biopsy tissue.

Values are representative of mean of a sample size n=18 for each group.*p<00.1**

Statistics:

E-Cadherin intensity per hpf
(arbitrary units)

Table	E-cadherine ct 0 and m	15
Column B	0	
vs	vs	
Column C	15	
Unpaired t test		
P value	< 0.0001	
P value sur	***	
Are means	Yes	
One- or twc	Two-tailed	
t, df	t=4.999 df=140	
How big is the difference?		
Mean ± SE	306.3 ± 16.81 N=71	
Mean ± SE	165.6 ± 22.57 N=71	
Difference	140.7 ± 28.14	
95% confid	85.54 to 195.9	
R squared	0.1515	
F test to compare variances		
F,DFn, Dfd	1.802, 70, 70	
P value	0.0148	
P value sur	*	
Are varianc	Yes	

Original data for Figure 2B. Quantification of b-Catenin analysis of control and EoE esophageal biopsy tissue

Original data for Figure 2B. Quantification of β-Catenin analysis of control and EoE esophageal biopsy tissue.
Values are representative of mean of sample size n= 18 ech group .*p<001**

	High Content Analysis (HCA) Average Cell Intensity/hpf	
**Negatice Control**	**Control**	**EoE (>15 Eos)**
0.009394	173	111
0	149	150
0	134	146
0.008866	82	117
0.011897	130	123
0	128	117
0.007712	124	114
0	122	135
0.01255	100	100
0.009825	112	112
0.015485	112	112
0.017976	134	143
0	120	135
0	120	126
0	130	211
0	145	112
0	155	111
0	176	110
0.009252	123	120
0	128	121
0	130	100
0	139	130
0	147	140
0.006929	143	123
0	155	124
0	160	122
0	165	112
0	120	134
0	140	135
0.01635	142	131
0	125	129
0	130	128
0	111	123
0.006581	115	134
0.008493	125	145
0	130	125
0	127	124
0.005884	124	111
0	122	112
0	124	123
0	122	122
0.019462	125	124
0.013113	123	135
0.013742	125	126
0	127	127
0.015775	122	128
0.013011	100	129
0	98	111
0	123	124
0.010463	111	133
0.017525	124	143
0	145	123
0	155	126
0.016919	143	130
0.008896	126	131
0.006114	146	127
0.009186	134	122
0.01891	156	127
0.038567	141	130
0	138	131
371.024	140	132
195.0829	137	134
134.0801	149	136
201.4514	148	137
414.702	138	135
84.7653	137	140
0	138	145
0.00602	141	155
0.013633	143	154
0.007618	155	165
0.010508	154	155
0	152	165

Original data for Figure 2B. Quantification of β-Catenin analysis of control and EoE esophageal biopsy tissue.

Values are representative of mean of sample size n= 18 ech group .*p<001**

Statistics:

Table	Data	β-catenin
Column B		
vs	vs	
Column C		
Unpaired t test		
P value	0.2113	
P value sun	ns	
Are means	No	
One- or tw	Two-tailed	
t, df	t=1.256 df=142	
How big is the difference?		
Mean ± SEI	133.2 ± 2.021 N=72	
Mean ± SEI	129.6 ± 1.952 N=72	
Difference	3.528 ± 2.809	
95% confid	-1.979 to 9.034	
R squared	0.01098	
F test to compare variances		
F,DFn, Dfd	1.072, 71, 71	
P value	0.7707	
P value sun	ns	
Are varianc	No	

Original data for Figure 2D, Densitometric analysis of Nuclear/Perinuclear B-catenin in control and EoE patient esophageal biopsy tissue.

Original data for Figure 2D, Densitometric analysis of Nuclear/Perinuclear B-catenin in control and EoE patient esophageal biopsy tissue.
Values are representative of the mean of a sample size N=18. p<0.05**

CONTROL Esophageal biobsy tissue					CONTROL Esophageal biobsy tissue				
Count	Total Area	Average Size			Count	Total Area	Average Size		
Nuclear area					Nuclear area				
1	40	255	255	255	1	10	255	255	255
2	1	255	255	255	2	374	255	255	255
3	119	255	255	255	3	25	255	255	255
4	14	255	255	255	4	12	255	255	255
5	2	255	255	255	5	24	255	255	255
6	1	255	255	255	6	1	255	255	255
7	10	255	255	255	7	18	255	255	255
8	1	255	255	255	8	3	255	255	255
9	2	255	255	255	9	1	255	255	255
10	3	255	255	255	10	4	255	255	255
11	1	255	255	255	11	1	255	255	255
12	2	255	255	255	12	486	255	255	255
13	2	255	255	255	13	7	255	255	255
14	999	255	255	255	14	4	255	255	255
15	24	255	255	255	15	1	255	255	255
16	80	255	255	255	16	1	255	255	255
17	1	255	255	255	17	1	255	255	255
18	6	255	255	255	18	6	255	255	255
19	11	255	255	255	19	19	255	255	255
20	3	255	255	255	20	1	255	255	255
21	2	255	255	255	21	1	255	255	255
22	1	255	255	255	22	80	255	255	255
23	44	255	255	255	23	6	255	255	255
24	62	255	255	255	24	63	255	255	255
25	2	255	255	255	25	22	255	255	255
26	1	255	255	255	26	240	255	255	255
27	13	255	255	255	27	41	255	255	255
28	9	255	255	255	28	1	255	255	255
29	65	255	255	255	29	1	255	255	255
30	20	255	255	255	30	1	255	255	255
31	1	255	255	255	31	22	255	255	255
32	79	255	255	255	32	68	255	255	255
33	1	255	255	255	33	248	255	255	255
34	1	255	255	255	34	2	255	255	255
35	6	255	255	255	35	1	255	255	255
36	22	255	255	255	36	2	255	255	255
37	1	255	255	255	37	2	255	255	255
38	13	255	255	255	38	10	255	255	255
39	78	255	255	255	39	1	255	255	255
40	1	255	255	255	40	238	255	255	255
41	1	255	255	255	41	59	255	255	255
42	4	255	255	255	42	1	255	255	255
43	7	255	255	255	43	3	255	255	255
44	2	255	255	255	44	141	255	255	255
45	1	255	255	255	45	152	255	255	255
46	5	255	255	255	46	2	255	255	255
47	2	255	255	255	47	1	255	255	255
48	1	255	255	255	48	3	255	255	255
49	46	255	255	255	49	37	255	255	255
50	11	255	255	255	50	1	255	255	255
51	1	255	255	255	51	1	255	255	255
52	7	255	255	255	52	61	255	255	255
53	119	255	255	255	53	37	255	255	255
54	31	255	255	255	54	932	255	255	255
55	1	255	255	255	55	744	255	255	255
56	2	255	255	255	56	45	255	255	255
57	1	255	255	255	57	2	255	255	255
58	1	255	255	255	58	3	255	255	255
59	1	255	255	255	59	1	255	255	255
60	5	255	255	255	60	4	255	255	255
61	44	255	255	255	61	2	255	255	255
62	1	255	255	255	62	1	255	255	255
63	5	255	255	255	63	1	255	255	255
64	1	255	255	255	64	1	255	255	255
65	13	255	255	255	65	365	255	255	255
66	54	255	255	255	66	1	255	255	255
67	33	255	255	255	67	2	255	255	255
68	1	255	255	255	68	6	255	255	255
69	1	255	255	255	69	193	255	255	255
70	66	255	255	255	70	3	255	255	255
71	1	255	255	255	71	1	255	255	255
72	37	255	255	255	72	2	255	255	255
73	1	255	255	255	73	6	255	255	255
74	1	255	255	255	74	4	255	255	255
75	265	255	255	255	75	2	255	255	255
76	34	255	255	255	76	4	255	255	255
77	5	255	255	255	77	1	255	255	255
78	5	255	255	255	78	1	255	255	255
79	273	255	255	255	79	1	255	255	255
80	2	255	255	255	80	1	255	255	255
81	3	255	255	255	81	7	255	255	255
82	1	255	255	255	82	1	255	255	255
83	1	255	255	255	83	2	255	255	255
84	1	255	255	255	84	266	255	255	255
85	120	255	255	255	85	2	255	255	255
86	4	255	255	255	86	1	255	255	255
87	264	255	255	255	87	1	255	255	255
88	42	255	255	255	88	2	255	255	255
89	2	255	255	255	89	1	255	255	255
90	1	255	255	255	90	1	255	255	255
91	1	255	255	255	91	115	255	255	255
92	140	255	255	255	92	1	255	255	255
93	1	255	255	255	93	825	255	255	255
94	1	255	255	255	94	26	255	255	255
95	1	255	255	255	95	32	255	255	255
96	1	255	255	255	96	1	255	255	255
97	17	255	255	255	97	1	255	255	255
98	3	255	255	255	98	2	255	255	255
99	1	255	255	255	99	2	255	255	255
100	2	255	255	255	100	2	255	255	255
101	1	255	255	255	101	13	255	255	255
102	2	255	255	255	102	1	255	255	255
103	7	255	255	255	103	1	255	255	255
104	1	255	255	255	104	1	255	255	255
105	1	255	255	255	105	1	255	255	255
106	3	255	255	255	106	95	255	255	255
107	1	255	255	255	107	1116	255	255	255
108	2	255	255	255	108	79	255	255	255
109	1	255	255	255	109	1	255	255	255
110	1	255	255	255	110	18	255	255	255
111	1	255	255	255	111	1	255	255	255
112	1	255	255	255	112	1	255	255	255
113	1	255	255	255	113	68	255	255	255
114	477	255	255	255	114	1	255	255	255
115	2	255	255	255	115	1	255	255	255
116	74	255	255	255	116	1	255	255	255
117	108	255	255	255	117	1	255	255	255
118	298	255	255	255	118	1	255	255	255
119	1	255	255	255	119	3	255	255	255
120	2	255	255	255	120	1	255	255	255
121	1	255	255	255	121	2	255	255	255
122	6	255	255	255	122	1	255	255	255
123	13	255	255	255	123	133	255	255	255
124	416	255	255	255	124	1	255	255	255

Original data for Figure 2D, Densitometric analysis of Nuclear/Perinuclear B-catenin in control and EoE patient esophageal biopsy tissue.

Values are representative of the mean of a sample size N=18. p<0.05**

125	1	255	255	255	125	1	255	255	255
126	2	255	255	255	126	133	255	255	255
127	16	255	255	255	127	2	255	255	255
128	103	255	255	255	128	1	255	255	255
129	40	255	255	255	129	403	255	255	255
130	1	255	255	255	130	58	255	255	255
131	1	255	255	255	131	6	255	255	255
132	9	255	255	255	132	1	255	255	255
133	256	255	255	255	133	137	255	255	255
134	70	255	255	255	134	17	255	255	255
135	8	255	255	255	135	1	255	255	255
136	1	255	255	255	136	1	255	255	255
137	1	255	255	255	137	3	255	255	255
138	221	255	255	255	138	2	255	255	255
139	1	255	255	255	139	2	255	255	255
140	584	255	255	255	140	1	255	255	255
141	1	255	255	255	141	2	255	255	255
142	1	255	255	255	142	25	255	255	255
143	46	255	255	255	143	31	255	255	255
144	41	255	255	255	144	52	255	255	255
145	9	255	255	255	145	42	255	255	255
146	1	255	255	255	146	1	255	255	255
147	6	255	255	255	147	2	255	255	255
148	1	255	255	255	148	1	255	255	255
149	1	255	255	255	149	4	255	255	255
150	777	255	255	255	150	2	255	255	255
151	2	255	255	255	151	1	255	255	255
152	2529	255	255	255	152	33	255	255	255
153	586	255	255	255	153	1	255	255	255
154	1	255	255	255	154	1	255	255	255
155	1	255	255	255	155	1	255	255	255
156	3	255	255	255	156	1	255	255	255
157	1	255	255	255	157	1	255	255	255
158	1	255	255	255	158	70	255	255	255
159	4	255	255	255	159	53	255	255	255
160	10	255	255	255	160	141	255	255	255
161	2	255	255	255	161	1	255	255	255
162	105	255	255	255	162	35	255	255	255
163	1	255	255	255	163	1	255	255	255
164	584	255	255	255	164	35	255	255	255
165	1	255	255	255	165	7	255	255	255
166	1	255	255	255	166	4	255	255	255
167	1	255	255	255	167	15	255	255	255
168	1	255	255	255	168	1	255	255	255
169	1	255	255	255	169	1	255	255	255
170	1	255	255	255	170	9	255	255	255
171	63	255	255	255	171	1	255	255	255
172	21	255	255	255	172	2	255	255	255
173	1	255	255	255	173	1	255	255	255
174	1	255	255	255	174	2	255	255	255
175	1	255	255	255	175	1	255	255	255
176	78	255	255	255	176	166	255	255	255
177	1	255	255	255	177	1	255	255	255
178	1	255	255	255	178	221	255	255	255
179	6	255	255	255	179	2	255	255	255
180	1	255	255	255	180	1	255	255	255
181	1	255	255	255	181	412	255	255	255
182	1	255	255	255	182	1	255	255	255
183	1	255	255	255	183	2	255	255	255
184	59	255	255	255	184	1	255	255	255
185	448	255	255	255	185	1	255	255	255
186	94	255	255	255	186	1	255	255	255
187	67	255	255	255	187	1	255	255	255
188	2	255	255	255	188	10	255	255	255
189	1	255	255	255	189	2	255	255	255
190	1	255	255	255	190	15	255	255	255
191	132	255	255	255	191	3	255	255	255
192	20	255	255	255	192	2	255	255	255
193	27	255	255	255	193	1	255	255	255
194	1	255	255	255	194	1	255	255	255
195	4	255	255	255	195	11	255	255	255
196	2	255	255	255	196	119	255	255	255
197	1	255	255	255	197	2	255	255	255
198	1	255	255	255	198	1	255	255	255
199	1200	255	255	255	199	27	255	255	255
200	1	255	255	255	200	6	255	255	255
201	1	255	255	255	201	2	255	255	255
202	1	255	255	255	202	1	255	255	255
203	764	255	255	255	203	1	255	255	255
204	1	255	255	255	204	1	255	255	255
205	32	255	255	255	205	2	255	255	255
206	2	255	255	255	206	35	255	255	255
207	1	255	255	255	207	1	255	255	255
208	1	255	255	255	208	1	255	255	255
209	9	255	255	255	209	2	255	255	255
210	1	255	255	255	210	2	255	255	255
211	2	255	255	255	211	4	255	255	255
212	1	255	255	255	212	106	255	255	255
213	1	255	255	255	213	1	255	255	255
214	4	255	255	255	214	1	255	255	255
215	1	255	255	255	215	62	255	255	255
216	1	255	255	255	216	2	255	255	255
217	1	255	255	255	217	10	255	255	255
218	1	255	255	255	218	1	255	255	255
219	2	255	255	255	219	6	255	255	255
220	1	255	255	255	220	8	255	255	255
221	1	255	255	255	221	24	255	255	255
222	1	255	255	255	222	4	255	255	255
223	2	255	255	255	223	5	255	255	255
224	1	255	255	255	224	7	255	255	255
225	1	255	255	255	225	2	255	255	255
226	1	255	255	255	226	3	255	255	255
227	1	255	255	255	227	1	255	255	255
228	15	255	255	255	228	1	255	255	255
229	83	255	255	255	229	1	255	255	255
230	2	255	255	255	230	2	255	255	255
231	1	255	255	255	231	14	255	255	255
232	2	255	255	255	232	74	255	255	255
233	1	255	255	255	233	11	255	255	255
234	1	255	255	255	234	1	255	255	255
235	1	255	255	255	235	1	255	255	255
236	3	255	255	255	236	1	255	255	255
237	1	255	255	255	237	1	255	255	255
238	1	255	255	255	238	42	255	255	255
239	1	255	255	255	239	1	255	255	255
240	1	255	255	255	240	26	255	255	255
241	4	255	255	255	241	1	255	255	255
242	2	255	255	255	242	33	255	255	255
243	4	255	255	255	243	1	255	255	255
244	26	255	255	255	244	44	255	255	255
245	3	255	255	255	245	14	255	255	255
246	1	255	255	255	246	15	255	255	255
247	4	255	255	255	247	2	255	255	255
248	587	255	255	255	248	2	255	255	255
249	1	255	255	255	249	59	255	255	255
250	3	255	255	255	250	37	255	255	255
251	1	255	255	255	251	2	255	255	255
252	2	255	255	255	252	2	255	255	255
253	1	255	255	255	253	18	255	255	255
254	1	255	255	255	254	1	255	255	255
255	2	255	255	255	255	9	255	255	255
256	1	255	255	255	256	4	255	255	255
257	119	255	255	255	257	1	255	255	255
258	877	255	255	255	258	81	255	255	255
259	1	255	255	255	259	2	255	255	255
260	16	255	255	255	260	3	255	255	255
261	9	255	255	255	261	1	255	255	255
262	119	255	255	255	262	20	255	255	255

Original data for Figure 2D, Densitometric analysis of Nuclear/Perinuclear B-catenin in control and EoE patient esophageal biopsy tissue.
Values are representative of the mean of a sample size N=18. p<0.05**

263	1	255	255	255	263	2	255	255	255
264	1	255	255	255	264	17	255	255	255
265	1	255	255	255	265	28	255	255	255
266	2	255	255	255	266	67	255	255	255
267	1	255	255	255	267	1	255	255	255
268	8	255	255	255	268	1	255	255	255
269	68	255	255	255	269	3	255	255	255
270	3	255	255	255	270	1	255	255	255
271	1	255	255	255	271	1	255	255	255
272	351	255	255	255	272	19	255	255	255
273	1	255	255	255	273	11	255	255	255
274	102	255	255	255	274	35	255	255	255
275	1	255	255	255	275	458	255	255	255
276	1085	255	255	255	276	2	255	255	255
277	240	255	255	255	277	1	255	255	255
278	1	255	255	255	278	13	255	255	255
279	1	255	255	255	279	1	255	255	255
280	233	255	255	255	280	10	255	255	255
281	1	255	255	255	281	5	255	255	255
282	1	255	255	255	282	1	255	255	255
283	2	255	255	255	283	349	255	255	255
284	6	255	255	255	284	1	255	255	255
285	1	255	255	255	285	83	255	255	255
286	4	255	255	255	286	3	255	255	255
287	1	255	255	255	287	1	255	255	255
288	2	255	255	255	288	124	255	255	255
289	189	255	255	255	289	1	255	255	255
290	1	255	255	255	290	84	255	255	255
291	2	255	255	255	291	2	255	255	255
292	1	255	255	255	292	1	255	255	255
293	1	255	255	255	293	1	255	255	255
294	1	255	255	255	294	1	255	255	255
295	373	255	255	255	295	1	255	255	255
296	4	255	255	255	296	1	255	255	255
297	1	255	255	255	297	2	255	255	255
298	1	255	255	255	298	1	255	255	255
299	1	255	255	255	299	3	255	255	255
300	1848	255	255	255	300	2	255	255	255
301	25	255	255	255	301	2	255	255	255
302	1	255	255	255	302	7	255	255	255
303	4	255	255	255	303	1	255	255	255
304	18	255	255	255	304	1	255	255	255
305	1	255	255	255	305	1	255	255	255
306	2	255	255	255	306	74	255	255	255
307	3	255	255	255	307	1	255	255	255
308	1	255	255	255	308	1	255	255	255
309	3	255	255	255	309	1	255	255	255
310	2	255	255	255	310	1	255	255	255
311	1	255	255	255	311	5	255	255	255
312	49	255	255	255	312	1	255	255	255
313	2	255	255	255	313	1	255	255	255
314	1	255	255	255	314	2	255	255	255
315	1	255	255	255	315	2	255	255	255
316	1	255	255	255	316	1	255	255	255
317	46	255	255	255	317	1	255	255	255
318	4	255	255	255	318	4	255	255	255
319	2	255	255	255	319	1	255	255	255
320	1	255	255	255	320	2	255	255	255
321	3	255	255	255	321	7	255	255	255
322	1	255	255	255	322	1	255	255	255
323	1	255	255	255	323	3	255	255	255
324	2	255	255	255	324	8	255	255	255
325	1	255	255	255	325	3	255	255	255
326	1	255	255	255	326	1	255	255	255
327	3	255	255	255	327	2	255	255	255
328	474	255	255	255	328	1	255	255	255
329	6	255	255	255	329	58	255	255	255
330	41	255	255	255	330	1	255	255	255
331	1	255	255	255	331	1	255	255	255
332	1	255	255	255	332	191	255	255	255
333	1	255	255	255	333	25	255	255	255
334	129	255	255	255	334	9	255	255	255
335	80	255	255	255	335	4	255	255	255
336	1	255	255	255	336	1	255	255	255
337	2	255	255	255	337	20	255	255	255
338	1	255	255	255	338	1	255	255	255
339	2	255	255	255	339	26	255	255	255
340	1	255	255	255	340	5	255	255	255
341	3	255	255	255	341	57	255	255	255
342	2	255	255	255	342	32	255	255	255
343	74	255	255	255	343	19	255	255	255
344	41	255	255	255	344	19	255	255	255
345	4	255	255	255	345	3	255	255	255
346	5	255	255	255	346	1	255	255	255
347	1	255	255	255	347	1	255	255	255
348	54	255	255	255	348	5	255	255	255
349	1	255	255	255	349	17	255	255	255
350	1	255	255	255	350	21	255	255	255
351	22	255	255	255	351	40	255	255	255
352	1	255	255	255	352	2	255	255	255
353	1	255	255	255	353	1	255	255	255
354	1497	255	255	255	354	8	255	255	255
355	1	255	255	255					
356	3	255	255	255					
357	1	255	255	255					
358	1029	255	255	255					
359	1	255	255	255					
360	1	255	255	255					
361	103	255	255	255					
362	1	255	255	255					
363	2	255	255	255					
364	1	255	255	255					
365	2	255	255	255					
366	1	255	255	255					
367	184	255	255	255					
368	1	255	255	255					
369	3	255	255	255					
370	3	255	255	255					
371	10	255	255	255					
372	1	255	255	255					
373	39	255	255	255					
374	1	255	255	255					
375	234	255	255	255					
376	1	255	255	255					
377	264	255	255	255					
378	1	255	255	255					
379	8	255	255	255					
380	3	255	255	255					
381	1242	255	255	255					
382	1	255	255	255					
383	1	255	255	255					
384	8	255	255	255					
385	2	255	255	255					
386	1	255	255	255					
387	2	255	255	255					
388	5	255	255	255					
389	13	255	255	255					
390	15	255	255	255					
391	4	255	255	255					
392	1	255	255	255					
393	5	255	255	255					
394	1	255	255	255					
395	1	255	255	255					
396	1	255	255	255					
397	1	255	255	255					
398	80	255	255	255					
399	1	255	255	255					
400	1	255	255	255					

Original data for Figure 2D, Densitometric analysis of Nuclear/Perinuclear B-catenin in control and EoE patient esophageal biopsy tissue.

Values are representative of the mean of a sample size N=18. p<0.05**

401	27	255	255	255
402	162	255	255	255
403	2	255	255	255
404	17	255	255	255
405	3	255	255	255
406	149	255	255	255
407	2	255	255	255
408	1	255	255	255
409	1	255	255	255
410	2	255	255	255
411	405	255	255	255
412	5	255	255	255
413	8	255	255	255
414	4	255	255	255
415	1	255	255	255
416	1	255	255	255
417	1	255	255	255
418	1	255	255	255
419	6	255	255	255
420	11	255	255	255
421	1	255	255	255
422	1	255	255	255
423	9	255	255	255
424	2	255	255	255
425	1	255	255	255
426	1	255	255	255
427	2	255	255	255
428	1	255	255	255
429	1	255	255	255
430	1	255	255	255
431	7	255	255	255
432	2	255	255	255
433	33	255	255	255
434	4	255	255	255
435	2	255	255	255
436	1	255	255	255
437	1	255	255	255
438	21	255	255	255
439	14	255	255	255
440	1	255	255	255
441	1	255	255	255
442	1	255	255	255
443	1	255	255	255
444	2	255	255	255
445	24	255	255	255
446	1	255	255	255
447	3	255	255	255
448	1	255	255	255
449	3	255	255	255
450	105	255	255	255
451	3	255	255	255
452	68	255	255	255
453	2	255	255	255
454	2	255	255	255
455	1	255	255	255
456	65	255	255	255
457	2	255	255	255
458	381	255	255	255
459	1	255	255	255
460	4	255	255	255
461	1	255	255	255
462	1	255	255	255
463	5	255	255	255
464	53	255	255	255
465	53	255	255	255
466	1	255	255	255
467	8	255	255	255
468	141	255	255	255
469	1	255	255	255
470	2	255	255	255
471	298	255	255	255
472	2	255	255	255
473	2	255	255	255
474	1	255	255	255
475	187	255	255	255
476	114	255	255	255
477	1	255	255	255
478	1	255	255	255
479	40	255	255	255
480	6	255	255	255
481	1	255	255	255
482	43	255	255	255
483	21	255	255	255
484	5	255	255	255
485	3	255	255	255
486	1	255	255	255
487	21	255	255	255
488	3	255	255	255
489	1	255	255	255
490	1	255	255	255
491	1	255	255	255
492	1	255	255	255
493	1	255	255	255
494	1	255	255	255
495	2	255	255	255
496	5	255	255	255
497	11	255	255	255
498	2	255	255	255
499	48	255	255	255
500	1	255	255	255
501	6	255	255	255
502	7	255	255	255
503	1	255	255	255
504	7	255	255	255
505	19	255	255	255
506	1	255	255	255
507	2	255	255	255
508	11	255	255	255
509	1	255	255	255
510	1	255	255	255
511	10	255	255	255
512	5	255	255	255
513	11	255	255	255
514	1	255	255	255
515	1	255	255	255
516	1	255	255	255
517	1	255	255	255
518	2	255	255	255
519	1	255	255	255
520	63	255	255	255
521	1	255	255	255
522	2	255	255	255
523	2	255	255	255
524	1	255	255	255
525	3	255	255	255
526	224	255	255	255
527	17	255	255	255
528	1	255	255	255
529	2	255	255	255
530	6	255	255	255
531	1	255	255	255
532	2	255	255	255
533	1	255	255	255
534	87	255	255	255
535	1	255	255	255
536	1	255	255	255
537	1	255	255	255
538	1	255	255	255

Original data for Figure 2D, Densitometric analysis of Nuclear/Perinuclear B-catenin in control and EoE patient esophageal biopsy tissue.

Values are representative of the mean of a sample size N=18. p<0.05**

539	1	255	255	255
540	1	255	255	255
541	2	255	255	255
542	1	255	255	255
543	1	255	255	255
544	29	255	255	255
545	72	255	255	255
546	418	255	255	255
547	1	255	255	255
548	426	255	255	255
549	1	255	255	255
550	1	255	255	255
551	1	255	255	255
552	399	255	255	255
553	30	255	255	255
554	1	255	255	255
555	1	255	255	255
556	2	255	255	255
557	1	255	255	255
558	27	255	255	255
559	15	255	255	255
560	2	255	255	255
561	1	255	255	255
562	2	255	255	255
563	8	255	255	255
564	1	255	255	255
565	1	255	255	255
566	1	255	255	255
567	5	255	255	255
568	44	255	255	255
569	1	255	255	255
570	2	255	255	255
571	49	255	255	255
572	6	255	255	255
573	1	255	255	255
574	6	255	255	255
575	91	255	255	255
576	336	255	255	255
577	20	255	255	255
578	1	255	255	255
579	1	255	255	255
580	44	255	255	255
581	1	255	255	255
582	5	255	255	255
583	1	255	255	255
584	1	255	255	255
585	14	255	255	255
586	1	255	255	255
587	2	255	255	255
588	1	255	255	255
589	264	255	255	255
590	1	255	255	255
591	7	255	255	255
592	1	255	255	255
593	14	255	255	255
594	40	255	255	255
595	1	255	255	255
596	6	255	255	255
597	1	255	255	255
598	35	255	255	255
599	1	255	255	255
600	88	255	255	255
601	1	255	255	255
602	1	255	255	255
603	1	255	255	255
604	1	255	255	255
605	515	255	255	255
606	196	255	255	255
607	1	255	255	255
608	666	255	255	255
609	1	255	255	255
610	54	255	255	255
611	1	255	255	255
612	1	255	255	255
613	7	255	255	255
614	1	255	255	255
615	738	255	255	255
616	25	255	255	255
617	33	255	255	255
618	2	255	255	255
619	3	255	255	255
620	2	255	255	255
621	6	255	255	255
622	68	255	255	255
623	1	255	255	255
624	1	255	255	255
625	12	255	255	255
626	37	255	255	255
627	2	255	255	255
628	1	255	255	255
629	3	255	255	255
630	52	255	255	255
631	14	255	255	255
632	1	255	255	255
633	1	255	255	255
634	2	255	255	255
635	3	255	255	255
636	1	255	255	255
637	1	255	255	255
638	2	255	255	255
639	1	255	255	255
640	4	255	255	255
641	44	255	255	255
642	16	255	255	255
643	2	255	255	255
644	1	255	255	255
645	21	255	255	255
646	2	255	255	255
647	1	255	255	255
648	8	255	255	255
649	7	255	255	255
650	1	255	255	255
651	7	255	255	255
652	1	255	255	255
653	3	255	255	255
654	19	255	255	255
655	1	255	255	255
656	5	255	255	255
657	51	255	255	255
658	2	255	255	255
659	56	255	255	255
660	1	255	255	255
661	56	255	255	255
662	1	255	255	255
663	1	255	255	255
664	116	255	255	255
665	1	255	255	255
666	1	255	255	255
667	2	255	255	255
668	6	255	255	255
669	2	255	255	255
670	78	255	255	255
671	27	255	255	255
672	2	255	255	255
673	1	255	255	255
674	312	255	255	255
675	2	255	255	255
676	1	255	255	255

Original data for Figure 2D, Densitometric analysis of Nuclear/Perinuclear B-catenin in control and EoE patient esophageal biopsy tissue.
Values are representative of the mean of a sample size N=18. p<0.05**

677	7	255	255	255
678	3	255	255	255
679	1	255	255	255
680	1	255	255	255
681	253	255	255	255
682	1	255	255	255
683	1	255	255	255
684	2	255	255	255
685	1	255	255	255
686	2	255	255	255
687	1	255	255	255
688	1	255	255	255
689	1	255	255	255
690	66	255	255	255
691	2	255	255	255
692	1	255	255	255
693	430	255	255	255
694	1	255	255	255
695	2	255	255	255
696	1	255	255	255
697	2	255	255	255
698	1	255	255	255
699	2	255	255	255
700	53	255	255	255
701	1	255	255	255
702	2	255	255	255
703	35	255	255	255
704	1	255	255	255
705	1	255	255	255
706	1	255	255	255
707	294	255	255	255
708	5	255	255	255
709	1	255	255	255
710	434	255	255	255
711	12	255	255	255
712	6	255	255	255
713	156	255	255	255
714	1	255	255	255
715	5	255	255	255
716	1	255	255	255
717	171	255	255	255
718	1	255	255	255
719	292	255	255	255
720	1	255	255	255
721	4	255	255	255
722	208	255	255	255
723	1	255	255	255
724	8	255	255	255
725	52	255	255	255
726	2	255	255	255
727	2	255	255	255
728	14	255	255	255
729	2	255	255	255
730	1	255	255	255
731	2	255	255	255
732	1	255	255	255
733	78	255	255	255
734	1	255	255	255
735	1	255	255	255
736	1	255	255	255
737	1	255	255	255
738	1	255	255	255
739	933	255	255	255
740	4	255	255	255
741	3	255	255	255
742	14	255	255	255
743	1	255	255	255
744	1	255	255	255
745	1	255	255	255
746	662	255	255	255
747	1	255	255	255
748	1	255	255	255
749	1	255	255	255
750	285	255	255	255
751	120	255	255	255
752	69	255	255	255
753	1	255	255	255
754	4	255	255	255
755	2	255	255	255
756	2	255	255	255
757	1	255	255	255
758	1	255	255	255
759	1	255	255	255
760	10	255	255	255
761	1	255	255	255
762	3	255	255	255
763	174	255	255	255
764	4	255	255	255
765	1	255	255	255
766	1	255	255	255
767	95	255	255	255
768	127	255	255	255
769	5	255	255	255
770	1	255	255	255
771	1	255	255	255
772	1	255	255	255
773	296	255	255	255
774	86	255	255	255
775	2	255	255	255
776	106	255	255	255
777	530	255	255	255
778	6	255	255	255
779	2	255	255	255
780	1	255	255	255
781	1	255	255	255
782	2	255	255	255
783	1	255	255	255
784	52	255	255	255
785	475	255	255	255
786	1	255	255	255
787	54	255	255	255
788	1	255	255	255
789	3	255	255	255
790	1	255	255	255
791	36	255	255	255
792	1	255	255	255
793	2	255	255	255
794	1	255	255	255
795	114	255	255	255
796	30	255	255	255
797	2	255	255	255
798	1	255	255	255
799	1	255	255	255
800	1	255	255	255
801	319	255	255	255
802	24	255	255	255
803	2	255	255	255
804	1	255	255	255
805	13	255	255	255
806	77	255	255	255
807	1	255	255	255
808	437	255	255	255
809	93	255	255	255
810	12	255	255	255
811	126	255	255	255
812	64	255	255	255
813	1	255	255	255
814	6	255	255	255

Original data for Figure 2D, Densitometric analysis of Nuclear/Perinuclear B-catenin in control and EoE patient esophageal biopsy tissue.
Values are representative of the mean of a sample size N=18. p<0.05**

815	1	255	255	255
816	52	255	255	255
817	13	255	255	255
818	25	255	255	255
819	151	255	255	255
820	2	255	255	255
821	1	255	255	255
822	2	255	255	255
823	140	255	255	255
824	3	255	255	255
825	1	255	255	255
826	5	255	255	255
827	3	255	255	255
828	21	255	255	255
829	1	255	255	255
830	14	255	255	255
831	1	255	255	255
832	2	255	255	255
833	3	255	255	255
834	1	255	255	255
835	59	255	255	255
836	35	255	255	255
837	128	255	255	255
838	161	255	255	255
839	1	255	255	255
840	4	255	255	255
841	2	255	255	255
842	8	255	255	255
843	1	255	255	255
844	704	255	255	255
845	75	255	255	255
846	1	255	255	255
847	1	255	255	255
848	1	255	255	255
849	1	255	255	255
850	1	255	255	255
851	2	255	255	255
852	118	255	255	255
853	1	255	255	255
854	22	255	255	255
855	22	255	255	255
856	2	255	255	255
857	1	255	255	255
858	74	255	255	255
859	756	255	255	255
860	1	255	255	255
861	1	255	255	255
862	1	255	255	255
863	1	255	255	255
864	3	255	255	255
865	16	255	255	255
866	38	255	255	255
867	95	255	255	255
868	7	255	255	255
869	822	255	255	255
870	1	255	255	255
871	971	255	255	255
872	33	255	255	255
873	1	255	255	255
874	1	255	255	255
875	119	255	255	255
876	38	255	255	255
877	2	255	255	255
878	1	255	255	255
879	2	255	255	255
880	4	255	255	255
881	2	255	255	255
882	2	255	255	255
883	35	255	255	255
884	1	255	255	255
885	45	255	255	255
886	1	255	255	255
887	3	255	255	255
888	675	255	255	255
889	1	255	255	255
890	143	255	255	255
891	1	255	255	255
892	579	255	255	255
893	57	255	255	255

Original data for Figure 2D, Densitometric analysis of Nuclear/Perinuclear B-catenin in control and EoE patient esophageal biopsy tissue.
Values are representative of the mean of a sample size N=18. p<0.05**

CONTROL
Esophageal biobsy tissue

CONTROL
Esophageal biobsy tissue

	Count	Total Area	Average Size	
	Nuclear area			
1	1	255	255	255
2	16	255	255	255
3	1	255	255	255
4	1	255	255	255
5	3	255	255	255
6	6	255	255	255
7	3	255	255	255
8	1	255	255	255
9	29	255	255	255
10	1	255	255	255
11	14	255	255	255
12	1	255	255	255
13	12	255	255	255
14	28	255	255	255
15	2	255	255	255
16	1	255	255	255
17	3	255	255	255
18	2	255	255	255
19	3	255	255	255
20	1	255	255	255
21	6	255	255	255
22	2	255	255	255
23	1	255	255	255
24	1	255	255	255
25	3	255	255	255
26	1	255	255	255
27	26	255	255	255
28	1	255	255	255
29	1	255	255	255
30	1	255	255	255
31	7	255	255	255
32	1	255	255	255
33	1	255	255	255
34	1	255	255	255
35	1	255	255	255
36	1	255	255	255
37	4	255	255	255
38	2	255	255	255
39	2	255	255	255
40	1	255	255	255
41	2	255	255	255
42	1	255	255	255
43	2	255	255	255
44	1	255	255	255
45	19	255	255	255
46	4	255	255	255
47	2	255	255	255
48	1	255	255	255
49	2	255	255	255
50	1	255	255	255
51	7	255	255	255
52	1	255	255	255
53	4	255	255	255
54	1	255	255	255
55	20	255	255	255
56	1	255	255	255
57	3	255	255	255
58	9	255	255	255
59	2	255	255	255
60	1	255	255	255
61	1	255	255	255
62	18	255	255	255
63	1	255	255	255
64	1	255	255	255
65	2	255	255	255
66	1	255	255	255
67	7	255	255	255
68	3	255	255	255
69	14	255	255	255
70	1	255	255	255
71	3	255	255	255
72	1	255	255	255
73	4	255	255	255
74	1	255	255	255
75	1	255	255	255
76	1	255	255	255
77	1	255	255	255
78	1	255	255	255
79	1	255	255	255
80	3	255	255	255
81	4	255	255	255
82	1	255	255	255
83	2	255	255	255
84	1	255	255	255
85	9	255	255	255
86	2	255	255	255
87	5	255	255	255
88	2	255	255	255
89	1	255	255	255
90	1	255	255	255
91	12	255	255	255
92	2	255	255	255
93	25	255	255	255
94	1	255	255	255
95	33	255	255	255
96	2	255	255	255
97	16	255	255	255
98	4	255	255	255
99	1	255	255	255
100	6	255	255	255
101	3	255	255	255
102	2	255	255	255
103	1	255	255	255
104	8	255	255	255
105	8	255	255	255
106	2	255	255	255
107	1	255	255	255
108	3	255	255	255
109	1	255	255	255
110	30	255	255	255
111	4	255	255	255
112	2	255	255	255
113	9	255	255	255
114	2	255	255	255
115	2	255	255	255
116	6	255	255	255
117	1	255	255	255
118	1	255	255	255
119	4	255	255	255
120	2	255	255	255
121	3	255	255	255
122	2	255	255	255
123	8	255	255	255
124	2	255	255	255

CONTROL
Esophageal biobsy tissue

	Count	Total Area	Average Size
	Nuclear area		
1	1	255	255
2	16	255	255
3	1	255	255
4	1	255	255
5	3	255	255
6	6	255	255
7	3	255	255
8	1	255	255
9	29	255	255
10	1	255	255
11	14	255	255
12	1	255	255
13	12	255	255
14	28	255	255
15	2	255	255
16	1	255	255
17	3	255	255
18	2	255	255
19	3	255	255
20	1	255	255
21	6	255	255
22	2	255	255
23	1	255	255
24	1	255	255
25	3	255	255
26	1	255	255
27	26	255	255
28	1	255	255
29	1	255	255
30	1	255	255
31	7	255	255
32	1	255	255
33	1	255	255
34	1	255	255
35	1	255	255
36	1	255	255
37	4	255	255
38	2	255	255
39	2	255	255
40	1	255	255
41	2	255	255
42	1	255	255
43	2	255	255
44	1	255	255
45	19	255	255
46	4	255	255
47	2	255	255
48	1	255	255
49	2	255	255
50	1	255	255
51	7	255	255
52	1	255	255
53	4	255	255
54	1	255	255
55	20	255	255
56	1	255	255
57	3	255	255
58	9	255	255
59	2	255	255
60	1	255	255
61	1	255	255
62	18	255	255
63	1	255	255
64	1	255	255
65	2	255	255
66	1	255	255
67	7	255	255
68	3	255	255
69	14	255	255
70	1	255	255
71	3	255	255
72	1	255	255
73	4	255	255
74	1	255	255
75	1	255	255
76	1	255	255
77	1	255	255
78	1	255	255
79	1	255	255
80	3	255	255
81	4	255	255
82	1	255	255
83	2	255	255
84	1	255	255
85	9	255	255
86	2	255	255
87	5	255	255
88	2	255	255
89	1	255	255
90	1	255	255
91	12	255	255
92	2	255	255
93	25	255	255
94	1	255	255
95	33	255	255
96	2	255	255
97	16	255	255
98	4	255	255
99	1	255	255
100	6	255	255
101	3	255	255
102	2	255	255
103	1	255	255
104	8	255	255
105	8	255	255
106	2	255	255
107	1	255	255
108	3	255	255
109	1	255	255
110	30	255	255
111	4	255	255
112	2	255	255
113	9	255	255
114	2	255	255
115	2	255	255
116	6	255	255
117	1	255	255
118	1	255	255
119	4	255	255
120	2	255	255
121	3	255	255
122	2	255	255
123	8	255	255
124	2	255	255

Original data for Figure 2D, Densitometric analysis of Nuclear/Perinuclear B-catenin in control and EoE patient esophageal biopsy tissue.

Values are representative of the mean of a sample size N=18. p<0.05**

125	8	255	255	255	125	8	255	255
126	4	255	255	255	126	4	255	255
127	1	255	255	255	127	1	255	255
128	1	255	255	255	128	1	255	255
129	1	255	255	255	129	1	255	255
130	3	255	255	255	130	3	255	255
131	1	255	255	255	131	1	255	255
132	14	255	255	255	132	14	255	255
133	2	255	255	255	133	2	255	255
134	3	255	255	255	134	3	255	255
135	8	255	255	255	135	8	255	255
136	4	255	255	255	136	4	255	255
137	19	255	255	255	137	19	255	255
138	7	255	255	255	138	7	255	255
139	2	255	255	255	139	2	255	255
140	53	255	255	255	140	53	255	255
141	1	255	255	255	141	1	255	255
142	3	255	255	255	142	3	255	255
143	1	255	255	255	143	1	255	255
144	1	255	255	255	144	1	255	255
145	3	255	255	255	145	3	255	255
146	38	255	255	255	146	38	255	255
147	1	255	255	255	147	1	255	255
148	7	255	255	255	148	7	255	255
149	12	255	255	255	149	12	255	255
150	1	255	255	255	150	1	255	255
151	1	255	255	255	151	1	255	255
152	3	255	255	255	152	3	255	255
153	7	255	255	255	153	7	255	255
154	2	255	255	255	154	2	255	255
155	1	255	255	255	155	1	255	255
156	1	255	255	255	156	1	255	255
157	2	255	255	255	157	2	255	255
158	1	255	255	255	158	1	255	255
159	7	255	255	255	159	7	255	255
160	12	255	255	255	160	12	255	255
161	1	255	255	255	161	1	255	255
162	16	255	255	255	162	16	255	255
163	1	255	255	255	163	1	255	255
164	2	255	255	255	164	2	255	255
165	6	255	255	255	165	6	255	255
166	4	255	255	255	166	4	255	255
167	1	255	255	255	167	1	255	255
168	1	255	255	255	168	1	255	255
169	24	255	255	255	169	24	255	255
170	18	255	255	255	170	18	255	255
171	1	255	255	255	171	1	255	255
172	11	255	255	255	172	11	255	255
173	15	255	255	255	173	15	255	255
174	1	255	255	255	174	1	255	255
175	2	255	255	255	175	2	255	255
176	1	255	255	255	176	1	255	255
177	4	255	255	255	177	4	255	255
178	3	255	255	255	178	3	255	255
179	2	255	255	255	179	2	255	255
180	24	255	255	255	180	24	255	255
181	1	255	255	255	181	1	255	255
182	2	255	255	255	182	2	255	255
183	2	255	255	255	183	2	255	255
184	1	255	255	255	184	1	255	255
185	1	255	255	255	185	1	255	255
186	1	255	255	255	186	1	255	255
187	19	255	255	255	187	19	255	255
188	4	255	255	255	188	4	255	255
189	1	255	255	255	189	1	255	255
190	1	255	255	255	190	1	255	255
191	2	255	255	255	191	2	255	255
192	2	255	255	255	192	2	255	255
193	22	255	255	255	193	22	255	255
194	1	255	255	255	194	1	255	255
195	5	255	255	255	195	5	255	255
196	1	255	255	255	196	1	255	255
197	12	255	255	255	197	12	255	255
198	1	255	255	255	198	1	255	255
199	14	255	255	255	199	14	255	255
200	1	255	255	255	200	1	255	255
201	2	255	255	255	201	2	255	255
202	1	255	255	255	202	1	255	255
202	46	255	255	255	203	13	255	255
203	1	255	255	255	204	7	255	255
204	1	255	255	255	205	2	255	255
205	29	255	255	255	206	1	255	255
206	1	255	255	255	207	2	255	255
207	41	255	255	255	208	104	255	255
208	558	255	255	255	209	2	255	255
209	1	255	255	255	210	2	255	255
210	1	255	255	255	211	1	255	255
211	149	255	255	255	212	1	255	255
212	1	255	255	255	213	2	255	255
213	1	255	255	255	214	10	255	255
214	9	255	255	255	215	1	255	255
215	1	255	255	255	216	49	255	255
216	34	255	255	255	217	10	255	255
217	1	255	255	255	218	2	255	255
218	3	255	255	255	219	538	255	255
219	73	255	255	255	220	5	255	255
220	203	255	255	255	221	1	255	255
221	1	255	255	255	222	1	255	255
222	3	255	255	255	223	2	255	255
223	12	255	255	255	224	1	255	255
224	61	255	255	255	225	5	255	255
225	14	255	255	255	226	523	255	255
226	1	255	255	255	227	2	255	255
227	7	255	255	255	228	1	255	255
228	2	255	255	255	229	135	255	255
229	1	255	255	255	230	191	255	255
230	1	255	255	255	231	205	255	255
231	1	255	255	255	232	3	255	255
232	297	255	255	255	233	51	255	255
233	4	255	255	255	234	59	255	255
234	1	255	255	255	235	136	255	255
235	1	255	255	255	236	5	255	255
236	1	255	255	255	237	9	255	255
237	84	255	255	255	238	1	255	255
238	2	255	255	255	239	1	255	255
239	1	255	255	255	240	2	255	255
240	1	255	255	255	241	2	255	255
241	127	255	255	255	242	1	255	255
242	1	255	255	255	243	37	255	255
243	330	255	255	255	244	179	255	255
244	1	255	255	255	245	1	255	255
245	1	255	255	255	246	111	255	255
246	1	255	255	255	247	6	255	255
247	1	255	255	255	248	1	255	255
248	1	255	255	255	249	34	255	255
249	1	255	255	255	250	2	255	255
250	2	255	255	255	251	193	255	255
251	1	255	255	255	252	243	255	255
252	90	255	255	255	253	1	255	255
253	3	255	255	255	254	254	255	255
254	10	255	255	255	255	1	255	255
255	1	255	255	255	256	311	255	255
256	2	255	255	255	257	3	255	255
257	289	255	255	255	258	2	255	255
258	26	255	255	255	259	6	255	255
259	1	255	255	255	260	537	255	255
260	121	255	255	255	261	1	255	255
261	195	255	255	255	262	1	255	255

Original data for Figure 2D, Densitometric analysis of Nuclear/Perinuclear B-catenin in control and EoE patient esophageal biopsy tissue.

Values are representative of the mean of a sample size N=18. p<0.05**

262	1	255	255	255
263	1	255	255	255
264	1	255	255	255
265	59	255	255	255
266	2	255	255	255
270	4	255	255	255
271	3	255	255	255
272	1	255	255	255
273	10	255	255	255
274	1	255	255	255
275	698	255	255	255
276	1	255	255	255
277	668	255	255	255
278	3	255	255	255
279	17	255	255	255
280	5	255	255	255
281	1	255	255	255
282	3	255	255	255
283	25	255	255	255
284	129	255	255	255
285	2	255	255	255
286	1	255	255	255
287	1	255	255	255
288	1	255	255	255
289	1	255	255	255
290	2	255	255	255
291	1	255	255	255
292	11	255	255	255
293	43	255	255	255
294	2	255	255	255
295	5	255	255	255
296	2	255	255	255
297	2	255	255	255
298	1	255	255	255
299	3	255	255	255
300	476	255	255	255

263	192	255	255
264	1	255	255
265	1	255	255
266	1	255	255
267	1	255	255
268	7	255	255
269	2	255	255
270	1	255	255
271	1	255	255
272	38	255	255
273	1	255	255
274	1	255	255
275	916	255	255
276	4	255	255
277	53	255	255
278	54	255	255
279	1	255	255
280	2	255	255
281	2	255	255
282	16	255	255
283	9	255	255
284	2	255	255
285	1	255	255
286	1	255	255
287	7	255	255
288	109	255	255
289	161	255	255
290	69	255	255
291	1	255	255
292	472	255	255
293	1	255	255
294	2	255	255
295	1	255	255
296	1	255	255
297	37	255	255
298	1	255	255
299	1	255	255
300	12	255	255
301	1	255	255
302	1	255	255
303	34	255	255
304	79	255	255
305	358	255	255
306	3	255	255
307	1	255	255
308	1	255	255
309	380	255	255
310	267	255	255
311	1	255	255
312	1	255	255
313	1	255	255
314	1	255	255
315	1	255	255
316	68	255	255
317	94	255	255
318	1	255	255
319	70	255	255
320	231	255	255
321	2	255	255
322	22	255	255
323	2	255	255
324	1	255	255
325	5	255	255
326	1	255	255
327	68	255	255
328	1	255	255
329	1	255	255
330	1473	255	255
331	63	255	255
332	1	255	255
333	38	255	255
334	3	255	255
335	2	255	255
336	31	255	255
337	2	255	255
338	1	255	255
339	3	255	255
340	130	255	255
341	1	255	255
342	4	255	255
343	587	255	255
344	2	255	255
345	56	255	255
346	2	255	255
347	1	255	255
348	1	255	255
349	12	255	255
350	1	255	255
351	1	255	255
352	250	255	255
353	1	255	255
354	3	255	255
355	3	255	255
356	103	255	255
357	1	255	255
358	3	255	255
359	2	255	255
360	13	255	255
361	1	255	255
362	4	255	255
363	3	255	255
364	3	255	255
365	654	255	255
366	1	255	255
367	1	255	255
368	25	255	255
369	1	255	255
370	599	255	255
371	40	255	255
372	1	255	255
373	1	255	255
374	1	255	255
375	2	255	255
376	4	255	255
377	1	255	255
378	1	255	255
379	2	255	255
380	3	255	255
381	2	255	255
382	1	255	255
383	3209	255	255
384	1	255	255
385	1	255	255
386	3	255	255
387	1	255	255
388	1	255	255
389	2	255	255
390	1	255	255
391	1	255	255
392	1	255	255
393	1	255	255
394	1	255	255
395	2	255	255
396	1	255	255
397	1	255	255
398	2	255	255
399	1	255	255
400	3	255	255

Original data for Figure 2D, Densitometric analysis of Nuclear/Perinuclear B-catenin in control and EoE patient esophageal biopsy tissue.
Values are representative of the mean of a sample size N=18. p<0.05**

401	334	255	255
402	1	255	255
403	1	255	255
404	1	255	255
405	2	255	255
406	2	255	255
407	63	255	255
408	2	255	255
409	11	255	255
410	1	255	255
411	1	255	255
412	37	255	255
413	3	255	255
414	7	255	255
415	118	255	255
416	1	255	255
417	40	255	255
418	1	255	255
419	2	255	255
420	2	255	255
421	1	255	255
422	27	255	255
423	4	255	255
424	1	255	255
425	1	255	255
426	1	255	255
427	70	255	255
428	1	255	255
429	55	255	255
430	3	255	255
431	5	255	255
432	1	255	255
433	1	255	255
434	2	255	255
435	2	255	255
436	2	255	255
437	1	255	255

Original data for Figure 2D, Densitometric analysis of Nuclear/Perinuclear B-catenin in control and EoE patient esophageal biopsy tissue.
Values are representative of the mean of a sample size N=18. p<0.05**

CONTROL
Esophageal biobsy tissue

Count	Total Area	Average Size		
Nuclear Area				
1	3	255	255	
2	1	255	255	255
3	197	255	255	255
4	2	255	255	255
5	1	255	255	255
6	4	255	255	255
7	2	255	255	255
8	1	255	255	255
9	1	255	255	255
10	3	255	255	255
11	20	255	255	255
12	1	255	255	255
13	8	255	255	255
14	83	255	255	255
15	146	255	255	255
16	100	255	255	255
17	1	255	255	255
18	1	255	255	255
19	4	255	255	255
20	8	255	255	255
21	4	255	255	255
22	1	255	255	255
23	2	255	255	255
24	14	255	255	255
25	130	255	255	255
26	4	255	255	255
27	157	255	255	255
28	6	255	255	255
29	1	255	255	255
30	1	255	255	255
31	2	255	255	255
32	3	255	255	255
33	1	255	255	255
34	1	255	255	255
35	1	255	255	255
36	4	255	255	255
37	5	255	255	255
38	1	255	255	255
39	238	255	255	255
40	2	255	255	255
41	2	255	255	255
42	57	255	255	255
43	1	255	255	255
44	3	255	255	255
45	2	255	255	255
46	2	255	255	255
47	3	255	255	255
48	1	255	255	255
49	1	255	255	255
50	3	255	255	255
51	2	255	255	255
52	2	255	255	255
53	2	255	255	255
54	3	255	255	255
55	1	255	255	255
56	411	255	255	255
57	8	255	255	255
58	1	255	255	255
59	1	255	255	255
60	3	255	255	255
61	31	255	255	255
62	8	255	255	255
63	5	255	255	255
64	1	255	255	255
65	3	255	255	255
66	1	255	255	255
67	1	255	255	255
68	2	255	255	255
69	3	255	255	255
70	3	255	255	255
71	1	255	255	255
72	2	255	255	255
73	1	255	255	255
74	1	255	255	255
75	1	255	255	255
76	8	255	255	255
77	25	255	255	255
78	2	255	255	255
79	1	255	255	255
80	6	255	255	255
81	2	255	255	255
82	1	255	255	255
83	4	255	255	255
84	3	255	255	255
85	1	255	255	255
86	1	255	255	255
87	1	255	255	255
88	5	255	255	255
89	1	255	255	255
90	1	255	255	255
91	52	255	255	255
92	8	255	255	255
93	99	255	255	255
94	1	255	255	255
95	2	255	255	255
96	864	255	255	255
97	1	255	255	255
98	1	255	255	255
99	1	255	255	255
100	2	255	255	255
101	1	255	255	255
102	1	255	255	255
103	2	255	255	255
104	2	255	255	255
105	1	255	255	255
106	1	255	255	255
107	2	255	255	255
108	1	255	255	255
109	1	255	255	255
110	1	255	255	255
111	1	255	255	255
112	1	255	255	255
113	1	255	255	255
114	1	255	255	255
115	1	255	255	255
116	1	255	255	255
117	3	255	255	255
118	10	255	255	255
119	9	255	255	255
120	1	255	255	255
121	541	255	255	255
122	1	255	255	255
123	13	255	255	255
124	2	255	255	255

CONTROL
Esophageal biobsy tissue

Count	Total Area	Average Size		
Nuclear Area				
1	164	255	255	255
2	1	255	255	255
3	417	255	255	255
4	1	255	255	255
5	1	255	255	255
6	1	255	255	255
7	1	255	255	255
8	1	255	255	255
9	352	255	255	255
10	2	255	255	255
11	1	255	255	255
12	141	255	255	255
13	51	255	255	255
14	2	255	255	255
15	3	255	255	255
16	42	255	255	255
17	1	255	255	255
18	2	255	255	255
19	2	255	255	255
20	10	255	255	255
21	1	255	255	255
22	1	255	255	255
23	293	255	255	255
24	6	255	255	255
25	25	255	255	255
26	1	255	255	255
27	3	255	255	255
28	2	255	255	255
29	36	255	255	255
30	2	255	255	255
31	2	255	255	255
32	3	255	255	255
33	12	255	255	255
34	2	255	255	255
35	2	255	255	255
36	1	255	255	255
37	5	255	255	255
38	1	255	255	255
39	1	255	255	255
40	70	255	255	255
41	1	255	255	255
42	2	255	255	255
43	1	255	255	255
44	1	255	255	255
45	1	255	255	255
46	3	255	255	255
47	1	255	255	255
48	58	255	255	255
49	138	255	255	255
50	755	255	255	255
51	1	255	255	255
52	1	255	255	255
53	2	255	255	255
54	2	255	255	255
55	3	255	255	255
56	21	255	255	255
57	2	255	255	255
58	4	255	255	255
59	1	255	255	255
60	12	255	255	255
61	3	255	255	255
62	3	255	255	255
63	41	255	255	255
64	1	255	255	255
65	5	255	255	255
66	1	255	255	255
67	1	255	255	255
68	1	255	255	255
69	2	255	255	255
70	7	255	255	255
71	5	255	255	255
72	1	255	255	255
73	1	255	255	255
74	101	255	255	255
75	3	255	255	255
76	2	255	255	255
77	1	255	255	255
78	1	255	255	255
79	1	255	255	255
80	1	255	255	255
81	1	255	255	255
82	3	255	255	255
83	1	255	255	255
84	7	255	255	255
85	3	255	255	255
86	1	255	255	255
87	10	255	255	255
88	13	255	255	255
89	3	255	255	255
90	3	255	255	255
91	2	255	255	255
92	2	255	255	255
93	14	255	255	255
94	2	255	255	255
95	3	255	255	255
96	4	255	255	255
97	1	255	255	255
98	8	255	255	255
99	2	255	255	255
100	2	255	255	255
101	1	255	255	255
102	1	255	255	255
103	2	255	255	255
104	1	255	255	255
105	14	255	255	255
106	77	255	255	255
107	1	255	255	255
108	6	255	255	255
109	1	255	255	255
110	1	255	255	255
111	35	255	255	255
112	1	255	255	255
113	2	255	255	255
114	1	255	255	255
115	2	255	255	255
116	2	255	255	255
117	2	255	255	255
118	5	255	255	255
119	176	255	255	255
120	1	255	255	255
121	6	255	255	255
122	1	255	255	255
123	1	255	255	255
124	10	255	255	255

Original data for Figure 2D, Densitometric analysis of Nuclear/Perinuclear B-catenin in control and EoE patient esophageal biopsy tissue.

Values are representative of the mean of a sample size N=18. p<0.05**

125	14	255	255	255	125	3	255	255	255
126	1	255	255	255	126	2	255	255	255
127	1	255	255	255	127	1	255	255	255
128	1	255	255	255	128	3	255	255	255
129	2	255	255	255	129	3	255	255	255
130	1	255	255	255	130	1	255	255	255
131	1	255	255	255	131	1	255	255	255
132	1	255	255	255	132	1	255	255	255
133	7	255	255	255	133	47	255	255	255
134	3	255	255	255	134	234	255	255	255
135	2	255	255	255	135	7	255	255	255
136	7	255	255	255	136	1	255	255	255
137	5	255	255	255	137	1	255	255	255
138	4	255	255	255	138	2	255	255	255
139	1	255	255	255	139	111	255	255	255
140	2	255	255	255	140	10	255	255	255
141	4	255	255	255	141	2	255	255	255
142	1	255	255	255	142	2	255	255	255
143	4	255	255	255	143	214	255	255	255
144	1	255	255	255	144	1	255	255	255
145	1	255	255	255	145	34	255	255	255
146	1	255	255	255	146	13	255	255	255
147	19	255	255	255	147	1	255	255	255
148	22	255	255	255	148	2	255	255	255
149	104	255	255	255	149	1	255	255	255
150	54	255	255	255	150	27	255	255	255
151	1	255	255	255	151	1	255	255	255
152	1	255	255	255	152	1	255	255	255
153	1	255	255	255	153	2	255	255	255
154	1	255	255	255	154	1	255	255	255
155	63	255	255	255	155	1	255	255	255
156	5	255	255	255	156	3	255	255	255
157	578	255	255	255	157	39	255	255	255
158	1	255	255	255	158	1	255	255	255
159	1	255	255	255	159	1	255	255	255
160	14	255	255	255	160	1	255	255	255
161	1	255	255	255	161	332	255	255	255
162	1	255	255	255	162	1	255	255	255
163	2	255	255	255	163	1	255	255	255
164	1	255	255	255	164	1	255	255	255
165	8	255	255	255	165	5	255	255	255
166	1	255	255	255	166	1	255	255	255
167	6	255	255	255	167	2	255	255	255
168	1	255	255	255	168	5	255	255	255
169	1	255	255	255	169	19	255	255	255
170	1	255	255	255	170	1	255	255	255
171	1	255	255	255	171	127	255	255	255
172	1	255	255	255	172	1	255	255	255
173	1	255	255	255	173	1	255	255	255
174	1	255	255	255	174	1	255	255	255
175	1	255	255	255	175	1	255	255	255
176	6	255	255	255	176	1	255	255	255
177	11	255	255	255	177	94	255	255	255
178	1	255	255	255	178	1	255	255	255
179	4	255	255	255	179	1	255	255	255
180	1	255	255	255	180	1	255	255	255
181	1	255	255	255	181	290	255	255	255
182	5	255	255	255	182	1	255	255	255
183	1	255	255	255	183	1	255	255	255
184	147	255	255	255	184	70	255	255	255
185	2	255	255	255	185	3	255	255	255
186	15	255	255	255	186	1	255	255	255
187	2	255	255	255	187	2	255	255	255
188	1	255	255	255	188	1	255	255	255
189	161	255	255	255	189	1	255	255	255
190	1	255	255	255	190	1	255	255	255
191	3	255	255	255	191	119	255	255	255
192	264	255	255	255	192	1	255	255	255
193	2	255	255	255	193	6	255	255	255
194	1	255	255	255	194	85	255	255	255
195	4	255	255	255	195	1	255	255	255
196	2	255	255	255	196	1	255	255	255
197	2	255	255	255	197	1	255	255	255
198	1	255	255	255	198	141	255	255	255
199	1	255	255	255	199	2	255	255	255
200	6	255	255	255	200	1	255	255	255
201	6	255	255	255	201	42	255	255	255
202	1	255	255	255	202	1	255	255	255
203	6	255	255	255	203	2	255	255	255
204	2	255	255	255	204	72	255	255	255
205	3	255	255	255	205	48	255	255	255
206	2	255	255	255	206	2293	255	255	255
207	1	255	255	255	207	7	255	255	255
208	1	255	255	255	208	1	255	255	255
209	3	255	255	255	209	3	255	255	255
210	47	255	255	255	210	27	255	255	255
211	754	255	255	255	211	3	255	255	255
212	3	255	255	255	212	1	255	255	255
213	1	255	255	255	213	2	255	255	255
214	1	255	255	255	214	2	255	255	255
215	1	255	255	255	215	3	255	255	255
216	3	255	255	255	216	24	255	255	255
217	3	255	255	255	217	1	255	255	255
218	2	255	255	255	218	104	255	255	255
219	1	255	255	255	219	1	255	255	255
220	10	255	255	255	220	90	255	255	255
221	1	255	255	255	221	1	255	255	255
222	54	255	255	255	222	4	255	255	255
223	1	255	255	255	223	3	255	255	255
224	2	255	255	255	224	5	255	255	255
225	2	255	255	255	225	965	255	255	255
226	1	255	255	255	226	2	255	255	255
227	4	255	255	255	227	3	255	255	255
228	49	255	255	255	228	207	255	255	255
229	3	255	255	255	229	3	255	255	255
230	49	255	255	255	230	12	255	255	255
231	387	255	255	255	231	11	255	255	255
232	1	255	255	255	232	384	255	255	255
233	3	255	255	255	233	1	255	255	255
234	1	255	255	255	234	2	255	255	255
235	3	255	255	255	235	1	255	255	255
236	1	255	255	255	236	2	255	255	255
237	15	255	255	255	237	1	255	255	255
238	1	255	255	255	238	2	255	255	255
239	1	255	255	255	239	1	255	255	255
240	1	255	255	255	240	2	255	255	255
241	1	255	255	255	241	1	255	255	255
242	2	255	255	255	242	3	255	255	255
243	27	255	255	255	243	1	255	255	255
244	7	255	255	255	244	1	255	255	255
245	1	255	255	255	245	4	255	255	255
246	55	255	255	255	246	2	255	255	255
247	148	255	255	255	247	3	255	255	255
248	1	255	255	255	248	1	255	255	255
249	9	255	255	255	249	1	255	255	255
250	3	255	255	255	250	2	255	255	255
251	5	255	255	255	251	4	255	255	255
252	1	255	255	255	252	6	255	255	255
253	1	255	255	255	253	1	255	255	255
254	1	255	255	255	254	2	255	255	255
255	3	255	255	255	255	2	255	255	255
256	2	255	255	255	256	3	255	255	255
257	1	255	255	255	257	1	255	255	255
258	1	255	255	255	258	1	255	255	255
259	1	255	255	255	259	25	255	255	255
260	3	255	255	255	260	54	255	255	255
261	1	255	255	255	261	202	255	255	255
262	2	255	255	255	262	2	255	255	255

Original data for Figure 2D, Densitometric analysis of Nuclear/Perinuclear B-catenin in control and EoE patient esophageal biopsy tissue.

Values are representative of the mean of a sample size N=18. p<0.05**

263	4	255	255	255	263	1	255	255	255
264	1	255	255	255	264	1	255	255	255
265	3	255	255	255	265	1	255	255	255
266	12	255	255	255	266	1	255	255	255
267	1	255	255	255	267	62	255	255	255
268	103	255	255	255	268	1	255	255	255
269	1	255	255	255	269	1	255	255	255
270	4	255	255	255	270	35	255	255	255
271	2	255	255	255	271	2	255	255	255
272	3	255	255	255	272	10	255	255	255
273	1	255	255	255	273	1	255	255	255
274	24	255	255	255	274	2	255	255	255
275	1	255	255	255	275	14	255	255	255
276	1	255	255	255	276	19	255	255	255
277	2	255	255	255	277	1	255	255	255
278	3	255	255	255	278	2	255	255	255
279	1	255	255	255	279	2	255	255	255
280	1	255	255	255	280	1	255	255	255
281	1	255	255	255	281	1	255	255	255
282	2	255	255	255	282	1	255	255	255
283	6	255	255	255	283	26	255	255	255
284	1	255	255	255	284	14	255	255	255
285	227	255	255	255	285	1	255	255	255
286	1	255	255	255	286	1	255	255	255
287	38	255	255	255	287	25	255	255	255
288	1	255	255	255	288	95	255	255	255
289	1	255	255	255	289	2	255	255	255
290	1	255	255	255	290	1	255	255	255
291	3	255	255	255	291	50	255	255	255
292	1	255	255	255	292	3	255	255	255
293	2	255	255	255	293	1	255	255	255
294	1	255	255	255	294	1	255	255	255
295	7	255	255	255	295	11	255	255	255
296	2	255	255	255	296	1	255	255	255
297	2	255	255	255	297	2	255	255	255
298	2	255	255	255	298	10	255	255	255
299	1	255	255	255	299	2	255	255	255
300	1	255	255	255	300	1	255	255	255
301	1	255	255	255	301	131	255	255	255
302	3	255	255	255	302	1	255	255	255
303	1	255	255	255	303	1	255	255	255
304	13	255	255	255	304	121	255	255	255
305	4	255	255	255	305	1	255	255	255
306	15	255	255	255	306	7	255	255	255
307	1	255	255	255	307	5	255	255	255
308	3	255	255	255	308	1	255	255	255
309	1	255	255	255	309	1	255	255	255
310	168	255	255	255	310	29	255	255	255
311	1	255	255	255	311	119	255	255	255
312	3	255	255	255	312	13	255	255	255
313	9	255	255	255	313	1	255	255	255
314	2	255	255	255	314	1	255	255	255
315	1	255	255	255	315	1	255	255	255
316	2	255	255	255	316	163	255	255	255
317	2	255	255	255	317	1	255	255	255
318	1	255	255	255	318	3	255	255	255
319	458	255	255	255	319	1	255	255	255
320	3	255	255	255	320	109	255	255	255
321	1	255	255	255	321	49	255	255	255
322	181	255	255	255	322	193	255	255	255
323	38	255	255	255	323	1	255	255	255
324	2	255	255	255	324	2	255	255	255
325	21	255	255	255	325	2	255	255	255
326	503	255	255	255	326	3	255	255	255
327	2	255	255	255	327	37	255	255	255
328	2	255	255	255	328	2	255	255	255
329	426	255	255	255	329	1	255	255	255
330	1	255	255	255	330	2	255	255	255
331	4	255	255	255	331	2	255	255	255
332	2	255	255	255	332	26	255	255	255
333	1	255	255	255	333	15	255	255	255
334	1	255	255	255	334	74	255	255	255
335	2	255	255	255	335	1	255	255	255
336	3	255	255	255	336	1	255	255	255
337	14	255	255	255	337	103	255	255	255
338	4	255	255	255	338	1	255	255	255
339	4	255	255	255	339	4	255	255	255
340	1	255	255	255	340	1	255	255	255
341	4	255	255	255	341	2	255	255	255
342	1	255	255	255	342	2	255	255	255
343	17	255	255	255	343	10	255	255	255
344	3	255	255	255	344	1	255	255	255
345	3	255	255	255	345	4	255	255	255
346	196	255	255	255	346	1	255	255	255
347	1	255	255	255	347	1	255	255	255
348	4	255	255	255	348	9	255	255	255
349	1	255	255	255	349	4	255	255	255
350	4	255	255	255	350	4	255	255	255
351	1	255	255	255	351	2	255	255	255
352	2	255	255	255	352	1	255	255	255
353	1	255	255	255	353	4	255	255	255
354	7	255	255	255	354	82	255	255	255
355	1	255	255	255	355	197	255	255	255
356	1	255	255	255	356	6	255	255	255
357	1	255	255	255	357	1	255	255	255
358	3	255	255	255	358	1	255	255	255
359	1	255	255	255	359	1	255	255	255
360	1	255	255	255	360	81	255	255	255
361	1	255	255	255	361	5	255	255	255
362	1	255	255	255	362	2	255	255	255
363	1	255	255	255	363	2	255	255	255
364	1	255	255	255	364	20	255	255	255
365	2	255	255	255	365	4	255	255	255
366	1	255	255	255	366	1	255	255	255
367	1	255	255	255	367	1	255	255	255
368	10	255	255	255	368	2	255	255	255
369	1	255	255	255	369	1	255	255	255
370	2	255	255	255	370	2	255	255	255
371	1	255	255	255	371	1	255	255	255
372	1	255	255	255	372	1	255	255	255
373	1	255	255	255	373	2	255	255	255
374	1	255	255	255	374	213	255	255	255
375	1	255	255	255	375	1	255	255	255
376	1	255	255	255	376	1	255	255	255
377	1	255	255	255	377	26	255	255	255
378	648	255	255	255	378	9	255	255	255
379	3	255	255	255	379	78	255	255	255
380	7	255	255	255	380	1	255	255	255
381	1	255	255	255	381	1	255	255	255
382	2	255	255	255	382	2	255	255	255
383	1936	255	255	255	383	140	255	255	255
384	1	255	255	255	384	3	255	255	255
385	1	255	255	255	385	21	255	255	255
386	1	255	255	255	386	1	255	255	255
387	3	255	255	255	387	2	255	255	255
388	7	255	255	255	388	1	255	255	255
389	2	255	255	255	389	1	255	255	255
390	1	255	255	255	390	1	255	255	255
391	1	255	255	255	391	1	255	255	255
392	1	255	255	255	392	5	255	255	255
393	1	255	255	255	393	110	255	255	255
394	1	255	255	255	394	1	255	255	255
395	6	255	255	255	395	1	255	255	255
396	7	255	255	255	396	3	255	255	255
397	2	255	255	255	397	3	255	255	255
398	1	255	255	255	398	1	255	255	255
399	2	255	255	255	399	3	255	255	255
400	4	255	255	255	400	4	255	255	255

Original data for Figure 2D, Densitometric analysis of Nuclear/Perinuclear B-catenin in control and EoE patient esophageal biopsy tissue.

Values are representative of the mean of a sample size N=18. p<0.05**

401	3	255	255	255
402	6	255	255	255
403	3	255	255	255
404	48	255	255	255
405	1	255	255	255
406	4	255	255	255
407	2	255	255	255
408	2	255	255	255
409	1	255	255	255
410	51	255	255	255
411	3	255	255	255
412	1	255	255	255
413	1	255	255	255
414	2	255	255	255
415	2	255	255	255
416	3	255	255	255
417	1	255	255	255
418	1	255	255	255
419	2	255	255	255
420	1	255	255	255
421	4	255	255	255
422	14	255	255	255
423	1	255	255	255
424	83	255	255	255
425	1	255	255	255
426	3	255	255	255
427	14	255	255	255
428	2	255	255	255
429	11	255	255	255
430	2	255	255	255
431	2	255	255	255
432	8	255	255	255
433	1	255	255	255
434	18	255	255	255
435	2	255	255	255
436	1	255	255	255
437	2	255	255	255
438	2	255	255	255
439	28	255	255	255
440	4	255	255	255
441	1	255	255	255
442	1	255	255	255
443	2	255	255	255
444	20	255	255	255
445	5	255	255	255
446	3	255	255	255
447	1	255	255	255
448	3	255	255	255
449	1	255	255	255
450	773	255	255	255
451	1	255	255	255
452	1	255	255	255
453	1	255	255	255
454	1	255	255	255
455	1	255	255	255
456	4	255	255	255
457	38	255	255	255
458	2	255	255	255
459	782	255	255	255
460	6	255	255	255
461	1	255	255	255
462	7	255	255	255
463	14	255	255	255
464	1	255	255	255
465	43	255	255	255
466	1	255	255	255
467	2	255	255	255
468	1	255	255	255
469	1	255	255	255
470	7	255	255	255
471	130	255	255	255
472	1	255	255	255
473	1	255	255	255
474	5	255	255	255
475	1	255	255	255
476	67	255	255	255
477	5	255	255	255
478	5	255	255	255
479	1	255	255	255
480	2	255	255	255
481	7	255	255	255
482	1	255	255	255
483	1	255	255	255
484	2	255	255	255
485	2	255	255	255
486	8	255	255	255
487	7	255	255	255
488	4	255	255	255
489	2	255	255	255
490	6	255	255	255
491	7	255	255	255
492	1	255	255	255
493	1	255	255	255
494	27	255	255	255
495	2	255	255	255
496	1	255	255	255
497	1	255	255	255
498	531	255	255	255
499	1	255	255	255
500	173	255	255	255
501	14	255	255	255
502	3	255	255	255
503	2	255	255	255
504	1	255	255	255
505	6	255	255	255
506	1	255	255	255
507	1	255	255	255
508	2	255	255	255
509	9	255	255	255
510	4	255	255	255
511	3	255	255	255
512	9	255	255	255
513	15	255	255	255
514	2	255	255	255
515	6	255	255	255
516	14	255	255	255
517	1	255	255	255
518	1	255	255	255
519	1	255	255	255
520	1	255	255	255
521	1	255	255	255
522	998	255	255	255
523	152	255	255	255
524	1	255	255	255
525	1	255	255	255
526	1609	255	255	255
527	7	255	255	255
528	2	255	255	255
529	2	255	255	255
530	475	255	255	255
531	2	255	255	255
532	2	255	255	255
533	2	255	255	255
534	13	255	255	255
535	2	255	255	255
536	29	255	255	255
537	1	255	255	255
538	3	255	255	255

401	5	255	255	255
402	511	255	255	255
403	1	255	255	255
404	12	255	255	255
405	1	255	255	255
406	9	255	255	255
407	2	255	255	255
408	2	255	255	255
409	6	255	255	255
410	1	255	255	255
411	1	255	255	255
412	50	255	255	255
413	1	255	255	255
414	17	255	255	255
415	52	255	255	255
416	1	255	255	255
417	1	255	255	255
418	4	255	255	255
419	1	255	255	255
420	47	255	255	255
421	2	255	255	255
422	60	255	255	255
423	1	255	255	255
424	1	255	255	255
425	3	255	255	255
426	1	255	255	255
427	1	255	255	255
428	2	255	255	255
429	4	255	255	255
430	2	255	255	255
431	83	255	255	255
432	1	255	255	255
433	1	255	255	255
434	3	255	255	255
435	1	255	255	255
436	1	255	255	255
437	2	255	255	255
438	34	255	255	255
439	24	255	255	255
440	1	255	255	255
441	3	255	255	255
442	1	255	255	255
443	1	255	255	255
444	24	255	255	255
445	2	255	255	255
446	404	255	255	255
447	7	255	255	255
448	3	255	255	255
449	1	255	255	255
450	2	255	255	255
451	1	255	255	255
452	14	255	255	255
453	2	255	255	255
454	1	255	255	255
455	2	255	255	255
456	72	255	255	255
457	92	255	255	255
458	32	255	255	255
459	3	255	255	255
460	254	255	255	255
461	4	255	255	255
462	17	255	255	255
463	4	255	255	255
464	1	255	255	255
465	6	255	255	255
466	2	255	255	255
467	1	255	255	255
468	7	255	255	255
469	1	255	255	255
470	4	255	255	255
471	1	255	255	255
472	1	255	255	255
473	10	255	255	255
474	3	255	255	255
475	6	255	255	255
476	4	255	255	255
477	28	255	255	255
478	1	255	255	255
479	5	255	255	255
480	348	255	255	255
481	153	255	255	255
482	2	255	255	255
483	20	255	255	255
484	6	255	255	255
485	17	255	255	255
486	1	255	255	255
487	1	255	255	255
488	1	255	255	255
489	152	255	255	255
490	2	255	255	255
491	2	255	255	255
492	1	255	255	255
493	6	255	255	255
494	2	255	255	255
495	1	255	255	255
496	1	255	255	255
497	1	255	255	255
498	1	255	255	255
499	1	255	255	255
500	2	255	255	255
501	2	255	255	255

Original data for Figure 2D, Densitometric analysis of Nuclear/Perinuclear B-catenin in control and EoE patient esophageal biopsy tissue.
Values are representative of the mean of a sample size N=18. p<0.05**

539	55	255	255	255
540	1	255	255	255
541	2	255	255	255
542	1	255	255	255
543	76	255	255	255
544	1	255	255	255
545	3	255	255	255
546	2	255	255	255
547	2	255	255	255
548	2	255	255	255
549	1	255	255	255
550	2	255	255	255
551	7	255	255	255
552	6	255	255	255
553	15	255	255	255
554	1	255	255	255
555	1	255	255	255
556	1	255	255	255
557	6	255	255	255
558	1	255	255	255
559	2	255	255	255
560	175	255	255	255
561	2	255	255	255
562	2	255	255	255
563	50	255	255	255
564	21	255	255	255
565	16	255	255	255
566	1	255	255	255
567	2	255	255	255
568	2	255	255	255
569	3	255	255	255
570	19	255	255	255
571	1	255	255	255
572	5	255	255	255
573	642	255	255	255
574	52	255	255	255
575	2	255	255	255
576	5	255	255	255
577	2	255	255	255
578	1	255	255	255
579	2	255	255	255
580	726	255	255	255
581	31	255	255	255
582	2	255	255	255
583	20	255	255	255
584	4	255	255	255
585	2	255	255	255
586	40	255	255	255
587	2	255	255	255
588	1	255	255	255
589	2	255	255	255
590	1	255	255	255
591	1	255	255	255
592	2	255	255	255
593	3	255	255	255
594	1	255	255	255
595	1	255	255	255
596	1	255	255	255
597	2	255	255	255
598	1	255	255	255
599	1	255	255	255
600	447	255	255	255
601	10	255	255	255
602	131	255	255	255
603	1	255	255	255
604	1	255	255	255
605	2	255	255	255
606	1	255	255	255
607	52	255	255	255
608	3	255	255	255
609	1	255	255	255
610	5	255	255	255
611	3	255	255	255
612	42	255	255	255
613	1	255	255	255
614	21	255	255	255
615	2	255	255	255
616	64	255	255	255
617	1	255	255	255
618	1	255	255	255
619	98	255	255	255
620	1	255	255	255
621	9	255	255	255
622	1	255	255	255
623	1	255	255	255
624	7	255	255	255
625	1	255	255	255
626	5	255	255	255
627	2	255	255	255
628	1	255	255	255
629	13	255	255	255
630	1	255	255	255
631	425	255	255	255
632	3	255	255	255
633	21	255	255	255
634	1098	255	255	255
635	5	255	255	255
636	6	255	255	255
637	1	255	255	255
638	2	255	255	255
639	15	255	255	255
640	1	255	255	255
641	2	255	255	255
642	1	255	255	255
643	1	255	255	255
644	1	255	255	255
645	6	255	255	255
646	187	255	255	255
647	1	255	255	255
648	1	255	255	255
649	1	255	255	255
650	10	255	255	255
651	1	255	255	255
652	6	255	255	255
653	2	255	255	255
654	2	255	255	255
655	2	255	255	255
656	58	255	255	255
657	2	255	255	255
658	1	255	255	255
659	3	255	255	255
660	2	255	255	255
661	2	255	255	255
662	2	255	255	255
663	108	255	255	255
664	4	255	255	255
665	1	255	255	255
666	109	255	255	255
667	1	255	255	255
668	7	255	255	255
669	57	255	255	255
670	1	255	255	255
671	5	255	255	255
672	8	255	255	255
673	7	255	255	255
674	1	255	255	255
675	417	255	255	255
676	1	255	255	255

Original data for Figure 2D, Densitometric analysis of Nuclear/Perinuclear B-catenin in control and EoE patient esophageal biopsy tissue.
Values are representative of the mean of a sample size N=18. p<0.05**

677	1	255	255	255
678	1	255	255	255
679	25	255	255	255
680	1	255	255	255
681	1	255	255	255
682	2	255	255	255
683	1	255	255	255
684	2	255	255	255
685	1	255	255	255
686	2	255	255	255
687	8	255	255	255
688	2	255	255	255
689	1	255	255	255
690	1	255	255	255
691	158	255	255	255
692	2230	255	255	255
693	1	255	255	255
694	1	255	255	255
695	1	255	255	255
696	1	255	255	255
697	3	255	255	255
698	103	255	255	255
699	2	255	255	255
700	2	255	255	255
701	2	255	255	255
702	1	255	255	255
703	1	255	255	255
704	29	255	255	255
705	2	255	255	255
706	2	255	255	255
707	2	255	255	255
708	2	255	255	255
709	1	255	255	255
710	7	255	255	255
711	400	255	255	255
712	1	255	255	255
713	5	255	255	255
714	1	255	255	255
715	22	255	255	255
716	2	255	255	255
717	3	255	255	255
718	6	255	255	255
719	1	255	255	255
720	2	255	255	255
721	1	255	255	255
722	1	255	255	255
723	1	255	255	255
724	8	255	255	255
725	47	255	255	255
726	2	255	255	255
727	1	255	255	255
728	1	255	255	255
729	1	255	255	255
730	2	255	255	255
731	2	255	255	255
732	1	255	255	255
733	1	255	255	255
734	4	255	255	255
735	2	255	255	255
736	120	255	255	255
737	11	255	255	255
738	1	255	255	255
739	1	255	255	255
740	1	255	255	255
741	22	255	255	255
742	2	255	255	255
743	94	255	255	255
744	6	255	255	255
745	1	255	255	255
746	3	255	255	255
747	1	255	255	255
748	6	255	255	255
749	56	255	255	255
750	1	255	255	255
751	3	255	255	255
752	1	255	255	255
753	1	255	255	255
754	1	255	255	255
755	2	255	255	255
756	2	255	255	255
757	4	255	255	255
758	5	255	255	255
759	1	255	255	255
760	1	255	255	255
761	2	255	255	255
762	3	255	255	255
763	4	255	255	255
764	144	255	255	255
765	9	255	255	255
766	1	255	255	255
767	1	255	255	255
768	2	255	255	255
769	4	255	255	255
770	1	255	255	255
771	6	255	255	255
772	1	255	255	255
773	1	255	255	255
774	1	255	255	255
775	3	255	255	255
776	1	255	255	255
777	10	255	255	255
778	1	255	255	255
779	1	255	255	255
780	1	255	255	255
781	2	255	255	255
782	22	255	255	255
783	2	255	255	255
784	3	255	255	255
785	1	255	255	255
786	5	255	255	255
787	1	255	255	255
788	5	255	255	255
789	13	255	255	255
790	6	255	255	255
791	3	255	255	255
792	1	255	255	255
793	1	255	255	255
794	19	255	255	255
795	2	255	255	255
796	1	255	255	255
797	1	255	255	255
798	1	255	255	255
799	1	255	255	255
800	1	255	255	255
801	2	255	255	255
802	2	255	255	255
803	1	255	255	255
804	1	255	255	255
805	7	255	255	255
806	1	255	255	255
807	2	255	255	255
808	1	255	255	255
809	2	255	255	255
810	2	255	255	255
811	1	255	255	255
812	1	255	255	255
813	1	255	255	255
814	10	255	255	255

Original data for Figure 2D, Densitometric analysis of Nuclear/Perinuclear B-catenin in control and EoE patient esophageal biopsy tissue.
Values are representative of the mean of a sample size N=18. p<0.05**

815	10	255	255	255
816	30	255	255	255
817	2	255	255	255
818	1	255	255	255
819	3	255	255	255
820	1	255	255	255
821	1	255	255	255
822	3	255	255	255
823	5	255	255	255
824	19	255	255	255
825	3	255	255	255
826	4	255	255	255
827	3	255	255	255
828	5	255	255	255
829	112	255	255	255
830	1	255	255	255
831	1	255	255	255
832	2	255	255	255
833	1	255	255	255
834	517	255	255	255
835	1	255	255	255
836	1	255	255	255
837	1	255	255	255
838	1	255	255	255
839	1	255	255	255
840	2	255	255	255
841	1	255	255	255
842	1	255	255	255
843	2	255	255	255
844	1	255	255	255
845	22	255	255	255
846	3	255	255	255
847	2	255	255	255
848	1	255	255	255
849	3	255	255	255
850	1	255	255	255
851	7	255	255	255
852	4	255	255	255
853	1	255	255	255
854	1	255	255	255
855	1	255	255	255
856	1	255	255	255
857	1	255	255	255
858	1	255	255	255
859	1	255	255	255
860	1	255	255	255
861	1	255	255	255
862	2	255	255	255
863	4	255	255	255
864	1	255	255	255
865	1	255	255	255
866	3	255	255	255
867	2	255	255	255
868	2	255	255	255
869	1	255	255	255
870	2	255	255	255
871	1	255	255	255
872	1	255	255	255
873	6	255	255	255
874	4	255	255	255
875	2	255	255	255
876	1	255	255	255
877	1	255	255	255
878	18	255	255	255
879	1	255	255	255
880	5	255	255	255
881	1	255	255	255
882	1	255	255	255
883	1	255	255	255
884	1	255	255	255
885	2	255	255	255
886	4	255	255	255
887	4	255	255	255
888	3	255	255	255
889	1	255	255	255
890	1	255	255	255
891	2	255	255	255
892	1	255	255	255
893	6	255	255	255
894	1	255	255	255
895	1	255	255	255
896	2	255	255	255
897	4	255	255	255
898	1	255	255	255
899	1	255	255	255
900	2	255	255	255
901	3	255	255	255
902	1	255	255	255
903	8	255	255	255
904	6	255	255	255
905	2	255	255	255
906	1	255	255	255
907	1	255	255	255
908	63	255	255	255
909	2	255	255	255
910	1	255	255	255
911	2	255	255	255
912	1	255	255	255
913	2	255	255	255
914	2	255	255	255
915	2	255	255	255
916	1	255	255	255
917	1	255	255	255
918	1	255	255	255
919	1	255	255	255
920	1	255	255	255
921	1	255	255	255
922	24	255	255	255
923	2	255	255	255
924	13	255	255	255
925	4	255	255	255
926	1	255	255	255
927	2	255	255	255
928	4	255	255	255
929	2	255	255	255
930	32	255	255	255
931	1	255	255	255
932	1	255	255	255
933	3	255	255	255
934	25	255	255	255
935	3	255	255	255
936	2	255	255	255
937	44	255	255	255
938	2	255	255	255
939	2	255	255	255
940	1	255	255	255
941	2	255	255	255
942	1	255	255	255
943	1	255	255	255
944	1	255	255	255
945	42	255	255	255
946	2	255	255	255
947	1	255	255	255
948	2	255	255	255
949	1	255	255	255
950	48	255	255	255
951	5	255	255	255
952	4	255	255	255

Original data for Figure 2D, Densitometric analysis of Nuclear/Perinuclear B-catenin in control and EoE patient esophageal biopsy tissue.

Values are representative of the mean of a sample size N=18. p<0.05**

953	2	255	255	255
954	4	255	255	255
955	1	255	255	255
956	1	255	255	255
957	4	255	255	255
958	2	255	255	255
959	33	255	255	255
960	1	255	255	255
961	1	255	255	255
962	2	255	255	255
963	46	255	255	255
964	1	255	255	255
965	11	255	255	255
966	4	255	255	255
967	2	255	255	255
968	1	255	255	255
969	2	255	255	255
970	3	255	255	255
971	1	255	255	255
972	2	255	255	255
973	1	255	255	255
974	3	255	255	255
975	3	255	255	255
976	9	255	255	255
977	1	255	255	255
978	1	255	255	255
979	2	255	255	255
980	1	255	255	255
981	1	255	255	255
982	1	255	255	255
983	14	255	255	255
984	1	255	255	255
985	1	255	255	255
986	1	255	255	255
987	1	255	255	255
988	3	255	255	255
989	8	255	255	255
990	1	255	255	255
991	1	255	255	255
992	23	255	255	255
993	1	255	255	255
994	1	255	255	255
995	2	255	255	255
996	1	255	255	255
997	1	255	255	255
998	1	255	255	255
999	34	255	255	255
1000	1	255	255	255
1001	57	255	255	255
1002	1	255	255	255
1003	1	255	255	255
1004	1	255	255	255
1005	1	255	255	255
1006	3	255	255	255
1007	26	255	255	255
1008	1	255	255	255
1009	176	255	255	255
1010	1	255	255	255
1011	1	255	255	255
1012	1	255	255	255
1013	2	255	255	255
1014	1	255	255	255
1015	45	255	255	255
1016	2	255	255	255
1017	2	255	255	255
1018	1	255	255	255
1019	4	255	255	255
1020	5	255	255	255
1021	613	255	255	255
1022	1	255	255	255
1023	2	255	255	255
1024	56	255	255	255
1025	2	255	255	255
1026	2	255	255	255
1027	5	255	255	255
1028	1	255	255	255
1029	1	255	255	255
1030	1	255	255	255
1031	34	255	255	255
1032	1	255	255	255
1033	1	255	255	255
1034	1	255	255	255
1035	1	255	255	255
1036	1	255	255	255
1037	1	255	255	255
1038	1	255	255	255
1039	1	255	255	255
1040	1	255	255	255
1041	1	255	255	255
1042	1	255	255	255
1043	3	255	255	255
1044	3	255	255	255
1045	1	255	255	255
1046	2	255	255	255
1047	1	255	255	255
1048	1	255	255	255
1049	33	255	255	255
1050	16	255	255	255
1051	1	255	255	255
1052	2	255	255	255
1053	40	255	255	255
1054	17	255	255	255
1055	11	255	255	255
1056	2	255	255	255
1057	1	255	255	255
1058	3	255	255	255
1059	1	255	255	255
1060	4	255	255	255
1061	1	255	255	255
1062	1	255	255	255

Original data for Figure 2D, Densitometric analysis of Nuclear/Perinuclear B-catenin in control and EoE patient esophageal biopsy tissue.
Values are representative of the mean of a sample size N=18. p<0.05**

CONTROL
Esophageal biobsy tissue

Count	Total Area	Average Size		
nuclear Area				
1	0.015	255	255	255
2	0.000109	255	255	255
3	0.000109	255	255	255
4	0.000326	255	255	255
5	0.032	255	255	255
6	0.004	255	255	255
7	0.000326	255	255	255
8	0.000109	255	255	255
9	0.011	255	255	255
10	0.000651	255	255	255
11	0.036	255	255	255
12	0.009	255	255	255
13	0.000109	255	255	255
14	0.000109	255	255	255
15	0.000868	255	255	255
16	0.000109	255	255	255
17	0.000651	255	255	255
18	0.000217	255	255	255
19	0.013	255	255	255
20	0.003	255	255	255
21	0.000109	255	255	255
22	0.000109	255	255	255
23	0.001	255	255	255
24	0.000109	255	255	255
25	0.000326	255	255	255
26	0.000217	255	255	255
27	0.002	255	255	255
28	0.000109	255	255	255
29	0.000109	255	255	255
30	0.000217	255	255	255
31	0.000109	255	255	255
32	0.000326	255	255	255
33	0.000109	255	255	255
34	0.000109	255	255	255
35	0.000217	255	255	255
36	0.000217	255	255	255
37	0.000217	255	255	255
38	0.000109	255	255	255
39	0.000217	255	255	255
40	0.000543	255	255	255
41	0.000109	255	255	255
42	0.029	255	255	255
43	0.000109	255	255	255
44	0.000109	255	255	255
45	0.006	255	255	255
46	0.000434	255	255	255
47	0.000543	255	255	255
48	0.00076	255	255	255
49	0.003	255	255	255
50	0.000217	255	255	255
51	0.000217	255	255	255
52	0.000109	255	255	255
53	0.000326	255	255	255
54	0.002	255	255	255
55	0.000109	255	255	255
56	0.000217	255	255	255
57	0.000109	255	255	255
58	0.000326	255	255	255
59	0.000977	255	255	255
60	0.002	255	255	255
61	0.000651	255	255	255
62	0.006	255	255	255
63	0.000217	255	255	255
64	0.000543	255	255	255
65	0.000109	255	255	255
66	0.000109	255	255	255
67	0.000109	255	255	255
68	0.018	255	255	255
69	0.007	255	255	255
70	0.000651	255	255	255
71	0.000651	255	255	255
72	0.067	255	255	255
73	0.000217	255	255	255
74	0.000109	255	255	255
75	0.000326	255	255	255
76	0.002	255	255	255
77	0.003	255	255	255
78	0.000109	255	255	255
79	0.000326	255	255	255
80	0.002	255	255	255
81	0.006	255	255	255
82	0.000109	255	255	255
83	0.000109	255	255	255
84	0.013	255	255	255
85	0.001	255	255	255
86	0.000109	255	255	255
87	0.000434	255	255	255
88	0.000109	255	255	255
89	0.000434	255	255	255
90	0.000217	255	255	255
91	0.000109	255	255	255
92	0.000109	255	255	255
93	0.000109	255	255	255
94	0.000109	255	255	255
95	0.000326	255	255	255
96	0.000109	255	255	255
97	0.000109	255	255	255
98	0.000109	255	255	255
99	0.000217	255	255	255
100	0.000651	255	255	255
101	0.000217	255	255	255
102	0.002	255	255	255
103	0.012	255	255	255
104	0.000109	255	255	255
105	0.000109	255	255	255
106	0.000109	255	255	255
107	0.000217	255	255	255
108	0.000543	255	255	255
109	0.000109	255	255	255
110	0.001	255	255	255
111	0.000217	255	255	255
112	0.000543	255	255	255
113	0.000543	255	255	255
114	0.000109	255	255	255
115	0.000109	255	255	255
116	0.000109	255	255	255
117	0.001	255	255	255
118	0.000109	255	255	255
119	0.000109	255	255	255
120	0.000434	255	255	255
121	0.000109	255	255	255
122	0.000217	255	255	255
123	0.000217	255	255	255
124	0.000109	255	255	255

CONTROL
Esophageal biobsy tissue

Count	Total Area	Average Size		
Nuclear Area				
1	0.000109	255	255	255
2	0.027	255	255	255
3	0.007	255	255	255
4	0.000326	255	255	255
5	0.000109	255	255	255
6	0.000217	255	255	255
7	0.022	255	255	255
8	0.000326	255	255	255
9	0.000109	255	255	255
10	0.000217	255	255	255
11	0.000217	255	255	255
12	0.000109	255	255	255
13	0.000217	255	255	255
14	0.000109	255	255	255
15	0.000543	255	255	255
16	1.455	255	255	255
17	0.000217	255	255	255
18	0.000109	255	255	255
19	0.000543	255	255	255
20	0.000217	255	255	255
21	0.000109	255	255	255
22	0.00076	255	255	255
23	0.03	255	255	255
24	0.000109	255	255	255
25	0.002	255	255	255
26	0.000109	255	255	255
27	0.000651	255	255	255
28	0.000543	255	255	255
29	0.000109	255	255	255
30	0.00076	255	255	255
31	0.000109	255	255	255
32	0.000109	255	255	255
33	0.000109	255	255	255
34	0.006	255	255	255
35	0.000217	255	255	255
36	0.000109	255	255	255
37	0.005	255	255	255
38	0.003	255	255	255
39	0.01	255	255	255
40	0.000109	255	255	255
41	0.003	255	255	255
42	0.000217	255	255	255
43	0.000651	255	255	255
44	0.000217	255	255	255
45	0.002	255	255	255
46	0.000543	255	255	255
47	0.000109	255	255	255
48	0.001	255	255	255
49	0.000651	255	255	255
50	0.000977	255	255	255
51	0.000109	255	255	255
52	0.000109	255	255	255
53	0.000109	255	255	255
54	0.003	255	255	255
55	0.000109	255	255	255
56	0.000217	255	255	255
57	0.000109	255	255	255
58	0.000109	255	255	255
59	0.000109	255	255	255
60	0.000109	255	255	255
61	0.000326	255	255	255
62	0.000543	255	255	255
63	0.000109	255	255	255
64	0.004	255	255	255
65	0.000109	255	255	255
66	0.018	255	255	255
67	0.000109	255	255	255
68	0.000217	255	255	255
69	0.000434	255	255	255
70	0.000434	255	255	255
71	0.006	255	255	255
72	0.000109	255	255	255
73	0.000326	255	255	255
74	0.000109	255	255	255
75	0.000217	255	255	255
76	0.000109	255	255	255
77	0.003	255	255	255
78	0.000217	255	255	255
79	0.000109	255	255	255
80	0.000109	255	255	255
81	0.000109	255	255	255
82	0.000109	255	255	255
83	0.000109	255	255	255
84	0.001	255	255	255
85	0.004	255	255	255
86	0.000109	255	255	255
87	0.000109	255	255	255
88	0.000217	255	255	255
89	0.000109	255	255	255
90	0.005	255	255	255
91	0.000109	255	255	255
92	0.000109	255	255	255
93	0.000217	255	255	255
94	0.001	255	255	255
95	0.000109	255	255	255
96	0.000109	255	255	255
97	0.000326	255	255	255
98	0.006	255	255	255
99	0.000109	255	255	255
100	0.000217	255	255	255
101	0.002	255	255	255
102	0.000326	255	255	255
103	0.000109	255	255	255
104	0.01	255	255	255
105	0.000434	255	255	255
106	0.000217	255	255	255
107	0.000109	255	255	255
108	0.006	255	255	255
109	0.000217	255	255	255
110	0.000326	255	255	255
111	0.000326	255	255	255
112	0.000977	255	255	255
113	0.000217	255	255	255
114	0.000109	255	255	255
115	0.000977	255	255	255
116	0.000109	255	255	255
117	0.002	255	255	255
118	0.001	255	255	255
119	0.000109	255	255	255
120	0.002	255	255	255
121	0.000651	255	255	255
122	0.003	255	255	255
123	0.000651	255	255	255
124	0.000109	255	255	255

Original data for Figure 2D, Densitometric analysis of Nuclear/Perinuclear B-catenin in control and EoE patient esophageal biopsy tissue.

Values are representative of the mean of a sample size N=18. p<0.05**

125	0.000109	255	255	255	125	0.000217	255	255	255
126	0.000109	255	255	255	126	0.000217	255	255	255
127	0.000109	255	255	255	127	0.000217	255	255	255
128	0.000109	255	255	255	128	0.000109	255	255	255
129	0.000217	255	255	255	129	0.492	255	255	255
130	0.000326	255	255	255	130	0.002	255	255	255
131	0.008	255	255	255	131	0.000109	255	255	255
132	0.000651	255	255	255	132	0.000109	255	255	255
133	0.000109	255	255	255	133	0.001	255	255	255
134	0.000217	255	255	255	134	0.000217	255	255	255
135	0.000109	255	255	255	135	0.000109	255	255	255
136	0.007	255	255	255	136	0.004	255	255	255
137	0.000109	255	255	255	137	0.000109	255	255	255
138	0.000109	255	255	255	138	0.006	255	255	255
139	0.000217	255	255	255	139	0.000109	255	255	255
140	0.000217	255	255	255	140	0.001	255	255	255
141	0.000326	255	255	255	141	0.000109	255	255	255
142	0.000109	255	255	255	142	0.000109	255	255	255
143	0.001	255	255	255	143	0.000868	255	255	255
144	0.029	255	255	255	144	0.002	255	255	255
145	0.000109	255	255	255	145	0.000109	255	255	255
146	0.000217	255	255	255	146	0.000109	255	255	255
147	0.000109	255	255	255	147	0.000868	255	255	255
148	0.007	255	255	255	148	0.000109	255	255	255
149	0.000217	255	255	255	149	0.000109	255	255	255
150	0.005	255	255	255	150	0.000217	255	255	255
151	0.003	255	255	255	151	0.000109	255	255	255
152	0.000109	255	255	255	152	0.000868	255	255	255
153	0.000109	255	255	255	153	0.024	255	255	255
154	0.001	255	255	255	154	0.013	255	255	255
155	0.000434	255	255	255	155	0.000868	255	255	255
156	0.011	255	255	255	156	0.000868	255	255	255
157	0.000109	255	255	255	157	0.013	255	255	255
158	0.029	255	255	255	158	0.000109	255	255	255
159	0.000109	255	255	255	159	0.31	255	255	255
160	0.000109	255	255	255	160	0.000109	255	255	255
161	0.009	255	255	255	161	0.005	255	255	255
162	0.000109	255	255	255	162	0.000543	255	255	255
163	0.000109	255	255	255	163	0.000977	255	255	255
164	0.000109	255	255	255	164	0.000109	255	255	255
165	0.015	255	255	255	165	0.015	255	255	255
166	0.000109	255	255	255	166	0.000217	255	255	255
167	0.000109	255	255	255	167	0.023	255	255	255
168	0.003	255	255	255	168	0.063	255	255	255
169	0.000217	255	255	255	169	0.000217	255	255	255
170	0.000109	255	255	255	170	0.000109	255	255	255
171	0.000109	255	255	255	171	0.003	255	255	255
172	0.000109	255	255	255	172	0.000109	255	255	255
173	0.000217	255	255	255	173	0.001	255	255	255
174	0.031	255	255	255	174	0.03	255	255	255
175	0.000109	255	255	255	175	0.000326	255	255	255
176	0.000109	255	255	255	176	0.000109	255	255	255
177	0.000326	255	255	255	177	0.000217	255	255	255
178	0.000434	255	255	255	178	0.000326	255	255	255
179	0.006	255	255	255	179	0.002	255	255	255
180	0.000109	255	255	255	180	0.000434	255	255	255
181	0.000109	255	255	255	181	0.000109	255	255	255
182	0.005	255	255	255	182	0.000109	255	255	255
183	0.000109	255	255	255	183	0.000217	255	255	255
184	0.000217	255	255	255	184	0.028	255	255	255
185	0.000543	255	255	255	185	0.000109	255	255	255
186	0.000109	255	255	255	186	0.000109	255	255	255
187	0.000109	255	255	255	187	0.003	255	255	255
188	0.000217	255	255	255	188	0.000543	255	255	255
189	0.000217	255	255	255	189	0.007	255	255	255
190	0.000109	255	255	255	190	0.000109	255	255	255
191	0.008	255	255	255	191	0.000217	255	255	255
192	0.000217	255	255	255	192	0.006	255	255	255
193	0.000217	255	255	255	193	0.000109	255	255	255
194	0.000326	255	255	255	194	0.000109	255	255	255
195	0.001	255	255	255	195	0.000109	255	255	255
196	0.000434	255	255	255	196	0.000109	255	255	255
197	0.001	255	255	255	197	0.014	255	255	255
198	0.011	255	255	255	198	0.003	255	255	255
199	0.000109	255	255	255	199	0.000326	255	255	255
200	0.000109	255	255	255	200	0.000109	255	255	255
201	0.000109	255	255	255	201	0.000109	255	255	255
202	0.008	255	255	255	202	0.000543	255	255	255
203	0.000326	255	255	255	203	0.000109	255	255	255
204	0.001	255	255	255	204	0.000217	255	255	255
205	0.006	255	255	255	205	0.000109	255	255	255
206	0.000217	255	255	255	206	0.000326	255	255	255
207	0.000109	255	255	255	207	0.000217	255	255	255
208	0.000109	255	255	255	208	0.000543	255	255	255
209	0.00076	255	255	255	209	0.000109	255	255	255
210	0.000109	255	255	255	210	0.000109	255	255	255
211	0.017	255	255	255	211	0.000543	255	255	255
212	0.000109	255	255	255	212	0.001	255	255	255
213	0.000109	255	255	255	213	0.000109	255	255	255
214	0.000109	255	255	255	214	0.002	255	255	255
215	0.000109	255	255	255	215	0.002	255	255	255
216	0.035	255	255	255	216	0.000109	255	255	255
217	0.000109	255	255	255	217	0.003	255	255	255
218	0.000217	255	255	255	218	0.000434	255	255	255
219	0.003	255	255	255	219	0.000217	255	255	255
220	0.000217	255	255	255	220	0.000109	255	255	255
221	0.000543	255	255	255	221	0.000977	255	255	255
222	0.005	255	255	255	222	0.000217	255	255	255
223	0.007	255	255	255	223	0.000109	255	255	255
224	0.000326	255	255	255	224	0.000217	255	255	255
225	0.000109	255	255	255	225	0.000326	255	255	255
226	0.000109	255	255	255	226	0.000109	255	255	255
227	0.002	255	255	255	227	0.000109	255	255	255
228	0.00076	255	255	255	228	0.001	255	255	255
229	0.000109	255	255	255	229	0.003	255	255	255
230	0.000109	255	255	255	230	0.002	255	255	255
231	0.000109	255	255	255	231	0.000217	255	255	255
232	0.000109	255	255	255	232	0.000109	255	255	255
233	0.000109	255	255	255	233	0.000109	255	255	255
234	0.021	255	255	255	234	0.000977	255	255	255
235	0.001	255	255	255	235	0.000109	255	255	255
236	0.000109	255	255	255	236	0.000326	255	255	255
237	0.000109	255	255	255	237	0.000217	255	255	255
238	0.001	255	255	255	238	0.000651	255	255	255
239	0.000434	255	255	255	239	0.004	255	255	255
240	0.006	255	255	255	240	0.000109	255	255	255
241	0.000109	255	255	255	241	0.002	255	255	255
242	0.026	255	255	255	242	0.000651	255	255	255
243	0.000109	255	255	255	243	0.000109	255	255	255
244	0.246	255	255	255	244	0.000434	255	255	255
245	0.000109	255	255	255	245	0.002	255	255	255
246	0.005	255	255	255	246	0.000543	255	255	255
247	0.000109	255	255	255	247	0.000109	255	255	255
248	0.000109	255	255	255	248	0.002	255	255	255
249	0.007	255	255	255	249	0.000326	255	255	255
250	0.009	255	255	255	250	0.002	255	255	255
251	0.015	255	255	255	251	0.000217	255	255	255
252	0.000109	255	255	255	252	0.003	255	255	255
253	0.000217	255	255	255	253	0.000326	255	255	255
254	0.000109	255	255	255	254	0.002	255	255	255
255	0.000109	255	255	255	255	0.000109	255	255	255
256	0.012	255	255	255	256	0.000543	255	255	255
257	0.000109	255	255	255	257	0.028	255	255	255
258	0.000109	255	255	255	258	0.000109	255	255	255
259	0.000109	255	255	255	259	0.000109	255	255	255
260	0.121	255	255	255	260	0.000326	255	255	255
261	0.000109	255	255	255	261	0.000434	255	255	255
262	0.000109	255	255	255	262	0.009	255	255	255

Original data for Figure 2D, Densitometric analysis of Nuclear/Perinuclear
B-catenin in control and EoE patient esophageal biopsy tissue.
Values are representative of the mean of a sample size N=18. p<0.05**

263	0.00076	255	255	255
264	0.000109	255	255	255
265	0.000109	255	255	255
266	0.000109	255	255	255
267	0.028	255	255	255
268	0.000109	255	255	255
269	0.000109	255	255	255
270	0.000217	255	255	255
271	0.00076	255	255	255
272	0.003	255	255	255
273	0.000109	255	255	255
274	0.002	255	255	255
275	0.000109	255	255	255
276	0.044	255	255	255
277	0.000217	255	255	255
278	0.000326	255	255	255
279	0.000109	255	255	255
280	0.008	255	255	255
281	0.000109	255	255	255
282	0.000651	255	255	255
283	0.002	255	255	255
284	0.000109	255	255	255
285	0.000109	255	255	255
286	0.000217	255	255	255
287	0.000109	255	255	255
288	0.004	255	255	255
289	0.000109	255	255	255
290	0.000109	255	255	255
291	0.000109	255	255	255
292	0.000109	255	255	255
293	0.005	255	255	255
294	0.000217	255	255	255
295	0.000543	255	255	255
296	0.000109	255	255	255
297	0.005	255	255	255
298	0.003	255	255	255
299	0.032	255	255	255
300	0.000109	255	255	255
301	0.000217	255	255	255
302	0.001	255	255	255
303	0.000977	255	255	255
304	0.000109	255	255	255
305	0.000543	255	255	255
306	0.000109	255	255	255
307	0.000543	255	255	255
308	0.000217	255	255	255
309	0.002	255	255	255
310	0.012	255	255	255
311	0.00076	255	255	255
312	0.000109	255	255	255
313	0.000109	255	255	255
314	0.000109	255	255	255
315	0.000434	255	255	255
316	0.007	255	255	255
317	0.000109	255	255	255
318	0.000109	255	255	255
319	0.001	255	255	255
320	0.007	255	255	255
321	0.012	255	255	255
322	0.000977	255	255	255
323	0.000109	255	255	255
324	0.000109	255	255	255
325	0.000109	255	255	255
326	0.000109	255	255	255
327	0.000434	255	255	255
328	0.000109	255	255	255
329	0.000109	255	255	255
330	0.007	255	255	255
331	0.004	255	255	255
332	0.000109	255	255	255
333	0.000326	255	255	255
334	0.000109	255	255	255
335	0.005	255	255	255
336	0.021	255	255	255
337	0.000217	255	255	255
338	0.000109	255	255	255
339	0.000109	255	255	255
340	0.000109	255	255	255
341	0.006	255	255	255
342	0.000109	255	255	255
343	0.000217	255	255	255
344	0.017	255	255	255
345	0.000217	255	255	255
346	0.003	255	255	255
347	0.000109	255	255	255
348	0.000109	255	255	255
349	0.000217	255	255	255
350	0.014	255	255	255
351	0.000109	255	255	255
352	0.000217	255	255	255
353	0.000109	255	255	255
354	0.000217	255	255	255
355	0.000217	255	255	255
356	0.000109	255	255	255
357	0.002	255	255	255
358	0.015	255	255	255
359	0.000434	255	255	255
360	0.000217	255	255	255
361	0.000109	255	255	255
362	0.000217	255	255	255
363	0.000109	255	255	255
364	0.002	255	255	255
365	0.019	255	255	255
366	0.000109	255	255	255
367	0.000109	255	255	255
368	0.00076	255	255	255
369	0.000217	255	255	255
370	0.000109	255	255	255
371	0.000217	255	255	255
372	0.000326	255	255	255
373	0.002	255	255	255
374	0.000326	255	255	255
375	0.000109	255	255	255
376	0.015	255	255	255
377	0.000217	255	255	255
378	0.000109	255	255	255
379	0.000109	255	255	255
380	0.000217	255	255	255
381	0.000109	255	255	255
382	0.005	255	255	255
383	0.000109	255	255	255
384	0.000109	255	255	255
385	0.000109	255	255	255
386	0.000109	255	255	255
387	0.000109	255	255	255
388	0.001	255	255	255
389	0.000217	255	255	255
390	0.000109	255	255	255
391	0.000217	255	255	255
392	0.000977	255	255	255
393	0.000217	255	255	255
394	0.000543	255	255	255
395	0.004	255	255	255
396	0.000543	255	255	255
397	0.000217	255	255	255
398	0.000217	255	255	255
399	0.000109	255	255	255
400	0.000109	255	255	255

263	0.021	255	255	255
264	0.041	255	255	255
265	0.007	255	255	255
266	0.000217	255	255	255
267	0.007	255	255	255
268	0.000326	255	255	255
269	0.002	255	255	255
270	0.000109	255	255	255
271	0.008	255	255	255
272	0.001	255	255	255
273	0.001	255	255	255
274	0.000109	255	255	255
275	0.000109	255	255	255
276	0.000109	255	255	255
277	0.000217	255	255	255
278	0.002	255	255	255
279	0.000217	255	255	255
280	0.03	255	255	255
281	0.000651	255	255	255
282	0.000109	255	255	255
283	0.000217	255	255	255
284	0.006	255	255	255
285	0.000217	255	255	255
286	0.000109	255	255	255

Original data for Figure 2D, Densitometric analysis of Nuclear/Perinuclear B-catenin in control and EoE patient esophageal biopsy tissue.
Values are representative of the mean of a sample size N=18. p<0.05**

401	0.000109	255	255	255
402	0.005	255	255	255
403	0.000217	255	255	255
404	0.002	255	255	255
405	0.000109	255	255	255
406	0.000109	255	255	255
407	0.015	255	255	255
408	0.000868	255	255	255
409	0.000109	255	255	255
410	0.000543	255	255	255
411	0.00076	255	255	255
412	0.000217	255	255	255
413	0.000326	255	255	255
414	0.001	255	255	255
415	0.000326	255	255	255
416	0.000109	255	255	255
417	0.000434	255	255	255
418	0.000543	255	255	255
419	0.000868	255	255	255
420	0.01	255	255	255
421	0.000109	255	255	255
422	0.000217	255	255	255
423	0.000326	255	255	255
424	0.002	255	255	255
425	0.01	255	255	255
426	0.000434	255	255	255
427	0.000109	255	255	255
428	0.000109	255	255	255
429	0.000109	255	255	255
430	0.002	255	255	255
431	0.000217	255	255	255
432	0.002	255	255	255
433	0.000109	255	255	255
434	0.01	255	255	255
435	0.000217	255	255	255
436	0.003	255	255	255
437	0.001	255	255	255
438	0.000109	255	255	255
439	0.000651	255	255	255
440	0.00076	255	255	255
441	0.006	255	255	255
442	0.000109	255	255	255
443	0.000109	255	255	255
444	0.000217	255	255	255
445	0.02	255	255	255
446	0.000109	255	255	255
447	0.001	255	255	255
448	0.000109	255	255	255
449	0.004	255	255	255
450	0.000217	255	255	255
451	0.000977	255	255	255
452	0.000109	255	255	255
453	0.000109	255	255	255
454	0.01	255	255	255
455	0.000217	255	255	255
456	0.000217	255	255	255
457	0.000109	255	255	255
458	0.001	255	255	255
459	0.000543	255	255	255
460	0.004	255	255	255
461	0.000109	255	255	255
462	0.002	255	255	255
463	0.000109	255	255	255
464	0.00076	255	255	255
465	0.002	255	255	255
466	0.000109	255	255	255
467	0.000109	255	255	255
468	0.001	255	255	255
469	0.000651	255	255	255
470	0.000217	255	255	255
471	0.000109	255	255	255
472	0.000434	255	255	255
473	0.000109	255	255	255
474	0.000109	255	255	255
475	0.000326	255	255	255
476	0.000651	255	255	255
477	0.000109	255	255	255
478	0.000109	255	255	255
479	0.000217	255	255	255
480	0.000109	255	255	255
481	0.000326	255	255	255
482	0.000326	255	255	255
483	0.000109	255	255	255
484	0.000109	255	255	255
485	0.000326	255	255	255
486	0.051	255	255	255
487	0.000326	255	255	255
488	0.000109	255	255	255
489	0.000651	255	255	255
490	0.000109	255	255	255
491	0.000868	255	255	255
492	0.000326	255	255	255
493	0.000543	255	255	255
494	0.000217	255	255	255
495	0.008	255	255	255
496	0.000109	255	255	255
497	0.007	255	255	255
498	0.000109	255	255	255
499	0.000326	255	255	255
500	0.000109	255	255	255
501	0.000109	255	255	255
502	0.003	255	255	255
503	0.000326	255	255	255
504	0.000109	255	255	255
505	0.000543	255	255	255
506	0.000434	255	255	255
507	0.000434	255	255	255
508	0.000543	255	255	255
509	0.000326	255	255	255
510	0.000543	255	255	255
511	0.000109	255	255	255
512	0.002	255	255	255
513	0.000109	255	255	255
514	0.000217	255	255	255
515	0.000109	255	255	255
516	0.000217	255	255	255
517	0.000109	255	255	255
518	0.002	255	255	255
519	0.001	255	255	255
520	0.001	255	255	255
521	0.000109	255	255	255
522	0.000651	255	255	255
523	0.000217	255	255	255
524	0.000543	255	255	255
525	0.00076	255	255	255
526	0.000217	255	255	255
527	0.000326	255	255	255
528	0.002	255	255	255
529	0.000109	255	255	255
530	0.003	255	255	255
531	0.005	255	255	255
532	0.000109	255	255	255
533	0.000109	255	255	255
534	0.003	255	255	255
535	0.000109	255	255	255
536	0.029	255	255	255
537	0.000109	255	255	255
538	0.000109	255	255	255

Original data for Figure 2D, Densitometric analysis of Nuclear/Perinuclear B-catenin in control and EoE patient esophageal biopsy tissue.

Values are representative of the mean of a sample size N=18. p<0.05**

539	0.000217	255	255	255
540	0.003	255	255	255
541	0.000868	255	255	255
542	0.000109	255	255	255
543	0.00076	255	255	255
544	0.000109	255	255	255
545	0.000109	255	255	255
546	0.000326	255	255	255
547	0.000109	255	255	255
548	0.005	255	255	255
549	0.000217	255	255	255
550	0.000109	255	255	255
551	0.000109	255	255	255
552	0.011	255	255	255
553	0.009	255	255	255
554	0.000109	255	255	255
555	0.005	255	255	255
556	0.000109	255	255	255
557	0.003	255	255	255
558	0.000109	255	255	255
559	0.000109	255	255	255
560	0.000109	255	255	255
561	0.000109	255	255	255
562	0.000434	255	255	255
563	0.000109	255	255	255
564	0.000217	255	255	255
565	0.001	255	255	255
566	0.000543	255	255	255
567	0.000109	255	255	255
568	0.000326	255	255	255
569	0.000651	255	255	255
570	0.000109	255	255	255
571	0.015	255	255	255
572	0.000326	255	255	255
573	0.000217	255	255	255
574	0.000326	255	255	255
575	0.000217	255	255	255
576	0.000109	255	255	255
577	0.000109	255	255	255
578	0.000109	255	255	255
579	0.000109	255	255	255
580	0.000651	255	255	255
581	0.013	255	255	255
582	0.000109	255	255	255
583	0.000109	255	255	255
584	0.000109	255	255	255
585	0.000217	255	255	255
586	0.000109	255	255	255
587	0.036	255	255	255
588	0.000109	255	255	255
589	0.000109	255	255	255
590	0.000217	255	255	255
591	0.000434	255	255	255
592	0.002	255	255	255
593	0.000543	255	255	255
594	0.000543	255	255	255
595	0.000543	255	255	255
596	0.000109	255	255	255
597	0.001	255	255	255
598	0.000109	255	255	255
599	0.014	255	255	255
600	0.000109	255	255	255
601	0.000109	255	255	255
602	0.000109	255	255	255
603	0.000109	255	255	255
604	0.005	255	255	255
605	0.000543	255	255	255
606	0.000109	255	255	255
607	0.000109	255	255	255
608	0.000868	255	255	255
609	0.000217	255	255	255
610	0.000109	255	255	255
611	0.000109	255	255	255
612	0.000326	255	255	255
613	0.005	255	255	255
614	0.000326	255	255	255
615	0.000326	255	255	255
616	0.000109	255	255	255
617	0.000109	255	255	255
618	0.000109	255	255	255
619	0.000109	255	255	255

Original data for Figure 2D, Densitometric analysis of Nuclear/Perinuclear B-catenin in control and EoE patient esophageal biopsy tissue.
Values are representative of the mean of a sample size N=18. p<0.05**

EoE
EoE patient esophageal biopsy tissue

Count				
Nuclear Ar	Total Area	Average Size		
1	199	255	255	255
2	286	255	255	255
3	1	255	255	255
4	143	255	255	255
5	1	255	255	255
6	1	255	255	255
7	14	255	255	255
8	2	255	255	255
9	9	255	255	255
10	1594	255	255	255
11	1	255	255	255
12	1	255	255	255
13	224	255	255	255
14	420	255	255	255
15	4	255	255	255
16	1	255	255	255
17	4	255	255	255
18	127	255	255	255
19	58	255	255	255
20	1	255	255	255
21	9	255	255	255
22	2	255	255	255
23	376	255	255	255
24	2	255	255	255
25	3	255	255	255
26	1	255	255	255
27	1	255	255	255
28	1	255	255	255
29	956	255	255	255
30	2	255	255	255
31	1	255	255	255
32	2	255	255	255
33	1	255	255	255
34	1	255	255	255
35	1	255	255	255
36	140	255	255	255
37	2	255	255	255
38	2	255	255	255
39	2	255	255	255
40	1	255	255	255
41	7118	255	255	255
42	2	255	255	255
43	16	255	255	255
44	118	255	255	255
45	1	255	255	255
46	170	255	255	255
47	1	255	255	255
48	2	255	255	255
49	1	255	255	255
50	2	255	255	255
51	1	255	255	255
52	3056	255	255	255
53	721	255	255	255
54	38	255	255	255
55	1	255	255	255
56	1	255	255	255
57	60	255	255	255
58	83	255	255	255
59	195	255	255	255
60	53	255	255	255
61	1	255	255	255
62	4	255	255	255
63	2	255	255	255
64	1	255	255	255
65	9	255	255	255
66	5	255	255	255
67	1	255	255	255
68	1	255	255	255
69	3	255	255	255
70	1	255	255	255
71	1	255	255	255
72	2	255	255	255
73	1	255	255	255
74	7	255	255	255
75	1	255	255	255
76	1	255	255	255
77	2	255	255	255
78	1	255	255	255
79	1	255	255	255
80	2	255	255	255
81	2	255	255	255
82	1	255	255	255
83	195	255	255	255
84	5456	255	255	255
85	1	255	255	255
86	2	255	255	255
87	1	255	255	255
88	1	255	255	255
89	4	255	255	255
90	2034	255	255	255
91	1	255	255	255
92	1	255	255	255
93	1	255	255	255
94	4	255	255	255
95	1164	255	255	255
96	2	255	255	255
97	21	255	255	255
98	1	255	255	255
99	1	255	255	255
100	2	255	255	255
101	7	255	255	255
102	3	255	255	255
103	125	255	255	255
104	1	255	255	255
105	5	255	255	255
106	1	255	255	255
107	5	255	255	255
108	69	255	255	255
109	1	255	255	255
110	1	255	255	255
111	2	255	255	255
112	240	255	255	255
113	385	255	255	255
114	2	255	255	255
115	1	255	255	255
116	1	255	255	255
117	5	255	255	255
118	2	255	255	255
119	1	255	255	255

EoE
EoE patient esophageal biopsy tissue

Count				
Nuclear A	Total Area	Average Size		
1	1	255	255	255
2	1	255	255	255
3	57	255	255	255
4	1	255	255	255
5	1	255	255	255
6	1	255	255	255
7	2	255	255	255
8	3	255	255	255
9	1	255	255	255
10	8843	255	255	255
11	1	255	255	255
12	2	255	255	255
13	32	255	255	255
14	1	255	255	255
15	1	255	255	255
16	1	255	255	255
17	58	255	255	255
18	1	255	255	255
19	1	255	255	255
20	4	255	255	255
21	1	255	255	255
22	1	255	255	255
23	1	255	255	255
24	1	255	255	255
25	1	255	255	255
26	2	255	255	255
27	1	255	255	255
28	1	255	255	255
29	1	255	255	255
30	1	255	255	255
31	1	255	255	255
32	1	255	255	255
33	14	255	255	255
34	1	255	255	255
35	321	255	255	255
36	3	255	255	255
37	1	255	255	255
38	2	255	255	255
39	2	255	255	255
40	2	255	255	255
41	1	255	255	255
42	2	255	255	255
43	2	255	255	255
44	1	255	255	255
45	1	255	255	255
46	2	255	255	255
47	1	255	255	255
48	1	255	255	255
49	1	255	255	255
50	1	255	255	255
51	1	255	255	255
52	1	255	255	255
53	1	255	255	255
54	1	255	255	255
55	2	255	255	255
56	1	255	255	255
57	1	255	255	255
58	2	255	255	255
59	1	255	255	255
60	4	255	255	255
61	1	255	255	255
62	232	255	255	255
63	192	255	255	255
64	12	255	255	255
65	145	255	255	255
66	1	255	255	255
67	1	255	255	255
68	1	255	255	255
69	1	255	255	255
70	2736	255	255	255
71	1	255	255	255
72	2	255	255	255
73	1	255	255	255
74	23	255	255	255
75	1	255	255	255
76	1	255	255	255
77	2	255	255	255
78	1	255	255	255
79	570	255	255	255
80	1	255	255	255
81	50	255	255	255
82	1	255	255	255
83	3	255	255	255
84	2	255	255	255
85	1	255	255	255
86	1	255	255	255
87	210	255	255	255
88	1	255	255	255
89	1	255	255	255
90	2	255	255	255
91	1	255	255	255
92	1	255	255	255
93	1	255	255	255
94	1	255	255	255
95	235	255	255	255
96	2	255	255	255
97	3	255	255	255
98	2	255	255	255
99	6	255	255	255
100	2	255	255	255
101	1027	255	255	255
102	2	255	255	255
103	8	255	255	255
104	1	255	255	255
105	3	255	255	255
106	1	255	255	255
107	1	255	255	255
108	2	255	255	255
109	2	255	255	255
110	1	255	255	255
111	1	255	255	255
112	1	255	255	255
113	1	255	255	255
114	1307	255	255	255
115	2	255	255	255
116	1	255	255	255
117	1	255	255	255
118	1	255	255	255
119	1	255	255	255

EoE
EoE patient esophageal biopsy

Count	Total Area	Average Size		
Nuclear A	2	255	255	255
2	2	255	255	255
3	1	255	255	255
4	2	255	255	255
5	1	255	255	255
6	1	255	255	255
7	49	255	255	255
8	1	255	255	255
9	1	255	255	255
10	4	255	255	255
11	1	255	255	255
12	1	255	255	255
13	291	255	255	255
14	2	255	255	255
15	1	255	255	255
16	1	255	255	255
17	4	255	255	255
18	1	255	255	255
19	2	255	255	255
20	1	255	255	255
21	173	255	255	255
22	1	255	255	255
23	1	255	255	255
24	1	255	255	255
25	1	255	255	255
26	264	255	255	255
27	169	255	255	255
28	1	255	255	255
29	2	255	255	255
30	1	255	255	255
31	2	255	255	255
32	4	255	255	255
33	9	255	255	255
34	1	255	255	255
35	3	255	255	255
36	3	255	255	255
37	35	255	255	255
38	11	255	255	255
39	1	255	255	255
40	76	255	255	255
41	1	255	255	255
42	1	255	255	255
43	1	255	255	255
44	4	255	255	255
45	1	255	255	255
46	1	255	255	255
47	1	255	255	255
48	1511	255	255	255
49	3	255	255	255
50	2	255	255	255
51	1	255	255	255
52	72	255	255	255
53	9	255	255	255
54	1	255	255	255
55	53	255	255	255
56	1	255	255	255
57	1	255	255	255
58	95	255	255	255
59	1	255	255	255
60	2	255	255	255
61	269	255	255	255
62	1	255	255	255
63	1	255	255	255
64	1	255	255	255
65	5305	255	255	255
66	6	255	255	255
67	1	255	255	255
68	1	255	255	255
69	1	255	255	255
70	1	255	255	255
71	1	255	255	255
72	2	255	255	255
73	1	255	255	255
74	2	255	255	255
75	2	255	255	255
76	1	255	255	255
77	2	255	255	255
78	392	255	255	255
79	4	255	255	255
80	2908	255	255	255
81	4	255	255	255
82	1	255	255	255
83	1	255	255	255
84	1	255	255	255
85	129	255	255	255
86	1	255	255	255
87	3	255	255	255
88	1	255	255	255
89	3	255	255	255
90	287	255	255	255
91	7	255	255	255
92	2	255	255	255
93	1	255	255	255
94	1	255	255	255
95	1	255	255	255
96	2	255	255	255
97	1	255	255	255
98	2	255	255	255
99	2	255	255	255
100	2	255	255	255
101	1	255	255	255
102	1	255	255	255
103	1	255	255	255
104	2	255	255	255
105	1	255	255	255
106	3	255	255	255
107	33	255	255	255
108	1	255	255	255
109	1	255	255	255
110	29	255	255	255
111	1	255	255	255
112	1	255	255	255
113	1	255	255	255
114	929	255	255	255
115	72	255	255	255
116	3558	255	255	255
117	1	255	255	255
118	2	255	255	255
119	1	255	255	255

Original data for Figure 2D, Densitometric analysis of Nuclear/Perinuclear B-catenin in control and EoE patient esophageal biopsy tissue.

Values are representative of the mean of a sample size N=18. p<0.05**

120	118	255	255	255	120	64	255	255	255	120	3	255	255	255
121	1	255	255	255	121	1	255	255	255	121	1127	255	255	255
122	637	255	255	255	122	1	255	255	255	122	1	255	255	255
123	1	255	255	255	123	3	255	255	255	123	2	255	255	255
124	7	255	255	255	124	2	255	255	255	124	4	255	255	255
125	3	255	255	255	125	5	255	255	255	125	9	255	255	255
126	1	255	255	255	126	3	255	255	255	126	1	255	255	255
127	34	255	255	255	127	1	255	255	255	127	1	255	255	255
128	1	255	255	255	128	17	255	255	255	128	1	255	255	255
129	37	255	255	255	129	27	255	255	255	129	1	255	255	255
130	1	255	255	255	130	1	255	255	255	130	19	255	255	255
131	55	255	255	255	131	1	255	255	255	131	449	255	255	255
132	85	255	255	255	132	1	255	255	255	132	1	255	255	255
133	2	255	255	255	133	1	255	255	255	133	1	255	255	255
134	1	255	255	255	134	2	255	255	255	134	1	255	255	255
135	1	255	255	255	135	1	255	255	255	135	1	255	255	255
136	2	255	255	255	136	58	255	255	255	136	8	255	255	255
137	1	255	255	255	137	2	255	255	255	137	3	255	255	255
138	2	255	255	255	138	1	255	255	255	138	5	255	255	255
139	2	255	255	255	139	1	255	255	255	139	1	255	255	255
140	52	255	255	255	140	2952	255	255	255	140	1	255	255	255
141	2	255	255	255	141	1	255	255	255	141	7	255	255	255
142	52	255	255	255	142	4	255	255	255	142	1	255	255	255
143	364	255	255	255	143	4	255	255	255	143	56	255	255	255
144	620	255	255	255	144	1	255	255	255	144	1	255	255	255
145	3	255	255	255	145	228	255	255	255	145	1	255	255	255
146	1	255	255	255	146	1207	255	255	255	146	1	255	255	255
147	1	255	255	255	147	2	255	255	255	147	826	255	255	255
148	1	255	255	255	148	1	255	255	255	148	1	255	255	255
149	4	255	255	255	149	1	255	255	255	149	1	255	255	255
150	74	255	255	255	150	1	255	255	255	150	1	255	255	255
151	1	255	255	255	151	1	255	255	255	151	1	255	255	255
152	75	255	255	255	152	1	255	255	255	152	1	255	255	255
153	18	255	255	255	153	3	255	255	255	153	11	255	255	255
154	26	255	255	255	154	2	255	255	255	154	1	255	255	255
155	50	255	255	255	155	1	255	255	255	155	1	255	255	255
156	1	255	255	255	156	2	255	255	255	156	1	255	255	255
157	1	255	255	255	157	1	255	255	255	157	2	255	255	255
158	14	255	255	255	158	9	255	255	255	158	67	255	255	255
159	1	255	255	255	159	10191	255	255	255	159	814	255	255	255
160	156	255	255	255	160	1	255	255	255	160	281	255	255	255
161	1	255	255	255	161	1	255	255	255	161	1	255	255	255
162	1	255	255	255	162	540	255	255	255	162	1	255	255	255
163	1398	255	255	255	163	3	255	255	255	163	6	255	255	255
164	1	255	255	255	164	2	255	255	255	164	1	255	255	255
165	1	255	255	255	165	1	255	255	255	165	1	255	255	255
166	1	255	255	255	166	1	255	255	255	166	2	255	255	255
167	2	255	255	255	167	1	255	255	255	167	5	255	255	255
168	3	255	255	255	168	2	255	255	255	168	58	255	255	255
169	1	255	255	255	169	215	255	255	255	169	1	255	255	255
170	77	255	255	255	170	147	255	255	255	170	2259	255	255	255
171	1	255	255	255	171	1	255	255	255	171	1	255	255	255
172	6	255	255	255	172	62	255	255	255	172	1	255	255	255
173	1	255	255	255	173	1	255	255	255	173	1	255	255	255
174	1	255	255	255	174	1	255	255	255	174	1	255	255	255
175	87	255	255	255	175	2	255	255	255	175	2	255	255	255
176	2	255	255	255	176	1	255	255	255	176	1	255	255	255
177	99	255	255	255	177	1	255	255	255	177	1	255	255	255
178	8	255	255	255	178	3	255	255	255	178	2	255	255	255
179	79	255	255	255	179	1	255	255	255	179	1	255	255	255
180	2	255	255	255	180	1	255	255	255	180	1	255	255	255
181	2	255	255	255	181	108	255	255	255	181	3	255	255	255
182	2	255	255	255	182	3	255	255	255	182	354	255	255	255
183	3517	255	255	255	183	1	255	255	255	183	2	255	255	255
184	424	255	255	255	184	2	255	255	255	184	1	255	255	255
185	129	255	255	255	185	1	255	255	255	185	1	255	255	255
186	1	255	255	255	186	1	255	255	255	186	2	255	255	255
187	1	255	255	255	187	1	255	255	255	187	1	255	255	255
188	19	255	255	255	188	1	255	255	255	188	1	255	255	255
189	124	255	255	255	189	3	255	255	255	189	1	255	255	255
190	1	255	255	255	190	1	255	255	255	190	3554	255	255	255
191	1	255	255	255	191	2	255	255	255	191	2	255	255	255
192	1	255	255	255	192	1	255	255	255	192	638	255	255	255
193	1	255	255	255	193	1	255	255	255	193	1	255	255	255
194	1	255	255	255	194	1	255	255	255	194	509	255	255	255
195	139	255	255	255	195	1	255	255	255	195	131	255	255	255
196	3	255	255	255	196	3	255	255	255	196	2	255	255	255
197	1	255	255	255	197	1	255	255	255	197	1536	255	255	255
198	17	255	255	255	198	1	255	255	255	198	2	255	255	255
199	101	255	255	255	199	1	255	255	255	199	2	255	255	255
200	2	255	255	255	200	1	255	255	255	200	1555	255	255	255
201	1	255	255	255	201	5781	255	255	255	201	1	255	255	255
202	1227	255	255	255	202	2	255	255	255	202	1	255	255	255
203	1	255	255	255	203	1	255	255	255	203	1	255	255	255
204	1	255	255	255	204	1	255	255	255	204	2	255	255	255
205	1	255	255	255	205	6	255	255	255	205	1	255	255	255
206	1	255	255	255	206	1	255	255	255	206	294	255	255	255
207	1	255	255	255	207	15	255	255	255	207	1	255	255	255
208	1	255	255	255	208	2	255	255	255	208	614	255	255	255
209	2	255	255	255	209	1157	255	255	255	209	1	255	255	255
210	533	255	255	255	210	597	255	255	255	210	1	255	255	255
211	2	255	255	255	211	2	255	255	255	211	1	255	255	255
212	1	255	255	255	212	1	255	255	255	212	1	255	255	255
213	1	255	255	255	213	3	255	255	255	213	2	255	255	255
214	1	255	255	255	214	1	255	255	255	214	129	255	255	255
215	3	255	255	255	215	1	255	255	255	215	1	255	255	255
216	9	255	255	255	216	1	255	255	255	216	331	255	255	255
217	1	255	255	255	217	127	255	255	255	217	1	255	255	255
218	34	255	255	255	218	1	255	255	255	218	4	255	255	255
219	1	255	255	255	219	1	255	255	255	219	1	255	255	255
220	398	255	255	255	220	1	255	255	255	220	1	255	255	255
221	1	255	255	255	221	1	255	255	255	221	1	255	255	255
222	2	255	255	255	222	2	255	255	255	222	1	255	255	255
223	1	255	255	255	223	1	255	255	255	223	14	255	255	255
224	1	255	255	255	224	2	255	255	255	224	1	255	255	255
225	4	255	255	255	225	6	255	255	255	225	1	255	255	255
226	3	255	255	255	226	1	255	255	255	226	2	255	255	255
227	1	255	255	255	227	1	255	255	255	227	22	255	255	255
228	2	255	255	255	228	1	255	255	255	228	2	255	255	255
229	30	255	255	255	229	1	255	255	255	229	3	255	255	255
230	1	255	255	255	230	11	255	255	255	230	2	255	255	255
231	81	255	255	255	231	1	255	255	255	231	3	255	255	255
232	1	255	255	255	232	1	255	255	255	232	1	255	255	255
233	3	255	255	255	233	3	255	255	255	233	1	255	255	255
234	3	255	255	255	234	3	255	255	255	234	1542	255	255	255
235	3	255	255	255	235	1	255	255	255	235	597	255	255	255
236	1	255	255	255	236	1	255	255	255	236	1	255	255	255
237	623	255	255	255	237	84	255	255	255	237	2	255	255	255
238	1	255	255	255	238	1	255	255	255	238	12	255	255	255
239	1	255	255	255	239	2	255	255	255	239	1	255	255	255
240	1	255	255	255	240	1	255	255	255	240	111	255	255	255
241	1	255	255	255	241	1	255	255	255	241	1	255	255	255
242	2402	255	255	255	242	2	255	255	255	242	481	255	255	255
243	26	255	255	255	243	1	255	255	255	243	1	255	255	255
244	1	255	255	255	244	1	255	255	255	244	166	255	255	255
245	89	255	255	255	245	1	255	255	255	245	1	255	255	255
246	1	255	255	255	246	4321	255	255	255	246	1	255	255	255
247	1	255	255	255	247	1	255	255	255	247	1	255	255	255
248	3	255	255	255	248	2	255	255	255	248	1	255	255	255
249	1	255	255	255	249	1	255	255	255	249	1	255	255	255
250	2	255	255	255	250	2	255	255	255	250	1	255	255	255
251	857	255	255	255	251	1	255	255	255	251	496	255	255	255
252	1	255	255	255	252	148	255	255	255	252	1	255	255	255
253	2	255	255	255	253	1	255	255	255	253	1	255	255	255
254	1	255	255	255	254	1	255	255	255	254	1	255	255	255
255	4	255	255	255	255	1	255	255	255	255	1	255	255	255
256	3485	255	255	255	256	727	255	255	255	256	1	255	255	255
257	5	255	255	255	257	2	255	255	255	257	1	255	255	255

Original data for Figure 2D, Densitometric analysis of Nuclear/Perinuclear B-catenin in control and EoE patient esophageal biopsy tissue.
Values are representative of the mean of a sample size N=18. p<0.05**

258	1	255	255	255	258	2	255	255	255	258	2	255	255	255
259	461	255	255	255	259	1	255	255	255	259	2	255	255	255
260	1	255	255	255	260	1	255	255	255	260	1117	255	255	255
261	13	255	255	255	261	1	255	255	255	261	3047	255	255	255
262	1	255	255	255	262	8	255	255	255	262	137	255	255	255
263	71	255	255	255	263	1	255	255	255	263	235	255	255	255
264	1	255	255	255	264	1	255	255	255	264	1	255	255	255
265	1	255	255	255	265	1	255	255	255	265	2	255	255	255
266	8704	255	255	255	266	1	255	255	255	266	1	255	255	255
267	1	255	255	255	267	543	255	255	255	267	2	255	255	255
268	2	255	255	255	268	2	255	255	255	268	560	255	255	255
269	1	255	255	255	269	1	255	255	255	269	1	255	255	255
270	4447	255	255	255	270	1	255	255	255	270	1	255	255	255
271	1	255	255	255	271	136	255	255	255	271	2	255	255	255
272	1	255	255	255	272	1	255	255	255	272	233	255	255	255
273	6	255	255	255	273	1	255	255	255	273	1	255	255	255
274	76	255	255	255	274	7	255	255	255	274	85	255	255	255
275	1	255	255	255	275	1	255	255	255	275	5	255	255	255
276	103	255	255	255	276	1	255	255	255	276	1	255	255	255
277	1	255	255	255	277	1	255	255	255	277	1	255	255	255
278	1	255	255	255	278	696	255	255	255	278	1	255	255	255
279	243	255	255	255	279	1	255	255	255	279	1	255	255	255
280	2	255	255	255	280	1	255	255	255	280	2	255	255	255
281	58	255	255	255	281	1	255	255	255	281	4	255	255	255
282	1	255	255	255	282	1578	255	255	255	282	46	255	255	255
283	1	255	255	255	283	1	255	255	255	283	1846	255	255	255
284	1	255	255	255	284	3262	255	255	255	284	1	255	255	255
285	138	255	255	255	285	15	255	255	255	285	1	255	255	255
286	2	255	255	255	286	1	255	255	255	286	6	255	255	255
287	2	255	255	255	287	1	255	255	255	287	3	255	255	255
288	66	255	255	255	288	1	255	255	255	288	1	255	255	255
289	1	255	255	255	289	1	255	255	255	289	20	255	255	255
290	3	255	255	255	290	1	255	255	255	290	1	255	255	255
291	2	255	255	255	291	1	255	255	255	291	222	255	255	255
292	1	255	255	255	292	1	255	255	255	292	1	255	255	255
293	1	255	255	255	293	40	255	255	255	293	1	255	255	255
294	1	255	255	255	294	2	255	255	255	294	1	255	255	255
295	17	255	255	255	295	2	255	255	255	295	36	255	255	255
296	1	255	255	255	296	1	255	255	255	296	375	255	255	255
297	1	255	255	255	297	1	255	255	255	297	1	255	255	255
298	1	255	255	255	298	2	255	255	255	298	3227	255	255	255
299	2	255	255	255	299	3	255	255	255	299	1	255	255	255
300	1	255	255	255	300	1	255	255	255	300	1	255	255	255
301	2	255	255	255	301	1	255	255	255	301	26	255	255	255
302	1	255	255	255	302	1	255	255	255	302	2	255	255	255
303	1195	255	255	255	303	1	255	255	255	303	1	255	255	255
304	3	255	255	255	304	1	255	255	255	304	18	255	255	255
305	1	255	255	255	305	1	255	255	255	305	1	255	255	255
306	11	255	255	255	306	1	255	255	255	306	3	255	255	255
307	1	255	255	255	307	1	255	255	255	307	105	255	255	255
308	1	255	255	255	308	2	255	255	255	308	4	255	255	255
309	169	255	255	255	309	1	255	255	255	309	1	255	255	255
310	1	255	255	255	310	1	255	255	255	310	1	255	255	255
311	2	255	255	255	311	1	255	255	255	311	4	255	255	255
312	1	255	255	255	312	1	255	255	255	312	1	255	255	255
313	2	255	255	255	313	1	255	255	255	313	1259	255	255	255
314	1866	255	255	255	314	1	255	255	255	314	344	255	255	255
315	6	255	255	255	315	1	255	255	255	315	2680	255	255	255
316	1	255	255	255	316	1	255	255	255	316	740	255	255	255
317	273	255	255	255	317	3	255	255	255	317	1	255	255	255
318	1	255	255	255	318	1	255	255	255	318	1	255	255	255
319	766	255	255	255	319	2	255	255	255	319	1646	255	255	255
320	2	255	255	255	320	1	255	255	255	320	2	255	255	255
321	1	255	255	255	321	10	255	255	255	321	1	255	255	255
322	1	255	255	255	322	1	255	255	255	322	23	255	255	255
323	1	255	255	255	323	3	255	255	255	323	1	255	255	255
324	1464	255	255	255	324	1	255	255	255	324	1	255	255	255
325	2	255	255	255	325	1	255	255	255	325	1	255	255	255
326	1	255	255	255	326	2	255	255	255	326	1	255	255	255
327	6	255	255	255	327	16	255	255	255	327	1	255	255	255
328	1	255	255	255	328	1	255	255	255	328	3539	255	255	255
329	24	255	255	255	329	3	255	255	255	329	1	255	255	255
330	2	255	255	255	330	1	255	255	255	330	2	255	255	255
331	1	255	255	255	331	1	255	255	255	331	1	255	255	255
332	80	255	255	255	332	1	255	255	255	332	1	255	255	255
333	1	255	255	255	333	1	255	255	255	333	1	255	255	255
334	1	255	255	255	334	278	255	255	255	334	1	255	255	255
335	58	255	255	255	335	1	255	255	255	335	2	255	255	255
336	144	255	255	255	336	2	255	255	255	336	1	255	255	255
337	1	255	255	255	337	1	255	255	255	337	1	255	255	255
338	26	255	255	255	338	1	255	255	255	338	1	255	255	255
339	2	255	255	255	339	5	255	255	255	339	4	255	255	255
340	3	255	255	255	340	1	255	255	255	340	120	255	255	255
341	1	255	255	255	341	1	255	255	255	341	1	255	255	255
342	187	255	255	255	342	1	255	255	255	342	7	255	255	255
343	1	255	255	255	343	2	255	255	255	343	1	255	255	255
344	1	255	255	255	344	1	255	255	255	344	1	255	255	255
345	1	255	255	255	345	4	255	255	255	345	342	255	255	255
346	1	255	255	255	346	362	255	255	255	346	1	255	255	255
347	2	255	255	255	347	1	255	255	255	347	1	255	255	255
348	282	255	255	255	348	1	255	255	255	348	1	255	255	255
349	2	255	255	255	349	20	255	255	255	349	1	255	255	255
350	2	255	255	255	350	1	255	255	255	350	1	255	255	255
351	5	255	255	255	351	1	255	255	255	351	4	255	255	255
352	1	255	255	255	352	343	255	255	255	352	2	255	255	255
353	3	255	255	255	353	5	255	255	255	353	1	255	255	255
354	10	255	255	255	354	1	255	255	255	354	1360	255	255	255
355	72	255	255	255	355	2	255	255	255	355	1	255	255	255
356	19	255	255	255	356	1	255	255	255	356	347	255	255	255
357	1	255	255	255	357	1	255	255	255	357	83	255	255	255
358	3	255	255	255	358	253	255	255	255	358	1	255	255	255
359	2	255	255	255	359	97	255	255	255	359	1	255	255	255
360	1	255	255	255	360	1	255	255	255	360	1	255	255	255
361	1	255	255	255	361	1	255	255	255	361	79	255	255	255
362	1	255	255	255	362	2	255	255	255	362	133	255	255	255
363	63	255	255	255	363	1	255	255	255	363	1	255	255	255
364	333	255	255	255	364	1	255	255	255	364	1	255	255	255
365	1	255	255	255	365	1	255	255	255	365	26	255	255	255
366	1	255	255	255	366	1	255	255	255	366	150	255	255	255
367	1	255	255	255	367	1	255	255	255	367	1	255	255	255
368	329	255	255	255	368	1	255	255	255	368	1	255	255	255
369	2	255	255	255	369	3	255	255	255	369	3	255	255	255
370	4	255	255	255	370	1	255	255	255	370	36	255	255	255
371	2	255	255	255	371	2	255	255	255	371	1	255	255	255
372	5035	255	255	255	372	4	255	255	255	372	1	255	255	255
373	849	255	255	255	373	644	255	255	255	373	1	255	255	255
374	81	255	255	255	374	1	255	255	255	374	270	255	255	255
375	16	255	255	255	375	1	255	255	255	375	1	255	255	255
376	1	255	255	255	376	1	255	255	255	376	2	255	255	255
377	24	255	255	255	377	3	255	255	255	377	1	255	255	255
378	8	255	255	255	378	1	255	255	255	378	1	255	255	255
379	1	255	255	255	379	370	255	255	255	379	2	255	255	255
380	1	255	255	255	380	1	255	255	255	380	1	255	255	255
381	1	255	255	255	381	7	255	255	255	381	1672	255	255	255
382	56	255	255	255	382	1	255	255	255	382	6	255	255	255
383	1	255	255	255	383	1	255	255	255	383	658	255	255	255
384	1	255	255	255	384	1	255	255	255	384	5	255	255	255
385	1	255	255	255	385	276	255	255	255	385	1139	255	255	255
386	357	255	255	255	386	75	255	255	255	386	1512	255	255	255
387	3	255	255	255	387	5	255	255	255	387	1	255	255	255
388	4	255	255	255	388	8	255	255	255	388	2	255	255	255
389	759	255	255	255	389	1	255	255	255	389	5	255	255	255
390	1	255	255	255	390	1	255	255	255	390	2	255	255	255
391	9	255	255	255	391	469	255	255	255	391	1	255	255	255
392	1	255	255	255	392	1	255	255	255	392	1	255	255	255
393	2	255	255	255	393	2	255	255	255	393	2	255	255	255
394	1	255	255	255	394	2	255	255	255	394	2	255	255	255
395	121	255	255	255	395	1	255	255	255	395	1	255	255	255

Original data for Figure 2D, Densitometric analysis of Nuclear/Perinuclear B-catenin in control and EoE patient esophageal biopsy tissue.

Values are representative of the mean of a sample size N=18. p<0.05**

396	1	255	255	255	396	1	255	255	255	396	1	255	255	255
397	1	255	255	255	397	2	255	255	255	397	1	255	255	255
398	1	255	255	255	398	2	255	255	255	398	1	255	255	255
399	1	255	255	255	399	1	255	255	255	399	2	255	255	255
400	46	255	255	255	400	1	255	255	255	400	1	255	255	255
401	88	255	255	255	401	2	255	255	255	401	280	255	255	255
402	4	255	255	255	402	1	255	255	255	402	2	255	255	255
403	2	255	255	255	403	2	255	255	255	403	2	255	255	255
404	1	255	255	255	404	2	255	255	255	404	13	255	255	255
405	2	255	255	255	405	1	255	255	255	405	1	255	255	255
406	168	255	255	255	406	3	255	255	255	406	3739	255	255	255
407	1	255	255	255	407	48	255	255	255	407	1	255	255	255
408	2	255	255	255	408	1	255	255	255	408	2221	255	255	255
409	176	255	255	255	409	1	255	255	255	409	1	255	255	255
410	3589	255	255	255	410	27	255	255	255	410	1	255	255	255
411	1	255	255	255	411	2	255	255	255	411	2	255	255	255
412	2	255	255	255	412	1	255	255	255	412	1	255	255	255
413	3044	255	255	255	413	1	255	255	255	413	2	255	255	255
414	1	255	255	255	414	1	255	255	255	414	1	255	255	255
415	166	255	255	255	415	3	255	255	255	415	215	255	255	255
416	3	255	255	255	416	2	255	255	255	416	74	255	255	255
417	135	255	255	255	417	1	255	255	255	417	2	255	255	255
418	351	255	255	255	418	51	255	255	255	418	1	255	255	255
419	3	255	255	255	419	2087	255	255	255	419	2	255	255	255
420	19	255	255	255	420	33	255	255	255	420	1	255	255	255
421	1	255	255	255	421	1	255	255	255	421	1	255	255	255
422	1	255	255	255	422	1	255	255	255	422	124	255	255	255
423	2	255	255	255	423	12	255	255	255	423	1	255	255	255
424	5	255	255	255	424	1	255	255	255	424	3	255	255	255
425	3	255	255	255	425	1	255	255	255	425	1	255	255	255
426	1	255	255	255	426	1	255	255	255	426	2	255	255	255
427	63	255	255	255	427	2	255	255	255	427	2	255	255	255
428	2	255	255	255	428	1	255	255	255	428	20	255	255	255
429	116	255	255	255	429	1	255	255	255	429	1	255	255	255
430	832	255	255	255	430	5	255	255	255	430	10	255	255	255
431	1	255	255	255	431	8	255	255	255	431	115	255	255	255
432	352	255	255	255	432	2	255	255	255	432	1	255	255	255
433	79	255	255	255	433	10	255	255	255	433	1	255	255	255
434	2	255	255	255	434	64	255	255	255	434	1172	255	255	255
435	3	255	255	255	435	17	255	255	255	435	2	255	255	255
436	159	255	255	255	436	2	255	255	255	436	1	255	255	255
437	2	255	255	255	437	1	255	255	255	437	1409	255	255	255
438	1	255	255	255	438	1	255	255	255	438	2	255	255	255
439	1	255	255	255	439	1	255	255	255	439	1	255	255	255
440	578	255	255	255	440	4	255	255	255	440	2	255	255	255
441	1	255	255	255	441	1	255	255	255	441	1	255	255	255
442	80	255	255	255	442	2	255	255	255	442	27	255	255	255
443	1	255	255	255	443	12	255	255	255	443	47	255	255	255
444	2	255	255	255	444	1	255	255	255	444	1	255	255	255
445	2721	255	255	255	445	1	255	255	255	445	2	255	255	255
446	1	255	255	255	446	1	255	255	255	446	2	255	255	255
447	1	255	255	255	447	1	255	255	255	447	1	255	255	255
448	1	255	255	255	448	1	255	255	255	448	5	255	255	255
449	1	255	255	255	449	1	255	255	255	449	1	255	255	255
450	78	255	255	255	450	1	255	255	255	450	1	255	255	255
451	1	255	255	255	451	1	255	255	255	451	291	255	255	255
452	1	255	255	255	452	560	255	255	255	452	1	255	255	255
453	1	255	255	255	453	1	255	255	255	453	1	255	255	255
454	1	255	255	255	454	1	255	255	255	454	3	255	255	255
455	1	255	255	255	455	1	255	255	255	455	1	255	255	255
456	5	255	255	255	456	1	255	255	255	456	2	255	255	255
457	2	255	255	255	457	1	255	255	255	457	2	255	255	255
458	1	255	255	255	458	2	255	255	255	458	5	255	255	255
459	848	255	255	255	459	1	255	255	255	459	5	255	255	255
460	75	255	255	255	460	1	255	255	255	460	1	255	255	255
461	287	255	255	255	461	1	255	255	255	461	1097	255	255	255
462	1	255	255	255	462	2	255	255	255	462	1	255	255	255
463	1	255	255	255	463	1	255	255	255	463	246	255	255	255
464	1	255	255	255	464	1	255	255	255	464	1	255	255	255
465	2585	255	255	255	465	4	255	255	255	465	1	255	255	255
466	1683	255	255	255	466	1	255	255	255	466	1	255	255	255
467	1	255	255	255	467	390	255	255	255	467	1	255	255	255
468	1	255	255	255	468	3	255	255	255	468	67	255	255	255
469	3	255	255	255	469	8	255	255	255	469	7	255	255	255
470	2	255	255	255	470	3	255	255	255	470	1	255	255	255
471	3	255	255	255	471	155	255	255	255	471	1	255	255	255
472	1	255	255	255	472	1	255	255	255	472	1	255	255	255
473	54	255	255	255	473	1	255	255	255	473	41	255	255	255
474	1	255	255	255	474	1	255	255	255	474	2984	255	255	255
475	1	255	255	255	475	2	255	255	255	475	1	255	255	255
476	224	255	255	255	476	226	255	255	255	476	1	255	255	255
477	1	255	255	255	477	1	255	255	255	477	3	255	255	255
478	3	255	255	255	478	1	255	255	255	478	1	255	255	255
479	958	255	255	255	479	1	255	255	255	479	1	255	255	255
480	1	255	255	255	480	2	255	255	255	480	2	255	255	255
481	4	255	255	255	481	1	255	255	255	481	1	255	255	255
482	3	255	255	255	482	1	255	255	255	482	1	255	255	255
483	1	255	255	255	483	3	255	255	255	483	89	255	255	255
484	1	255	255	255	484	5	255	255	255	484	1	255	255	255
485	744	255	255	255	485	1	255	255	255	485	1	255	255	255
486	28	255	255	255	486	1	255	255	255	486	1053	255	255	255
487	3	255	255	255	487	1	255	255	255	487	85	255	255	255
488	1	255	255	255	488	1	255	255	255	488	2	255	255	255
489	1	255	255	255	489	2	255	255	255	489	1	255	255	255
490	1	255	255	255	490	2	255	255	255	490	1	255	255	255
491	4	255	255	255	491	1047	255	255	255	491	1	255	255	255
492	1	255	255	255	492	1	255	255	255	492	1	255	255	255
493	4	255	255	255	493	1	255	255	255	493	1	255	255	255
494	26	255	255	255	494	1	255	255	255	494	3	255	255	255
495	2	255	255	255	495	1	255	255	255	495	10	255	255	255
496	2	255	255	255	496	57	255	255	255	496	14	255	255	255
497	54	255	255	255	497	1	255	255	255	497	1	255	255	255
498	1	255	255	255	498	1	255	255	255	498	1	255	255	255
499	1	255	255	255	499	1	255	255	255	499	1	255	255	255
500	90	255	255	255	500	129	255	255	255	500	1	255	255	255
501	46	255	255	255	501	1	255	255	255	501	815	255	255	255
502	1	255	255	255	502	4	255	255	255	502	3	255	255	255
503	3	255	255	255	503	1	255	255	255	503	1	255	255	255
504	2	255	255	255	504	2	255	255	255	504	4	255	255	255
505	1	255	255	255	505	1	255	255	255	505	1	255	255	255
506	2	255	255	255	506	1	255	255	255	506	2	255	255	255
507	1	255	255	255	507	2	255	255	255	507	3	255	255	255
508	76	255	255	255	508	1	255	255	255	508	1	255	255	255
509	1	255	255	255	509	3	255	255	255	509	3	255	255	255
510	1	255	255	255	510	2	255	255	255	510	1	255	255	255
511	1	255	255	255	511	2	255	255	255	511	3	255	255	255
512	38	255	255	255	512	1	255	255	255	512	1	255	255	255
513	1	255	255	255	513	976	255	255	255	513	1	255	255	255
514	1	255	255	255	514	212	255	255	255	514	2	255	255	255
515	1	255	255	255	515	4	255	255	255	515	1	255	255	255
516	1	255	255	255	516	383	255	255	255	516	2	255	255	255
517	54	255	255	255	517	2	255	255	255	517	9	255	255	255
518	214	255	255	255	518	1	255	255	255	518	10	255	255	255
519	1	255	255	255	519	1	255	255	255	519	1	255	255	255
520	2	255	255	255	520	1	255	255	255	520	2520	255	255	255
521	74	255	255	255	521	1	255	255	255	521	395	255	255	255
522	1	255	255	255	522	2	255	255	255	522	2	255	255	255
523	212	255	255	255	523	1	255	255	255	523	1	255	255	255
524	6	255	255	255	524	1	255	255	255	524	2	255	255	255
525	20	255	255	255	525	66	255	255	255	525	6	255	255	255
526	3219	255	255	255	526	34	255	255	255	526	1	255	255	255
527	60	255	255	255	527	3	255	255	255	527	1	255	255	255
528	4	255	255	255	528	1	255	255	255	528	2	255	255	255
529	1	255	255	255	529	1	255	255	255	529	2	255	255	255
530	236	255	255	255	530	1	255	255	255	530	2	255	255	255
531	2	255	255	255	531	1	255	255	255	531	2	255	255	255
532	5	255	255	255	532	1	255	255	255	532	1	255	255	255
533	1	255	255	255	533	1	255	255	255	533	1	255	255	255

Original data for Figure 2D, Densitometric analysis of Nuclear/Perinuclear B-catenin in control and EoE patient esophageal biopsy tissue.

Values are representative of the mean of a sample size N=18. p<0.05**

534	2	255	255	255	534	1	255	255	255	534	197	255	255	255
535	460	255	255	255	535	1	255	255	255	535	286	255	255	255
536	1	255	255	255	536	2	255	255	255	536	1	255	255	255
537	1	255	255	255	537	1	255	255	255	537	1	255	255	255
538	1	255	255	255	538	1	255	255	255	538	5	255	255	255
539	3	255	255	255	539	1	255	255	255	539	345	255	255	255
540	33	255	255	255	540	741	255	255	255	540	1	255	255	255
541	2	255	255	255	541	2	255	255	255	541	208	255	255	255
542	2	255	255	255	542	327	255	255	255	542	6	255	255	255
543	6	255	255	255	543	180	255	255	255	543	3	255	255	255
544	704	255	255	255	544	2	255	255	255	544	1	255	255	255
545	65	255	255	255	545	1	255	255	255	545	329	255	255	255
546	1	255	255	255	546	1	255	255	255	546	1	255	255	255
547	1	255	255	255	547	38	255	255	255	547	3	255	255	255
548	123	255	255	255	548	1	255	255	255	548	378	255	255	255
549	49	255	255	255	549	1	255	255	255	549	1	255	255	255
550	2	255	255	255	550	176	255	255	255	550	4307	255	255	255
551	2	255	255	255	551	1	255	255	255	551	2	255	255	255
552	1	255	255	255	552	2	255	255	255	552	2	255	255	255
553	4	255	255	255	553	622	255	255	255	553	1	255	255	255
554	1	255	255	255	554	3	255	255	255	554	1	255	255	255
555	1	255	255	255	555	3	255	255	255	555	2	255	255	255
556	34	255	255	255	556	1	255	255	255	556	2	255	255	255
557	5	255	255	255	557	3	255	255	255	557	1	255	255	255
558	666	255	255	255	558	3	255	255	255	558	1	255	255	255
559	2	255	255	255	559	1	255	255	255	559	1	255	255	255
560	358	255	255	255	560	2	255	255	255	560	1	255	255	255
561	3	255	255	255	561	122	255	255	255	561	3	255	255	255
562	2	255	255	255	562	1	255	255	255	562	245	255	255	255
563	458	255	255	255	563	2	255	255	255	563	1	255	255	255
564	138	255	255	255	564	1	255	255	255	564	10	255	255	255
565	1	255	255	255	565	1	255	255	255	565	2	255	255	255
566	60	255	255	255	566	5	255	255	255	566	1	255	255	255
567	8	255	255	255	567	3	255	255	255	567	1	255	255	255
568	44	255	255	255	568	1	255	255	255	568	4	255	255	255
569	6	255	255	255	569	197	255	255	255	569	2	255	255	255
570	1	255	255	255	570	1	255	255	255	570	2	255	255	255
571	12	255	255	255	571	298	255	255	255	571	1	255	255	255
572	6	255	255	255	572	1	255	255	255	572	1	255	255	255
573	1	255	255	255	573	103	255	255	255	573	1	255	255	255
574	2	255	255	255	574	1	255	255	255	574	2	255	255	255
575	2	255	255	255	575	2	255	255	255	575	1	255	255	255
576	2	255	255	255	576	1	255	255	255	576	283	255	255	255
577	67	255	255	255	577	1	255	255	255	577	3	255	255	255
578	1	255	255	255	578	344	255	255	255	578	1	255	255	255
579	1	255	255	255	579	1	255	255	255	579	13	255	255	255
580	2	255	255	255	580	1	255	255	255	580	23	255	255	255
581	185	255	255	255	581	1	255	255	255	581	1	255	255	255
582	2	255	255	255	582	1	255	255	255	582	1018	255	255	255
583	2	255	255	255	583	1	255	255	255	583	1	255	255	255
584	1	255	255	255	584	1	255	255	255	584	25	255	255	255
585	16	255	255	255	585	106	255	255	255	585	1	255	255	255
586	2	255	255	255	586	1	255	255	255	586	1	255	255	255
587	69	255	255	255	587	1	255	255	255	587	34	255	255	255
588	107	255	255	255	588	3	255	255	255	588	1	255	255	255
589	1	255	255	255	589	6	255	255	255	589	1	255	255	255
590	5	255	255	255	590	7	255	255	255	590	1	255	255	255
591	2	255	255	255	591	1	255	255	255	591	798	255	255	255
592	36	255	255	255	592	2	255	255	255	592	1	255	255	255
593	1	255	255	255	593	1	255	255	255	593	1	255	255	255
594	1	255	255	255	594	1	255	255	255	594	2	255	255	255
595	37	255	255	255	595	2	255	255	255	595	1	255	255	255
596	134	255	255	255	596	2	255	255	255	596	1	255	255	255
597	51	255	255	255	597	825	255	255	255	597	1	255	255	255
598	1	255	255	255	598	1	255	255	255	598	1	255	255	255
599	1	255	255	255	599	1	255	255	255	599	1	255	255	255
600	3	255	255	255	600	37	255	255	255	600	4939	255	255	255
601	1	255	255	255	601	1	255	255	255	601	1	255	255	255
602	1	255	255	255	602	3	255	255	255	602	1	255	255	255
603	1	255	255	255	603	3	255	255	255	603	12	255	255	255
604	3	255	255	255	604	1	255	255	255	604	220	255	255	255
605	1	255	255	255	605	1	255	255	255	605	2	255	255	255
606	1	255	255	255	606	1	255	255	255	606	1	255	255	255
607	1	255	255	255	607	1	255	255	255	607	1	255	255	255
608	21	255	255	255	608	1	255	255	255	608	1	255	255	255
609	8	255	255	255	609	1	255	255	255	609	3	255	255	255
610	1	255	255	255	610	1	255	255	255	610	73	255	255	255
611	1	255	255	255	611	96	255	255	255	611	1	255	255	255
612	1	255	255	255	612	1	255	255	255	612	1	255	255	255
613	1	255	255	255	613	2944	255	255	255	613	1	255	255	255
614	3	255	255	255	614	3	255	255	255	614	1	255	255	255
615	2	255	255	255	615	1	255	255	255	615	182	255	255	255
616	2	255	255	255	616	6	255	255	255	616	457	255	255	255
617	3	255	255	255	617	1	255	255	255	617	3	255	255	255
618	1	255	255	255	618	1	255	255	255	618	84	255	255	255
619	6	255	255	255	619	1	255	255	255	619	10	255	255	255
620	34	255	255	255	620	1	255	255	255	620	3	255	255	255
621	1	255	255	255	621	1	255	255	255	621	3	255	255	255
622	1	255	255	255	622	1	255	255	255	622	1	255	255	255
623	109	255	255	255	623	212	255	255	255	623	2	255	255	255
624	2	255	255	255	624	1	255	255	255	624	1080	255	255	255
625	1	255	255	255	625	1	255	255	255	625	1	255	255	255
626	1	255	255	255	626	2	255	255	255	626	1	255	255	255
627	1	255	255	255	627	1	255	255	255	627	1	255	255	255
628	2	255	255	255	628	1	255	255	255	628	3	255	255	255
629	1	255	255	255	629	1	255	255	255	629	1	255	255	255
630	3	255	255	255	630	155	255	255	255	630	1	255	255	255
631	2	255	255	255	631	1	255	255	255	631	1	255	255	255
632	1	255	255	255	632	1	255	255	255	632	107	255	255	255
633	1	255	255	255	633	1	255	255	255	633	77	255	255	255
634	1	255	255	255	634	2	255	255	255	634	1	255	255	255
635	18	255	255	255	635	1	255	255	255	635	1	255	255	255
636	1	255	255	255	636	1	255	255	255	636	1	255	255	255
637	152	255	255	255	637	2	255	255	255	637	1	255	255	255
638	1	255	255	255	638	1	255	255	255	638	1	255	255	255
639	1	255	255	255	639	1	255	255	255	639	1	255	255	255
640	1	255	255	255	640	118	255	255	255	640	1	255	255	255
641	373	255	255	255	641	1	255	255	255	641	2	255	255	255
642	32	255	255	255	642	1	255	255	255	642	50	255	255	255
643	199	255	255	255	643	362	255	255	255	643	1	255	255	255
644	53	255	255	255	644	1	255	255	255	644	1	255	255	255
645	2	255	255	255	645	20	255	255	255	645	37	255	255	255
646	552	255	255	255	646	1	255	255	255	646	2	255	255	255
647	2	255	255	255	647	1	255	255	255	647	4	255	255	255
648	1	255	255	255	648	1	255	255	255	648	1	255	255	255
649	1	255	255	255	649	1	255	255	255	649	1	255	255	255
650	1	255	255	255	650	2	255	255	255	650	4	255	255	255
651	94	255	255	255	651	25	255	255	255	651	2	255	255	255
652	2	255	255	255	652	1	255	255	255	652	1	255	255	255
653	1	255	255	255	653	185	255	255	255	653	1	255	255	255
654	1	255	255	255	654	1	255	255	255	654	1	255	255	255
655	1	255	255	255	655	208	255	255	255	655	726	255	255	255
656	1	255	255	255	656	69	255	255	255	656	1	255	255	255
657	134	255	255	255	657	76	255	255	255	657	1	255	255	255
658	1	255	255	255	658	1	255	255	255	658	42	255	255	255
659	391	255	255	255	659	3	255	255	255	659	1	255	255	255
660	3	255	255	255	660	1	255	255	255	660	1	255	255	255
661	2	255	255	255	661	1	255	255	255	661	1	255	255	255
662	1	255	255	255	662	2	255	255	255	662	1	255	255	255
663	4193	255	255	255	663	1	255	255	255	663	1	255	255	255
664	5	255	255	255	664	1	255	255	255	664	1	255	255	255
665	3	255	255	255	665	3	255	255	255	665	1	255	255	255
666	2	255	255	255	666	1	255	255	255	666	1	255	255	255
667	1	255	255	255	667	1	255	255	255	667	524	255	255	255
668	1	255	255	255	668	2	255	255	255	668	28	255	255	255
669	1	255	255	255	669	1	255	255	255	669	1	255	255	255
670	1	255	255	255	670	9	255	255	255	670	1	255	255	255
671	1	255	255	255	671	1	255	255	255	671	1	255	255	255

Original data for Figure 2D, Densitometric analysis of Nuclear/Perinuclear B-catenin in control and EoE patient esophageal biopsy tissue.
Values are representative of the mean of a sample size N=18. p<0.05**

672	1	255	255	255	672	5	255	255	255	672	1	255	255	255
673	2534	255	255	255	673	66	255	255	255	673	2	255	255	255
674	1	255	255	255	674	1899	255	255	255	674	1	255	255	255
675	1	255	255	255	675	1	255	255	255	675	1	255	255	255
676	1	255	255	255	676	1	255	255	255	676	1	255	255	255
677	1	255	255	255	677	1	255	255	255	677	3516	255	255	255
678	1	255	255	255	678	1	255	255	255	678	1	255	255	255
679	12	255	255	255	679	5	255	255	255	679	1	255	255	255
680	1	255	255	255	680	4	255	255	255	680	1	255	255	255
681	1	255	255	255	681	1	255	255	255	681	5269	255	255	255
682	1	255	255	255	682	1	255	255	255	682	2	255	255	255
683	500	255	255	255	683	2	255	255	255	683	2503	255	255	255
684	1	255	255	255	684	1	255	255	255	684	1	255	255	255
685	7	255	255	255	685	1	255	255	255	685	121	255	255	255
686	1	255	255	255	686	1	255	255	255	686	1	255	255	255
687	1	255	255	255	687	6	255	255	255	687	1	255	255	255
688	1	255	255	255	688	1	255	255	255	688	1	255	255	255
689	56	255	255	255	689	3	255	255	255	689	85	255	255	255
690	1	255	255	255	690	1	255	255	255	690	1	255	255	255
691	12	255	255	255	691	283	255	255	255	691	1255	255	255	255
692	1	255	255	255	692	7	255	255	255	692	941	255	255	255
693	4	255	255	255	693	63	255	255	255	693	199	255	255	255
694	78	255	255	255	694	1	255	255	255	694	1	255	255	255
695	1	255	255	255	695	1	255	255	255	695	1	255	255	255
696	3	255	255	255	696	2	255	255	255	696	1	255	255	255
697	1	255	255	255	697	1	255	255	255	697	1	255	255	255
698	1	255	255	255	698	1	255	255	255	698	2	255	255	255
699	1	255	255	255	699	1	255	255	255	699	1	255	255	255
700	1	255	255	255	700	3	255	255	255	700	109	255	255	255
701	2	255	255	255	701	1	255	255	255	701	146	255	255	255
702	2	255	255	255	702	104	255	255	255	702	4	255	255	255
703	3	255	255	255	703	11	255	255	255	703	1	255	255	255
704	1	255	255	255	704	5	255	255	255	704	1	255	255	255
705	2	255	255	255	705	1	255	255	255	705	2	255	255	255
706	130	255	255	255	706	2	255	255	255	706	359	255	255	255
707	3	255	255	255	707	515	255	255	255	707	284	255	255	255
708	1	255	255	255	708	85	255	255	255	708	2	255	255	255
709	209	255	255	255	709	1	255	255	255	709	3	255	255	255
710	1	255	255	255	710	1	255	255	255	710	7	255	255	255
711	2	255	255	255	711	661	255	255	255	711	1759	255	255	255
712	409	255	255	255	712	1	255	255	255	712	2	255	255	255
713	5	255	255	255	713	104	255	255	255	713	2	255	255	255
714	1	255	255	255	714	895	255	255	255	714	1	255	255	255
715	1	255	255	255	715	1	255	255	255	715	7	255	255	255
716	1	255	255	255	716	1	255	255	255	716	1	255	255	255
717	1	255	255	255	717	1	255	255	255	717	9	255	255	255
718	2	255	255	255	718	1	255	255	255	718	1	255	255	255
719	34	255	255	255	719	2	255	255	255	719	1	255	255	255
720	1	255	255	255	720	3	255	255	255	720	3	255	255	255
721	3	255	255	255	721	15	255	255	255	721	1	255	255	255
722	259	255	255	255	722	1	255	255	255	722	1	255	255	255
723	2	255	255	255	723	5	255	255	255	723	1	255	255	255
724	387	255	255	255	724	1	255	255	255	724	1	255	255	255
725	2047	255	255	255	725	1	255	255	255	725	1	255	255	255
726	8	255	255	255	726	2	255	255	255	726	1421	255	255	255
727	1	255	255	255	727	1	255	255	255	727	1	255	255	255
728	1	255	255	255	728	2	255	255	255	728	40	255	255	255
729	4	255	255	255	729	1	255	255	255	729	3	255	255	255
730	2	255	255	255	730	3	255	255	255	730	2	255	255	255
731	1	255	255	255	731	2	255	255	255	731	1	255	255	255
732	3	255	255	255	732	16	255	255	255	732	2	255	255	255
733	3	255	255	255	733	1	255	255	255	733	1	255	255	255
734	3	255	255	255	734	8	255	255	255	734	8	255	255	255
735	39	255	255	255	735	1	255	255	255	735	1	255	255	255
736	3	255	255	255	736	1	255	255	255	736	4	255	255	255
737	59	255	255	255	737	1	255	255	255	737	1087	255	255	255
738	1	255	255	255	738	1	255	255	255	738	11031	255	255	255
739	2	255	255	255	739	2	255	255	255	739	1	255	255	255
740	11	255	255	255	740	1	255	255	255	740	1	255	255	255
741	1311	255	255	255	741	5	255	255	255	741	1	255	255	255
742	47	255	255	255	742	1	255	255	255	742	1	255	255	255
743	834	255	255	255	743	1	255	255	255	743	35	255	255	255
744	2	255	255	255	744	1	255	255	255	744	2	255	255	255
745	11	255	255	255	745	1	255	255	255	745	8	255	255	255
746	4	255	255	255	746	1	255	255	255	746	1	255	255	255
747	4	255	255	255	747	1	255	255	255	747	1	255	255	255
748	3	255	255	255	748	1	255	255	255	748	3	255	255	255
749	1	255	255	255	749	1	255	255	255	749	667	255	255	255
750	3	255	255	255	750	960	255	255	255	750	1	255	255	255
751	149	255	255	255	751	2	255	255	255	751	16	255	255	255
752	2	255	255	255	752	2	255	255	255	752	2	255	255	255
753	1	255	255	255	753	38	255	255	255	753	1	255	255	255
754	126	255	255	255	754	1	255	255	255	754	51	255	255	255
755	141	255	255	255	755	3	255	255	255	755	1	255	255	255
756	1	255	255	255	756	1	255	255	255	756	1	255	255	255
757	66	255	255	255	757	2	255	255	255	757	2	255	255	255
758	1	255	255	255	758	1	255	255	255	758	1	255	255	255
759	2	255	255	255	759	1	255	255	255	759	1	255	255	255
760	2	255	255	255	760	2	255	255	255	760	1	255	255	255
761	1	255	255	255	761	5997	255	255	255	761	2	255	255	255
762	68	255	255	255	762	1	255	255	255	762	3	255	255	255
763	5	255	255	255	763	1	255	255	255	763	1	255	255	255
764	2	255	255	255	764	1	255	255	255	764	1	255	255	255
765	1	255	255	255	765	1	255	255	255	765	2	255	255	255
766	1	255	255	255	766	489	255	255	255	766	2668	255	255	255
767	1	255	255	255	767	2	255	255	255	767	6	255	255	255
768	21	255	255	255	768	13	255	255	255	768	759	255	255	255
769	124	255	255	255	769	1	255	255	255	769	1	255	255	255
770	133	255	255	255	770	1	255	255	255	770	1	255	255	255
771	78	255	255	255	771	1	255	255	255	771	1	255	255	255
772	1	255	255	255	772	1	255	255	255	772	1	255	255	255
773	1	255	255	255	773	1	255	255	255	773	1	255	255	255
774	127	255	255	255	774	2	255	255	255	774	3	255	255	255
775	2	255	255	255	775	991	255	255	255	775	1	255	255	255
776	1	255	255	255	776	1	255	255	255	776	1	255	255	255
777	4	255	255	255	777	1	255	255	255	777	284	255	255	255
778	18	255	255	255	778	4	255	255	255	778	35	255	255	255
779	93	255	255	255	779	1	255	255	255	779	1	255	255	255
780	2	255	255	255	780	2	255	255	255	780	1	255	255	255
781	1	255	255	255	781	162	255	255	255	781	2	255	255	255
782	1	255	255	255	782	1	255	255	255	782	1	255	255	255
783	1	255	255	255	783	2	255	255	255	783	1	255	255	255
784	1	255	255	255	784	1	255	255	255	784	1	255	255	255
785	2	255	255	255	785	1	255	255	255	785	4	255	255	255
786	1	255	255	255	786	8	255	255	255	786	1	255	255	255
787	115	255	255	255	787	1	255	255	255	787	1	255	255	255
788	1	255	255	255	788	1	255	255	255	788	34	255	255	255
789	3	255	255	255	789	2	255	255	255	789	3598	255	255	255
790	56	255	255	255	790	14	255	255	255	790	1	255	255	255
791	1	255	255	255	791	83	255	255	255	791	1	255	255	255
792	343	255	255	255	792	852	255	255	255	792	1	255	255	255
793	1	255	255	255	793	1	255	255	255	793	4082	255	255	255
794	1	255	255	255	794	1	255	255	255	794	1	255	255	255
795	19	255	255	255	795	1	255	255	255	795	1	255	255	255
796	1	255	255	255	796	1	255	255	255	796	2	255	255	255
797	1	255	255	255	797	2	255	255	255	797	22	255	255	255
798	1	255	255	255	798	2	255	255	255	798	1	255	255	255
799	284	255	255	255	799	1	255	255	255	799	1	255	255	255
800	125	255	255	255	800	4	255	255	255	800	513	255	255	255
801	60	255	255	255	801	1	255	255	255	801	2	255	255	255
802	21	255	255	255	802	1	255	255	255	802	1	255	255	255
803	1	255	255	255	803	1	255	255	255	803	1	255	255	255
804	62	255	255	255	804	2	255	255	255	804	1	255	255	255
805	105	255	255	255	805	298	255	255	255	805	390	255	255	255
806	47	255	255	255	806	1	255	255	255	806	1	255	255	255
807	122	255	255	255	807	1	255	255	255	807	1	255	255	255
808	48	255	255	255	808	1	255	255	255	808	1	255	255	255
809	43	255	255	255	809	1	255	255	255	809	3	255	255	255

Original data for Figure 2D, Densitometric analysis of Nuclear/Perinuclear B-catenin in control and EoE patient esophageal biopsy tissue.

Values are representative of the mean of a sample size N=18. p<0.05**

810	2	255	255	255	810	1	255	255	255	810	1	255	255	255
811	2	255	255	255	811	1	255	255	255	811	1	255	255	255
812	1	255	255	255	812	1188	255	255	255	812	1	255	255	255
813	1	255	255	255	813	2	255	255	255	813	232	255	255	255
814	2	255	255	255	814	1	255	255	255	814	3	255	255	255
815	142	255	255	255	815	625	255	255	255	815	26	255	255	255
816	85	255	255	255	816	1	255	255	255	816	7	255	255	255
817	29	255	255	255	817	11	255	255	255	817	1	255	255	255
818	1	255	255	255	818	1	255	255	255	818	1	255	255	255
819	1	255	255	255	819	473	255	255	255	819	1	255	255	255
820	14	255	255	255	820	1	255	255	255	820	13	255	255	255
821	1	255	255	255	821	1	255	255	255	821	1	255	255	255
822	2	255	255	255	822	1	255	255	255	822	2	255	255	255
823	1	255	255	255	823	1	255	255	255	823	1	255	255	255
824	1	255	255	255	824	2	255	255	255	824	1	255	255	255
825	54	255	255	255	825	14	255	255	255	825	2	255	255	255
826	1	255	255	255	826	1	255	255	255	826	1	255	255	255
827	235	255	255	255	827	3	255	255	255	827	1	255	255	255
828	172	255	255	255	828	2	255	255	255	828	2	255	255	255
829	1	255	255	255	829	1	255	255	255	829	1	255	255	255
830	114	255	255	255	830	1	255	255	255	830	12	255	255	255
831	40	255	255	255	831	2	255	255	255	831	1	255	255	255
832	1	255	255	255	832	1	255	255	255	832	1304	255	255	255
833	23	255	255	255	833	16	255	255	255	833	86	255	255	255
834	1	255	255	255	834	3	255	255	255	834	1	255	255	255
835	2	255	255	255	835	1	255	255	255	835	1	255	255	255
836	1	255	255	255	836	2	255	255	255	836	1	255	255	255
837	1	255	255	255	837	6	255	255	255	837	1	255	255	255
838	1	255	255	255	838	3	255	255	255	838	1	255	255	255
839	6	255	255	255	839	1	255	255	255	839	1	255	255	255
840	275	255	255	255	840	2	255	255	255	840	14883	255	255	255
841	1	255	255	255	841	1	255	255	255	841	2	255	255	255
842	437	255	255	255	842	2	255	255	255	842	3	255	255	255
843	1	255	255	255	843	4	255	255	255	843	331	255	255	255
844	1	255	255	255	844	1	255	255	255	844	1	255	255	255
845	1	255	255	255	845	1	255	255	255	845	1	255	255	255
846	2	255	255	255	846	1	255	255	255	846	2	255	255	255
847	308	255	255	255	847	6	255	255	255	847	1	255	255	255
848	1	255	255	255	848	4	255	255	255	848	6	255	255	255
849	14	255	255	255	849	106	255	255	255	849	1	255	255	255
850	1	255	255	255	850	2	255	255	255	850	1	255	255	255
851	1633	255	255	255	851	1	255	255	255	851	3	255	255	255
852	3	255	255	255	852	2	255	255	255	852	1	255	255	255
853	3	255	255	255	853	1	255	255	255	853	2	255	255	255
854	1	255	255	255	854	1	255	255	255	854	1	255	255	255
855	2	255	255	255	855	1	255	255	255	855	2	255	255	255
856	1	255	255	255	856	3	255	255	255	856	1	255	255	255
857	151	255	255	255	857	1	255	255	255	857	1	255	255	255
858	2	255	255	255	858	1	255	255	255	858	1	255	255	255
859	2	255	255	255	859	3	255	255	255	859	1	255	255	255
860	42	255	255	255	860	1	255	255	255	860	13	255	255	255
861	2	255	255	255	861	2	255	255	255	861	1	255	255	255
862	2	255	255	255	862	1	255	255	255	862	1	255	255	255
863	1	255	255	255	863	1	255	255	255	863	1	255	255	255
864	29	255	255	255	864	305	255	255	255	864	3	255	255	255
865	1	255	255	255	865	2	255	255	255	865	1	255	255	255
866	51	255	255	255	866	1	255	255	255	866	4	255	255	255
867	200	255	255	255	867	1	255	255	255	867	1	255	255	255
868	4	255	255	255	868	2	255	255	255	868	1	255	255	255
869	2	255	255	255	869	2	255	255	255	869	28	255	255	255
870	86	255	255	255	870	1	255	255	255	870	1	255	255	255
871	91	255	255	255	871	3	255	255	255	871	5	255	255	255
872	1	255	255	255	872	1	255	255	255	872	2	255	255	255
873	3	255	255	255	873	1	255	255	255	873	38	255	255	255
874	3	255	255	255	874	3	255	255	255	874	301	255	255	255
875	2	255	255	255	875	122	255	255	255	875	1	255	255	255
876	2	255	255	255	876	2	255	255	255	876	2	255	255	255
877	2	255	255	255	877	3	255	255	255	877	1	255	255	255
878	1	255	255	255	878	1	255	255	255	878	3	255	255	255
879	1	255	255	255	879	1	255	255	255	879	2	255	255	255
880	1	255	255	255	880	98	255	255	255	880	1	255	255	255
881	30	255	255	255	881	1	255	255	255	881	2	255	255	255
882	399	255	255	255	882	4	255	255	255	882	836	255	255	255
883	2	255	255	255	883	2	255	255	255	883	2	255	255	255
884	21	255	255	255	884	1	255	255	255	884	1	255	255	255
885	1	255	255	255	885	1	255	255	255	885	322	255	255	255
886	1	255	255	255	886	1	255	255	255	886	2	255	255	255
887	1	255	255	255	887	1	255	255	255	887	75	255	255	255
888	1	255	255	255	888	1	255	255	255	888	1	255	255	255
889	1	255	255	255	889	1	255	255	255	889	1	255	255	255
890	207	255	255	255	890	1	255	255	255	890	1	255	255	255
891	2	255	255	255	891	2	255	255	255	891	2	255	255	255
892	3	255	255	255	892	1	255	255	255	892	1	255	255	255
893	1	255	255	255	893	1	255	255	255	893	1	255	255	255
894	2	255	255	255	894	1	255	255	255	894	1	255	255	255
895	2	255	255	255	895	2	255	255	255	895	1	255	255	255
896	39	255	255	255	896	1	255	255	255	896	1	255	255	255
897	12	255	255	255	897	15	255	255	255	897	1	255	255	255
898	47	255	255	255	898	1	255	255	255	898	22	255	255	255
899	1	255	255	255	899	69	255	255	255	899	31	255	255	255
900	6	255	255	255	900	2	255	255	255	900	17	255	255	255
901	13	255	255	255	901	13	255	255	255	901	2	255	255	255
902	1	255	255	255	902	5	255	255	255	902	405	255	255	255
903	1	255	255	255	903	1	255	255	255	903	1	255	255	255
904	71	255	255	255	904	2	255	255	255	904	32	255	255	255
905	1	255	255	255	905	2	255	255	255	905	1	255	255	255
906	3	255	255	255	906	399	255	255	255	906	1	255	255	255
907	2	255	255	255	907	5	255	255	255	907	1	255	255	255
908	2	255	255	255	908	2	255	255	255	908	406	255	255	255
909	1	255	255	255	909	1	255	255	255	909	1	255	255	255
910	2	255	255	255	910	1	255	255	255	910	9	255	255	255
911	4	255	255	255	911	952	255	255	255	911	2	255	255	255
912	1	255	255	255	912	1	255	255	255	912	1	255	255	255
913	79	255	255	255	913	1	255	255	255	913	11	255	255	255
914	1	255	255	255	914	3	255	255	255	914	236	255	255	255
915	1	255	255	255	915	2	255	255	255	915	11	255	255	255
916	1	255	255	255	916	2	255	255	255	916	2	255	255	255
917	2	255	255	255	917	1	255	255	255	917	1	255	255	255
918	136	255	255	255	918	1	255	255	255	918	449	255	255	255
919	2	255	255	255	919	1	255	255	255	919	1	255	255	255
920	1	255	255	255	920	1	255	255	255	920	1	255	255	255
921	1	255	255	255	921	201	255	255	255	921	1	255	255	255
922	352	255	255	255	922	1	255	255	255	922	37	255	255	255
923	1	255	255	255	923	1	255	255	255	923	2	255	255	255
924	3	255	255	255	924	1	255	255	255	924	5	255	255	255
925	1	255	255	255	925	1	255	255	255	925	3	255	255	255
926	1	255	255	255	926	5	255	255	255	926	403	255	255	255
927	1	255	255	255	927	1	255	255	255	927	1	255	255	255
928	2	255	255	255	928	2	255	255	255	928	1	255	255	255
929	19	255	255	255	929	1	255	255	255	929	1	255	255	255
930	20	255	255	255	930	1	255	255	255	930	1	255	255	255
931	1242	255	255	255	931	2704	255	255	255	931	1	255	255	255
932	1	255	255	255	932	1	255	255	255	932	1	255	255	255
933	2	255	255	255	933	4	255	255	255	933	6	255	255	255
934	1	255	255	255	934	1	255	255	255	934	1	255	255	255
935	4	255	255	255	935	1	255	255	255	935	1	255	255	255
936	1	255	255	255	936	1	255	255	255	936	8188	255	255	255
937	1	255	255	255	937	1	255	255	255	937	1	255	255	255
938	350	255	255	255	938	1	255	255	255	938	434	255	255	255
939	1	255	255	255	939	2	255	255	255	939	1	255	255	255
940	2	255	255	255	940	1	255	255	255	940	2	255	255	255
941	1	255	255	255	941	2	255	255	255	941	105	255	255	255
942	2	255	255	255	942	1	255	255	255	942	3	255	255	255
943	4	255	255	255	943	1	255	255	255	943	1	255	255	255
944	15	255	255	255	944	1	255	255	255	944	1	255	255	255
945	82	255	255	255	945	1	255	255	255	945	2	255	255	255
946	1	255	255	255	946	1	255	255	255	946	1	255	255	255
947	1	255	255	255	947	1	255	255	255	947	2	255	255	255

Original data for Figure 2D, Densitometric analysis of Nuclear/Perinuclear B-catenin in control and EoE patient esophageal biopsy tissue.

Values are representative of the mean of a sample size N=18. p<0.05**

948	1	255	255	255	948	7	255	255	255	948	2	255	255	255
949	1	255	255	255	949	61	255	255	255	949	1	255	255	255
950	3	255	255	255	950	1	255	255	255	950	2534	255	255	255
951	22	255	255	255	951	3	255	255	255	951	1	255	255	255
952	43	255	255	255	952	1	255	255	255	952	2	255	255	255
953	198	255	255	255	953	2	255	255	255	953	1	255	255	255
954	1	255	255	255	954	1	255	255	255	954	187	255	255	255
955	1	255	255	255	955	1	255	255	255	955	1	255	255	255
956	1	255	255	255	956	1	255	255	255	956	7	255	255	255
957	1	255	255	255	957	1	255	255	255	957	805	255	255	255
958	241	255	255	255	958	331	255	255	255	958	5	255	255	255
959	43	255	255	255	959	1	255	255	255	959	1	255	255	255
960	222	255	255	255	960	1	255	255	255	960	1	255	255	255
961	2	255	255	255	961	1	255	255	255	961	1	255	255	255
962	1	255	255	255	962	1	255	255	255	962	61	255	255	255
963	3	255	255	255	963	3	255	255	255	963	1	255	255	255
964	65	255	255	255	964	2	255	255	255	964	1	255	255	255
965	40	255	255	255	965	3	255	255	255	965	286	255	255	255
966	335	255	255	255	966	1063	255	255	255	966	2	255	255	255
967	1	255	255	255	967	614	255	255	255	967	1	255	255	255
968	389	255	255	255	968	1	255	255	255	968	1	255	255	255
969	1	255	255	255	969	2	255	255	255	969	1	255	255	255
970	4	255	255	255	970	1	255	255	255	970	3	255	255	255
971	125	255	255	255	971	2	255	255	255	971	1	255	255	255
972	1	255	255	255	972	1	255	255	255	972	6	255	255	255
973	2	255	255	255	973	1	255	255	255	973	3	255	255	255
974	2	255	255	255	974	2	255	255	255	974	3	255	255	255
975	3	255	255	255	975	1	255	255	255	975	7	255	255	255
976	3	255	255	255	976	1	255	255	255	976	3	255	255	255
977	1	255	255	255	977	1	255	255	255	977	1	255	255	255
978	587	255	255	255	978	1	255	255	255	978	1	255	255	255
979	1	255	255	255	979	7	255	255	255	979	2	255	255	255
980	1	255	255	255	980	2	255	255	255	980	8	255	255	255
981	2	255	255	255	981	1	255	255	255	981	1	255	255	255
982	3	255	255	255	982	5	255	255	255	982	2	255	255	255
983	114	255	255	255	983	586	255	255	255	983	1	255	255	255
984	2	255	255	255	984	1	255	255	255	984	18	255	255	255
985	285	255	255	255	985	1	255	255	255	985	1	255	255	255
986	1	255	255	255	986	15	255	255	255	986	1	255	255	255
987	2	255	255	255	987	1	255	255	255	987	2	255	255	255
988	191	255	255	255	988	1	255	255	255	988	4	255	255	255
989	4	255	255	255	989	2	255	255	255	989	1	255	255	255
990	1	255	255	255	990	1	255	255	255	990	1	255	255	255
991	8	255	255	255	991	4	255	255	255	991	43	255	255	255
992	3	255	255	255	992	2	255	255	255	992	1	255	255	255
993	5	255	255	255	993	1	255	255	255	993	1012	255	255	255
994	5	255	255	255	994	1	255	255	255	994	485	255	255	255
995	3	255	255	255	995	1	255	255	255	995	1	255	255	255
996	1	255	255	255	996	1	255	255	255	996	201	255	255	255
997	2	255	255	255	997	1	255	255	255	997	2	255	255	255
998	26	255	255	255	998	26	255	255	255	998	1	255	255	255
999	173	255	255	255	999	1	255	255	255	999	1	255	255	255
1000	1	255	255	255	1000	1	255	255	255	1000	1	255	255	255
1001	6	255	255	255	1001	1	255	255	255	1001	1	255	255	255
1002	30	255	255	255	1002	1	255	255	255	1002	2	255	255	255
1003	98	255	255	255	1003	1	255	255	255	1003	5	255	255	255
1004	1	255	255	255	1004	1	255	255	255	1004	1	255	255	255
1005	17	255	255	255	1005	1	255	255	255	1005	1	255	255	255
1006	1	255	255	255	1006	1	255	255	255	1006	1	255	255	255
1007	1	255	255	255	1007	1	255	255	255	1007	5	255	255	255
1008	102	255	255	255	1008	1	255	255	255	1008	461	255	255	255
1009	1	255	255	255	1009	291	255	255	255	1009	220	255	255	255
1010	1	255	255	255	1010	17	255	255	255	1010	63	255	255	255
1011	1	255	255	255	1011	1	255	255	255	1011	1	255	255	255
1012	1	255	255	255	1012	1	255	255	255	1012	1	255	255	255
1013	365	255	255	255	1013	1	255	255	255	1013	2	255	255	255
1014	1	255	255	255	1014	1	255	255	255	1014	1	255	255	255
1015	5	255	255	255	1015	3	255	255	255	1015	1	255	255	255
1016	1	255	255	255	1016	10	255	255	255	1016	1	255	255	255
1017	1	255	255	255	1017	1	255	255	255	1017	5	255	255	255
1018	2	255	255	255	1018	3	255	255	255	1018	1	255	255	255
1019	1	255	255	255	1019	1	255	255	255	1019	2	255	255	255
1020	3	255	255	255	1020	2	255	255	255	1020	1	255	255	255
1021	1	255	255	255	1021	143	255	255	255	1021	3	255	255	255
1022	85	255	255	255	1022	2	255	255	255	1022	1	255	255	255
1023	2	255	255	255	1023	1	255	255	255	1023	3	255	255	255
1024	144	255	255	255	1024	1	255	255	255	1024	13	255	255	255
1025	4	255	255	255	1025	2012	255	255	255	1025	1	255	255	255
1026	1	255	255	255	1026	1	255	255	255	1026	10	255	255	255
1027	1	255	255	255	1027	4	255	255	255	1027	436	255	255	255
1028	193	255	255	255	1028	4	255	255	255	1028	2	255	255	255
1029	1	255	255	255	1029	1	255	255	255	1029	1	255	255	255
1030	4	255	255	255	1030	1	255	255	255	1030	2	255	255	255
1031	1	255	255	255	1031	2	255	255	255	1031	1	255	255	255
1032	471	255	255	255	1032	1	255	255	255	1032	1	255	255	255
1033	1	255	255	255	1033	1	255	255	255	1033	12241	255	255	255
1034	4	255	255	255	1034	1	255	255	255	1034	1	255	255	255
1035	2	255	255	255	1035	1	255	255	255	1035	1	255	255	255
1036	7	255	255	255	1036	117	255	255	255	1036	544	255	255	255
1037	1553	255	255	255	1037	1	255	255	255	1037	3	255	255	255
1038	1	255	255	255	1038	2	255	255	255	1038	1	255	255	255
1039	55	255	255	255	1039	1714	255	255	255	1039	43	255	255	255
1040	61	255	255	255	1040	2	255	255	255	1040	1	255	255	255
1041	1	255	255	255	1041	2	255	255	255	1041	100	255	255	255
1042	318	255	255	255	1042	1	255	255	255	1042	1	255	255	255
1043	1	255	255	255	1043	2	255	255	255	1043	1	255	255	255
1044	1	255	255	255	1044	3	255	255	255	1044	2	255	255	255
1045	90	255	255	255	1045	1	255	255	255	1045	5	255	255	255
1046	1	255	255	255	1046	2	255	255	255	1046	1	255	255	255
1047	1	255	255	255	1047	2	255	255	255	1047	1	255	255	255
1048	10	255	255	255	1048	2	255	255	255	1048	3	255	255	255
1049	1	255	255	255	1049	1	255	255	255	1049	1	255	255	255
1050	1	255	255	255	1050	11	255	255	255	1050	1	255	255	255
1051	6	255	255	255	1051	1	255	255	255	1051	1	255	255	255
1052	170	255	255	255	1052	2852	255	255	255	1052	2	255	255	255
1053	546	255	255	255	1053	1	255	255	255	1053	2	255	255	255
1054	6	255	255	255	1054	1	255	255	255	1054	1	255	255	255
1055	2	255	255	255	1055	1	255	255	255	1055	1	255	255	255
1056	1	255	255	255	1056	170	255	255	255	1056	5	255	255	255
1057	1	255	255	255	1057	1	255	255	255	1057	1	255	255	255
1058	1	255	255	255	1058	1	255	255	255	1058	1	255	255	255
1059	2	255	255	255	1059	1	255	255	255	1059	2	255	255	255
1060	1	255	255	255	1060	3	255	255	255	1060	100	255	255	255
1061	1	255	255	255	1061	4	255	255	255	1061	15	255	255	255
1062	1	255	255	255	1062	1	255	255	255	1062	1	255	255	255
1063	71	255	255	255	1063	1	255	255	255	1063	1	255	255	255
1064	816	255	255	255	1064	1	255	255	255	1064	43	255	255	255
1065	3	255	255	255	1065	2	255	255	255	1065	1	255	255	255
1066	130	255	255	255	1066	68	255	255	255	1066	376	255	255	255
1067	1	255	255	255	1067	3	255	255	255	1067	418	255	255	255
1068	1	255	255	255	1068	1	255	255	255	1068	1	255	255	255
1069	242	255	255	255	1069	1	255	255	255	1069	8	255	255	255
1070	1	255	255	255	1070	1	255	255	255	1070	2	255	255	255
1071	887	255	255	255	1071	2	255	255	255	1071	1	255	255	255
1072	35	255	255	255	1072	1	255	255	255	1072	1	255	255	255
1073	2	255	255	255	1073	1	255	255	255	1073	1	255	255	255
1074	1	255	255	255	1074	2	255	255	255	1074	1	255	255	255
1075	175	255	255	255	1075	1	255	255	255	1075	3	255	255	255
1076	2	255	255	255	1076	2	255	255	255	1076	2	255	255	255
1077	72	255	255	255	1077	4	255	255	255	1077	1	255	255	255
1078	5	255	255	255	1078	1	255	255	255	1078	5	255	255	255
1079	200	255	255	255	1079	2	255	255	255	1079	1	255	255	255
1080	306	255	255	255	1080	1	255	255	255	1080	1	255	255	255
1081	45	255	255	255	1081	6	255	255	255	1081	731	255	255	255
1082	1	255	255	255	1082	1	255	255	255	1082	1	255	255	255
1083	18	255	255	255	1083	1	255	255	255	1083	2	255	255	255
1084	1	255	255	255	1084	3	255	255	255	1084	4	255	255	255
1085	2	255	255	255	1085	1	255	255	255	1085	1	255	255	255

Original data for Figure 2D, Densitometric analysis of Nuclear/Perinuclear B-catenin in control and EoE patient esophageal biopsy tissue.

Values are representative of the mean of a sample size N=18. p<0.05**

1086	1	255	255	255	1086	1	255	255	255	1086	1	255	255	255
1087	1	255	255	255	1087	1	255	255	255	1087	2	255	255	255
1088	2	255	255	255	1088	1	255	255	255	1088	1	255	255	255
1089	2	255	255	255	1089	1	255	255	255	1089	628	255	255	255
1090	201	255	255	255	1090	2	255	255	255	1090	1	255	255	255
1091	1	255	255	255	1091	3	255	255	255	1091	4	255	255	255
1092	1	255	255	255	1092	1	255	255	255	1092	2	255	255	255
1093	149	255	255	255	1093	1	255	255	255	1093	696	255	255	255
1094	203	255	255	255	1094	1	255	255	255	1094	1	255	255	255
1095	166	255	255	255	1095	1	255	255	255	1095	1	255	255	255
1096	1	255	255	255	1096	1	255	255	255	1096	1	255	255	255
1097	3	255	255	255	1097	1	255	255	255	1097	1	255	255	255
1098	87	255	255	255	1098	1	255	255	255	1098	1	255	255	255
1099	2	255	255	255	1099	3	255	255	255	1099	1	255	255	255
1100	3	255	255	255	1100	1	255	255	255	1100	1	255	255	255
1101	1	255	255	255	1101	1	255	255	255	1101	6	255	255	255
1102	1	255	255	255	1102	1	255	255	255	1102	1	255	255	255
1103	1	255	255	255	1103	1	255	255	255	1103	1	255	255	255
1104	1	255	255	255	1104	1	255	255	255	1104	1	255	255	255
1105	1	255	255	255	1105	4	255	255	255	1105	95	255	255	255
1106	2	255	255	255	1106	1	255	255	255	1106	84	255	255	255
1107	1	255	255	255	1107	1	255	255	255	1107	16	255	255	255
1108	2	255	255	255	1108	258	255	255	255	1108	1	255	255	255
1109	10	255	255	255	1109	1	255	255	255	1109	2	255	255	255
1110	1	255	255	255	1110	1	255	255	255	1110	1	255	255	255
1111	1	255	255	255	1111	5	255	255	255	1111	1	255	255	255
1112	2	255	255	255	1112	2	255	255	255	1112	1	255	255	255
1113	1	255	255	255	1113	2	255	255	255	1113	2	255	255	255
1114	291	255	255	255	1114	2	255	255	255	1114	1	255	255	255
1115	1	255	255	255	1115	4	255	255	255	1115	1	255	255	255
1116	1	255	255	255	1116	1	255	255	255	1116	2	255	255	255
1117	1	255	255	255	1117	1	255	255	255	1117	1	255	255	255
1118	1	255	255	255	1118	1	255	255	255	1118	2	255	255	255
1119	2	255	255	255	1119	1	255	255	255	1119	1	255	255	255
1120	157	255	255	255	1120	1	255	255	255	1120	1	255	255	255
1121	94	255	255	255	1121	3357	255	255	255	1121	1	255	255	255
1122	3	255	255	255	1122	1	255	255	255	1122	8	255	255	255
1123	1	255	255	255	1123	2	255	255	255	1123	1	255	255	255
1124	173	255	255	255	1124	1	255	255	255	1124	3	255	255	255
1125	122	255	255	255	1125	1	255	255	255	1125	1	255	255	255
1126	4	255	255	255	1126	139	255	255	255	1126	1	255	255	255
1127	952	255	255	255	1127	1	255	255	255	1127	5	255	255	255
1128	1	255	255	255	1128	1	255	255	255	1128	1	255	255	255
1129	4	255	255	255	1129	1	255	255	255	1129	1	255	255	255
1130	1	255	255	255	1130	2	255	255	255	1130	9	255	255	255
1131	1	255	255	255	1131	1	255	255	255	1131	1	255	255	255
1132	325	255	255	255	1132	661	255	255	255	1132	391	255	255	255
1133	33	255	255	255	1133	2	255	255	255	1133	1	255	255	255
1134	4	255	255	255	1134	3	255	255	255	1134	1	255	255	255
1135	2	255	255	255	1135	1	255	255	255	1135	1	255	255	255
1136	7	255	255	255	1136	1	255	255	255	1136	1	255	255	255
1137	2	255	255	255	1137	1	255	255	255	1137	1	255	255	255
1138	1	255	255	255	1138	1	255	255	255	1138	1	255	255	255
1139	19	255	255	255	1139	1	255	255	255	1139	1	255	255	255
1140	1	255	255	255	1140	1	255	255	255	1140	2	255	255	255
1141	271	255	255	255	1141	3	255	255	255	1141	2	255	255	255
1142	43	255	255	255	1142	1	255	255	255	1142	1	255	255	255
1143	2	255	255	255	1143	89	255	255	255	1143	1	255	255	255
1144	550	255	255	255	1144	1	255	255	255	1144	1	255	255	255
1145	2	255	255	255	1145	3	255	255	255	1145	1	255	255	255
1146	1	255	255	255	1146	4	255	255	255	1146	2	255	255	255
1147	2	255	255	255	1147	25	255	255	255	1147	28	255	255	255
1148	1	255	255	255	1148	9	255	255	255	1148	1	255	255	255
1149	2	255	255	255	1149	2	255	255	255	1149	146	255	255	255
1150	95	255	255	255	1150	6	255	255	255	1150	2	255	255	255
1151	1	255	255	255	1151	278	255	255	255	1151	134	255	255	255
1152	3	255	255	255	1152	2	255	255	255	1152	1	255	255	255
1153	1	255	255	255	1153	63	255	255	255	1153	1	255	255	255
1154	2	255	255	255	1154	1	255	255	255	1154	1	255	255	255
1155	1	255	255	255	1155	3	255	255	255	1155	1	255	255	255
1156	1	255	255	255	1156	1	255	255	255	1156	4	255	255	255
1157	1	255	255	255	1157	1	255	255	255	1157	1	255	255	255
1158	1	255	255	255	1158	3	255	255	255	1158	2	255	255	255
1159	1	255	255	255	1159	5	255	255	255	1159	2	255	255	255
1160	2	255	255	255	1160	1	255	255	255	1160	1	255	255	255
1161	1	255	255	255	1161	1	255	255	255	1161	1	255	255	255
1162	21	255	255	255	1162	1	255	255	255	1162	1	255	255	255
1163	2	255	255	255	1163	2	255	255	255	1163	1609	255	255	255
1164	2	255	255	255	1164	4	255	255	255	1164	1	255	255	255
1165	2	255	255	255	1165	3	255	255	255	1165	2	255	255	255
1166	8	255	255	255	1166	1	255	255	255	1166	2	255	255	255
1167	6	255	255	255	1167	1	255	255	255	1167	1	255	255	255
1168	1	255	255	255	1168	100	255	255	255	1168	1	255	255	255
1169	1	255	255	255	1169	1662	255	255	255	1169	1	255	255	255
1170	2	255	255	255	1170	1	255	255	255	1170	2	255	255	255
1171	29	255	255	255	1171	3	255	255	255	1171	1	255	255	255
1172	1	255	255	255	1172	1	255	255	255	1172	1	255	255	255
1173	28	255	255	255	1173	2	255	255	255	1173	1	255	255	255
1174	1	255	255	255	1174	1	255	255	255	1174	1	255	255	255
1175	1	255	255	255	1175	1	255	255	255	1175	29	255	255	255
1176	15	255	255	255	1176	1	255	255	255	1176	2	255	255	255
1177	209	255	255	255	1177	2	255	255	255	1177	1	255	255	255
1178	1	255	255	255	1178	1	255	255	255	1178	1	255	255	255
1179	1	255	255	255	1179	1796	255	255	255	1179	784	255	255	255
1180	137	255	255	255	1180	8	255	255	255	1180	1	255	255	255
1181	11	255	255	255	1181	1	255	255	255	1181	2	255	255	255
1182	21	255	255	255	1182	1	255	255	255	1182	1	255	255	255
1183	2	255	255	255	1183	1	255	255	255	1183	8	255	255	255
1184	1	255	255	255	1184	3	255	255	255	1184	12	255	255	255
1185	1	255	255	255	1185	3	255	255	255	1185	5	255	255	255
1186	3	255	255	255	1186	15	255	255	255	1186	29	255	255	255
1187	3	255	255	255	1187	1	255	255	255	1187	1	255	255	255
1188	1506	255	255	255	1188	1	255	255	255	1188	1	255	255	255
1189	71	255	255	255	1189	2	255	255	255	1189	1130	255	255	255
1190	80	255	255	255	1190	1	255	255	255	1190	3	255	255	255
1191	1	255	255	255	1191	1	255	255	255	1191	4	255	255	255
1192	4	255	255	255	1192	2	255	255	255	1192	3	255	255	255
1193	1	255	255	255	1193	14	255	255	255	1193	1	255	255	255
1194	5	255	255	255	1194	1	255	255	255	1194	2	255	255	255
1195	1	255	255	255	1195	1	255	255	255	1195	4	255	255	255
1196	2	255	255	255	1196	6	255	255	255	1196	261	255	255	255
1197	1	255	255	255	1197	76	255	255	255	1197	2	255	255	255
1198	2	255	255	255	1198	2	255	255	255	1198	1	255	255	255
1199	4	255	255	255	1199	28	255	255	255	1199	1	255	255	255
1200	3	255	255	255	1200	1	255	255	255	1200	2	255	255	255
1201	1	255	255	255	1201	1	255	255	255	1201	1894	255	255	255
1202	3	255	255	255	1202	3	255	255	255	1202	1	255	255	255
1203	1	255	255	255	1203	1	255	255	255	1203	2	255	255	255
1204	1	255	255	255	1204	2	255	255	255	1204	2	255	255	255
1205	1	255	255	255	1205	1	255	255	255	1205	1	255	255	255
1206	13	255	255	255	1206	1	255	255	255	1206	12	255	255	255
1207	1	255	255	255	1207	1	255	255	255	1207	2	255	255	255
1208	2	255	255	255	1208	27	255	255	255	1208	1	255	255	255
1209	1	255	255	255	1209	1	255	255	255	1209	2	255	255	255
1210	102	255	255	255	1210	2	255	255	255	1210	1	255	255	255
1211	383	255	255	255	1211	1	255	255	255	1211	1	255	255	255
1212	6	255	255	255	1212	1	255	255	255	1212	1	255	255	255
1213	2	255	255	255	1213	1	255	255	255	1213	3	255	255	255
1214	2	255	255	255	1214	1	255	255	255	1214	15918	255	255	255
1215	1	255	255	255	1215	1	255	255	255	1215	1	255	255	255
1216	1	255	255	255	1216	23	255	255	255	1216	1	255	255	255
1217	110	255	255	255	1217	3	255	255	255	1217	1	255	255	255
1218	2	255	255	255	1218	16	255	255	255	1218	1	255	255	255
1219	332	255	255	255	1219	2	255	255	255	1219	743	255	255	255
1220	4	255	255	255	1220	1	255	255	255	1220	1	255	255	255
1221	1	255	255	255	1221	1	255	255	255	1221	272	255	255	255
1222	1	255	255	255	1222	1	255	255	255	1222	1	255	255	255
1223	9	255	255	255	1223	1	255	255	255	1223	208	255	255	255

Original data for Figure 2D, Densitometric analysis of Nuclear/Perinuclear B-catenin in control and EoE patient esophageal biopsy tissue.
Values are representative of the mean of a sample size N=18. p<0.05**

1224	1	255	255	255	1224	2	255	255	255	1224	1	255	255	255
1225	1	255	255	255	1225	1	255	255	255	1225	6	255	255	255
1226	1	255	255	255	1226	192	255	255	255	1226	94	255	255	255
1227	38	255	255	255	1227	1	255	255	255	1227	1	255	255	255
1228	3	255	255	255	1228	1	255	255	255	1228	2	255	255	255
1229	288	255	255	255	1229	1	255	255	255	1229	332	255	255	255
1230	3	255	255	255	1230	1	255	255	255	1230	1	255	255	255
1231	3	255	255	255	1231	9	255	255	255	1231	73	255	255	255
1232	1400	255	255	255	1232	1	255	255	255	1232	77	255	255	255
1233	29	255	255	255	1233	1	255	255	255	1233	163	255	255	255
1234	9	255	255	255	1234	1	255	255	255	1234	1	255	255	255
1235	2	255	255	255	1235	1	255	255	255	1235	51	255	255	255
1236	1	255	255	255	1236	2	255	255	255	1236	1	255	255	255
1237	1	255	255	255	1237	2	255	255	255	1237	2	255	255	255
1238	73	255	255	255	1238	1	255	255	255	1238	1	255	255	255
1239	1	255	255	255	1239	1	255	255	255	1239	2	255	255	255
1240	7	255	255	255	1240	3	255	255	255	1240	5	255	255	255
1241	7	255	255	255	1241	1	255	255	255	1241	2	255	255	255
1242	1	255	255	255	1242	2	255	255	255	1242	1	255	255	255
1243	1	255	255	255	1243	7	255	255	255	1243	1	255	255	255
1244	526	255	255	255	1244	2	255	255	255	1244	51	255	255	255
1245	299	255	255	255	1245	1	255	255	255	1245	1	255	255	255
1246	93	255	255	255	1246	3	255	255	255	1246	5	255	255	255
1247	4	255	255	255	1247	1	255	255	255	1247	2	255	255	255
1248	1	255	255	255	1248	3	255	255	255	1248	1	255	255	255
1249	1	255	255	255	1249	1	255	255	255	1249	1	255	255	255
1250	1	255	255	255	1250	750	255	255	255	1250	1	255	255	255
1251	1	255	255	255	1251	1	255	255	255	1251	1	255	255	255
1252	1	255	255	255	1252	2	255	255	255	1252	569	255	255	255
1253	7	255	255	255	1253	1	255	255	255	1253	1	255	255	255
1254	1	255	255	255	1254	1	255	255	255	1254	112	255	255	255
1255	2	255	255	255	1255	1	255	255	255	1255	87	255	255	255
1256	563	255	255	255	1256	3	255	255	255	1256	98	255	255	255
1257	80	255	255	255	1257	129	255	255	255	1257	1	255	255	255
1258	75	255	255	255	1258	1	255	255	255	1258	196	255	255	255
1259	7	255	255	255	1259	1	255	255	255	1259	558	255	255	255
1260	1	255	255	255	1260	2	255	255	255	1260	1	255	255	255
1261	1	255	255	255	1261	1	255	255	255	1261	3	255	255	255
1262	9	255	255	255	1262	2	255	255	255	1262	281	255	255	255
1263	2	255	255	255	1263	11	255	255	255	1263	1	255	255	255
1264	156	255	255	255	1264	1	255	255	255	1264	1	255	255	255
1265	1	255	255	255	1265	1	255	255	255	1265	1	255	255	255
1266	1	255	255	255	1266	6	255	255	255	1266	43	255	255	255
1267	11	255	255	255	1267	1	255	255	255	1267	1	255	255	255
1268	1	255	255	255	1268	2	255	255	255	1268	58	255	255	255
1269	1	255	255	255	1269	1	255	255	255	1269	1	255	255	255
1270	213	255	255	255	1270	2	255	255	255	1270	1	255	255	255
1271	51	255	255	255	1271	1	255	255	255	1271	2	255	255	255
1272	52	255	255	255	1272	1	255	255	255	1272	171	255	255	255
1273	2	255	255	255	1273	2	255	255	255	1273	67	255	255	255
1274	31	255	255	255	1274	2	255	255	255	1274	1	255	255	255
1275	92	255	255	255	1275	2	255	255	255	1275	4708	255	255	255
1276	2	255	255	255	1276	3	255	255	255	1276	1	255	255	255
1277	1	255	255	255	1277	171	255	255	255	1277	432	255	255	255
1278	4	255	255	255	1278	2	255	255	255	1278	2	255	255	255
1279	108	255	255	255	1279	1	255	255	255	1279	1	255	255	255
1280	2	255	255	255	1280	1	255	255	255	1280	2	255	255	255
1281	3	255	255	255	1281	4	255	255	255	1281	1	255	255	255
1282	3	255	255	255	1282	3	255	255	255	1282	1	255	255	255
1283	2	255	255	255	1283	4	255	255	255	1283	218	255	255	255
1284	23	255	255	255	1284	71	255	255	255	1284	1	255	255	255
1285	92	255	255	255	1285	1	255	255	255	1285	1104	255	255	255
1286	3478	255	255	255	1286	1	255	255	255	1286	9282	255	255	255
1287	2	255	255	255	1287	1	255	255	255	1287	3	255	255	255
1288	2	255	255	255	1288	19	255	255	255	1288	374	255	255	255
1289	15	255	255	255	1289	1	255	255	255	1289	29	255	255	255
1290	2	255	255	255	1290	6	255	255	255	1290	1	255	255	255
1291	4	255	255	255	1291	2	255	255	255	1291	2	255	255	255
1292	2	255	255	255	1292	1	255	255	255	1292	175	255	255	255
1293	3	255	255	255	1293	1	255	255	255	1293	1	255	255	255
1294	1	255	255	255	1294	1	255	255	255	1294	1	255	255	255
1295	143	255	255	255	1295	1	255	255	255	1295	1	255	255	255
1296	24	255	255	255	1296	2	255	255	255	1296	1	255	255	255
1297	1	255	255	255	1297	1	255	255	255	1297	1	255	255	255
1298	1	255	255	255	1298	10520	255	255	255	1298	1	255	255	255
1299	38	255	255	255	1299	1	255	255	255	1299	1	255	255	255
1300	2	255	255	255	1300	238	255	255	255	1300	1	255	255	255
1301	2	255	255	255	1301	1	255	255	255	1301	827	255	255	255
1302	17	255	255	255	1302	1	255	255	255	1302	1	255	255	255
1303	125	255	255	255	1303	1	255	255	255	1303	1	255	255	255
1304	1	255	255	255	1304	1	255	255	255	1304	4	255	255	255
1305	1	255	255	255	1305	1	255	255	255	1305	6168	255	255	255
1306	1	255	255	255	1306	1	255	255	255	1306	1	255	255	255
1307	1	255	255	255	1307	1	255	255	255	1307	82	255	255	255
1308	26	255	255	255	1308	3	255	255	255	1308	1	255	255	255
1309	3	255	255	255	1309	2	255	255	255	1309	4	255	255	255
1310	137	255	255	255	1310	1	255	255	255	1310	4	255	255	255
1311	1	255	255	255	1311	3	255	255	255	1311	1	255	255	255
1312	2	255	255	255	1312	2	255	255	255	1312	1	255	255	255
1313	113	255	255	255	1313	1	255	255	255	1313	1	255	255	255
1314	1	255	255	255	1314	1	255	255	255	1314	1	255	255	255
1315	1	255	255	255	1315	1	255	255	255	1315	1	255	255	255
1316	416	255	255	255	1316	1	255	255	255	1316	1	255	255	255
1317	1	255	255	255	1317	1	255	255	255	1317	1	255	255	255
1318	22	255	255	255	1318	1	255	255	255	1318	6	255	255	255
1319	135	255	255	255	1319	1	255	255	255	1319	103	255	255	255
1320	71	255	255	255	1320	1	255	255	255	1320	92	255	255	255
1321	231	255	255	255	1321	73	255	255	255	1321	9	255	255	255
1322	1	255	255	255	1322	1	255	255	255	1322	1	255	255	255
1323	1	255	255	255	1323	3005	255	255	255	1323	2	255	255	255
1324	1	255	255	255	1324	3	255	255	255	1324	26	255	255	255
1325	2	255	255	255	1325	15	255	255	255	1325	1	255	255	255
1326	29	255	255	255	1326	1	255	255	255	1326	2	255	255	255
1327	1	255	255	255	1327	1	255	255	255	1327	570	255	255	255
1328	62	255	255	255	1328	1	255	255	255	1328	1	255	255	255
1329	311	255	255	255	1329	1	255	255	255	1329	2	255	255	255
1330	1	255	255	255	1330	53	255	255	255	1330	3	255	255	255
1331	1	255	255	255	1331	2	255	255	255	1331	1	255	255	255
1332	2	255	255	255	1332	2	255	255	255	1332	234	255	255	255
1333	6140	255	255	255	1333	1	255	255	255	1333	17	255	255	255
1334	1	255	255	255	1334	1	255	255	255	1334	3	255	255	255
1335	5	255	255	255	1335	1051	255	255	255	1335	3	255	255	255
1336	1	255	255	255	1336	1	255	255	255	1336	2	255	255	255
1337	65	255	255	255	1337	1	255	255	255	1337	1	255	255	255
1338	1	255	255	255	1338	3	255	255	255	1338	3	255	255	255
1339	2	255	255	255	1339	2	255	255	255	1339	1	255	255	255
1340	1	255	255	255	1340	1	255	255	255	1340	10	255	255	255
1341	5	255	255	255	1341	1	255	255	255	1341	1	255	255	255
1342	1	255	255	255	1342	1	255	255	255	1342	15	255	255	255
1343	1	255	255	255	1343	1	255	255	255	1343	7	255	255	255
1344	1	255	255	255	1344	1	255	255	255	1344	2	255	255	255
1345	2	255	255	255	1345	1	255	255	255	1345	3	255	255	255
1346	1	255	255	255	1346	1	255	255	255	1346	219	255	255	255
1347	7	255	255	255	1347	2	255	255	255	1347	15	255	255	255
1348	1	255	255	255	1348	1	255	255	255	1348	9	255	255	255
1349	6	255	255	255	1349	1	255	255	255	1349	2	255	255	255
1350	70	255	255	255	1350	1	255	255	255	1350	112	255	255	255
1351	1	255	255	255	1351	5	255	255	255	1351	1	255	255	255
1352	3	255	255	255	1352	1	255	255	255	1352	5	255	255	255
1353	3	255	255	255	1353	1	255	255	255	1353	1	255	255	255
1354	4	255	255	255	1354	1	255	255	255	1354	7	255	255	255
1355	1	255	255	255	1355	8	255	255	255	1355	138	255	255	255
1356	286	255	255	255	1356	1	255	255	255	1356	1	255	255	255
1357	2	255	255	255	1357	1	255	255	255	1357	3	255	255	255
1358	1	255	255	255	1358	2	255	255	255	1358	34	255	255	255
1359	1	255	255	255	1359	2	255	255	255	1359	567	255	255	255
1360	1	255	255	255	1360	2	255	255	255	1360	19	255	255	255
1361	2	255	255	255	1361	2	255	255	255	1361	3	255	255	255

Original data for Figure 2D, Densitometric analysis of Nuclear/Perinuclear B-catenin in control and EoE patient esophageal biopsy tissue.
Values are representative of the mean of a sample size N=18. p<0.05**

1362	1	255	255	255	1362	79	255	255	255	1362	1	255	255	255
1363	4	255	255	255	1363	2	255	255	255	1363	7	255	255	255
1364	1	255	255	255	1364	1	255	255	255	1364	3	255	255	255
1365	1	255	255	255	1365	6	255	255	255	1365	1	255	255	255
1366	1	255	255	255	1366	1	255	255	255	1366	1	255	255	255
1367	3	255	255	255	1367	1	255	255	255	1367	1	255	255	255
1368	30	255	255	255	1368	1	255	255	255	1368	1053	255	255	255
1369	1	255	255	255	1369	1	255	255	255	1369	115	255	255	255
1370	1	255	255	255	1370	112	255	255	255	1370	1	255	255	255
1371	352	255	255	255	1371	1	255	255	255	1371	2	255	255	255
1372	2	255	255	255	1372	2	255	255	255	1372	1741	255	255	255
1373	2	255	255	255	1373	4	255	255	255	1373	1	255	255	255
1374	305	255	255	255	1374	2	255	255	255	1374	2	255	255	255
1375	724	255	255	255	1375	1	255	255	255	1375	1	255	255	255
1376	1	255	255	255	1376	12	255	255	255	1376	25	255	255	255
1377	99	255	255	255	1377	1	255	255	255	1377	1	255	255	255
1378	1	255	255	255	1378	1	255	255	255	1378	1	255	255	255
1379	37	255	255	255	1379	167	255	255	255	1379	1	255	255	255
1380	31	255	255	255	1380	24	255	255	255	1380	1	255	255	255
1381	167	255	255	255	1381	1	255	255	255	1381	1097	255	255	255
1382	132	255	255	255	1382	4	255	255	255	1382	3	255	255	255
1383	77	255	255	255	1383	1	255	255	255	1383	325	255	255	255
1384	14	255	255	255	1384	1	255	255	255	1384	1	255	255	255
1385	1	255	255	255	1385	1	255	255	255	1385	1	255	255	255
1386	1	255	255	255	1386	1	255	255	255	1386	1	255	255	255
1387	4	255	255	255	1387	1	255	255	255	1387	1	255	255	255
1388	1	255	255	255	1388	1	255	255	255	1388	2	255	255	255
1389	1	255	255	255	1389	1	255	255	255	1389	349	255	255	255
1390	1	255	255	255	1390	1	255	255	255	1390	1	255	255	255
1391	7	255	255	255	1391	1	255	255	255	1391	1	255	255	255
1392	29	255	255	255	1392	1	255	255	255	1392	1	255	255	255
1393	2	255	255	255	1393	2	255	255	255	1393	1	255	255	255
1394	1	255	255	255	1394	1	255	255	255	1394	1	255	255	255
1395	74	255	255	255	1395	595	255	255	255	1395	1	255	255	255
1396	1	255	255	255	1396	1	255	255	255	1396	408	255	255	255
1397	1	255	255	255	1397	1	255	255	255	1397	1	255	255	255
1398	1	255	255	255	1398	1	255	255	255	1398	166	255	255	255
1399	1	255	255	255	1399	542	255	255	255	1399	18	255	255	255
1400	1	255	255	255	1400	1	255	255	255	1400	234	255	255	255
1401	2	255	255	255	1401	5	255	255	255	1401	1	255	255	255
1402	1	255	255	255	1402	1	255	255	255	1402	1	255	255	255
1403	1	255	255	255	1403	1	255	255	255	1403	1	255	255	255
1404	4	255	255	255	1404	1	255	255	255	1404	1	255	255	255
1405	1	255	255	255	1405	2	255	255	255	1405	2	255	255	255
1406	4	255	255	255	1406	1	255	255	255	1406	1	255	255	255
1407	4	255	255	255	1407	1	255	255	255	1407	1	255	255	255
1408	1	255	255	255	1408	11	255	255	255	1408	1	255	255	255
1409	1	255	255	255	1409	4	255	255	255	1409	1	255	255	255
1410	2	255	255	255	1410	1	255	255	255	1410	1	255	255	255
1411	29	255	255	255	1411	1	255	255	255	1411	41	255	255	255
1412	2	255	255	255	1412	1	255	255	255	1412	1	255	255	255
1413	1	255	255	255	1413	1	255	255	255	1413	5	255	255	255
1414	4	255	255	255	1414	1	255	255	255	1414	1	255	255	255
1415	2	255	255	255	1415	1	255	255	255	1415	232	255	255	255
1416	5	255	255	255	1416	2	255	255	255	1416	2	255	255	255
1417	1	255	255	255	1417	1	255	255	255	1417	1	255	255	255
1418	1	255	255	255	1418	1	255	255	255	1418	1	255	255	255
1419	5	255	255	255	1419	2	255	255	255	1419	1	255	255	255
1420	13	255	255	255	1420	2	255	255	255	1420	1	255	255	255
1421	164	255	255	255	1421	1	255	255	255	1421	187	255	255	255
1422	117	255	255	255	1422	334	255	255	255	1422	1	255	255	255
1423	53	255	255	255	1423	1	255	255	255	1423	2	255	255	255
1424	96	255	255	255	1424	2	255	255	255	1424	200	255	255	255
1425	3	255	255	255	1425	1	255	255	255	1425	3	255	255	255
1426	123	255	255	255	1426	1	255	255	255	1426	1	255	255	255
1427	30	255	255	255	1427	1	255	255	255	1427	1	255	255	255
1428	6549	255	255	255	1428	3	255	255	255	1428	1	255	255	255
1429	7	255	255	255	1429	1	255	255	255	1429	1	255	255	255
1430	1	255	255	255	1430	1	255	255	255	1430	1681	255	255	255
1431	4	255	255	255	1431	1	255	255	255	1431	1	255	255	255
1432	1	255	255	255	1432	1	255	255	255	1432	1	255	255	255
1433	1	255	255	255	1433	1	255	255	255	1433	1	255	255	255
1434	1	255	255	255	1434	233	255	255	255	1434	301	255	255	255
1435	6	255	255	255	1435	3	255	255	255	1435	10215	255	255	255
1436	3	255	255	255	1436	1	255	255	255	1436	443	255	255	255
1437	1	255	255	255	1437	1	255	255	255	1437	1	255	255	255
1438	47	255	255	255	1438	2	255	255	255	1438	3	255	255	255
1439	1	255	255	255	1439	1	255	255	255	1439	3	255	255	255
1440	1	255	255	255	1440	1	255	255	255	1440	1	255	255	255
1441	1	255	255	255	1441	5	255	255	255	1441	1	255	255	255
1442	4	255	255	255	1442	919	255	255	255	1442	1	255	255	255
1443	106	255	255	255	1443	1	255	255	255	1443	3	255	255	255
1444	10	255	255	255	1444	2	255	255	255	1444	1	255	255	255
1445	1	255	255	255	1445	3	255	255	255	1445	1	255	255	255
1446	2	255	255	255	1446	1	255	255	255	1446	1	255	255	255
1447	2	255	255	255	1447	1	255	255	255	1447	1	255	255	255
1448	895	255	255	255	1448	2	255	255	255	1448	1	255	255	255
1449	116	255	255	255	1449	3	255	255	255	1449	1	255	255	255
1450	31	255	255	255	1450	1	255	255	255	1450	1	255	255	255
1451	788	255	255	255	1451	1	255	255	255	1451	1	255	255	255
1452	228	255	255	255	1452	15	255	255	255	1452	4	255	255	255
1453	4	255	255	255	1453	1	255	255	255	1453	1	255	255	255
1454	1	255	255	255	1454	1	255	255	255	1454	2	255	255	255
1455	60	255	255	255	1455	1	255	255	255	1455	1	255	255	255
1456	383	255	255	255	1456	148	255	255	255	1456	2003	255	255	255
1457	3	255	255	255	1457	1	255	255	255	1457	1	255	255	255
1458	1	255	255	255	1458	1	255	255	255	1458	3	255	255	255
1459	2	255	255	255	1459	1	255	255	255	1459	1	255	255	255
1460	2	255	255	255	1460	1	255	255	255	1460	1	255	255	255
1461	1	255	255	255	1461	1	255	255	255	1461	2	255	255	255
1462	1	255	255	255	1462	1	255	255	255	1462	1	255	255	255
1463	189	255	255	255	1463	69	255	255	255	1463	1	255	255	255
1464	9	255	255	255	1464	131	255	255	255	1464	1	255	255	255
1465	313	255	255	255	1465	707	255	255	255	1465	1	255	255	255
1466	1	255	255	255	1466	562	255	255	255	1466	2	255	255	255
1467	2	255	255	255	1467	3	255	255	255	1467	7	255	255	255
1468	1	255	255	255	1468	3	255	255	255	1468	2	255	255	255
1469	2	255	255	255	1469	1595	255	255	255	1469	1	255	255	255
1470	319	255	255	255	1470	30	255	255	255	1470	4	255	255	255
1471	39	255	255	255	1471	2	255	255	255	1471	21	255	255	255
1472	1	255	255	255	1472	1	255	255	255	1472	64	255	255	255
1473	1	255	255	255	1473	19	255	255	255	1473	1	255	255	255
1474	1	255	255	255	1474	1	255	255	255	1474	14	255	255	255
1475	59	255	255	255	1475	1	255	255	255	1475	1	255	255	255
1476	1	255	255	255	1476	5	255	255	255	1476	1	255	255	255
1477	11	255	255	255	1477	1	255	255	255	1477	1	255	255	255
1478	1	255	255	255	1478	4	255	255	255	1478	1223	255	255	255
1479	1	255	255	255	1479	1	255	255	255	1479	1	255	255	255
1480	2	255	255	255	1480	1	255	255	255	1480	2	255	255	255
1481	1	255	255	255	1481	1	255	255	255	1481	74	255	255	255
1482	4	255	255	255	1482	1	255	255	255	1482	1	255	255	255
1483	8	255	255	255	1483	1	255	255	255	1483	1	255	255	255
1484	1	255	255	255	1484	1	255	255	255	1484	1	255	255	255
1485	168	255	255	255	1485	1	255	255	255	1485	15	255	255	255
1486	6465	255	255	255	1486	51	255	255	255	1486	2	255	255	255
1487	1	255	255	255	1487	1	255	255	255	1487	1	255	255	255
1488	5	255	255	255	1488	2	255	255	255	1488	1	255	255	255
1489	2	255	255	255	1489	1	255	255	255	1489	3	255	255	255
1490	2	255	255	255	1490	1	255	255	255	1490	2	255	255	255
1491	1	255	255	255	1491	1	255	255	255	1491	1	255	255	255
1492	1	255	255	255	1492	7	255	255	255	1492	1	255	255	255
1493	5	255	255	255	1493	2	255	255	255	1493	2	255	255	255
1494	1	255	255	255	1494	254	255	255	255	1494	1	255	255	255
1495	1	255	255	255	1495	1	255	255	255	1495	1	255	255	255
1496	26	255	255	255	1496	1	255	255	255	1496	1	255	255	255
1497	1	255	255	255	1497	2	255	255	255	1497	1	255	255	255
1498	3	255	255	255	1498	2	255	255	255	1498	1	255	255	255
1499	42	255	255	255	1499	51	255	255	255	1499	6	255	255	255

Original data for Figure 2D, Densitometric analysis of Nuclear/Perinuclear B-catenin in control and EoE patient esophageal biopsy tissue.

Values are representative of the mean of a sample size N=18. p<0.05**

1500	8	255	255	255	1500	1	255	255	255	1500	1	255	255	255
1501	2	255	255	255	1501	2	255	255	255	1501	3	255	255	255
1502	46	255	255	255	1502	1	255	255	255	1502	1	255	255	255
1503	1	255	255	255	1503	1	255	255	255	1503	1	255	255	255
1504	1	255	255	255	1504	1	255	255	255	1504	195	255	255	255
1505	1	255	255	255	1505	2	255	255	255	1505	1	255	255	255
1506	1	255	255	255	1506	3	255	255	255	1506	2	255	255	255
1507	1	255	255	255	1507	1	255	255	255	1507	3	255	255	255
1508	2	255	255	255	1508	1	255	255	255	1508	1	255	255	255
1509	91	255	255	255	1509	3	255	255	255	1509	1	255	255	255
1510	4	255	255	255	1510	1	255	255	255	1510	3	255	255	255
1511	2	255	255	255	1511	1	255	255	255	1511	1	255	255	255
1512	5	255	255	255	1512	4	255	255	255	1512	27	255	255	255
1513	2	255	255	255	1513	7	255	255	255	1513	1	255	255	255
1514	1	255	255	255	1514	1	255	255	255	1514	1917	255	255	255
1515	2	255	255	255	1515	2	255	255	255	1515	1	255	255	255
1516	66	255	255	255	1516	2	255	255	255	1516	1	255	255	255
1517	5	255	255	255	1517	1	255	255	255	1517	1	255	255	255
1518	61	255	255	255	1518	2	255	255	255	1518	203	255	255	255
1519	1	255	255	255	1519	2	255	255	255	1519	1	255	255	255
1520	15	255	255	255	1520	185	255	255	255	1520	416	255	255	255
1521	43	255	255	255	1521	1	255	255	255	1521	2	255	255	255
1522	1	255	255	255	1522	1	255	255	255	1522	1	255	255	255
1523	2	255	255	255	1523	1	255	255	255	1523	5	255	255	255
1524	4	255	255	255	1524	101	255	255	255	1524	2	255	255	255
1525	2	255	255	255	1525	3	255	255	255	1525	1	255	255	255
1526	79	255	255	255	1526	20	255	255	255	1526	4	255	255	255
1527	1	255	255	255	1527	1	255	255	255	1527	1	255	255	255
1528	27	255	255	255	1528	1	255	255	255	1528	1	255	255	255
1529	1	255	255	255	1529	1	255	255	255	1529	1	255	255	255
1530	1	255	255	255	1530	1	255	255	255	1530	1	255	255	255
1531	74	255	255	255	1531	1	255	255	255	1531	170	255	255	255
1532	1	255	255	255	1532	1	255	255	255	1532	180	255	255	255
1533	3	255	255	255	1533	2	255	255	255	1533	1	255	255	255
1534	32	255	255	255	1534	2	255	255	255	1534	1	255	255	255
1535	1	255	255	255						1535	1	255	255	255
1536	1	255	255	255						1536	1	255	255	255
1537	13	255	255	255						1537	2	255	255	255
1538	67	255	255	255						1538	2	255	255	255
1539	76	255	255	255						1539	2	255	255	255
1540	1	255	255	255						1540	3	255	255	255
1541	2	255	255	255						1541	1	255	255	255
1542	1	255	255	255						1542	1840	255	255	255
1543	34	255	255	255						1543	2	255	255	255
1544	1	255	255	255						1544	2	255	255	255
1545	1	255	255	255						1545	1	255	255	255
1546	1	255	255	255						1546	17	255	255	255
1547	28	255	255	255						1547	1	255	255	255
1548	1	255	255	255						1548	4983	255	255	255
1549	1	255	255	255						1549	1	255	255	255
1550	5	255	255	255						1550	50	255	255	255
1551	1	255	255	255						1551	76	255	255	255
1552	59	255	255	255						1552	2	255	255	255
1553	1	255	255	255						1553	5052	255	255	255
1554	184	255	255	255						1554	265	255	255	255
1555	2	255	255	255										
1556	2	255	255	255										
1557	1	255	255	255										
1558	1	255	255	255										
1559	1	255	255	255										
1560	6	255	255	255										
1561	2	255	255	255										
1562	1	255	255	255										
1563	23	255	255	255										
1564	2	255	255	255										
1565	1	255	255	255										
1566	8	255	255	255										
1567	117	255	255	255										
1568	2	255	255	255										
1569	5	255	255	255										
1570	20	255	255	255										
1571	263	255	255	255										
1572	115	255	255	255										
1573	1800	255	255	255										
1574	43	255	255	255										
1575	2	255	255	255										
1576	1	255	255	255										
1577	2	255	255	255										
1578	3	255	255	255										
1579	18	255	255	255										
1580	1	255	255	255										
1581	40	255	255	255										
1582	14	255	255	255										
1583	1	255	255	255										
1584	3	255	255	255										
1585	2	255	255	255										
1586	1	255	255	255										
1587	1	255	255	255										

Original data for Figure 2D, Densitometric analysis of Nuclear/Perinuclear B-catenin in control and EoE patient esophageal biopsy tissue.

Values are representative of the mean of a sample size N=18. p<0.05**

EoE
EoE patient esophageal biopsy tissue

tissue

Count	Total Area	Average Size		
Nuclear A	21	255	255	255
2	1	255	255	255
3	16	255	255	255
4	53	255	255	255
5	3	255	255	255
6	128	255	255	255
7	1	255	255	255
8	430	255	255	255
9	1	255	255	255
10	33	255	255	255
11	13	255	255	255
12	1	255	255	255
13	15	255	255	255
14	60	255	255	255
15	499	255	255	255
16	48	255	255	255
17	2	255	255	255
18	77	255	255	255
19	2	255	255	255
20	55	255	255	255
21	180	255	255	255
22	1	255	255	255
23	63	255	255	255
24	2	255	255	255
25	28	255	255	255
26	1	255	255	255
27	23	255	255	255
28	1	255	255	255
29	326	255	255	255
30	136	255	255	255
31	123	255	255	255
32	6	255	255	255
33	2	255	255	255
34	3	255	255	255
35	2	255	255	255
36	447	255	255	255
37	1	255	255	255
38	103	255	255	255
39	45	255	255	255
40	4	255	255	255
41	1	255	255	255
42	1	255	255	255
43	11	255	255	255
44	1	255	255	255
45	159	255	255	255
46	85	255	255	255
47	325	255	255	255
48	24	255	255	255
49	190	255	255	255
50	20	255	255	255
51	43	255	255	255
52	15	255	255	255
53	731	255	255	255
54	1	255	255	255
55	1	255	255	255
56	1	255	255	255
57	289	255	255	255
58	2	255	255	255
59	75	255	255	255
60	46	255	255	255
61	84	255	255	255
62	1268	255	255	255
63	776	255	255	255
64	1	255	255	255
65	13	255	255	255
66	6	255	255	255
67	68	255	255	255
68	155	255	255	255
69	1	255	255	255
70	19	255	255	255
71	53	255	255	255
72	1	255	255	255
73	1	255	255	255
74	38	255	255	255
75	58	255	255	255
76	3	255	255	255
77	1	255	255	255
78	1	255	255	255
79	8	255	255	255
80	1	255	255	255
81	1	255	255	255
82	158	255	255	255
83	1	255	255	255
84	3	255	255	255
85	20	255	255	255
86	126	255	255	255
87	4	255	255	255
88	2	255	255	255
89	3	255	255	255
90	79	255	255	255
91	229	255	255	255
92	123	255	255	255
93	21	255	255	255
94	1	255	255	255
95	62	255	255	255
96	36	255	255	255
97	3	255	255	255
98	1	255	255	255
99	1	255	255	255
100	229	255	255	255
101	1	255	255	255
102	21	255	255	255
103	2	255	255	255
104	1	255	255	255
105	4	255	255	255
106	1	255	255	255
107	2	255	255	255
108	355	255	255	255
109	25	255	255	255
110	1	255	255	255
111	2	255	255	255
112	2	255	255	255
113	1	255	255	255
114	29	255	255	255
115	1	255	255	255
116	1	255	255	255
117	1	255	255	255
118	1	255	255	255
119	25	255	255	255

EoE
EoE patient esophageal biopsy tissue

Count	Total Area	Average Size		
Nuclear A	43	255	255	255
2	24	255	255	255
3	4	255	255	255
4	2	255	255	255
5	79	255	255	255
6	1	255	255	255
7	7	255	255	255
8	2	255	255	255
9	17	255	255	255
10	2	255	255	255
11	7	255	255	255
12	45	255	255	255
13	17	255	255	255
14	147	255	255	255
15	1	255	255	255
16	5	255	255	255
17	2	255	255	255
18	1	255	255	255
19	1	255	255	255
20	1	255	255	255
21	16	255	255	255
22	64	255	255	255
23	66	255	255	255
24	2	255	255	255
25	2	255	255	255
26	1	255	255	255
27	3	255	255	255
28	1	255	255	255
29	137	255	255	255
30	1	255	255	255
31	1	255	255	255
32	1	255	255	255
33	26	255	255	255
34	1	255	255	255
35	1	255	255	255
36	5	255	255	255
37	6	255	255	255
38	19	255	255	255
39	4	255	255	255
40	38	255	255	255
41	10	255	255	255
42	1	255	255	255
43	1	255	255	255
44	1	255	255	255
45	74	255	255	255
46	52	255	255	255
47	4	255	255	255
48	6	255	255	255
49	2	255	255	255
50	20	255	255	255
51	1	255	255	255
52	1	255	255	255
53	1	255	255	255
54	10	255	255	255
55	81	255	255	255
56	149	255	255	255
57	10	255	255	255
58	1	255	255	255
59	1	255	255	255
60	790	255	255	255
61	2	255	255	255
62	3	255	255	255
63	1	255	255	255
64	49	255	255	255
65	53	255	255	255
66	88	255	255	255
67	1	255	255	255
68	648	255	255	255
69	4	255	255	255
70	1	255	255	255
71	22	255	255	255
72	1	255	255	255
73	2	255	255	255
74	50	255	255	255
75	4	255	255	255
76	1	255	255	255
77	1	255	255	255
78	5	255	255	255
79	1	255	255	255
80	5	255	255	255
81	2	255	255	255
82	1	255	255	255
83	1	255	255	255
84	239	255	255	255
85	3	255	255	255
86	1	255	255	255
87	88	255	255	255
88	1	255	255	255
89	675	255	255	255
90	1	255	255	255
91	1	255	255	255
92	270	255	255	255
93	11	255	255	255
94	45	255	255	255
95	1	255	255	255
96	26	255	255	255
97	2	255	255	255
98	1	255	255	255
99	1	255	255	255
100	1	255	255	255
101	3	255	255	255
102	22	255	255	255
103	4	255	255	255
104	971	255	255	255
105	3	255	255	255
106	162	255	255	255
107	1	255	255	255
108	1	255	255	255
109	1	255	255	255
110	22	255	255	255
111	1	255	255	255
112	10	255	255	255
113	21	255	255	255
114	4	255	255	255
115	1	255	255	255
116	2	255	255	255
117	2	255	255	255
118	1	255	255	255
119	34	255	255	255

EoE
EoE patient esophageal biopsy tissue

Count	Total Area	Average Size		
Nuclear A	32	255	255	255
2	1	255	255	255
3	15	255	255	255
4	2	255	255	255
5	1	255	255	255
6	2	255	255	255
7	1	255	255	255
8	1	255	255	255
9	33	255	255	255
10	232	255	255	255
11	523	255	255	255
12	1	255	255	255
13	1	255	255	255
14	56	255	255	255
15	1	255	255	255
16	2	255	255	255
17	58	255	255	255
18	1	255	255	255
19	1	255	255	255
20	6	255	255	255
21	2	255	255	255
22	1	255	255	255
23	1	255	255	255
24	18	255	255	255
25	1	255	255	255
26	1	255	255	255
27	3	255	255	255
28	17	255	255	255
29	1	255	255	255
30	5	255	255	255
31	17	255	255	255
32	46	255	255	255
33	1	255	255	255
34	25	255	255	255
35	17	255	255	255
36	29	255	255	255
37	1	255	255	255
38	61	255	255	255
39	522	255	255	255
40	27	255	255	255
41	2	255	255	255
42	9	255	255	255
43	1	255	255	255
44	8	255	255	255
45	83	255	255	255
46	1	255	255	255
47	1	255	255	255
48	1	255	255	255
49	13	255	255	255
50	6	255	255	255
51	3	255	255	255
52	8	255	255	255
53	1	255	255	255
54	1	255	255	255
55	61	255	255	255
56	1	255	255	255
57	1	255	255	255
58	2	255	255	255
59	13	255	255	255
60	1	255	255	255
61	36	255	255	255
62	50	255	255	255
63	5	255	255	255
64	1	255	255	255
65	2	255	255	255
66	2	255	255	255
67	14	255	255	255
68	5	255	255	255
69	19	255	255	255
70	7	255	255	255
71	41	255	255	255
72	1	255	255	255
73	210	255	255	255
74	3	255	255	255
75	1	255	255	255
76	49	255	255	255
77	12	255	255	255
78	2	255	255	255
79	7	255	255	255
80	2	255	255	255
81	26	255	255	255
82	431	255	255	255
83	203	255	255	255
84	19	255	255	255
85	192	255	255	255
86	1	255	255	255
87	1	255	255	255
88	3	255	255	255
89	64	255	255	255
90	9	255	255	255
91	47	255	255	255
92	1	255	255	255
93	1	255	255	255
94	2	255	255	255
95	122	255	255	255
96	22	255	255	255
97	21	255	255	255
98	8	255	255	255
99	2	255	255	255
100	19	255	255	255
101	1	255	255	255
102	5	255	255	255
103	2	255	255	255
104	56	255	255	255
105	2	255	255	255
106	1	255	255	255
107	2	255	255	255
108	3	255	255	255
109	15	255	255	255
110	7	255	255	255
111	1	255	255	255
112	1	255	255	255
113	1	255	255	255
114	2	255	255	255
115	12	255	255	255
116	60	255	255	255
117	1	255	255	255
118	3	255	255	255
119	1	255	255	255

Original data for Figure 2D, Densitometric analysis of Nuclear/Perinuclear B-catenin in control and EoE patient esophageal biopsy tissue.
Values are representative of the mean of a sample size N=18. p<0.05**

120	2	255	255	255	120	5	255	255	255	120	1	255	255	255
121	23	255	255	255	121	1	255	255	255	121	15	255	255	255
122	1	255	255	255	122	1	255	255	255	122	70	255	255	255
123	48	255	255	255	123	4	255	255	255	123	2	255	255	255
124	407	255	255	255	124	3	255	255	255	124	3	255	255	255
125	1	255	255	255	125	1	255	255	255	125	74	255	255	255
126	164	255	255	255	126	4	255	255	255	126	1	255	255	255
127	1	255	255	255	127	2	255	255	255	127	5	255	255	255
128	29	255	255	255	128	1	255	255	255	128	1	255	255	255
129	1	255	255	255	129	1	255	255	255	129	1	255	255	255
130	1	255	255	255	130	3	255	255	255	130	4	255	255	255
131	166	255	255	255	131	2	255	255	255	131	4031	255	255	255
132	1	255	255	255	132	943	255	255	255	132	1	255	255	255
133	88	255	255	255	133	2	255	255	255	133	2	255	255	255
134	94	255	255	255	134	2	255	255	255	134	1	255	255	255
135	29	255	255	255	135	49	255	255	255	135	50	255	255	255
136	1	255	255	255	136	1	255	255	255	136	3	255	255	255
137	3	255	255	255	137	1	255	255	255	137	954	255	255	255
138	340	255	255	255	138	1	255	255	255	138	1	255	255	255
139	15	255	255	255	139	3	255	255	255	139	1	255	255	255
140	16	255	255	255	140	1	255	255	255	140	2	255	255	255
141	2	255	255	255	141	62	255	255	255	141	1	255	255	255
142	2	255	255	255	142	1	255	255	255	142	3	255	255	255
143	2	255	255	255	143	4	255	255	255	143	13	255	255	255
144	19	255	255	255	144	3	255	255	255	144	1	255	255	255
145	1	255	255	255	145	1	255	255	255	145	2	255	255	255
146	21	255	255	255	146	263	255	255	255	146	92	255	255	255
147	1	255	255	255	147	1	255	255	255	147	288	255	255	255
148	1	255	255	255	148	241	255	255	255	148	4	255	255	255
149	1	255	255	255	149	2	255	255	255	149	1	255	255	255
150	2	255	255	255	150	98	255	255	255	150	11	255	255	255
151	2	255	255	255	151	13	255	255	255	151	2	255	255	255
152	3	255	255	255	152	1	255	255	255	152	1	255	255	255
153	236	255	255	255	153	1	255	255	255	153	1	255	255	255
154	13	255	255	255	154	3	255	255	255	154	18	255	255	255
155	1	255	255	255	155	2	255	255	255	155	35	255	255	255
156	5	255	255	255	156	1	255	255	255	156	12	255	255	255
157	2	255	255	255	157	1	255	255	255	157	2	255	255	255
158	6	255	255	255	158	43	255	255	255	158	141	255	255	255
159	1	255	255	255	159	2	255	255	255	159	17	255	255	255
160	2	255	255	255	160	1	255	255	255	160	6	255	255	255
161	1794	255	255	255	161	1	255	255	255	161	312	255	255	255
162	2	255	255	255	162	5	255	255	255	162	55	255	255	255
163	2	255	255	255	163	1	255	255	255	163	3	255	255	255
164	2	255	255	255	164	162	255	255	255	164	2	255	255	255
165	1	255	255	255	165	1	255	255	255	165	47	255	255	255
166	73	255	255	255	166	505	255	255	255	166	51	255	255	255
167	1	255	255	255	167	2	255	255	255	167	1	255	255	255
168	1	255	255	255	168	2	255	255	255	168	5	255	255	255
169	1	255	255	255	169	52	255	255	255	169	1	255	255	255
170	59	255	255	255	170	1	255	255	255	170	27	255	255	255
171	1	255	255	255	171	1	255	255	255	171	88	255	255	255
172	8	255	255	255	172	11	255	255	255	172	2	255	255	255
173	112	255	255	255	173	34	255	255	255	173	121	255	255	255
174	1	255	255	255	174	1	255	255	255	174	1	255	255	255
175	10	255	255	255	175	111	255	255	255	175	2	255	255	255
176	2	255	255	255	176	1	255	255	255	176	2	255	255	255
177	5	255	255	255	177	1	255	255	255	177	4	255	255	255
178	1	255	255	255	178	748	255	255	255	178	6	255	255	255
179	4	255	255	255	179	4	255	255	255	179	3	255	255	255
180	396	255	255	255	180	17	255	255	255	180	47	255	255	255
181	230	255	255	255	181	9	255	255	255	181	1	255	255	255
182	2	255	255	255	182	1	255	255	255	182	23	255	255	255
183	2	255	255	255	183	1	255	255	255	183	2	255	255	255
184	7	255	255	255	184	1	255	255	255	184	1	255	255	255
185	1	255	255	255	185	1	255	255	255	185	32	255	255	255
186	1	255	255	255	186	10	255	255	255	186	81	255	255	255
187	1	255	255	255	187	3	255	255	255	187	5	255	255	255
188	81	255	255	255	188	665	255	255	255	188	2	255	255	255
189	1	255	255	255	189	1	255	255	255	189	25	255	255	255
190	3	255	255	255	190	38	255	255	255	190	1	255	255	255
191	2	255	255	255	191	77	255	255	255	191	1	255	255	255
192	8	255	255	255	192	2	255	255	255	192	161	255	255	255
193	61	255	255	255	193	1	255	255	255	193	2	255	255	255
194	1	255	255	255	194	2	255	255	255	194	30	255	255	255
195	85	255	255	255	195	3	255	255	255	195	3	255	255	255
196	20	255	255	255	196	2	255	255	255	196	1	255	255	255
197	1	255	255	255	197	18	255	255	255	197	4	255	255	255
198	229	255	255	255	198	1	255	255	255	198	22	255	255	255
199	2	255	255	255	199	1	255	255	255	199	3	255	255	255
200	36	255	255	255	200	6	255	255	255	200	1	255	255	255
201	1	255	255	255	201	1	255	255	255	201	113	255	255	255
202	59	255	255	255	202	13	255	255	255	202	1	255	255	255
203	187	255	255	255	203	1	255	255	255	203	1	255	255	255
204	7	255	255	255	204	162	255	255	255	204	2	255	255	255
205	53	255	255	255	205	14	255	255	255	205	20	255	255	255
206	149	255	255	255	206	4	255	255	255	206	14	255	255	255
207	1	255	255	255	207	56	255	255	255	207	26	255	255	255
208	1	255	255	255	208	1	255	255	255	208	2	255	255	255
209	1	255	255	255	209	2	255	255	255	209	1	255	255	255
210	2	255	255	255	210	1	255	255	255	210	1	255	255	255
211	1	255	255	255	211	9	255	255	255	211	2	255	255	255
212	29	255	255	255	212	1	255	255	255	212	1	255	255	255
213	3	255	255	255	213	1	255	255	255	213	94	255	255	255
214	47	255	255	255	214	77	255	255	255	214	1	255	255	255
215	66	255	255	255	215	23	255	255	255	215	2	255	255	255
216	35	255	255	255	216	1	255	255	255	216	1	255	255	255
217	5	255	255	255	217	149	255	255	255	217	2	255	255	255
218	241	255	255	255	218	38	255	255	255	218	1	255	255	255
219	22	255	255	255	219	7	255	255	255	219	1	255	255	255
220	2	255	255	255	220	1	255	255	255	220	1	255	255	255
221	4	255	255	255	221	34	255	255	255	221	450	255	255	255
222	18	255	255	255	222	1980	255	255	255	222	6	255	255	255
223	53	255	255	255	223	946	255	255	255	223	55	255	255	255
224	107	255	255	255	224	2	255	255	255	224	1	255	255	255
225	2	255	255	255	225	43	255	255	255	225	177	255	255	255
226	2	255	255	255	226	1	255	255	255	226	1	255	255	255
227	35	255	255	255	227	1	255	255	255	227	41	255	255	255
228	65	255	255	255	228	1	255	255	255	228	1	255	255	255
229	57	255	255	255	229	1	255	255	255	229	33	255	255	255
230	10	255	255	255	230	2	255	255	255	230	2	255	255	255
231	84	255	255	255	231	14	255	255	255	231	22	255	255	255
232	1	255	255	255	232	4	255	255	255	232	8	255	255	255
233	254	255	255	255	233	38	255	255	255	233	5	255	255	255
234	69	255	255	255	234	4	255	255	255	234	1	255	255	255
235	1	255	255	255	235	8	255	255	255	235	108	255	255	255
236	653	255	255	255	236	2	255	255	255	236	1	255	255	255
237	796	255	255	255	237	20	255	255	255	237	15	255	255	255
238	3	255	255	255	238	2	255	255	255	238	3	255	255	255
239	3	255	255	255	239	3	255	255	255	239	5	255	255	255
240	1	255	255	255	240	21	255	255	255	240	1	255	255	255
241	1	255	255	255	241	1	255	255	255	241	59	255	255	255
242	39	255	255	255	242	20	255	255	255	242	2	255	255	255
243	11	255	255	255	243	1	255	255	255	243	55	255	255	255
244	7	255	255	255	244	1	255	255	255	244	1	255	255	255
245	1	255	255	255	245	38	255	255	255	245	1	255	255	255
246	7	255	255	255	246	24	255	255	255	246	79	255	255	255
247	279	255	255	255	247	66	255	255	255	247	1	255	255	255
248	128	255	255	255	248	1	255	255	255	248	11	255	255	255
249	1	255	255	255	249	2	255	255	255	249	113	255	255	255
250	1	255	255	255	250	1	255	255	255	250	3	255	255	255
251	1	255	255	255	251	196	255	255	255	251	73	255	255	255
252	28	255	255	255	252	17	255	255	255	252	25	255	255	255
253	5	255	255	255	253	7	255	255	255	253	2	255	255	255
254	4	255	255	255	254	3	255	255	255	254	38	255	255	255
255	1	255	255	255	255	2	255	255	255	255	7	255	255	255
256	1	255	255	255	256	11	255	255	255	256	3	255	255	255
257	21	255	255	255	257	4	255	255	255	257	1	255	255	255

Original data for Figure 2D, Densitometric analysis of Nuclear/Perinuclear B-catenin in control and EoE patient esophageal biopsy tissue.
Values are representative of the mean of a sample size N=18. p<0.05**

258	2883	255	255	255	258	25	255	255	255	258	12	255	255	255
259	1	255	255	255	259	1	255	255	255	259	9	255	255	255
260	4	255	255	255	260	2	255	255	255	260	2	255	255	255
261	1	255	255	255	261	27	255	255	255	261	2	255	255	255
262	1	255	255	255	262	1	255	255	255	262	2	255	255	255
263	1	255	255	255	263	755	255	255	255	263	1	255	255	255
264	2	255	255	255	264	1	255	255	255	264	1	255	255	255
265	160	255	255	255	265	11	255	255	255	265	5	255	255	255
266	1	255	255	255	266	1	255	255	255	266	2	255	255	255
267	26	255	255	255	267	5	255	255	255	267	2	255	255	255
268	16	255	255	255	268	2	255	255	255	268	2	255	255	255
269	1	255	255	255	269	1	255	255	255	269	169	255	255	255
270	244	255	255	255	270	82	255	255	255	270	2	255	255	255
271	35	255	255	255	271	119	255	255	255	271	1	255	255	255
272	110	255	255	255	272	3	255	255	255	272	1	255	255	255
273	9	255	255	255	273	310	255	255	255	273	16	255	255	255
274	55	255	255	255	274	2	255	255	255	274	1	255	255	255
275	103	255	255	255	275	2	255	255	255	275	5	255	255	255
276	10	255	255	255	276	3	255	255	255	276	2	255	255	255
277	1	255	255	255	277	3	255	255	255	277	2	255	255	255
278	4	255	255	255	278	2	255	255	255	278	14	255	255	255
279	2	255	255	255	279	2	255	255	255	279	8	255	255	255
280	6	255	255	255	280	14	255	255	255	280	7	255	255	255
281	1	255	255	255	281	1	255	255	255	281	1	255	255	255
282	1	255	255	255	282	2	255	255	255	282	6	255	255	255
283	1	255	255	255	283	16	255	255	255	283	1	255	255	255
284	810	255	255	255	284	23	255	255	255	284	132	255	255	255
285	2	255	255	255	285	20	255	255	255	285	44	255	255	255
286	1	255	255	255	286	1	255	255	255	286	17	255	255	255
287	1	255	255	255	287	141	255	255	255	287	181	255	255	255
288	2	255	255	255	288	1	255	255	255	288	2	255	255	255
289	11	255	255	255	289	6	255	255	255	289	2	255	255	255
290	154	255	255	255	290	1	255	255	255	290	2	255	255	255
291	1	255	255	255	291	3	255	255	255	291	1	255	255	255
292	1	255	255	255	292	4	255	255	255	292	1	255	255	255
293	2	255	255	255	293	36	255	255	255	293	30	255	255	255
294	26	255	255	255	294	1	255	255	255	294	1	255	255	255
295	1	255	255	255	295	25	255	255	255	295	1	255	255	255
296	2	255	255	255	296	1	255	255	255	296	1	255	255	255
297	2	255	255	255	297	7	255	255	255	297	1	255	255	255
298	1	255	255	255	298	1	255	255	255	298	1	255	255	255
299	1	255	255	255	299	1	255	255	255	299	1	255	255	255
300	1	255	255	255	300	2	255	255	255	300	2	255	255	255
301	4	255	255	255	301	3	255	255	255	301	1	255	255	255
302	2	255	255	255	302	2	255	255	255	302	1	255	255	255
303	1	255	255	255	303	14	255	255	255	303	1	255	255	255
304	24	255	255	255	304	11	255	255	255	304	8	255	255	255
305	62	255	255	255	305	1599	255	255	255	305	1	255	255	255
306	162	255	255	255	306	1	255	255	255	306	1	255	255	255
307	1	255	255	255	307	78	255	255	255	307	3	255	255	255
308	78	255	255	255	308	50	255	255	255	308	45	255	255	255
309	1	255	255	255	309	1	255	255	255	309	6	255	255	255
310	4	255	255	255	310	7	255	255	255	310	1	255	255	255
311	78	255	255	255	311	15	255	255	255	311	1	255	255	255
312	1	255	255	255	312	3	255	255	255	312	21	255	255	255
313	190	255	255	255	313	1	255	255	255	313	1	255	255	255
314	111	255	255	255	314	17	255	255	255	314	1	255	255	255
315	30	255	255	255	315	5	255	255	255	315	10	255	255	255
316	38	255	255	255	316	3	255	255	255	316	35	255	255	255
317	93	255	255	255	317	6	255	255	255	317	2	255	255	255
318	14	255	255	255	318	30	255	255	255	318	3	255	255	255
319	324	255	255	255	319	1	255	255	255	319	3	255	255	255
320	36	255	255	255	320	45	255	255	255	320	10	255	255	255
321	1	255	255	255	321	1	255	255	255	321	2	255	255	255
322	3	255	255	255	322	124	255	255	255	322	73	255	255	255
323	1	255	255	255	323	10	255	255	255	323	10	255	255	255
324	1	255	255	255	324	6	255	255	255	324	16	255	255	255
325	1	255	255	255	325	3	255	255	255	325	18	255	255	255
326	92	255	255	255	326	1	255	255	255	326	1	255	255	255
327	1	255	255	255	327	17	255	255	255	327	1	255	255	255
328	1	255	255	255	328	2	255	255	255	328	1	255	255	255
329	25	255	255	255	329	145	255	255	255	329	1	255	255	255
330	334	255	255	255	330	41	255	255	255	330	9	255	255	255
331	1	255	255	255	331	2	255	255	255	331	2	255	255	255
332	1	255	255	255	332	2	255	255	255	332	34	255	255	255
333	74	255	255	255	333	5	255	255	255	333	2	255	255	255
334	2	255	255	255	334	1	255	255	255	334	203	255	255	255
335	2	255	255	255	335	1	255	255	255	335	24	255	255	255
336	1	255	255	255	336	1	255	255	255	336	26	255	255	255
337	4	255	255	255	337	131	255	255	255	337	111	255	255	255
338	1	255	255	255	338	1	255	255	255	338	4	255	255	255
339	2	255	255	255	339	3	255	255	255	339	2	255	255	255
340	46	255	255	255	340	1	255	255	255	340	5	255	255	255
341	4	255	255	255	341	96	255	255	255	341	3	255	255	255
342	33	255	255	255	342	11	255	255	255	342	7	255	255	255
343	2	255	255	255	343	22	255	255	255	343	1	255	255	255
344	1	255	255	255	344	1	255	255	255	344	21	255	255	255
345	51	255	255	255	345	3	255	255	255	345	3	255	255	255
346	11	255	255	255	346	4	255	255	255	346	8	255	255	255
347	2	255	255	255	347	3659	255	255	255	347	1	255	255	255
348	54	255	255	255	348	1	255	255	255	348	98	255	255	255
349	21	255	255	255	349	1	255	255	255	349	1	255	255	255
350	18	255	255	255	350	1	255	255	255	350	1	255	255	255
351	110	255	255	255	351	1	255	255	255	351	1	255	255	255
352	1	255	255	255	352	1	255	255	255	352	46	255	255	255
353	55	255	255	255	353	1	255	255	255	353	10	255	255	255
354	38	255	255	255	354	1	255	255	255	354	1	255	255	255
355	1	255	255	255	355	8	255	255	255	355	18	255	255	255
356	92	255	255	255	356	21	255	255	255	356	1	255	255	255
357	5	255	255	255	357	35	255	255	255	357	5	255	255	255
358	111	255	255	255	358	1	255	255	255	358	27	255	255	255
359	100	255	255	255	359	3	255	255	255	359	7	255	255	255
360	1	255	255	255	360	53	255	255	255	360	2	255	255	255
361	109	255	255	255	361	1	255	255	255	361	1	255	255	255
362	20	255	255	255	362	1	255	255	255	362	1	255	255	255
363	1	255	255	255	363	5	255	255	255	363	2	255	255	255
364	2	255	255	255	364	1	255	255	255	364	2	255	255	255
365	24	255	255	255	365	1	255	255	255	365	3	255	255	255
366	1	255	255	255	366	1	255	255	255	366	1	255	255	255
367	77	255	255	255	367	2	255	255	255	367	1	255	255	255
368	1	255	255	255	368	1	255	255	255	368	1	255	255	255
369	1	255	255	255	369	2	255	255	255	369	1	255	255	255
370	1	255	255	255	370	2	255	255	255	370	1	255	255	255
371	399	255	255	255	371	4	255	255	255	371	1	255	255	255
372	14	255	255	255	372	2	255	255	255	372	2	255	255	255
373	5	255	255	255	373	1	255	255	255	373	3	255	255	255
374	1	255	255	255	374	9	255	255	255	374	1	255	255	255
375	28	255	255	255	375	5	255	255	255	375	1	255	255	255
376	5	255	255	255	376	43	255	255	255	376	57	255	255	255
377	2	255	255	255	377	11	255	255	255	377	1	255	255	255
378	169	255	255	255	378	2	255	255	255	378	1	255	255	255
379	2	255	255	255	379	84	255	255	255	379	2	255	255	255
380	18	255	255	255	380	1	255	255	255	380	6	255	255	255
381	1	255	255	255	381	1	255	255	255	381	58	255	255	255
382	5	255	255	255	382	48	255	255	255	382	1	255	255	255
383	1	255	255	255	383	1	255	255	255	383	1	255	255	255
384	108	255	255	255	384	596	255	255	255	384	2	255	255	255
385	108	255	255	255	385	3	255	255	255	385	5	255	255	255
386	13	255	255	255	386	55	255	255	255	386	2	255	255	255
387	56	255	255	255	387	2	255	255	255	387	1	255	255	255
388	5	255	255	255	388	56	255	255	255	388	19	255	255	255
389	25	255	255	255	389	23	255	255	255	389	1	255	255	255
390	39	255	255	255	390	1	255	255	255	390	5	255	255	255
391	4	255	255	255	391	1	255	255	255	391	2	255	255	255
392	93	255	255	255	392	23	255	255	255	392	52	255	255	255
393	1	255	255	255	393	5	255	255	255	393	1	255	255	255
394	61	255	255	255	394	1	255	255	255	394	2	255	255	255
395	1	255	255	255	395	1	255	255	255	395	3	255	255	255

Original data for Figure 2D, Densitometric analysis of Nuclear/Perinuclear B-catenin in control and EoE patient esophageal biopsy tissue.
Values are representative of the mean of a sample size N=18. p<0.05**

396	1	255	255	255	396	1	255	255	255	396	1	255	255	255
397	1	255	255	255	397	3	255	255	255	397	64	255	255	255
398	39	255	255	255	398	1	255	255	255	398	181	255	255	255
399	1	255	255	255	399	198	255	255	255	399	1	255	255	255
400	1	255	255	255	400	1	255	255	255	400	1	255	255	255
401	2	255	255	255	401	46	255	255	255	401	128	255	255	255
402	1	255	255	255	402	2	255	255	255	402	238	255	255	255
403	1	255	255	255	403	8	255	255	255	403	19	255	255	255
404	1	255	255	255	404	21	255	255	255	404	1	255	255	255
405	4	255	255	255	405	100	255	255	255	405	1	255	255	255
406	5	255	255	255	406	4	255	255	255	406	2	255	255	255
407	3	255	255	255	407	48	255	255	255	407	35	255	255	255
408	27	255	255	255	408	1	255	255	255	408	1	255	255	255
409	24	255	255	255	409	1	255	255	255	409	5	255	255	255
410	15	255	255	255	410	53	255	255	255	410	2	255	255	255
411	85	255	255	255	411	2	255	255	255	411	8	255	255	255
412	10	255	255	255	412	1	255	255	255	412	1	255	255	255
413	1	255	255	255	413	3	255	255	255	413	1	255	255	255
414	105	255	255	255	414	3	255	255	255	414	1	255	255	255
415	1	255	255	255	415	2	255	255	255	415	44	255	255	255
416	53	255	255	255	416	2	255	255	255	416	1	255	255	255
417	4	255	255	255	417	1	255	255	255	417	2	255	255	255
418	87	255	255	255	418	53	255	255	255	418	2	255	255	255
419	33	255	255	255	419	1	255	255	255	419	2	255	255	255
420	17	255	255	255	420	5	255	255	255	420	3	255	255	255
421	5	255	255	255	421	2	255	255	255	421	2	255	255	255
422	1	255	255	255	422	7	255	255	255	422	24	255	255	255
423	41	255	255	255	423	3	255	255	255	423	3	255	255	255
424	1	255	255	255	424	1	255	255	255	424	98	255	255	255
425	20	255	255	255	425	1	255	255	255	425	4	255	255	255
426	35	255	255	255	426	1	255	255	255	426	6	255	255	255
427	1	255	255	255	427	1	255	255	255	427	34	255	255	255
428	21	255	255	255	428	2	255	255	255	428	3	255	255	255
429	5	255	255	255	429	2	255	255	255	429	34	255	255	255
430	1	255	255	255	430	2	255	255	255	430	8	255	255	255
431	73	255	255	255	431	77	255	255	255	431	114	255	255	255
432	1	255	255	255	432	1	255	255	255	432	2	255	255	255
433	3	255	255	255	433	1	255	255	255	433	54	255	255	255
434	1	255	255	255	434	3	255	255	255	434	25	255	255	255
435	1	255	255	255	435	1	255	255	255	435	150	255	255	255
436	1	255	255	255	436	2	255	255	255	436	1	255	255	255
437	1	255	255	255	437	48	255	255	255	437	32	255	255	255
438	1	255	255	255	438	1	255	255	255	438	41	255	255	255
439	1	255	255	255	439	1	255	255	255	439	1	255	255	255
440	2	255	255	255	440	597	255	255	255	440	3	255	255	255
441	2	255	255	255	441	2	255	255	255	441	24	255	255	255
442	1	255	255	255	442	44	255	255	255	442	136	255	255	255
443	193	255	255	255	443	5	255	255	255	443	1	255	255	255
444	2	255	255	255	444	48	255	255	255	444	12	255	255	255
445	15	255	255	255	445	1	255	255	255	445	73	255	255	255
446	40	255	255	255	446	14	255	255	255	446	181	255	255	255
447	3	255	255	255	447	1	255	255	255	447	47	255	255	255
448	1	255	255	255	448	3	255	255	255	448	3	255	255	255
449	2	255	255	255	449	56	255	255	255	449	82	255	255	255
450	1	255	255	255	450	1	255	255	255	450	1	255	255	255
451	1	255	255	255	451	5	255	255	255	451	24	255	255	255
452	1	255	255	255	452	5	255	255	255	452	64	255	255	255
453	1	255	255	255	453	1	255	255	255	453	12	255	255	255
454	34	255	255	255	454	44	255	255	255	454	169	255	255	255
455	2	255	255	255	455	6	255	255	255	455	62	255	255	255
456	2	255	255	255	456	21	255	255	255	456	2	255	255	255
457	59	255	255	255	457	1	255	255	255	457	12	255	255	255
458	146	255	255	255	458	2	255	255	255	458	3	255	255	255
459	11	255	255	255	459	1	255	255	255	459	3	255	255	255
460	83	255	255	255	460	2	255	255	255	460	1	255	255	255
461	3	255	255	255	461	1	255	255	255	461	66	255	255	255
462	16	255	255	255	462	1	255	255	255	462	1	255	255	255
463	1235	255	255	255	463	1	255	255	255	463	1	255	255	255
464	1	255	255	255	464	1	255	255	255	464	1	255	255	255
465	1	255	255	255	465	2	255	255	255	465	55	255	255	255
466	31	255	255	255	466	141	255	255	255	466	61	255	255	255
467	4	255	255	255	467	2	255	255	255	467	69	255	255	255
468	1	255	255	255	468	1	255	255	255	468	1	255	255	255
469	1	255	255	255	469	9	255	255	255	469	16	255	255	255
470	1	255	255	255	470	1	255	255	255	470	1	255	255	255
471	80	255	255	255	471	5	255	255	255	471	1	255	255	255
472	133	255	255	255	472	2	255	255	255	472	79	255	255	255
473	1	255	255	255	473	4	255	255	255	473	2	255	255	255
474	15	255	255	255	474	1	255	255	255	474	4	255	255	255
475	1	255	255	255	475	3	255	255	255	475	17	255	255	255
476	1	255	255	255	476	2	255	255	255	476	3	255	255	255
477	2	255	255	255	477	3	255	255	255	477	2	255	255	255
478	77	255	255	255	478	90	255	255	255	478	22	255	255	255
479	57	255	255	255	479	2	255	255	255	479	2	255	255	255
480	200	255	255	255	480	1	255	255	255	480	168	255	255	255
481	54	255	255	255	481	1	255	255	255	481	44	255	255	255
482	22	255	255	255	482	4	255	255	255	482	65	255	255	255
483	2	255	255	255	483	1	255	255	255	483	14	255	255	255
484	2	255	255	255	484	31	255	255	255	484	2	255	255	255
485	1	255	255	255	485	2	255	255	255	485	1	255	255	255
486	31	255	255	255	486	1	255	255	255	486	1	255	255	255
487	1	255	255	255	487	1	255	255	255	487	2	255	255	255
488	55	255	255	255	488	1	255	255	255	488	2	255	255	255
489	1	255	255	255	489	60	255	255	255	489	20	255	255	255
490	2	255	255	255	490	21	255	255	255	490	78	255	255	255
491	1	255	255	255	491	9	255	255	255	491	2	255	255	255
492	7	255	255	255	492	1	255	255	255	492	46	255	255	255
493	1	255	255	255	493	1	255	255	255	493	97	255	255	255
494	1	255	255	255	494	1022	255	255	255	494	30	255	255	255
495	74	255	255	255	495	2	255	255	255	495	1	255	255	255
496	4	255	255	255	496	14	255	255	255	496	116	255	255	255
497	137	255	255	255	497	1	255	255	255	497	1	255	255	255
498	24	255	255	255	498	1	255	255	255	498	2	255	255	255
499	15	255	255	255	499	179	255	255	255	499	5	255	255	255
500	56	255	255	255	500	1	255	255	255	500	14	255	255	255
501	2792	255	255	255	501	1	255	255	255	501	5	255	255	255
502	15	255	255	255	502	2	255	255	255	502	715	255	255	255
503	1	255	255	255	503	1	255	255	255	503	19	255	255	255
504	1	255	255	255	504	1	255	255	255	504	13	255	255	255
505	1	255	255	255	505	1	255	255	255	505	3	255	255	255
506	1	255	255	255	506	1	255	255	255	506	2	255	255	255
507	21	255	255	255	507	2	255	255	255	507	1	255	255	255
508	26	255	255	255	508	1	255	255	255	508	1	255	255	255
509	2	255	255	255	509	8	255	255	255	509	1	255	255	255
510	118	255	255	255	510	55	255	255	255	510	5	255	255	255
511	52	255	255	255	511	1	255	255	255	511	1	255	255	255
512	4	255	255	255	512	28	255	255	255	512	1	255	255	255
513	111	255	255	255	513	1	255	255	255	513	1	255	255	255
514	4	255	255	255	514	11	255	255	255	514	1	255	255	255
515	2	255	255	255	515	48	255	255	255	515	3	255	255	255
516	57	255	255	255	516	2	255	255	255	516	3	255	255	255
517	20	255	255	255	517	3	255	255	255	517	8	255	255	255
518	1	255	255	255	518	16	255	255	255	518	6	255	255	255
519	1	255	255	255	519	5	255	255	255	519	1	255	255	255
520	10	255	255	255	520	1	255	255	255	520	1	255	255	255
521	2	255	255	255	521	1	255	255	255	521	32	255	255	255
522	39	255	255	255	522	48	255	255	255	522	1	255	255	255
523	1	255	255	255	523	328	255	255	255	523	1	255	255	255
524	2	255	255	255	524	4	255	255	255	524	5	255	255	255
525	2	255	255	255	525	1	255	255	255	525	36	255	255	255
526	182	255	255	255	526	18	255	255	255	526	1	255	255	255
527	98	255	255	255	527	1	255	255	255	527	410	255	255	255
528	8	255	255	255	528	1	255	255	255	528	1	255	255	255
529	1	255	255	255	529	4	255	255	255	529	1	255	255	255
530	1	255	255	255	530	1	255	255	255	530	77	255	255	255
531	1	255	255	255	531	47	255	255	255	531	40	255	255	255
532	1	255	255	255	532	16	255	255	255	532	3	255	255	255
533	39	255	255	255	533	1	255	255	255	533	4	255	255	255

Original data for Figure 2D, Densitometric analysis of Nuclear/Perinuclear B-catenin in control and EoE patient esophageal biopsy tissue.
Values are representative of the mean of a sample size N=18. p<0.05**

534	5	255	255	255	534	131	255	255	255	534	14	255	255	255
535	30	255	255	255	535	1	255	255	255	535	4	255	255	255
536	89	255	255	255	536	1	255	255	255	536	7	255	255	255
537	119	255	255	255	537	1	255	255	255	537	1	255	255	255
538	55	255	255	255	538	3	255	255	255	538	1	255	255	255
539	21	255	255	255	539	1	255	255	255	539	35	255	255	255
540	3	255	255	255	540	1	255	255	255	540	6	255	255	255
541	1	255	255	255	541	1	255	255	255	541	9	255	255	255
542	45	255	255	255	542	4	255	255	255	542	3	255	255	255
543	55	255	255	255	543	1	255	255	255	543	18	255	255	255
544	1	255	255	255	544	2	255	255	255	544	1	255	255	255
545	58	255	255	255	545	3	255	255	255	545	1	255	255	255
546	19	255	255	255	546	1	255	255	255	546	2	255	255	255
547	13	255	255	255	547	17	255	255	255	547	1	255	255	255
548	3	255	255	255	548	2	255	255	255	548	152	255	255	255
549	12	255	255	255	549	6	255	255	255	549	1	255	255	255
550	57	255	255	255	550	1	255	255	255	550	19	255	255	255
551	356	255	255	255	551	1	255	255	255	551	14	255	255	255
552	22	255	255	255	552	141	255	255	255	552	30	255	255	255
553	3	255	255	255	553	1	255	255	255	553	1	255	255	255
554	27	255	255	255	554	1101	255	255	255	554	1	255	255	255
555	85	255	255	255	555	2	255	255	255	555	2	255	255	255
556	5	255	255	255	556	1	255	255	255	556	1	255	255	255
557	1	255	255	255	557	13	255	255	255	557	47	255	255	255
558	1	255	255	255	558	1	255	255	255	558	71	255	255	255
559	1	255	255	255	559	629	255	255	255	559	40	255	255	255
560	1	255	255	255	560	1	255	255	255	560	1	255	255	255
561	104	255	255	255	561	1	255	255	255	561	2	255	255	255
562	103	255	255	255	562	1	255	255	255	562	4	255	255	255
563	1	255	255	255	563	1	255	255	255	563	1	255	255	255
564	4	255	255	255	564	78	255	255	255	564	18	255	255	255
565	1	255	255	255	565	1	255	255	255	565	13	255	255	255
566	252	255	255	255	566	1	255	255	255	566	1	255	255	255
567	86	255	255	255	567	1	255	255	255	567	1	255	255	255
568	2	255	255	255	568	3	255	255	255	568	1	255	255	255
569	1	255	255	255	569	38	255	255	255	569	2	255	255	255
570	5	255	255	255	570	1	255	255	255	570	30	255	255	255
571	32	255	255	255	571	2	255	255	255	571	6	255	255	255
572	2	255	255	255	572	1	255	255	255	572	25	255	255	255
573	19	255	255	255	573	21	255	255	255	573	2	255	255	255
574	118	255	255	255	574	67	255	255	255	574	5	255	255	255
575	108	255	255	255	575	1	255	255	255	575	36	255	255	255
576	1	255	255	255	576	307	255	255	255	576	1	255	255	255
577	156	255	255	255	577	6	255	255	255	577	1	255	255	255
578	1	255	255	255	578	1	255	255	255	578	1	255	255	255
579	2	255	255	255	579	1	255	255	255	579	1	255	255	255
580	1644	255	255	255	580	2	255	255	255	580	3	255	255	255
581	197	255	255	255	581	20	255	255	255	581	2	255	255	255
582	8	255	255	255	582	1	255	255	255	582	188	255	255	255
583	52	255	255	255	583	587	255	255	255	583	1	255	255	255
584	119	255	255	255	584	1	255	255	255	584	1	255	255	255
585	1	255	255	255	585	1	255	255	255	585	13	255	255	255
586	5	255	255	255	586	119	255	255	255	586	1	255	255	255
587	5	255	255	255	587	1	255	255	255	587	8	255	255	255
588	115	255	255	255	588	1	255	255	255	588	8	255	255	255
589	1	255	255	255	589	43	255	255	255	589	2	255	255	255
590	1	255	255	255	590	296	255	255	255	590	1	255	255	255
591	77	255	255	255	591	1	255	255	255	591	1	255	255	255
592	1	255	255	255	592	829	255	255	255	592	1	255	255	255
593	1	255	255	255	593	1	255	255	255	593	1	255	255	255
594	1	255	255	255	594	2	255	255	255	594	42	255	255	255
595	1	255	255	255	595	1	255	255	255	595	34	255	255	255
596	9	255	255	255	596	52	255	255	255	596	1	255	255	255
597	1	255	255	255	597	43	255	255	255	597	23	255	255	255
598	91	255	255	255	598	1	255	255	255	598	2	255	255	255
599	1	255	255	255	599	1	255	255	255	599	1	255	255	255
600	2	255	255	255	600	43	255	255	255	600	12	255	255	255
601	1	255	255	255	601	1	255	255	255	601	1	255	255	255
602	8	255	255	255	602	3	255	255	255	602	14	255	255	255
603	1	255	255	255	603	1	255	255	255	603	1	255	255	255
604	105	255	255	255	604	5	255	255	255	604	1	255	255	255
605	3	255	255	255	605	1	255	255	255	605	38	255	255	255
606	1	255	255	255	606	8	255	255	255	606	2	255	255	255
607	82	255	255	255	607	1	255	255	255	607	1	255	255	255
608	2	255	255	255	608	1	255	255	255	608	59	255	255	255
609	3	255	255	255	609	1	255	255	255	609	42	255	255	255
610	1	255	255	255	610	4	255	255	255	610	9	255	255	255
611	2	255	255	255	611	2	255	255	255	611	3	255	255	255
612	3	255	255	255	612	1	255	255	255	612	7	255	255	255
613	63	255	255	255	613	1	255	255	255	613	68	255	255	255
614	2	255	255	255	614	61	255	255	255	614	1	255	255	255
615	27	255	255	255	615	2	255	255	255	615	61	255	255	255
616	67	255	255	255	616	1	255	255	255	616	37	255	255	255
617	165	255	255	255	617	3	255	255	255	617	1	255	255	255
618	6	255	255	255	618	13	255	255	255	618	5	255	255	255
619	5	255	255	255	619	2	255	255	255	619	5	255	255	255
620	33	255	255	255	620	258	255	255	255	620	12	255	255	255
621	33	255	255	255	621	1	255	255	255	621	3	255	255	255
622	48	255	255	255	622	1	255	255	255	622	61	255	255	255
623	85	255	255	255	623	15	255	255	255	623	17	255	255	255
624	24	255	255	255	624	1	255	255	255	624	2	255	255	255
625	2	255	255	255	625	21	255	255	255	625	1	255	255	255
626	9	255	255	255	626	2	255	255	255	626	1	255	255	255
627	1	255	255	255	627	2	255	255	255	627	35	255	255	255
628	54	255	255	255	628	3	255	255	255	628	1	255	255	255
629	1	255	255	255	629	70	255	255	255	629	1	255	255	255
630	20	255	255	255	630	1	255	255	255	630	1	255	255	255
631	1	255	255	255	631	1	255	255	255	631	1	255	255	255
632	16	255	255	255	632	1	255	255	255	632	2	255	255	255
633	67	255	255	255	633	16	255	255	255	633	1	255	255	255
634	56	255	255	255	634	2	255	255	255	634	1	255	255	255
635	44	255	255	255	635	23	255	255	255	635	1	255	255	255
636	29	255	255	255	636	7	255	255	255	636	8	255	255	255
637	64	255	255	255	637	2	255	255	255	637	222	255	255	255
638	15	255	255	255	638	1	255	255	255	638	2	255	255	255
639	110	255	255	255	639	2	255	255	255	639	2	255	255	255
640	1	255	255	255	640	1	255	255	255	640	1	255	255	255
641	12	255	255	255	641	136	255	255	255	641	1	255	255	255
642	1	255	255	255	642	2	255	255	255	642	157	255	255	255
643	1	255	255	255	643	1	255	255	255	643	3	255	255	255
644	1	255	255	255	644	73	255	255	255	644	8	255	255	255
645	16	255	255	255	645	2	255	255	255	645	6	255	255	255
646	2	255	255	255	646	1	255	255	255	646	1	255	255	255
647	369	255	255	255	647	23	255	255	255	647	22	255	255	255
648	52	255	255	255	648	4	255	255	255	648	15	255	255	255
649	1	255	255	255	649	409	255	255	255	649	2	255	255	255
650	2	255	255	255	650	1	255	255	255	650	11	255	255	255
651	19	255	255	255	651	1	255	255	255	651	3	255	255	255
652	32	255	255	255	652	1	255	255	255	652	37	255	255	255
653	1	255	255	255	653	105	255	255	255	653	1	255	255	255
654	17	255	255	255	654	1	255	255	255	654	2	255	255	255
655	1	255	255	255	655	5	255	255	255	655	46	255	255	255
656	1	255	255	255	656	1	255	255	255	656	3	255	255	255
657	9	255	255	255	657	1	255	255	255	657	1	255	255	255
658	7	255	255	255	658	3	255	255	255	658	95	255	255	255
659	242	255	255	255	659	1	255	255	255	659	1	255	255	255
660	6	255	255	255	660	4	255	255	255	660	6	255	255	255
661	38	255	255	255	661	26	255	255	255	661	1	255	255	255
662	124	255	255	255	662	586	255	255	255	662	3	255	255	255
663	18	255	255	255	663	2	255	255	255	663	1	255	255	255
664	137	255	255	255	664	1	255	255	255	664	3	255	255	255
665	5	255	255	255	665	1	255	255	255	665	1	255	255	255
666	1	255	255	255	666	74	255	255	255	666	1	255	255	255
667	9	255	255	255	667	1	255	255	255	667	2	255	255	255
668	1	255	255	255	668	9	255	255	255	668	6	255	255	255
669	31	255	255	255	669	132	255	255	255	669	27	255	255	255
670	49	255	255	255	670	1	255	255	255	670	59	255	255	255
671	8	255	255	255	671	1	255	255	255	671	1	255	255	255

Original data for Figure 2D, Densitometric analysis of Nuclear/Perinuclear B-catenin in control and EoE patient esophageal biopsy tissue.

Values are representative of the mean of a sample size N=18. p<0.05**

672	107	255	255	255	672	3	255	255	255	672	1	255	255	255
673	12	255	255	255	673	2	255	255	255	673	6	255	255	255
674	172	255	255	255	674	1	255	255	255	674	1	255	255	255
675	171	255	255	255	675	1	255	255	255	675	54	255	255	255
676	45	255	255	255	676	2	255	255	255	676	21	255	255	255
677	5	255	255	255	677	5	255	255	255	677	2	255	255	255
678	60	255	255	255	678	1	255	255	255	678	1	255	255	255
679	1	255	255	255	679	5	255	255	255	679	5	255	255	255
680	13	255	255	255	680	83	255	255	255	680	1	255	255	255
681	2	255	255	255	681	2	255	255	255	681	1	255	255	255
682	5	255	255	255	682	5	255	255	255	682	1	255	255	255
683	23	255	255	255	683	1	255	255	255	683	1	255	255	255
684	1	255	255	255	684	1	255	255	255	684	56	255	255	255
685	101	255	255	255	685	3	255	255	255	685	2	255	255	255
686	20	255	255	255	686	2	255	255	255	686	3	255	255	255
687	1	255	255	255	687	6	255	255	255	687	1	255	255	255
688	2	255	255	255	688	1	255	255	255	688	336	255	255	255
689	17	255	255	255	689	9	255	255	255	689	2	255	255	255
690	1	255	255	255	690	56	255	255	255	690	12	255	255	255
691	21	255	255	255	691	2	255	255	255	691	1	255	255	255
692	1	255	255	255	692	1	255	255	255	692	8	255	255	255
693	48	255	255	255	693	1	255	255	255	693	72	255	255	255
694	6	255	255	255	694	1	255	255	255	694	1	255	255	255
695	88	255	255	255	695	19	255	255	255	695	144	255	255	255
696	1	255	255	255	696	103	255	255	255	696	77	255	255	255
697	68	255	255	255	697	79	255	255	255	697	3	255	255	255
698	231	255	255	255	698	1	255	255	255	698	2	255	255	255
699	1	255	255	255	699	1	255	255	255	699	1	255	255	255
700	3	255	255	255	700	113	255	255	255	700	1	255	255	255
701	1	255	255	255	701	38	255	255	255	701	1	255	255	255
702	8	255	255	255	702	13	255	255	255	702	13	255	255	255
703	48	255	255	255	703	124	255	255	255	703	28	255	255	255
704	7	255	255	255	704	3	255	255	255	704	1	255	255	255
705	1	255	255	255	705	1	255	255	255	705	174	255	255	255
706	1	255	255	255	706	1	255	255	255	706	336	255	255	255
707	21	255	255	255	707	2	255	255	255	707	1	255	255	255
708	385	255	255	255	708	47	255	255	255	708	1	255	255	255
709	1	255	255	255	709	1	255	255	255	709	50	255	255	255
710	2	255	255	255	710	177	255	255	255	710	1	255	255	255
711	28	255	255	255	711	3	255	255	255	711	77	255	255	255
712	7	255	255	255	712	181	255	255	255	712	1	255	255	255
713	8	255	255	255	713	24	255	255	255	713	41	255	255	255
714	5	255	255	255	714	1	255	255	255	714	6	255	255	255
715	1	255	255	255	715	1	255	255	255	715	1	255	255	255
716	61	255	255	255	716	4	255	255	255	716	1	255	255	255
717	3	255	255	255	717	17	255	255	255	717	38	255	255	255
718	2	255	255	255	718	3	255	255	255	718	3	255	255	255
719	1	255	255	255	719	160	255	255	255	719	1	255	255	255
720	1	255	255	255	720	1	255	255	255	720	128	255	255	255
721	1	255	255	255	721	4	255	255	255	721	60	255	255	255
722	9	255	255	255	722	1	255	255	255	722	1	255	255	255
723	14	255	255	255	723	1	255	255	255	723	2	255	255	255
724	1	255	255	255	724	1	255	255	255	724	3	255	255	255
725	1	255	255	255	725	3	255	255	255	725	9	255	255	255
726	1	255	255	255	726	54	255	255	255	726	2	255	255	255
727	28	255	255	255	727	3	255	255	255	727	1	255	255	255
728	1	255	255	255	728	1	255	255	255	728	46	255	255	255
729	3	255	255	255	729	1	255	255	255	729	23	255	255	255
730	3	255	255	255	730	1	255	255	255	730	1	255	255	255
731	1	255	255	255	731	29	255	255	255	731	2	255	255	255
732	191	255	255	255	732	1	255	255	255	732	84	255	255	255
733	1	255	255	255	733	2	255	255	255	733	102	255	255	255
734	1	255	255	255	734	2	255	255	255	734	19	255	255	255
735	39	255	255	255	735	12	255	255	255	735	36	255	255	255
736	1	255	255	255	736	3	255	255	255	736	3	255	255	255
737	1	255	255	255	737	48	255	255	255	737	40	255	255	255
738	1134	255	255	255	738	18	255	255	255	738	1	255	255	255
739	7	255	255	255	739	4	255	255	255	739	35	255	255	255
740	3	255	255	255	740	2	255	255	255	740	1	255	255	255
741	2	255	255	255	741	33	255	255	255	741	2	255	255	255
742	178	255	255	255	742	267	255	255	255	742	1	255	255	255
743	1	255	255	255	743	1	255	255	255	743	6	255	255	255
744	6	255	255	255	744	4	255	255	255	744	91	255	255	255
745	41	255	255	255	745	3	255	255	255	745	1	255	255	255
746	1	255	255	255	746	1	255	255	255	746	1	255	255	255
747	12	255	255	255	747	55	255	255	255	747	2	255	255	255
748	1	255	255	255	748	1	255	255	255	748	1	255	255	255
749	162	255	255	255	749	142	255	255	255	749	40	255	255	255
750	2	255	255	255	750	61	255	255	255	750	30	255	255	255
751	18	255	255	255	751	1	255	255	255	751	1	255	255	255
752	10	255	255	255	752	5	255	255	255	752	1	255	255	255
753	325	255	255	255	753	51	255	255	255	753	12	255	255	255
754	7	255	255	255	754	3	255	255	255	754	1	255	255	255
755	1	255	255	255	755	5	255	255	255	755	2	255	255	255
756	2	255	255	255	756	107	255	255	255	756	2	255	255	255
757	4	255	255	255	757	3	255	255	255	757	2	255	255	255
758	15	255	255	255	758	2	255	255	255	758	20	255	255	255
759	15	255	255	255	759	1	255	255	255	759	36	255	255	255
760	162	255	255	255	760	5	255	255	255	760	225	255	255	255
761	1	255	255	255	761	18	255	255	255	761	5	255	255	255
762	1	255	255	255	762	1	255	255	255	762	2	255	255	255
763	2	255	255	255	763	2	255	255	255	763	43	255	255	255
764	1	255	255	255	764	1	255	255	255	764	7	255	255	255
765	1	255	255	255	765	21	255	255	255	765	79	255	255	255
766	1	255	255	255	766	13	255	255	255	766	2	255	255	255
767	184	255	255	255	767	1	255	255	255	767	4	255	255	255
768	1	255	255	255	768	1	255	255	255	768	58	255	255	255
769	1	255	255	255	769	1	255	255	255	769	93	255	255	255
770	8	255	255	255	770	1	255	255	255	770	1	255	255	255
771	201	255	255	255	771	115	255	255	255	771	9	255	255	255
772	10	255	255	255	772	1	255	255	255	772	3	255	255	255
773	1	255	255	255	773	2	255	255	255	773	8	255	255	255
774	4	255	255	255	774	2	255	255	255	774	12	255	255	255
775	1	255	255	255	775	1	255	255	255	775	61	255	255	255
776	112	255	255	255	776	1	255	255	255	776	2	255	255	255
777	43	255	255	255	777	14	255	255	255	777	2	255	255	255
778	137	255	255	255	778	1	255	255	255	778	36	255	255	255
779	1	255	255	255	779	1	255	255	255	779	5	255	255	255
780	2	255	255	255	780	2	255	255	255	780	1	255	255	255
781	86	255	255	255	781	1	255	255	255	781	2	255	255	255
782	2	255	255	255	782	1	255	255	255	782	1	255	255	255
783	2	255	255	255	783	4	255	255	255	783	1	255	255	255
784	3	255	255	255	784	3	255	255	255	784	44	255	255	255
785	19	255	255	255	785	1	255	255	255	785	1	255	255	255
786	80	255	255	255	786	2	255	255	255	786	37	255	255	255
787	1	255	255	255	787	2	255	255	255	787	5	255	255	255
788	7	255	255	255	788	18	255	255	255	788	2	255	255	255
789	273	255	255	255	789	3	255	255	255	789	2	255	255	255
790	3	255	255	255	790	47	255	255	255	790	11200	255	255	255
791	1	255	255	255	791	3	255	255	255	791	6	255	255	255
792	45	255	255	255	792	37	255	255	255	792	1	255	255	255
793	507	255	255	255	793	1	255	255	255	793	1	255	255	255
794	123	255	255	255	794	2	255	255	255	794	19	255	255	255
795	46	255	255	255	795	6	255	255	255	795	1	255	255	255
796	33	255	255	255	796	1	255	255	255	796	99	255	255	255
797	48	255	255	255	797	58	255	255	255	797	3	255	255	255
798	4	255	255	255	798	1	255	255	255	798	1	255	255	255
799	208	255	255	255	799	1	255	255	255	799	4	255	255	255
800	2	255	255	255	800	3	255	255	255	800	3	255	255	255
801	68	255	255	255	801	90	255	255	255	801	6	255	255	255
802	1	255	255	255	802	5	255	255	255	802	1	255	255	255
803	1	255	255	255	803	1	255	255	255	803	2	255	255	255
804	1	255	255	255	804	2	255	255	255	804	37	255	255	255
805	40	255	255	255	805	144	255	255	255	805	1	255	255	255
806	4	255	255	255	806	2	255	255	255	806	2	255	255	255
807	1	255	255	255	807	132	255	255	255	807	211	255	255	255
808	1	255	255	255	808	1	255	255	255	808	1	255	255	255
809	11	255	255	255	809	45	255	255	255	809	6	255	255	255

Original data for Figure 2D, Densitometric analysis of Nuclear/Perinuclear B-catenin in control and EoE patient esophageal biopsy tissue.
Values are representative of the mean of a sample size N=18. p<0.05**

810	6	255	255	255	810	3	255	255	255	810	11	255	255	255
811	6	255	255	255	811	2	255	255	255	811	3	255	255	255
812	118	255	255	255	812	682	255	255	255	812	15	255	255	255
813	22	255	255	255	813	1	255	255	255	813	1	255	255	255
814	3	255	255	255	814	1333	255	255	255	814	54	255	255	255
815	1	255	255	255	815	1	255	255	255	815	68	255	255	255
816	2	255	255	255	816	1	255	255	255	816	6	255	255	255
817	30	255	255	255	817	105	255	255	255	817	1	255	255	255
818	25	255	255	255	818	1	255	255	255	818	19	255	255	255
819	40	255	255	255	819	62	255	255	255	819	1	255	255	255
820	30	255	255	255	820	53	255	255	255	820	49	255	255	255
821	9	255	255	255	821	1	255	255	255	821	1	255	255	255
822	2	255	255	255	822	1	255	255	255	822	2	255	255	255
823	93	255	255	255	823	62	255	255	255	823	30	255	255	255
824	1	255	255	255	824	1	255	255	255	824	2	255	255	255
825	1	255	255	255	825	2	255	255	255	825	63	255	255	255
826	3	255	255	255	826	1	255	255	255	826	102	255	255	255
827	281	255	255	255	827	1	255	255	255	827	14	255	255	255
828	200	255	255	255	828	4	255	255	255	828	2	255	255	255
829	90	255	255	255	829	1	255	255	255	829	3	255	255	255
830	1	255	255	255	830	3	255	255	255	830	1	255	255	255
831	1	255	255	255	831	37	255	255	255	831	26	255	255	255
832	1	255	255	255	832	3	255	255	255	832	2	255	255	255
833	2	255	255	255	833	39	255	255	255	833	53	255	255	255
834	88	255	255	255	834	2	255	255	255	834	19	255	255	255
835	1	255	255	255	835	1	255	255	255	835	1	255	255	255
836	1	255	255	255	836	1	255	255	255	836	26	255	255	255
837	4	255	255	255	837	1	255	255	255	837	92	255	255	255
838	1	255	255	255	838	1	255	255	255	838	17	255	255	255
839	57	255	255	255	839	1196	255	255	255	839	1	255	255	255
840	121	255	255	255	840	4	255	255	255	840	61	255	255	255
841	1	255	255	255	841	1	255	255	255	841	17	255	255	255
842	34	255	255	255	842	3	255	255	255	842	2	255	255	255
843	4	255	255	255	843	763	255	255	255	843	36	255	255	255
844	1	255	255	255	844	1	255	255	255	844	12	255	255	255
845	250	255	255	255	845	2	255	255	255	845	6	255	255	255
846	2	255	255	255	846	1	255	255	255	846	1	255	255	255
847	1	255	255	255	847	1	255	255	255	847	34	255	255	255
848	77	255	255	255	848	1	255	255	255	848	2	255	255	255
849	32	255	255	255	849	1	255	255	255	849	2	255	255	255
850	4	255	255	255	850	1	255	255	255	850	64	255	255	255
851	2	255	255	255	851	1	255	255	255	851	1	255	255	255
852	48	255	255	255	852	3	255	255	255	852	1	255	255	255
853	1	255	255	255	853	1	255	255	255	853	7	255	255	255
854	1	255	255	255	854	61	255	255	255	854	1	255	255	255
855	2	255	255	255	855	7	255	255	255	855	18	255	255	255
856	1	255	255	255	856	4	255	255	255	856	1	255	255	255
857	1	255	255	255	857	1	255	255	255	857	5	255	255	255
858	1	255	255	255	858	157	255	255	255	858	2	255	255	255
859	72	255	255	255	859	840	255	255	255	859	1	255	255	255
860	1	255	255	255	860	1	255	255	255	860	1	255	255	255
861	7	255	255	255	861	1	255	255	255	861	76	255	255	255
862	1	255	255	255	862	1	255	255	255	862	2	255	255	255
863	9	255	255	255	863	1	255	255	255	863	1	255	255	255
864	1	255	255	255	864	259	255	255	255	864	2	255	255	255
865	69	255	255	255	865	3	255	255	255	865	1	255	255	255
866	4	255	255	255	866	2	255	255	255	866	108	255	255	255
867	1	255	255	255	867	1	255	255	255	867	25	255	255	255
868	1	255	255	255	868	91	255	255	255	868	1	255	255	255
869	1	255	255	255	869	40	255	255	255	869	49	255	255	255
870	210	255	255	255	870	1	255	255	255	870	50	255	255	255
871	1	255	255	255	871	3	255	255	255	871	14	255	255	255
872	48	255	255	255	872	19	255	255	255	872	2	255	255	255
873	40	255	255	255	873	23	255	255	255	873	28	255	255	255
874	2	255	255	255	874	1336	255	255	255	874	6	255	255	255
875	58	255	255	255	875	2	255	255	255	875	60	255	255	255
876	1	255	255	255	876	1	255	255	255	876	59	255	255	255
877	1	255	255	255	877	35	255	255	255	877	2	255	255	255
878	5	255	255	255	878	5	255	255	255	878	3	255	255	255
879	1	255	255	255	879	1	255	255	255	879	1	255	255	255
880	1	255	255	255	880	2	255	255	255	880	15	255	255	255
881	3	255	255	255	881	7	255	255	255	881	2	255	255	255
882	1	255	255	255	882	2	255	255	255	882	3	255	255	255
883	1	255	255	255	883	37	255	255	255	883	133	255	255	255
884	2	255	255	255	884	139	255	255	255	884	2	255	255	255
885	6	255	255	255	885	1	255	255	255	885	1	255	255	255
886	2	255	255	255	886	1	255	255	255	886	1	255	255	255
887	7	255	255	255	887	1	255	255	255	887	20	255	255	255
888	1	255	255	255	888	1	255	255	255	888	23	255	255	255
889	2	255	255	255	889	28	255	255	255	889	2	255	255	255
890	425	255	255	255	890	1	255	255	255	890	2	255	255	255
891	4	255	255	255	891	1	255	255	255	891	1	255	255	255
892	1	255	255	255	892	2	255	255	255	892	1	255	255	255
893	3	255	255	255	893	1	255	255	255	893	1	255	255	255
894	188	255	255	255	894	1	255	255	255	894	1	255	255	255
895	12	255	255	255	895	37	255	255	255	895	1	255	255	255
896	1	255	255	255	896	10	255	255	255	896	1	255	255	255
897	30	255	255	255	897	56	255	255	255	897	2	255	255	255
898	95	255	255	255	898	101	255	255	255	898	142	255	255	255
899	2	255	255	255	899	19	255	255	255	899	1	255	255	255
900	149	255	255	255	900	24	255	255	255	900	1	255	255	255
901	24	255	255	255	901	1	255	255	255	901	9	255	255	255
902	82	255	255	255	902	65	255	255	255	902	1	255	255	255
903	36	255	255	255	903	1	255	255	255	903	6	255	255	255
904	1	255	255	255	904	1	255	255	255	904	1	255	255	255
905	12	255	255	255	905	9	255	255	255	905	10	255	255	255
906	1	255	255	255	906	3	255	255	255	906	1	255	255	255
907	16	255	255	255	907	1	255	255	255	907	56	255	255	255
908	25	255	255	255	908	1	255	255	255	908	2	255	255	255
909	22	255	255	255	909	2	255	255	255	909	1	255	255	255
910	2	255	255	255	910	5	255	255	255	910	1	255	255	255
911	1	255	255	255	911	1	255	255	255	911	1	255	255	255
912	28	255	255	255	912	1	255	255	255	912	1	255	255	255
913	1	255	255	255	913	4	255	255	255	913	2	255	255	255
914	17	255	255	255	914	179	255	255	255	914	2	255	255	255
915	35	255	255	255	915	1	255	255	255	915	1	255	255	255
916	3	255	255	255	916	124	255	255	255	916	1	255	255	255
917	1	255	255	255	917	1	255	255	255	917	42	255	255	255
918	1	255	255	255	918	5	255	255	255	918	1	255	255	255
919	1	255	255	255	919	2	255	255	255	919	3	255	255	255
920	1	255	255	255	920	4	255	255	255	920	1	255	255	255
921	8	255	255	255	921	4	255	255	255	921	1	255	255	255
922	1	255	255	255	922	1	255	255	255	922	1	255	255	255
923	11	255	255	255	923	151	255	255	255	923	2	255	255	255
924	74	255	255	255	924	6	255	255	255	924	1	255	255	255
925	4	255	255	255	925	4	255	255	255	925	5	255	255	255
926	145	255	255	255	926	1	255	255	255	926	1	255	255	255
927	48	255	255	255	927	12	255	255	255	927	5	255	255	255
928	6	255	255	255	928	4	255	255	255	928	1	255	255	255
929	1	255	255	255	929	323	255	255	255	929	1	255	255	255
930	54	255	255	255	930	840	255	255	255	930	1	255	255	255
931	107	255	255	255	931	64	255	255	255	931	18	255	255	255
932	131	255	255	255	932	1	255	255	255	932	1	255	255	255
933	1	255	255	255	933	1	255	255	255	933	2	255	255	255
934	219	255	255	255	934	2	255	255	255	934	2	255	255	255
935	2	255	255	255	935	1	255	255	255	935	19	255	255	255
936	30	255	255	255	936	1	255	255	255	936	36	255	255	255
937	19	255	255	255	937	3	255	255	255	937	1	255	255	255
938	110	255	255	255	938	2	255	255	255	938	5	255	255	255
939	1	255	255	255	939	1	255	255	255	939	29	255	255	255
940	196	255	255	255	940	2	255	255	255	940	1	255	255	255
941	2	255	255	255	941	2	255	255	255	941	20	255	255	255
942	15	255	255	255	942	4	255	255	255	942	1	255	255	255
943	8	255	255	255	943	2	255	255	255	943	19	255	255	255
944	1	255	255	255	944	2	255	255	255	944	12	255	255	255
945	26	255	255	255	945	1	255	255	255	945	27	255	255	255
946	11	255	255	255	946	1	255	255	255	946	1	255	255	255
947	2	255	255	255	947	48	255	255	255	947	2	255	255	255

Original data for Figure 2D, Densitometric analysis of Nuclear/Perinuclear B-catenin in control and EoE patient esophageal biopsy tissue.

Values are representative of the mean of a sample size N=18. p<0.05**

948	2	255	255	255	948	2	255	255	255	948	1	255	255	255
949	1	255	255	255	949	43	255	255	255	949	47	255	255	255
950	1	255	255	255	950	1	255	255	255	950	8	255	255	255
951	1	255	255	255	951	1	255	255	255	951	1	255	255	255
952	160	255	255	255	952	1	255	255	255	952	64	255	255	255
953	145	255	255	255	953	55	255	255	255	953	5	255	255	255
954	4	255	255	255	954	1	255	255	255	954	2	255	255	255
955	22	255	255	255	955	24	255	255	255	955	2	255	255	255
956	62	255	255	255	956	1	255	255	255	956	1	255	255	255
957	701	255	255	255	957	2	255	255	255	957	101	255	255	255
958	1	255	255	255	958	1	255	255	255	958	2	255	255	255
959	5	255	255	255	959	2	255	255	255	959	4	255	255	255
960	34	255	255	255	960	221	255	255	255	960	1	255	255	255
961	1	255	255	255	961	1	255	255	255	961	29	255	255	255
962	63	255	255	255	962	1	255	255	255	962	92	255	255	255
963	40	255	255	255	963	3	255	255	255	963	76	255	255	255
964	3	255	255	255	964	1	255	255	255	964	102	255	255	255
965	3	255	255	255	965	1	255	255	255	965	7	255	255	255
966	1	255	255	255	966	1	255	255	255	966	2	255	255	255
967	21	255	255	255	967	10	255	255	255	967	2	255	255	255
968	5	255	255	255	968	11	255	255	255	968	19	255	255	255
969	1	255	255	255	969	18	255	255	255	969	4	255	255	255
970	1946	255	255	255	970	52	255	255	255	970	2	255	255	255
971	37	255	255	255	971	5	255	255	255	971	5	255	255	255
972	1	255	255	255	972	1	255	255	255	972	1	255	255	255
973	1	255	255	255	973	1	255	255	255	973	1	255	255	255
974	6	255	255	255	974	3	255	255	255	974	7	255	255	255
975	1	255	255	255	975	4	255	255	255	975	2	255	255	255
976	5	255	255	255	976	559	255	255	255	976	1	255	255	255
977	30	255	255	255	977	26	255	255	255	977	11	255	255	255
978	3	255	255	255	978	4	255	255	255	978	2	255	255	255
979	80	255	255	255	979	48	255	255	255	979	22	255	255	255
980	2	255	255	255	980	38	255	255	255	980	13	255	255	255
981	4	255	255	255	981	1	255	255	255	981	65	255	255	255
982	1	255	255	255	982	1	255	255	255	982	72	255	255	255
983	29	255	255	255	983	2	255	255	255	983	101	255	255	255
984	14	255	255	255	984	1	255	255	255	984	1	255	255	255
985	1	255	255	255	985	1	255	255	255	985	13	255	255	255
986	112	255	255	255	986	32	255	255	255	986	1	255	255	255
987	2	255	255	255	987	29	255	255	255	987	6	255	255	255
988	1	255	255	255	988	1	255	255	255	988	1	255	255	255
989	3	255	255	255	989	1	255	255	255	989	3	255	255	255
990	7	255	255	255	990	42	255	255	255	990	9	255	255	255
991	87	255	255	255	991	1	255	255	255	991	1	255	255	255
992	2	255	255	255	992	2	255	255	255	992	4	255	255	255
993	11	255	255	255	993	1	255	255	255	993	4	255	255	255
994	4	255	255	255	994	1	255	255	255	994	1	255	255	255
995	1	255	255	255	995	7	255	255	255	995	78	255	255	255
996	1	255	255	255	996	3	255	255	255	996	47	255	255	255
997	5	255	255	255	997	2	255	255	255	997	31	255	255	255
998	22	255	255	255	998	3	255	255	255	998	8	255	255	255
999	1	255	255	255	999	1	255	255	255	999	5	255	255	255
1000	104	255	255	255	1000	1	255	255	255	1000	9	255	255	255
1001	1	255	255	255	1001	2	255	255	255	1001	88	255	255	255
1002	1	255	255	255	1002	1	255	255	255	1002	1	255	255	255
1003	1	255	255	255	1003	2	255	255	255	1003	1	255	255	255
1004	9	255	255	255	1004	178	255	255	255	1004	4	255	255	255
1005	2	255	255	255	1005	192	255	255	255	1005	52	255	255	255
1006	1	255	255	255	1006	1	255	255	255	1006	42	255	255	255
1007	22	255	255	255	1007	2	255	255	255	1007	1	255	255	255
1008	30	255	255	255	1008	4	255	255	255	1008	1	255	255	255
1009	3	255	255	255	1009	2	255	255	255	1009	4	255	255	255
1010	57	255	255	255	1010	1	255	255	255	1010	18	255	255	255
1011	14	255	255	255	1011	2	255	255	255	1011	9	255	255	255
1012	2	255	255	255	1012	5	255	255	255	1012	4	255	255	255
1013	2	255	255	255	1013	65	255	255	255	1013	1	255	255	255
1014	43	255	255	255	1014	37	255	255	255	1014	6	255	255	255
1015	4	255	255	255	1015	3	255	255	255	1015	228	255	255	255
1016	7	255	255	255	1016	124	255	255	255	1016	2	255	255	255
1017	1	255	255	255	1017	103	255	255	255	1017	1	255	255	255
1018	2	255	255	255	1018	1	255	255	255	1018	45	255	255	255
1019	1	255	255	255	1019	4	255	255	255	1019	1	255	255	255
1020	27	255	255	255	1020	1	255	255	255	1020	1	255	255	255
1021	2	255	255	255	1021	5	255	255	255	1021	1	255	255	255
1022	32	255	255	255	1022	3	255	255	255	1022	2	255	255	255
1023	4	255	255	255	1023	1	255	255	255	1023	28	255	255	255
1024	1	255	255	255	1024	1	255	255	255	1024	3	255	255	255
1025	1	255	255	255	1025	5	255	255	255	1025	3	255	255	255
1026	2	255	255	255	1026	19	255	255	255	1026	7	255	255	255
1027	43	255	255	255	1027	10	255	255	255	1027	1	255	255	255
1028	25	255	255	255	1028	1	255	255	255	1028	1494	255	255	255
1029	75	255	255	255	1029	525	255	255	255	1029	8	255	255	255
1030	1	255	255	255	1030	13	255	255	255	1030	1	255	255	255
1031	3	255	255	255	1031	2	255	255	255	1031	2	255	255	255
1032	7	255	255	255	1032	22	255	255	255	1032	51	255	255	255
1033	29	255	255	255	1033	2	255	255	255	1033	109	255	255	255
1034	2	255	255	255	1034	24	255	255	255	1034	1	255	255	255
1035	92	255	255	255	1035	1	255	255	255	1035	2	255	255	255
1036	34	255	255	255	1036	1	255	255	255	1036	32	255	255	255
1037	1	255	255	255	1037	3	255	255	255	1037	1	255	255	255
1038	77	255	255	255	1038	3	255	255	255	1038	1	255	255	255
1039	26	255	255	255	1039	73	255	255	255	1039	114	255	255	255
1040	2	255	255	255	1040	4	255	255	255	1040	1	255	255	255
1041	26	255	255	255	1041	10	255	255	255	1041	2	255	255	255
1042	3	255	255	255	1042	1	255	255	255	1042	25	255	255	255
1043	3	255	255	255	1043	6	255	255	255	1043	9	255	255	255
1044	1	255	255	255	1044	68	255	255	255	1044	1	255	255	255
1045	5	255	255	255	1045	9	255	255	255	1045	2	255	255	255
1046	1	255	255	255	1046	1	255	255	255	1046	1	255	255	255
1047	180	255	255	255	1047	2	255	255	255	1047	4	255	255	255
1048	4	255	255	255	1048	64	255	255	255	1048	1	255	255	255
1049	2	255	255	255	1049	5	255	255	255	1049	2	255	255	255
1050	1	255	255	255	1050	4	255	255	255	1050	6	255	255	255
1051	41	255	255	255	1051	1	255	255	255	1051	3	255	255	255
1052	1	255	255	255	1052	60	255	255	255	1052	59	255	255	255
1053	46	255	255	255	1053	1	255	255	255	1053	17	255	255	255
1054	1	255	255	255	1054	5	255	255	255	1054	37	255	255	255
1055	24	255	255	255	1055	1	255	255	255	1055	1	255	255	255
1056	130	255	255	255	1056	1	255	255	255	1056	2	255	255	255
1057	39	255	255	255	1057	1349	255	255	255	1057	9	255	255	255
1058	14	255	255	255	1058	1	255	255	255	1058	19	255	255	255
1059	5	255	255	255	1059	6	255	255	255	1059	2	255	255	255
1060	48	255	255	255	1060	25	255	255	255	1060	4	255	255	255
1061	65	255	255	255	1061	24	255	255	255	1061	1	255	255	255
1062	37	255	255	255	1062	1	255	255	255	1062	7	255	255	255
1063	7	255	255	255	1063	83	255	255	255	1063	4	255	255	255
1064	5	255	255	255	1064	450	255	255	255	1064	2	255	255	255
1065	2	255	255	255	1065	1	255	255	255	1065	14	255	255	255
1066	30	255	255	255	1066	82	255	255	255	1066	4	255	255	255
1067	15	255	255	255	1067	1	255	255	255	1067	1	255	255	255
1068	47	255	255	255	1068	3	255	255	255	1068	2	255	255	255
1069	158	255	255	255	1069	3	255	255	255	1069	17	255	255	255
1070	1	255	255	255	1070	1	255	255	255	1070	15	255	255	255
1071	11	255	255	255	1071	37	255	255	255	1071	9	255	255	255
1072	1	255	255	255	1072	1	255	255	255	1072	1	255	255	255
1073	1	255	255	255	1073	3	255	255	255	1073	8	255	255	255
1074	226	255	255	255	1074	17	255	255	255	1074	4	255	255	255
1075	4	255	255	255	1075	1	255	255	255	1075	28	255	255	255
1076	2	255	255	255	1076	4	255	255	255	1076	5	255	255	255
1077	25	255	255	255	1077	1	255	255	255	1077	1	255	255	255
1078	1	255	255	255	1078	1	255	255	255	1078	11	255	255	255
1079	5	255	255	255	1079	2	255	255	255	1079	2	255	255	255
1080	18	255	255	255	1080	1	255	255	255	1080	170	255	255	255
1081	2	255	255	255	1081	145	255	255	255	1081	1	255	255	255
1082	40	255	255	255	1082	1	255	255	255	1082	1	255	255	255
1083	5	255	255	255	1083	22	255	255	255	1083	1	255	255	255
1084	8	255	255	255	1084	1	255	255	255	1084	118	255	255	255
1085	48	255	255	255	1085	3	255	255	255	1085	9	255	255	255

Original data for Figure 2D, Densitometric analysis of Nuclear/Perinuclear B-catenin in control and EoE patient esophageal biopsy tissue.

Values are representative of the mean of a sample size N=18. p<0.05**

1086	1	255	255	255	1086	1	255	255	255	1086	45	255	255	255
1087	28	255	255	255	1087	2	255	255	255	1087	2	255	255	255
1088	4	255	255	255	1088	4	255	255	255	1088	2	255	255	255
1089	360	255	255	255	1089	111	255	255	255	1089	418	255	255	255
1090	2	255	255	255	1090	2	255	255	255	1090	2	255	255	255
1091	1	255	255	255	1091	1	255	255	255	1091	2	255	255	255
1092	1	255	255	255	1092	3	255	255	255	1092	2	255	255	255
1093	1	255	255	255	1093	4	255	255	255	1093	230	255	255	255
1094	71	255	255	255	1094	439	255	255	255	1094	2	255	255	255
1095	1	255	255	255	1095	410	255	255	255	1095	1	255	255	255
1096	2	255	255	255	1096	1	255	255	255	1096	5	255	255	255
1097	4	255	255	255	1097	3	255	255	255	1097	6	255	255	255
1098	2	255	255	255	1098	2	255	255	255	1098	6	255	255	255
1099	5	255	255	255	1099	1	255	255	255	1099	1	255	255	255
1100	342	255	255	255	1100	2	255	255	255	1100	1	255	255	255
1101	1	255	255	255	1101	106	255	255	255	1101	2	255	255	255
1102	1	255	255	255	1102	152	255	255	255	1102	65	255	255	255
1103	1	255	255	255	1103	15	255	255	255	1103	287	255	255	255
1104	227	255	255	255	1104	16	255	255	255	1104	1	255	255	255
1105	55	255	255	255	1105	1	255	255	255	1105	2	255	255	255
1106	2	255	255	255	1106	166	255	255	255	1106	2	255	255	255
1107	2	255	255	255	1107	75	255	255	255	1107	1	255	255	255
1108	2	255	255	255	1108	1	255	255	255	1108	2	255	255	255
1109	1	255	255	255	1109	3	255	255	255	1109	6	255	255	255
1110	87	255	255	255	1110	1	255	255	255	1110	8	255	255	255
1111	1	255	255	255	1111	1	255	255	255	1111	13	255	255	255
1112	4	255	255	255	1112	1	255	255	255	1112	119	255	255	255
1113	74	255	255	255	1113	1	255	255	255	1113	23	255	255	255
1114	18	255	255	255	1114	3	255	255	255	1114	1	255	255	255
1115	1	255	255	255	1115	2	255	255	255	1115	41	255	255	255
1116	64	255	255	255	1116	3	255	255	255	1116	9	255	255	255
1117	87	255	255	255	1117	2	255	255	255	1117	1	255	255	255
1118	7	255	255	255	1118	1	255	255	255	1118	6	255	255	255
1119	26	255	255	255	1119	1049	255	255	255	1119	2	255	255	255
1120	2	255	255	255	1120	399	255	255	255	1120	19	255	255	255
1121	51	255	255	255	1121	2	255	255	255	1121	1	255	255	255
1122	2	255	255	255	1122	201	255	255	255	1122	16	255	255	255
1123	2	255	255	255	1123	1	255	255	255	1123	1	255	255	255
1124	406	255	255	255	1124	68	255	255	255	1124	7	255	255	255
1125	1	255	255	255	1125	1	255	255	255	1125	5	255	255	255
1126	19	255	255	255	1126	2	255	255	255	1126	1	255	255	255
1127	125	255	255	255	1127	14	255	255	255	1127	1	255	255	255
1128	90	255	255	255	1128	17	255	255	255	1128	1	255	255	255
1129	1	255	255	255	1129	118	255	255	255	1129	1	255	255	255
1130	1	255	255	255	1130	1	255	255	255	1130	3	255	255	255
1131	1	255	255	255	1131	3	255	255	255	1131	3	255	255	255
1132	1	255	255	255	1132	2	255	255	255	1132	1	255	255	255
1133	33	255	255	255	1133	2	255	255	255	1133	1	255	255	255
1134	7	255	255	255	1134	1096	255	255	255	1134	9	255	255	255
1135	2	255	255	255	1135	8	255	255	255	1135	44	255	255	255
1136	3	255	255	255	1136	1	255	255	255	1136	85	255	255	255
1137	1	255	255	255	1137	1	255	255	255	1137	1	255	255	255
1138	1	255	255	255	1138	1	255	255	255	1138	5	255	255	255
1139	1	255	255	255	1139	1	255	255	255	1139	4	255	255	255
1140	1	255	255	255	1140	108	255	255	255	1140	1	255	255	255
1141	135	255	255	255	1141	1	255	255	255	1141	1	255	255	255
1142	14	255	255	255	1142	4	255	255	255	1142	2	255	255	255
1143	1	255	255	255	1143	37	255	255	255	1143	22	255	255	255
1144	36	255	255	255	1144	1	255	255	255	1144	17	255	255	255
1145	2	255	255	255	1145	4	255	255	255	1145	18	255	255	255
1146	32	255	255	255	1146	7	255	255	255	1146	3	255	255	255
1147	257	255	255	255	1147	1	255	255	255	1147	1	255	255	255
1148	3	255	255	255	1148	280	255	255	255	1148	100	255	255	255
1149	8	255	255	255	1149	1	255	255	255	1149	35	255	255	255
1150	64	255	255	255	1150	1	255	255	255	1150	1	255	255	255
1151	1	255	255	255	1151	1	255	255	255	1151	1	255	255	255
1152	94	255	255	255	1152	3	255	255	255	1152	1	255	255	255
1153	10	255	255	255	1153	3	255	255	255	1153	1	255	255	255
1154	13	255	255	255	1154	129	255	255	255	1154	7	255	255	255
1155	1	255	255	255	1155	1	255	255	255	1155	1	255	255	255
1156	343	255	255	255	1156	1	255	255	255	1156	48	255	255	255
1157	1	255	255	255	1157	3	255	255	255	1157	41	255	255	255
1158	4	255	255	255	1158	3	255	255	255	1158	7	255	255	255
1159	1	255	255	255	1159	1	255	255	255	1159	81	255	255	255
1160	124	255	255	255	1160	1	255	255	255	1160	83	255	255	255
1161	1	255	255	255	1161	3	255	255	255	1161	7	255	255	255
1162	52	255	255	255	1162	1	255	255	255	1162	18	255	255	255
1163	955	255	255	255	1163	1	255	255	255	1163	2	255	255	255
1164	160	255	255	255	1164	5	255	255	255	1164	1	255	255	255
1165	10	255	255	255	1165	4	255	255	255	1165	1	255	255	255
1166	2	255	255	255	1166	24	255	255	255	1166	2	255	255	255
1167	46	255	255	255	1167	1	255	255	255	1167	1	255	255	255
1168	50	255	255	255	1168	1	255	255	255	1168	45	255	255	255
1169	2	255	255	255	1169	1	255	255	255	1169	47	255	255	255
1170	1	255	255	255	1170	12	255	255	255	1170	2	255	255	255
1171	7	255	255	255	1171	2	255	255	255	1171	72	255	255	255
1172	1	255	255	255	1172	7	255	255	255	1172	71	255	255	255
1173	71	255	255	255	1173	1	255	255	255	1173	4	255	255	255
1174	1	255	255	255	1174	1	255	255	255	1174	1	255	255	255
1175	10	255	255	255	1175	2	255	255	255	1175	1	255	255	255
1176	9	255	255	255	1176	1	255	255	255	1176	1	255	255	255
1177	1	255	255	255	1177	13	255	255	255	1177	1	255	255	255
1178	804	255	255	255	1178	1	255	255	255	1178	157	255	255	255
1179	12	255	255	255	1179	2	255	255	255	1179	1	255	255	255
1180	51	255	255	255	1180	3	255	255	255	1180	3	255	255	255
1181	2	255	255	255	1181	25	255	255	255	1181	7	255	255	255
1182	6	255	255	255	1182	1	255	255	255	1182	2	255	255	255
1183	1	255	255	255	1183	3	255	255	255	1183	1	255	255	255
1184	1	255	255	255	1184	1	255	255	255	1184	11	255	255	255
1185	44	255	255	255	1185	1	255	255	255	1185	101	255	255	255
1186	1	255	255	255	1186	3	255	255	255	1186	84	255	255	255
1187	2	255	255	255	1187	3	255	255	255	1187	1	255	255	255
1188	1	255	255	255	1188	20	255	255	255	1188	1	255	255	255
1189	36	255	255	255	1189	3848	255	255	255	1189	12	255	255	255
1190	1	255	255	255	1190	1	255	255	255	1190	2	255	255	255
1191	16	255	255	255	1191	1	255	255	255	1191	2	255	255	255
1192	40	255	255	255	1192	1	255	255	255	1192	1	255	255	255
1193	3	255	255	255	1193	3	255	255	255	1193	73	255	255	255
1194	12	255	255	255	1194	338	255	255	255	1194	1	255	255	255
1195	2	255	255	255	1195	3	255	255	255	1195	1	255	255	255
1196	362	255	255	255	1196	1	255	255	255	1196	16	255	255	255
1197	1	255	255	255	1197	495	255	255	255	1197	1	255	255	255
1198	58	255	255	255	1198	15	255	255	255	1198	1	255	255	255
1199	133	255	255	255	1199	2	255	255	255	1199	73	255	255	255
1200	16	255	255	255	1200	1	255	255	255	1200	1	255	255	255
1201	1	255	255	255	1201	1	255	255	255	1201	7	255	255	255
1202	1	255	255	255	1202	82	255	255	255	1202	2	255	255	255
1203	9	255	255	255	1203	2	255	255	255	1203	1	255	255	255
1204	6	255	255	255	1204	1	255	255	255	1204	30	255	255	255
1205	2	255	255	255	1205	69	255	255	255	1205	1	255	255	255
1206	24	255	255	255	1206	24	255	255	255	1206	41	255	255	255
1207	3	255	255	255	1207	47	255	255	255	1207	6	255	255	255
1208	1	255	255	255	1208	33	255	255	255	1208	9	255	255	255
1209	95	255	255	255	1209	49	255	255	255	1209	18	255	255	255
1210	47	255	255	255	1210	1	255	255	255	1210	7	255	255	255
1211	24	255	255	255	1211	40	255	255	255	1211	24	255	255	255
1212	2	255	255	255	1212	463	255	255	255	1212	32	255	255	255
1213	26	255	255	255	1213	23	255	255	255	1213	29	255	255	255
1214	12	255	255	255	1214	52	255	255	255	1214	2	255	255	255
1215	18	255	255	255	1215	3	255	255	255	1215	1	255	255	255
1216	262	255	255	255	1216	1	255	255	255	1216	1	255	255	255
1217	129	255	255	255	1217	1	255	255	255	1217	2	255	255	255
1218	2	255	255	255	1218	1	255	255	255	1218	2	255	255	255
1219	1	255	255	255	1219	1	255	255	255	1219	42	255	255	255
1220	2	255	255	255	1220	47	255	255	255	1220	7	255	255	255
1221	110	255	255	255	1221	2	255	255	255	1221	11	255	255	255
1222	1	255	255	255	1222	1	255	255	255	1222	34	255	255	255
1223	1	255	255	255	1223	144	255	255	255	1223	21	255	255	255

Original data for Figure 2D, Densitometric analysis of Nuclear/Perinuclear B-catenin in control and EoE patient esophageal biopsy tissue.

Values are representative of the mean of a sample size N=18. p<0.05**

1224	2	255	255	255	1224	2	255	255	255	1224	10	255	255	255
1225	1	255	255	255	1225	1	255	255	255	1225	52	255	255	255
1226	1	255	255	255	1226	1	255	255	255	1226	8	255	255	255
1227	16	255	255	255	1227	1	255	255	255	1227	6	255	255	255
1228	5	255	255	255	1228	1	255	255	255	1228	3	255	255	255
1229	4	255	255	255	1229	2	255	255	255	1229	9	255	255	255
1230	10	255	255	255	1230	90	255	255	255	1230	1	255	255	255
1231	2	255	255	255	1231	4	255	255	255	1231	1	255	255	255
1232	374	255	255	255	1232	2	255	255	255	1232	93	255	255	255
1233	3	255	255	255	1233	4	255	255	255	1233	41	255	255	255
1234	1	255	255	255	1234	1	255	255	255	1234	619	255	255	255
1235	8	255	255	255	1235	2	255	255	255	1235	1	255	255	255
1236	4	255	255	255	1236	1	255	255	255	1236	411	255	255	255
1237	35	255	255	255	1237	10	255	255	255	1237	8	255	255	255
1238	8	255	255	255	1238	27	255	255	255	1238	29	255	255	255
1239	7	255	255	255	1239	26	255	255	255	1239	93	255	255	255
1240	1	255	255	255	1240	1	255	255	255	1240	1103	255	255	255
1241	1	255	255	255	1241	27	255	255	255	1241	10	255	255	255
1242	117	255	255	255	1242	1	255	255	255	1242	1	255	255	255
1243	122	255	255	255	1243	20	255	255	255	1243	1	255	255	255
1244	1	255	255	255	1244	1	255	255	255	1244	2	255	255	255
1245	2	255	255	255	1245	13	255	255	255	1245	3	255	255	255
1246	1	255	255	255	1246	202	255	255	255	1246	1	255	255	255
1247	5	255	255	255	1247	1	255	255	255	1247	4	255	255	255
1248	22	255	255	255	1248	1	255	255	255	1248	50	255	255	255
1249	1	255	255	255	1249	43	255	255	255	1249	1	255	255	255
1250	1	255	255	255	1250	2	255	255	255	1250	1	255	255	255
1251	1	255	255	255	1251	5	255	255	255	1251	32	255	255	255
1252	118	255	255	255	1252	1	255	255	255	1252	4	255	255	255
1253	1	255	255	255	1253	6	255	255	255	1253	48	255	255	255
1254	1	255	255	255	1254	1	255	255	255	1254	4	255	255	255
1255	19	255	255	255	1255	1	255	255	255	1255	27	255	255	255
1256	62	255	255	255	1256	9	255	255	255	1256	1	255	255	255
1257	59	255	255	255	1257	1	255	255	255	1257	3	255	255	255
1258	63	255	255	255	1258	1	255	255	255	1258	4	255	255	255
1259	1	255	255	255	1259	1	255	255	255	1259	13	255	255	255
1260	3	255	255	255	1260	2	255	255	255	1260	5	255	255	255
1261	1	255	255	255	1261	3	255	255	255	1261	2	255	255	255
1262	8	255	255	255	1262	34	255	255	255	1262	2	255	255	255
1263	1	255	255	255	1263	31	255	255	255	1263	73	255	255	255
1264	2	255	255	255	1264	2	255	255	255	1264	43	255	255	255
1265	5	255	255	255	1265	1	255	255	255	1265	1	255	255	255
1266	162	255	255	255	1266	195	255	255	255	1266	1	255	255	255
1267	75	255	255	255	1267	84	255	255	255	1267	1	255	255	255
1268	91	255	255	255	1268	2	255	255	255	1268	1	255	255	255
1269	1	255	255	255	1269	5	255	255	255	1269	1	255	255	255
1270	34	255	255	255	1270	15	255	255	255	1270	6	255	255	255
1271	4	255	255	255	1271	1	255	255	255	1271	423	255	255	255
1272	61	255	255	255	1272	1	255	255	255	1272	1	255	255	255
1273	1	255	255	255	1273	37	255	255	255	1273	45	255	255	255
1274	1	255	255	255	1274	43	255	255	255	1274	118	255	255	255
1275	1	255	255	255	1275	4	255	255	255	1275	1	255	255	255
1276	41	255	255	255	1276	2	255	255	255	1276	40	255	255	255
1277	1	255	255	255	1277	3	255	255	255	1277	29	255	255	255
1278	1	255	255	255	1278	22	255	255	255	1278	240	255	255	255
1279	1	255	255	255	1279	1	255	255	255	1279	28	255	255	255
1280	171	255	255	255	1280	30	255	255	255	1280	1	255	255	255
1281	169	255	255	255	1281	4	255	255	255	1281	1	255	255	255
1282	37	255	255	255	1282	1	255	255	255	1282	7	255	255	255
1283	2	255	255	255	1283	1	255	255	255	1283	50	255	255	255
1284	1	255	255	255	1284	3	255	255	255	1284	1	255	255	255
1285	256	255	255	255	1285	29	255	255	255	1285	2	255	255	255
1286	94	255	255	255	1286	3	255	255	255	1286	1	255	255	255
1287	3	255	255	255	1287	148	255	255	255	1287	1	255	255	255
1288	16	255	255	255	1288	3	255	255	255	1288	4	255	255	255
1289	1	255	255	255	1289	15	255	255	255	1289	178	255	255	255
1290	1	255	255	255	1290	73	255	255	255	1290	9	255	255	255
1291	118	255	255	255	1291	1	255	255	255	1291	293	255	255	255
1292	3	255	255	255	1292	1	255	255	255	1292	2	255	255	255
1293	1	255	255	255	1293	5	255	255	255	1293	1	255	255	255
1294	1	255	255	255	1294	1	255	255	255	1294	1	255	255	255
1295	20	255	255	255	1295	1	255	255	255	1295	8	255	255	255
1296	61	255	255	255	1296	17	255	255	255	1296	2	255	255	255
1297	29	255	255	255	1297	1	255	255	255	1297	6	255	255	255
1298	1	255	255	255	1298	1	255	255	255	1298	1	255	255	255
1299	1	255	255	255	1299	2	255	255	255	1299	1	255	255	255
1300	2	255	255	255	1300	4	255	255	255	1300	88	255	255	255
1301	8	255	255	255	1301	2	255	255	255	1301	3	255	255	255
1302	129	255	255	255	1302	1	255	255	255	1302	1	255	255	255
1303	138	255	255	255	1303	823	255	255	255	1303	1	255	255	255
1304	5	255	255	255	1304	1	255	255	255	1304	1	255	255	255
1305	56	255	255	255	1305	1	255	255	255	1305	1	255	255	255
1306	146	255	255	255	1306	2	255	255	255	1306	1	255	255	255
1307	18	255	255	255	1307	16	255	255	255	1307	9	255	255	255
1308	1	255	255	255	1308	45	255	255	255	1308	3	255	255	255
1309	2	255	255	255	1309	1	255	255	255	1309	1	255	255	255
1310	2	255	255	255	1310	4	255	255	255	1310	24	255	255	255
1311	303	255	255	255	1311	2	255	255	255	1311	1	255	255	255
1312	27	255	255	255	1312	1	255	255	255	1312	1	255	255	255
1313	34	255	255	255	1313	59	255	255	255	1313	2	255	255	255
1314	2	255	255	255	1314	1	255	255	255	1314	13	255	255	255
1315	1	255	255	255	1315	5	255	255	255	1315	2	255	255	255
1316	237	255	255	255	1316	4	255	255	255	1316	40	255	255	255
1317	56	255	255	255	1317	159	255	255	255	1317	1	255	255	255
1318	9	255	255	255	1318	1	255	255	255	1318	36	255	255	255
1319	14	255	255	255	1319	1	255	255	255	1319	1	255	255	255
1320	2	255	255	255	1320	9	255	255	255	1320	1	255	255	255
1321	3	255	255	255	1321	13	255	255	255	1321	1	255	255	255
1322	128	255	255	255	1322	2	255	255	255	1322	3	255	255	255
1323	1	255	255	255	1323	21	255	255	255	1323	3	255	255	255
1324	1	255	255	255	1324	1	255	255	255	1324	53	255	255	255
1325	49	255	255	255	1325	79	255	255	255	1325	1	255	255	255
1326	2	255	255	255	1326	260	255	255	255	1326	1	255	255	255
1327	1	255	255	255	1327	3	255	255	255	1327	2	255	255	255
1328	1	255	255	255	1328	1	255	255	255	1328	281	255	255	255
1329	9	255	255	255	1329	1	255	255	255	1329	4	255	255	255
1330	46	255	255	255	1330	68	255	255	255	1330	1	255	255	255
1331	29	255	255	255	1331	1	255	255	255	1331	1	255	255	255
1332	25	255	255	255	1332	91	255	255	255	1332	2	255	255	255
1333	2	255	255	255	1333	217	255	255	255	1333	1	255	255	255
1334	1	255	255	255	1334	10	255	255	255	1334	1	255	255	255
1335	83	255	255	255	1335	274	255	255	255	1335	28	255	255	255
1336	40	255	255	255	1336	2	255	255	255	1336	4	255	255	255
1337	1	255	255	255	1337	3	255	255	255	1337	431	255	255	255
1338	1	255	255	255	1338	23	255	255	255	1338	14	255	255	255
1339	41	255	255	255	1339	1	255	255	255	1339	2	255	255	255
1340	160	255	255	255	1340	329	255	255	255	1340	108	255	255	255
1341	4	255	255	255	1341	1	255	255	255	1341	15	255	255	255
1342	1	255	255	255	1342	4	255	255	255	1342	2	255	255	255
1343	2	255	255	255	1343	4	255	255	255	1343	4	255	255	255
1344	221	255	255	255	1344	1	255	255	255	1344	1	255	255	255
1345	2	255	255	255	1345	1	255	255	255	1345	2	255	255	255
1346	3	255	255	255	1346	24	255	255	255	1346	1	255	255	255
1347	2	255	255	255	1347	1	255	255	255	1347	2	255	255	255
1348	1	255	255	255	1348	49	255	255	255	1348	70	255	255	255
1349	1	255	255	255	1349	2	255	255	255	1349	4	255	255	255
1350	1	255	255	255	1350	1	255	255	255	1350	1	255	255	255
1351	1	255	255	255	1351	4	255	255	255	1351	18	255	255	255
1352	1	255	255	255	1352	115	255	255	255	1352	2	255	255	255
1353	1	255	255	255	1353	97	255	255	255	1353	1	255	255	255
1354	145	255	255	255	1354	3	255	255	255	1354	24	255	255	255
1355	171	255	255	255	1355	38	255	255	255	1355	1	255	255	255
1356	18	255	255	255	1356	1	255	255	255	1356	1	255	255	255
1357	38	255	255	255	1357	1	255	255	255	1357	1	255	255	255
1358	1	255	255	255	1358	55	255	255	255	1358	39	255	255	255
1359	240	255	255	255	1359	64	255	255	255	1359	1	255	255	255
1360	27	255	255	255	1360	13	255	255	255	1360	3	255	255	255
1361	2	255	255	255	1361	1	255	255	255	1361	1	255	255	255

Original data for Figure 2D, Densitometric analysis of Nuclear/Perinuclear B-catenin in control and EoE patient esophageal biopsy tissue.

Values are representative of the mean of a sample size N=18. p<0.05**

1362	3	255	255	255	1362	1	255	255	255	1362	26	255	255	255
1363	20	255	255	255	1363	2	255	255	255	1363	20	255	255	255
1364	4	255	255	255	1364	16	255	255	255	1364	1	255	255	255
1365	19	255	255	255	1365	85	255	255	255	1365	13	255	255	255
1366	205	255	255	255	1366	1	255	255	255	1366	2	255	255	255
1367	39	255	255	255	1367	12	255	255	255	1367	109	255	255	255
1368	84	255	255	255	1368	1	255	255	255	1368	1	255	255	255
1369	1	255	255	255	1369	238	255	255	255	1369	6	255	255	255
1370	8	255	255	255	1370	28	255	255	255	1370	4	255	255	255
1371	10	255	255	255	1371	76	255	255	255	1371	1	255	255	255
1372	231	255	255	255	1372	219	255	255	255	1372	1	255	255	255
1373	22	255	255	255	1373	1	255	255	255	1373	2	255	255	255
1374	18	255	255	255	1374	9	255	255	255	1374	1	255	255	255
1375	156	255	255	255	1375	11	255	255	255	1375	78	255	255	255
1376	1	255	255	255	1376	1	255	255	255	1376	2	255	255	255
1377	1	255	255	255	1377	1	255	255	255	1377	1	255	255	255
1378	140	255	255	255	1378	70	255	255	255	1378	5	255	255	255
1379	1	255	255	255	1379	2	255	255	255	1379	5	255	255	255
1380	43	255	255	255	1380	19	255	255	255	1380	10	255	255	255
1381	70	255	255	255	1381	69	255	255	255	1381	2	255	255	255
1382	2	255	255	255	1382	3	255	255	255	1382	3	255	255	255
1383	2	255	255	255	1383	502	255	255	255	1383	15	255	255	255
1384	35	255	255	255	1384	1	255	255	255	1384	88	255	255	255
1385	129	255	255	255	1385	1	255	255	255	1385	60	255	255	255
1386	140	255	255	255	1386	37	255	255	255	1386	2	255	255	255
1387	1	255	255	255	1387	2	255	255	255	1387	3	255	255	255
1388	6	255	255	255	1388	2	255	255	255	1388	2	255	255	255
1389	1	255	255	255	1389	1	255	255	255	1389	1	255	255	255
1390	1	255	255	255	1390	7	255	255	255	1390	56	255	255	255
1391	1	255	255	255	1391	1	255	255	255	1391	3	255	255	255
1392	33	255	255	255	1392	219	255	255	255	1392	1	255	255	255
1393	2	255	255	255	1393	1	255	255	255	1393	6	255	255	255
1394	1	255	255	255	1394	3	255	255	255	1394	3	255	255	255
1395	1	255	255	255	1395	44	255	255	255	1395	13	255	255	255
1396	1	255	255	255	1396	1	255	255	255	1396	5	255	255	255
1397	2	255	255	255	1397	1	255	255	255	1397	2	255	255	255
1398	1	255	255	255	1398	38	255	255	255	1398	2	255	255	255
1399	1	255	255	255	1399	1	255	255	255	1399	54	255	255	255
1400	218	255	255	255	1400	1	255	255	255	1400	315	255	255	255
1401	3	255	255	255	1401	1	255	255	255	1401	2	255	255	255
1402	7	255	255	255	1402	1	255	255	255	1402	46	255	255	255
1403	1	255	255	255	1403	38	255	255	255	1403	1	255	255	255
1404	138	255	255	255	1404	110	255	255	255	1404	1	255	255	255
1405	80	255	255	255	1405	490	255	255	255	1405	19	255	255	255
1406	79	255	255	255	1406	27	255	255	255	1406	44	255	255	255
1407	71	255	255	255	1407	1	255	255	255	1407	76	255	255	255
1408	1	255	255	255	1408	3	255	255	255	1408	12	255	255	255
1409	2	255	255	255	1409	19	255	255	255	1409	4	255	255	255
1410	4	255	255	255	1410	1	255	255	255	1410	1	255	255	255
1411	1	255	255	255	1411	18	255	255	255	1411	3	255	255	255
1412	57	255	255	255	1412	1	255	255	255	1412	43	255	255	255
1413	2	255	255	255	1413	5	255	255	255	1413	56	255	255	255
1414	8	255	255	255	1414	2	255	255	255	1414	2	255	255	255
1415	1	255	255	255	1415	5	255	255	255	1415	2	255	255	255
1416	1	255	255	255	1416	1	255	255	255	1416	2	255	255	255
1417	1	255	255	255	1417	4	255	255	255	1417	52	255	255	255
1418	2	255	255	255	1418	6	255	255	255	1418	11	255	255	255
1419	108	255	255	255	1419	59	255	255	255	1419	10	255	255	255
1420	8	255	255	255	1420	1	255	255	255	1420	16	255	255	255
1421	1	255	255	255	1421	1	255	255	255	1421	12	255	255	255
1422	96	255	255	255	1422	1	255	255	255	1422	13	255	255	255
1423	1	255	255	255	1423	294	255	255	255	1423	5	255	255	255
1424	49	255	255	255	1424	1	255	255	255	1424	3	255	255	255
1425	1	255	255	255	1425	602	255	255	255	1425	12	255	255	255
1426	1	255	255	255	1426	8	255	255	255	1426	28	255	255	255
1427	2	255	255	255	1427	1	255	255	255	1427	94	255	255	255
1428	1	255	255	255	1428	2	255	255	255	1428	1	255	255	255
1429	2	255	255	255	1429	1	255	255	255	1429	2	255	255	255
1430	2	255	255	255	1430	1	255	255	255	1430	3	255	255	255
1431	2	255	255	255	1431	1	255	255	255	1431	58	255	255	255
1432	1	255	255	255	1432	1185	255	255	255	1432	26	255	255	255
1433	11	255	255	255	1433	68	255	255	255	1433	3	255	255	255
1434	34	255	255	255	1434	5	255	255	255	1434	1	255	255	255
1435	72	255	255	255	1435	1	255	255	255	1435	1	255	255	255
1436	10	255	255	255	1436	1	255	255	255	1436	1	255	255	255
1437	1	255	255	255	1437	57	255	255	255	1437	1	255	255	255
1438	37	255	255	255	1438	1	255	255	255	1438	28	255	255	255
1439	11	255	255	255	1439	1	255	255	255	1439	1	255	255	255
1440	2	255	255	255	1440	1	255	255	255	1440	42	255	255	255
1441	4	255	255	255	1441	1	255	255	255	1441	1	255	255	255
1442	2	255	255	255	1442	1	255	255	255	1442	1	255	255	255
1443	2	255	255	255	1443	1	255	255	255	1443	53	255	255	255
1444	100	255	255	255	1444	1	255	255	255	1444	1	255	255	255
1445	315	255	255	255	1445	1	255	255	255	1445	1	255	255	255
1446	2	255	255	255	1446	2	255	255	255	1446	104	255	255	255
1447	1	255	255	255	1447	70	255	255	255	1447	1	255	255	255
1448	34	255	255	255	1448	1	255	255	255	1448	4	255	255	255
1449	3	255	255	255	1449	1	255	255	255	1449	27	255	255	255
1450	640	255	255	255	1450	1	255	255	255	1450	4	255	255	255
1451	128	255	255	255	1451	2	255	255	255	1451	1	255	255	255
1452	37	255	255	255	1452	2	255	255	255	1452	1	255	255	255
1453	2	255	255	255	1453	1	255	255	255	1453	1	255	255	255
1454	1	255	255	255	1454	203	255	255	255	1454	13	255	255	255
1455	41	255	255	255	1455	21	255	255	255	1455	1	255	255	255
1456	71	255	255	255	1456	1	255	255	255	1456	1	255	255	255
1457	17	255	255	255	1457	1	255	255	255	1457	196	255	255	255
1458	43	255	255	255	1458	38	255	255	255	1458	3	255	255	255
1459	1	255	255	255	1459	1	255	255	255	1459	27	255	255	255
1460	2	255	255	255	1460	14	255	255	255	1460	256	255	255	255
1461	169	255	255	255	1461	111	255	255	255	1461	3	255	255	255
1462	5	255	255	255	1462	2	255	255	255	1462	11	255	255	255
1463	14	255	255	255	1463	6	255	255	255	1463	174	255	255	255
1464	1	255	255	255	1464	1	255	255	255	1464	10	255	255	255
1465	713	255	255	255	1465	4	255	255	255	1465	4	255	255	255
1466	3	255	255	255	1466	1	255	255	255	1466	1	255	255	255
1467	48	255	255	255	1467	2	255	255	255	1467	30	255	255	255
1468	11	255	255	255	1468	1	255	255	255	1468	2	255	255	255
1469	4	255	255	255	1469	1	255	255	255	1469	1	255	255	255
1470	72	255	255	255	1470	11	255	255	255	1470	1	255	255	255
1471	1	255	255	255	1471	20	255	255	255	1471	1	255	255	255
1472	1	255	255	255	1472	895	255	255	255	1472	35	255	255	255
1473	6	255	255	255	1473	1	255	255	255	1473	1	255	255	255
1474	1	255	255	255	1474	2	255	255	255	1474	2	255	255	255
1475	14	255	255	255	1475	39	255	255	255	1475	9	255	255	255
1476	17	255	255	255	1476	1	255	255	255	1476	3	255	255	255
1477	1	255	255	255	1477	1	255	255	255	1477	276	255	255	255
1478	257	255	255	255	1478	80	255	255	255	1478	1	255	255	255
1479	3	255	255	255	1479	2	255	255	255	1479	23	255	255	255
1480	42	255	255	255	1480	1	255	255	255	1480	1	255	255	255
1481	38	255	255	255	1481	1	255	255	255	1481	3	255	255	255
1482	1	255	255	255	1482	11	255	255	255	1482	122	255	255	255
1483	1	255	255	255	1483	1	255	255	255	1483	2	255	255	255
1484	198	255	255	255	1484	69	255	255	255	1484	1	255	255	255
1485	1	255	255	255	1485	3	255	255	255	1485	84	255	255	255
1486	27	255	255	255	1486	3	255	255	255	1486	2	255	255	255
1487	19	255	255	255	1487	342	255	255	255	1487	1	255	255	255
1488	3	255	255	255	1488	78	255	255	255	1488	68	255	255	255
1489	54	255	255	255	1489	1	255	255	255	1489	2	255	255	255
1490	142	255	255	255	1490	1	255	255	255	1490	20	255	255	255
1491	3	255	255	255	1491	2	255	255	255	1491	2	255	255	255
1492	67	255	255	255	1492	1	255	255	255	1492	1	255	255	255
1493	2	255	255	255	1493	209	255	255	255	1493	39	255	255	255
1494	6	255	255	255	1494	1	255	255	255	1494	20	255	255	255
1495	2	255	255	255	1495	1243	255	255	255	1495	1	255	255	255
1496	1	255	255	255	1496	1	255	255	255	1496	1	255	255	255
1497	1	255	255	255	1497	1	255	255	255	1497	38	255	255	255
1498	1	255	255	255	1498	2	255	255	255	1498	22	255	255	255
1499	1	255	255	255	1499	1	255	255	255	1499	73	255	255	255

Original data for Figure 2D, Densitometric analysis of Nuclear/Perinuclear B-catenin in control and EoE patient esophageal biopsy tissue.

Values are representative of the mean of a sample size N=18. p<0.05**

1500	127	255	255	255
1501	2	255	255	255
1502	1	255	255	255
1503	1	255	255	255
1504	2	255	255	255
1505	1	255	255	255
1506	282	255	255	255
1507	25	255	255	255
1508	1	255	255	255
1509	1	255	255	255
1510	3	255	255	255
1511	9	255	255	255
1512	1	255	255	255
1513	1	255	255	255
1514	1	255	255	255
1515	2	255	255	255
1516	13	255	255	255
1517	1	255	255	255
1518	1	255	255	255
1519	229	255	255	255
1520	1	255	255	255
1521	11	255	255	255
1522	2	255	255	255
1523	21	255	255	255
1524	41	255	255	255
1525	1	255	255	255
1526	2	255	255	255
1527	32	255	255	255
1528	106	255	255	255
1529	35	255	255	255
1530	28	255	255	255
1531	29	255	255	255
1532	7	255	255	255
1533	1	255	255	255
1534	1	255	255	255
1535	1	255	255	255
1536	11	255	255	255
1537	1	255	255	255
1538	1	255	255	255
1539	1	255	255	255
1540	2	255	255	255
1541	1	255	255	255
1542	97	255	255	255
1543	1	255	255	255
1544	2	255	255	255
1545	1	255	255	255
1546	127	255	255	255
1547	2	255	255	255
1548	16	255	255	255
1549	3	255	255	255
1550	1	255	255	255
1551	11	255	255	255
1552	4	255	255	255
1553	2	255	255	255
1554	1	255	255	255
1555	25	255	255	255
1556	2	255	255	255
1557	100	255	255	255
1558	197	255	255	255
1559	3	255	255	255
1560	78	255	255	255
1561	1	255	255	255
1562	9	255	255	255
1563	3	255	255	255
1564	1	255	255	255
1565	1	255	255	255
1566	1	255	255	255
1567	3	255	255	255
1568	6	255	255	255
1569	2	255	255	255
1570	2	255	255	255
1571	1	255	255	255
1572	1	255	255	255
1573	1	255	255	255
1574	32	255	255	255
1575	1	255	255	255
1576	13	255	255	255
1577	1	255	255	255
1578	512	255	255	255
1579	1	255	255	255
1580	1	255	255	255
1581	2	255	255	255
1582	1	255	255	255
1583	6	255	255	255
1584	8	255	255	255
1585	50	255	255	255
1586	140	255	255	255
1587	7	255	255	255

1500	2	255	255	255
1501	34	255	255	255
1502	1	255	255	255
1503	3	255	255	255
1504	4	255	255	255
1505	8	255	255	255
1506	50	255	255	255
1507	263	255	255	255
1508	1	255	255	255
1509	246	255	255	255
1510	22	255	255	255
1511	2	255	255	255
1512	4	255	255	255
1513	160	255	255	255
1514	12	255	255	255
1515	1	255	255	255
1516	5	255	255	255
1517	1	255	255	255
1518	1	255	255	255
1519	3	255	255	255
1520	44	255	255	255
1521	3	255	255	255
1522	30	255	255	255
1523	60	255	255	255
1524	15	255	255	255
1525	1	255	255	255
1526	1	255	255	255
1527	17	255	255	255
1528	1	255	255	255
1529	1	255	255	255
1530	1	255	255	255
1531	13	255	255	255
1532	1	255	255	255
1533	1	255	255	255
1534	1	255	255	255
1535	1	255	255	255
1536	3	255	255	255
1537	3	255	255	255
1538	1	255	255	255
1539	28	255	255	255
1540	73	255	255	255
1541	2	255	255	255
1542	2	255	255	255
1543	1	255	255	255
1544	1	255	255	255
1545	42	255	255	255
1546	67	255	255	255
1547	1	255	255	255
1548	2	255	255	255
1549	1	255	255	255
1550	17	255	255	255
1551	1	255	255	255
1552	4	255	255	255
1553	20	255	255	255
1554	1	255	255	255
1555	56	255	255	255
1556	43	255	255	255
1557	1	255	255	255
1558	152	255	255	255
1559	30	255	255	255
1560	1	255	255	255
1561	31	255	255	255
1562	2	255	255	255
1563	16	255	255	255
1564	10	255	255	255
1565	7	255	255	255
1566	19	255	255	255
1567	1	255	255	255
1568	32	255	255	255
1569	1	255	255	255
1570	1	255	255	255
1571	3	255	255	255
1572	94	255	255	255
1573	24	255	255	255
1574	1	255	255	255
1575	1	255	255	255
1576	3	255	255	255
1577	11	255	255	255
1578	3	255	255	255
1579	54	255	255	255
1580	5	255	255	255
1581	1	255	255	255
1582	1	255	255	255
1583	757	255	255	255
1584	15	255	255	255
1585	26	255	255	255
1586	50	255	255	255
1587	69	255	255	255

1500	1	255	255	255
1501	6	255	255	255
1502	3	255	255	255
1503	9	255	255	255
1504	1	255	255	255
1505	1	255	255	255
1506	3	255	255	255
1507	85	255	255	255
1508	15	255	255	255
1509	24	255	255	255
1510	5	255	255	255
1511	8	255	255	255
1512	3	255	255	255
1513	20	255	255	255
1514	6	255	255	255
1515	1	255	255	255
1516	27	255	255	255
1517	4	255	255	255
1518	18	255	255	255
1519	2	255	255	255
1520	9	255	255	255
1521	1	255	255	255
1522	79	255	255	255
1523	39	255	255	255
1524	6	255	255	255
1525	1	255	255	255
1526	3	255	255	255
1527	1	255	255	255
1528	14	255	255	255
1529	1	255	255	255
1530	1	255	255	255
1531	1	255	255	255
1532	65	255	255	255
1533	1	255	255	255
1534	350	255	255	255
1535	2	255	255	255
1536	160	255	255	255
1537	52	255	255	255
1538	1	255	255	255
1539	58	255	255	255
1540	1	255	255	255
1541	4	255	255	255
1542	1	255	255	255
1543	15	255	255	255
1544	16	255	255	255
1545	1	255	255	255
1546	2723	255	255	255
1547	1	255	255	255
1548	15	255	255	255
1549	1	255	255	255
1550	4	255	255	255
1551	5	255	255	255
1552	1	255	255	255
1553	164	255	255	255
1554	3	255	255	255
1555	1	255	255	255
1556	1	255	255	255
1557	8	255	255	255
1558	13	255	255	255
1559	34	255	255	255
1560	169	255	255	255
1561	188	255	255	255
1562	1	255	255	255
1563	4	255	255	255
1564	75	255	255	255
1565	2	255	255	255
1566	2	255	255	255
1567	1	255	255	255
1568	6	255	255	255
1569	19	255	255	255
1570	50	255	255	255
1571	1	255	255	255
1572	243	255	255	255
1573	59	255	255	255
1574	5	255	255	255
1575	547	255	255	255
1576	15	255	255	255

Original data for Figure 2D, Densitometric analysis of Nuclear/Perinuclear B-catenin in control and EoE patient esophageal biopsy tissue.
Values are representative of the mean of a sample size N=18. p<0.05**

EoE
EoE patient esophageal biopsy tissue

Count	Total Area	Average Size			Count	Total Area	Average Size		
Nuclear A	32	255	255	255	Nuclear A	1	255	255	255
2	1	255	255	255	2	108	255	255	255
3	15	255	255	255	3	7	255	255	255
4	2	255	255	255	4	3	255	255	255
5	1	255	255	255	5	3	255	255	255
6	2	255	255	255	6	2	255	255	255
7	1	255	255	255	7	13	255	255	255
8	1	255	255	255	8	16	255	255	255
9	33	255	255	255	9	1	255	255	255
10	232	255	255	255	10	47	255	255	255
11	523	255	255	255	11	3	255	255	255
12	1	255	255	255	12	2	255	255	255
13	1	255	255	255	13	2	255	255	255
14	56	255	255	255	14	1	255	255	255
15	1	255	255	255	15	1	255	255	255
16	2	255	255	255	16	13	255	255	255
17	58	255	255	255	17	94	255	255	255
18	1	255	255	255	18	2	255	255	255
19	1	255	255	255	19	4	255	255	255
20	6	255	255	255	20	2	255	255	255
21	2	255	255	255	21	1	255	255	255
22	1	255	255	255	22	1	255	255	255
23	1	255	255	255	23	11	255	255	255
24	18	255	255	255	24	3	255	255	255
25	1	255	255	255	25	32	255	255	255
26	1	255	255	255	26	1	255	255	255
27	3	255	255	255	27	5	255	255	255
28	17	255	255	255	28	2	255	255	255
29	1	255	255	255	29	26	255	255	255
30	5	255	255	255	30	1	255	255	255
31	17	255	255	255	31	1	255	255	255
32	46	255	255	255	32	1	255	255	255
33	1	255	255	255	33	36	255	255	255
34	25	255	255	255	34	16	255	255	255
35	17	255	255	255	35	1	255	255	255
36	29	255	255	255	36	2	255	255	255
37	1	255	255	255	37	9	255	255	255
38	61	255	255	255	38	20	255	255	255
39	522	255	255	255	39	6	255	255	255
40	27	255	255	255	40	2	255	255	255
41	2	255	255	255	41	2	255	255	255
42	9	255	255	255	42	4	255	255	255
43	1	255	255	255	43	3	255	255	255
44	8	255	255	255	44	13	255	255	255
45	83	255	255	255	45	4	255	255	255
46	1	255	255	255	46	1	255	255	255
47	1	255	255	255	47	1	255	255	255
48	1	255	255	255	48	1	255	255	255
49	13	255	255	255	49	1	255	255	255
50	6	255	255	255	50	1	255	255	255
51	3	255	255	255	51	31	255	255	255
52	8	255	255	255	52	1	255	255	255
53	1	255	255	255	53	34	255	255	255
54	1	255	255	255	54	3	255	255	255
55	61	255	255	255	55	5	255	255	255
56	1	255	255	255	56	1	255	255	255
57	1	255	255	255	57	19	255	255	255
58	2	255	255	255	58	9	255	255	255
59	13	255	255	255	59	7	255	255	255
60	1	255	255	255	60	1	255	255	255
61	36	255	255	255	61	4	255	255	255
62	50	255	255	255	62	3	255	255	255
63	5	255	255	255	63	2	255	255	255
64	1	255	255	255	64	1	255	255	255
65	2	255	255	255	65	26	255	255	255
66	2	255	255	255	66	2	255	255	255
67	14	255	255	255	67	1	255	255	255
68	5	255	255	255	68	1	255	255	255
69	19	255	255	255	69	5	255	255	255
70	7	255	255	255	70	2	255	255	255
71	41	255	255	255	71	2	255	255	255
72	1	255	255	255	72	1	255	255	255
73	210	255	255	255	73	2	255	255	255
74	3	255	255	255	74	1	255	255	255
75	1	255	255	255	75	54	255	255	255
76	49	255	255	255	76	6	255	255	255
77	12	255	255	255	77	2	255	255	255
78	2	255	255	255	78	1	255	255	255
79	7	255	255	255	79	1	255	255	255
80	2	255	255	255	80	2	255	255	255
81	26	255	255	255	81	6	255	255	255
82	431	255	255	255	82	9	255	255	255
83	203	255	255	255	83	25	255	255	255
84	19	255	255	255	84	6	255	255	255
85	192	255	255	255	85	14	255	255	255
86	1	255	255	255	86	15	255	255	255
87	1	255	255	255	87	2	255	255	255
88	3	255	255	255	88	1	255	255	255
89	64	255	255	255	89	1	255	255	255
90	9	255	255	255	90	2	255	255	255
91	47	255	255	255	91	265	255	255	255
92	1	255	255	255	92	6	255	255	255
93	1	255	255	255	93	2	255	255	255
94	2	255	255	255	94	30	255	255	255
95	122	255	255	255	95	6	255	255	255
96	22	255	255	255	96	2	255	255	255
97	21	255	255	255	97	2	255	255	255
98	8	255	255	255	98	1	255	255	255
99	2	255	255	255	99	25	255	255	255
100	19	255	255	255	100	93	255	255	255
101	1	255	255	255	101	30	255	255	255
102	5	255	255	255	102	1	255	255	255
103	2	255	255	255	103	4	255	255	255
104	56	255	255	255	104	1	255	255	255
105	2	255	255	255	105	1	255	255	255
106	1	255	255	255	106	1	255	255	255
107	2	255	255	255	107	24	255	255	255
108	3	255	255	255	108	106	255	255	255
109	15	255	255	255	109	4	255	255	255
110	7	255	255	255	110	4	255	255	255
111	1	255	255	255	111	2	255	255	255
112	1	255	255	255	112	1	255	255	255
113	1	255	255	255	113	2	255	255	255
114	2	255	255	255	114	118	255	255	255
115	12	255	255	255	115	67	255	255	255
116	60	255	255	255	116	3	255	255	255
117	1	255	255	255	117	185	255	255	255
118	3	255	255	255	118	1	255	255	255
119	1	255	255	255	119	20	255	255	255

Statistics:

Table	Final analysis IHC Beta catenin Nuclear
Column A	Control
vs	vs
Column B	EoE
Unpaired t test	
P value	< 0.004
P value sur	**
Are means	Yes
One- or tw	Two-tailed
t, df	t=6.969 df=15
How big is the difference?	
Mean ± SEI	652.3 ± 109.5 N=9
Mean ± SEI	1504 ± 39.11 N=8
Difference	-852.0 ± 122.3
95% confid	-1113 to -591.5
R squared	0.764
F test to compare variances	
F,DFn, Dfd	8.826, 8, 7
P value	0.0095
P value sur	**
Are varianc	Yes

Original data for Figure 2D, Densitometric analysis of Nuclear/Perinuclear B-catenin in control and EoE patient esophageal biopsy tissue.

Values are representative of the mean of a sample size N=18. p<0.05**

120	1	255	255	255	120	8	255	255	255
121	15	255	255	255	121	77	255	255	255
122	70	255	255	255	122	2	255	255	255
123	2	255	255	255	123	6	255	255	255
124	3	255	255	255	124	1	255	255	255
125	74	255	255	255	125	3	255	255	255
126	1	255	255	255	126	11	255	255	255
127	5	255	255	255	127	5	255	255	255
128	1	255	255	255	128	8	255	255	255
129	1	255	255	255	129	7	255	255	255
130	4	255	255	255	130	2	255	255	255
131	4031	255	255	255	131	2	255	255	255
132	1	255	255	255	132	30	255	255	255
133	2	255	255	255	133	37	255	255	255
134	1	255	255	255	134	4	255	255	255
135	50	255	255	255	135	1	255	255	255
136	3	255	255	255	136	86	255	255	255
137	954	255	255	255	137	4	255	255	255
138	1	255	255	255	138	5	255	255	255
139	1	255	255	255	139	2	255	255	255
140	2	255	255	255	140	7	255	255	255
141	1	255	255	255	141	24	255	255	255
142	3	255	255	255	142	9	255	255	255
143	13	255	255	255	143	2	255	255	255
144	1	255	255	255	144	21	255	255	255
145	2	255	255	255	145	19	255	255	255
146	92	255	255	255	146	1	255	255	255
147	288	255	255	255	147	2	255	255	255
148	4	255	255	255	148	1	255	255	255
149	1	255	255	255	149	2	255	255	255
150	11	255	255	255	150	41	255	255	255
151	2	255	255	255	151	10	255	255	255
152	1	255	255	255	152	1	255	255	255
153	1	255	255	255	153	1	255	255	255
154	18	255	255	255	154	2	255	255	255
155	35	255	255	255	155	2	255	255	255
156	12	255	255	255	156	35	255	255	255
157	2	255	255	255	157	56	255	255	255
158	141	255	255	255	158	7	255	255	255
159	17	255	255	255	159	1	255	255	255
160	6	255	255	255	160	1	255	255	255
161	312	255	255	255	161	3	255	255	255
162	55	255	255	255	162	1	255	255	255
163	3	255	255	255	163	8	255	255	255
164	2	255	255	255	164	1	255	255	255
165	47	255	255	255	165	2	255	255	255
166	51	255	255	255	166	1	255	255	255
167	1	255	255	255	167	11	255	255	255
168	5	255	255	255	168	6	255	255	255
169	1	255	255	255	169	1	255	255	255
170	27	255	255	255	170	4	255	255	255
171	88	255	255	255	171	36	255	255	255
172	2	255	255	255	172	3	255	255	255
173	121	255	255	255	173	34	255	255	255
174	1	255	255	255	174	41	255	255	255
175	2	255	255	255	175	32	255	255	255
176	2	255	255	255	176	3	255	255	255
177	4	255	255	255	177	9	255	255	255
178	6	255	255	255	178	2	255	255	255
179	3	255	255	255	179	1	255	255	255
180	47	255	255	255	180	12	255	255	255
181	1	255	255	255	181	1	255	255	255
182	23	255	255	255	182	1	255	255	255
183	2	255	255	255	183	1	255	255	255
184	1	255	255	255	184	6	255	255	255
185	32	255	255	255	185	7	255	255	255
186	81	255	255	255	186	1	255	255	255
187	5	255	255	255	187	2	255	255	255
188	2	255	255	255	188	1	255	255	255
189	25	255	255	255	189	13	255	255	255
190	1	255	255	255	190	1	255	255	255
191	1	255	255	255	191	5	255	255	255
192	161	255	255	255	192	3	255	255	255
193	2	255	255	255	193	1	255	255	255
194	30	255	255	255	194	1	255	255	255
195	3	255	255	255	195	2	255	255	255
196	1	255	255	255	196	5	255	255	255
197	4	255	255	255	197	288	255	255	255
198	22	255	255	255	198	2	255	255	255
199	3	255	255	255	199	3	255	255	255
200	1	255	255	255	200	1	255	255	255
201	113	255	255	255	201	1	255	255	255
202	1	255	255	255	202	7	255	255	255
203	1	255	255	255	203	2	255	255	255
204	2	255	255	255	204	2	255	255	255
205	20	255	255	255	205	42	255	255	255
206	14	255	255	255	206	1	255	255	255
207	26	255	255	255	207	3	255	255	255
208	2	255	255	255	208	17	255	255	255
209	1	255	255	255	209	23	255	255	255
210	1	255	255	255	210	3	255	255	255
211	2	255	255	255	211	19	255	255	255
212	1	255	255	255	212	4	255	255	255
213	94	255	255	255	213	2	255	255	255
214	1	255	255	255	214	181	255	255	255
215	2	255	255	255	215	5	255	255	255
216	1	255	255	255	216	9	255	255	255
217	2	255	255	255	217	1	255	255	255
218	1	255	255	255	218	2	255	255	255
219	1	255	255	255	219	2	255	255	255
220	1	255	255	255	220	1	255	255	255
221	450	255	255	255	221	5	255	255	255
222	6	255	255	255	222	2	255	255	255
223	55	255	255	255	223	3	255	255	255
224	1	255	255	255	224	2	255	255	255
225	177	255	255	255	225	43	255	255	255
226	1	255	255	255	226	14	255	255	255
227	41	255	255	255	227	24	255	255	255
228	1	255	255	255	228	2	255	255	255
229	33	255	255	255	229	35	255	255	255
230	2	255	255	255	230	18	255	255	255
231	22	255	255	255	231	1	255	255	255
232	8	255	255	255	232	3	255	255	255
233	5	255	255	255	233	57	255	255	255
234	1	255	255	255	234	36	255	255	255
235	108	255	255	255	235	1	255	255	255
236	1	255	255	255	236	2	255	255	255
237	15	255	255	255	237	3	255	255	255
238	3	255	255	255	238	2	255	255	255
239	5	255	255	255	239	4	255	255	255
240	1	255	255	255	240	5	255	255	255
241	59	255	255	255	241	2	255	255	255
242	2	255	255	255	242	5	255	255	255
243	55	255	255	255	243	4	255	255	255
244	1	255	255	255	244	5	255	255	255
245	1	255	255	255	245	12	255	255	255
246	79	255	255	255	246	13	255	255	255
247	1	255	255	255	247	1	255	255	255
248	11	255	255	255	248	1	255	255	255
249	113	255	255	255	249	3	255	255	255
250	3	255	255	255	250	14	255	255	255
251	73	255	255	255	251	2	255	255	255
252	25	255	255	255	252	1	255	255	255
253	2	255	255	255	253	2	255	255	255
254	38	255	255	255	254	1	255	255	255
255	7	255	255	255	255	12	255	255	255
256	3	255	255	255	256	44	255	255	255
257	1	255	255	255	257	1	255	255	255

Original data for Figure 2D, Densitometric analysis of Nuclear/Perinuclear B-catenin in control and EoE patient esophageal biopsy tissue.

Values are representative of the mean of a sample size N=18. p<0.05**

258	12	255	255	255	258	10	255	255	255
259	9	255	255	255	259	58	255	255	255
260	2	255	255	255	260	27	255	255	255
261	2	255	255	255	261	1	255	255	255
262	2	255	255	255	262	1	255	255	255
263	1	255	255	255	263	1	255	255	255
264	1	255	255	255	264	1	255	255	255
265	5	255	255	255	265	2	255	255	255
266	2	255	255	255	266	1	255	255	255
267	2	255	255	255	267	8	255	255	255
268	2	255	255	255	268	51	255	255	255
269	169	255	255	255	269	1	255	255	255
270	2	255	255	255	270	1	255	255	255
271	1	255	255	255	271	1	255	255	255
272	1	255	255	255	272	2	255	255	255
273	16	255	255	255	273	2	255	255	255
274	1	255	255	255	274	64	255	255	255
275	5	255	255	255	275	25	255	255	255
276	2	255	255	255	276	4	255	255	255
277	2	255	255	255	277	2	255	255	255
278	14	255	255	255	278	1	255	255	255
279	8	255	255	255	279	37	255	255	255
280	7	255	255	255	280	1	255	255	255
281	1	255	255	255	281	3	255	255	255
282	6	255	255	255	282	4	255	255	255
283	1	255	255	255	283	1	255	255	255
284	132	255	255	255	284	5	255	255	255
285	44	255	255	255	285	34	255	255	255
286	17	255	255	255	286	1	255	255	255
287	181	255	255	255	287	16	255	255	255
288	2	255	255	255	288	10	255	255	255
289	2	255	255	255	289	2	255	255	255
290	2	255	255	255	290	10	255	255	255
291	1	255	255	255	291	3	255	255	255
292	1	255	255	255	292	2	255	255	255
293	30	255	255	255	293	1	255	255	255
294	1	255	255	255	294	4	255	255	255
295	1	255	255	255	295	1	255	255	255
296	1	255	255	255	296	76	255	255	255
297	1	255	255	255	297	2	255	255	255
298	1	255	255	255	298	38	255	255	255
299	1	255	255	255	299	38	255	255	255
300	2	255	255	255	300	63	255	255	255
301	1	255	255	255	301	2	255	255	255
302	1	255	255	255	302	1	255	255	255
303	1	255	255	255	303	41	255	255	255
304	8	255	255	255	304	12	255	255	255
305	1	255	255	255	305	1	255	255	255
306	1	255	255	255	306	1	255	255	255
307	3	255	255	255	307	1	255	255	255
308	45	255	255	255	308	3	255	255	255
309	6	255	255	255	309	10	255	255	255
310	1	255	255	255	310	25	255	255	255
311	1	255	255	255	311	2	255	255	255
312	21	255	255	255	312	1	255	255	255
313	1	255	255	255	313	1	255	255	255
314	1	255	255	255	314	1	255	255	255
315	10	255	255	255	315	3	255	255	255
316	35	255	255	255	316	17	255	255	255
317	2	255	255	255	317	7	255	255	255
318	3	255	255	255	318	22	255	255	255
319	3	255	255	255	319	3	255	255	255
320	10	255	255	255	320	4	255	255	255
321	2	255	255	255	321	1	255	255	255
322	73	255	255	255	322	1	255	255	255
323	10	255	255	255	323	386	255	255	255
324	16	255	255	255	324	1	255	255	255
325	18	255	255	255	325	3	255	255	255
326	1	255	255	255	326	1	255	255	255
327	1	255	255	255	327	6	255	255	255
328	1	255	255	255	328	1	255	255	255
329	1	255	255	255	329	1	255	255	255
330	9	255	255	255	330	1	255	255	255
331	2	255	255	255	331	99	255	255	255
332	34	255	255	255	332	11	255	255	255
333	2	255	255	255	333	17	255	255	255
334	203	255	255	255	334	17	255	255	255
335	24	255	255	255	335	54	255	255	255
336	26	255	255	255	336	50	255	255	255
337	111	255	255	255	337	2	255	255	255
338	4	255	255	255	338	1	255	255	255
339	2	255	255	255	339	1	255	255	255
340	5	255	255	255	340	1	255	255	255
341	3	255	255	255	341	24	255	255	255
342	7	255	255	255	342	2	255	255	255
343	1	255	255	255	343	2	255	255	255
344	21	255	255	255	344	3	255	255	255
345	3	255	255	255	345	1	255	255	255
346	8	255	255	255	346	1	255	255	255
347	1	255	255	255	347	7	255	255	255
348	98	255	255	255	348	21	255	255	255
349	1	255	255	255	349	1	255	255	255
350	1	255	255	255	350	83	255	255	255
351	1	255	255	255	351	78	255	255	255
352	46	255	255	255	352	9	255	255	255
353	10	255	255	255	353	8	255	255	255
354	1	255	255	255	354	2	255	255	255
355	18	255	255	255	355	6	255	255	255
356	1	255	255	255	356	4	255	255	255
357	5	255	255	255	357	21	255	255	255
358	27	255	255	255	358	2	255	255	255
359	7	255	255	255	359	9	255	255	255
360	2	255	255	255	360	1	255	255	255
361	1	255	255	255	361	28	255	255	255
362	1	255	255	255	362	47	255	255	255
363	2	255	255	255	363	6	255	255	255
364	2	255	255	255	364	3	255	255	255
365	3	255	255	255	365	1	255	255	255
366	1	255	255	255	366	6	255	255	255
367	1	255	255	255	367	1	255	255	255
368	1	255	255	255	368	3	255	255	255
369	1	255	255	255	369	1	255	255	255
370	1	255	255	255	370	41	255	255	255
371	1	255	255	255	371	15	255	255	255
372	2	255	255	255	372	3	255	255	255
373	3	255	255	255	373	1	255	255	255
374	1	255	255	255	374	3	255	255	255
375	1	255	255	255	375	1	255	255	255
376	57	255	255	255	376	1	255	255	255
377	1	255	255	255	377	5	255	255	255
378	1	255	255	255	378	75	255	255	255
379	2	255	255	255	379	6	255	255	255
380	6	255	255	255	380	1	255	255	255
381	58	255	255	255	381	2	255	255	255
382	1	255	255	255	382	2	255	255	255
383	1	255	255	255	383	1	255	255	255
384	2	255	255	255	384	2	255	255	255
385	5	255	255	255	385	1	255	255	255
386	2	255	255	255	386	1	255	255	255
387	1	255	255	255	387	54	255	255	255
388	19	255	255	255	388	23	255	255	255
389	1	255	255	255	389	2	255	255	255
390	5	255	255	255	390	3	255	255	255
391	2	255	255	255	391	2	255	255	255
392	52	255	255	255	392	2	255	255	255
393	1	255	255	255	393	5	255	255	255
394	2	255	255	255	394	7	255	255	255
395	3	255	255	255	395	1	255	255	255

Original data for Figure 2D, Densitometric analysis of Nuclear/Perinuclear B-catenin in control and EoE patient esophageal biopsy tissue.

Values are representative of the mean of a sample size N=18. p<0.05**

396	1	255	255	255	396	1	255	255	255
397	64	255	255	255	397	1	255	255	255
398	181	255	255	255	398	12	255	255	255
399	1	255	255	255	399	7	255	255	255
400	1	255	255	255	400	5	255	255	255
401	128	255	255	255	401	13	255	255	255
402	238	255	255	255	402	5	255	255	255
403	19	255	255	255	403	10	255	255	255
404	1	255	255	255	404	2	255	255	255
405	1	255	255	255	405	1	255	255	255
406	2	255	255	255	406	5	255	255	255
407	35	255	255	255	407	50	255	255	255
408	1	255	255	255	408	7	255	255	255
409	5	255	255	255	409	9	255	255	255
410	2	255	255	255	410	34	255	255	255
411	8	255	255	255	411	8	255	255	255
412	1	255	255	255	412	4	255	255	255
413	1	255	255	255	413	1	255	255	255
414	1	255	255	255	414	37	255	255	255
415	44	255	255	255	415	1	255	255	255
416	1	255	255	255	416	1	255	255	255
417	2	255	255	255	417	1	255	255	255
418	2	255	255	255	418	1	255	255	255
419	2	255	255	255	419	33	255	255	255
420	3	255	255	255	420	95	255	255	255
421	2	255	255	255	421	26	255	255	255
422	24	255	255	255	422	3	255	255	255
423	3	255	255	255	423	1	255	255	255
424	98	255	255	255	424	14	255	255	255
425	4	255	255	255	425	1	255	255	255
426	6	255	255	255	426	11	255	255	255
427	34	255	255	255	427	1	255	255	255
428	3	255	255	255	428	1	255	255	255
429	34	255	255	255	429	2	255	255	255
430	8	255	255	255	430	1	255	255	255
431	114	255	255	255	431	3	255	255	255
432	2	255	255	255	432	60	255	255	255
433	54	255	255	255	433	59	255	255	255
434	25	255	255	255	434	1	255	255	255
435	150	255	255	255	435	1	255	255	255
436	1	255	255	255	436	22	255	255	255
437	32	255	255	255	437	1	255	255	255
438	41	255	255	255	438	2	255	255	255
439	1	255	255	255	439	4	255	255	255
440	3	255	255	255	440	6	255	255	255
441	24	255	255	255	441	8	255	255	255
442	136	255	255	255	442	1	255	255	255
443	1	255	255	255	443	102	255	255	255
444	12	255	255	255	444	87	255	255	255
445	73	255	255	255	445	47	255	255	255
446	181	255	255	255	446	1	255	255	255
447	47	255	255	255	447	97	255	255	255
448	3	255	255	255	448	4	255	255	255
449	82	255	255	255	449	131	255	255	255
450	1	255	255	255	450	9	255	255	255
451	24	255	255	255	451	1	255	255	255
452	64	255	255	255	452	177	255	255	255
453	12	255	255	255	453	2	255	255	255
454	169	255	255	255	454	1	255	255	255
455	62	255	255	255	455	1	255	255	255
456	2	255	255	255	456	1	255	255	255
457	12	255	255	255	457	1	255	255	255
458	3	255	255	255	458	2	255	255	255
459	3	255	255	255	459	1	255	255	255
460	1	255	255	255	460	1	255	255	255
461	66	255	255	255	461	19	255	255	255
462	1	255	255	255	462	86	255	255	255
463	1	255	255	255	463	1	255	255	255
464	1	255	255	255	464	4	255	255	255
465	55	255	255	255	465	1	255	255	255
466	61	255	255	255	466	11	255	255	255
467	69	255	255	255	467	10	255	255	255
468	1	255	255	255	468	40	255	255	255
469	16	255	255	255	469	80	255	255	255
470	1	255	255	255	470	2	255	255	255
471	1	255	255	255	471	21	255	255	255
472	79	255	255	255	472	3	255	255	255
473	2	255	255	255	473	1	255	255	255
474	4	255	255	255	474	1	255	255	255
475	17	255	255	255	475	1	255	255	255
476	3	255	255	255	476	1	255	255	255
477	2	255	255	255	477	1	255	255	255
478	22	255	255	255	478	4	255	255	255
479	2	255	255	255	479	29	255	255	255
480	168	255	255	255	480	2	255	255	255
481	44	255	255	255	481	2	255	255	255
482	65	255	255	255	482	1	255	255	255
483	14	255	255	255	483	4	255	255	255
484	2	255	255	255	484	15	255	255	255
485	1	255	255	255	485	9	255	255	255
486	1	255	255	255	486	19	255	255	255
487	2	255	255	255	487	21	255	255	255
488	2	255	255	255	488	7	255	255	255
489	20	255	255	255	489	2	255	255	255
490	78	255	255	255	490	2	255	255	255
491	2	255	255	255	491	72	255	255	255
492	46	255	255	255	492	3	255	255	255
493	97	255	255	255	493	18	255	255	255
494	30	255	255	255	494	22	255	255	255
495	1	255	255	255	495	17	255	255	255
496	116	255	255	255	496	25	255	255	255
497	1	255	255	255	497	1	255	255	255
498	2	255	255	255	498	1	255	255	255
499	5	255	255	255	499	2	255	255	255
500	14	255	255	255	500	19	255	255	255
501	5	255	255	255	501	5	255	255	255
502	715	255	255	255	502	1	255	255	255
503	19	255	255	255	503	1	255	255	255
504	13	255	255	255	504	15	255	255	255
505	3	255	255	255	505	3	255	255	255
506	2	255	255	255	506	26	255	255	255
507	1	255	255	255	507	16	255	255	255
508	1	255	255	255	508	2	255	255	255
509	1	255	255	255	509	2	255	255	255
510	5	255	255	255	510	21	255	255	255
511	1	255	255	255	511	7	255	255	255
512	1	255	255	255	512	1	255	255	255
513	1	255	255	255	513	1	255	255	255
514	1	255	255	255	514	1	255	255	255
515	3	255	255	255	515	27	255	255	255
516	3	255	255	255	516	8	255	255	255
517	8	255	255	255	517	2	255	255	255
518	6	255	255	255	518	1	255	255	255
519	1	255	255	255	519	1	255	255	255
520	1	255	255	255	520	2	255	255	255
521	32	255	255	255	521	4	255	255	255
522	1	255	255	255	522	1	255	255	255
523	1	255	255	255	523	20	255	255	255
524	5	255	255	255	524	4	255	255	255
525	36	255	255	255	525	5	255	255	255
526	1	255	255	255	526	142	255	255	255
527	410	255	255	255	527	1	255	255	255
528	1	255	255	255	528	10	255	255	255
529	1	255	255	255	529	119	255	255	255
530	77	255	255	255	530	1	255	255	255
531	40	255	255	255	531	1	255	255	255
532	3	255	255	255	532	6	255	255	255
533	4	255	255	255	533	29	255	255	255

Original data for Figure 2D, Densitometric analysis of Nuclear/Perinuclear B-catenin in control and EoE patient esophageal biopsy tissue.

Values are representative of the mean of a sample size N=18. p<0.05**

534	14	255	255	255	534	43	255	255	255
535	4	255	255	255	535	1	255	255	255
536	7	255	255	255	536	1	255	255	255
537	1	255	255	255	537	1	255	255	255
538	1	255	255	255	538	1	255	255	255
539	35	255	255	255	539	77	255	255	255
540	6	255	255	255	540	1	255	255	255
541	9	255	255	255	541	4	255	255	255
542	3	255	255	255	542	3	255	255	255
543	18	255	255	255	543	1	255	255	255
544	1	255	255	255	544	1	255	255	255
545	1	255	255	255	545	2	255	255	255
546	2	255	255	255	546	1	255	255	255
547	1	255	255	255	547	205	255	255	255
548	152	255	255	255	548	17	255	255	255
549	1	255	255	255	549	24	255	255	255
550	19	255	255	255	550	10	255	255	255
551	14	255	255	255	551	9	255	255	255
552	30	255	255	255	552	1	255	255	255
553	1	255	255	255	553	2	255	255	255
554	1	255	255	255	554	54	255	255	255
555	2	255	255	255	555	14	255	255	255
556	1	255	255	255	556	1	255	255	255
557	47	255	255	255	557	7	255	255	255
558	71	255	255	255	558	2	255	255	255
559	40	255	255	255	559	5	255	255	255
560	1	255	255	255	560	2	255	255	255
561	2	255	255	255	561	1	255	255	255
562	4	255	255	255	562	2	255	255	255
563	1	255	255	255	563	6	255	255	255
564	18	255	255	255	564	41	255	255	255
565	13	255	255	255	565	1	255	255	255
566	1	255	255	255	566	5	255	255	255
567	1	255	255	255	567	1	255	255	255
568	1	255	255	255	568	124	255	255	255
569	2	255	255	255	569	1	255	255	255
570	30	255	255	255	570	6	255	255	255
571	6	255	255	255	571	2	255	255	255
572	25	255	255	255	572	8	255	255	255
573	2	255	255	255	573	11	255	255	255
574	5	255	255	255	574	7	255	255	255
575	36	255	255	255	575	106	255	255	255
576	1	255	255	255	576	3	255	255	255
577	1	255	255	255	577	1	255	255	255
578	1	255	255	255	578	1	255	255	255
579	1	255	255	255	579	2	255	255	255
580	3	255	255	255	580	2	255	255	255
581	2	255	255	255	581	2	255	255	255
582	188	255	255	255	582	1	255	255	255
583	1	255	255	255	583	5	255	255	255
584	1	255	255	255	584	3	255	255	255
585	13	255	255	255	585	12	255	255	255
586	1	255	255	255	586	1	255	255	255
587	8	255	255	255	587	1	255	255	255
588	8	255	255	255	588	2	255	255	255
589	2	255	255	255	589	1	255	255	255
590	1	255	255	255	590	5	255	255	255
591	1	255	255	255	591	52	255	255	255
592	1	255	255	255	592	1	255	255	255
593	1	255	255	255	593	10	255	255	255
594	42	255	255	255	594	78	255	255	255
595	34	255	255	255	595	8	255	255	255
596	1	255	255	255	596	5	255	255	255
597	23	255	255	255	597	3	255	255	255
598	2	255	255	255	598	4	255	255	255
599	1	255	255	255	599	1	255	255	255
600	12	255	255	255	600	1	255	255	255
601	1	255	255	255	601	2	255	255	255
602	14	255	255	255	602	3	255	255	255
603	1	255	255	255	603	1	255	255	255
604	1	255	255	255	604	1	255	255	255
605	38	255	255	255	605	1	255	255	255
606	2	255	255	255	606	208	255	255	255
607	1	255	255	255	607	3	255	255	255
608	59	255	255	255	608	116	255	255	255
609	42	255	255	255	609	1	255	255	255
610	9	255	255	255	610	10	255	255	255
611	3	255	255	255	611	3	255	255	255
612	7	255	255	255	612	16	255	255	255
613	68	255	255	255	613	1	255	255	255
614	1	255	255	255	614	17	255	255	255
615	61	255	255	255	615	14	255	255	255
616	37	255	255	255	616	7	255	255	255
617	1	255	255	255	617	4	255	255	255
618	5	255	255	255	618	2	255	255	255
619	5	255	255	255	619	3	255	255	255
620	12	255	255	255	620	55	255	255	255
621	3	255	255	255	621	1	255	255	255
622	61	255	255	255	622	54	255	255	255
623	17	255	255	255	623	30	255	255	255
624	2	255	255	255	624	2	255	255	255
625	1	255	255	255	625	62	255	255	255
626	1	255	255	255	626	29	255	255	255
627	35	255	255	255	627	3	255	255	255
628	1	255	255	255	628	2	255	255	255
629	1	255	255	255	629	2	255	255	255
630	1	255	255	255	630	3	255	255	255
631	1	255	255	255	631	5	255	255	255
632	2	255	255	255	632	2	255	255	255
633	1	255	255	255	633	1	255	255	255
634	1	255	255	255	634	1	255	255	255
635	1	255	255	255	635	4	255	255	255
636	8	255	255	255	636	3	255	255	255
637	222	255	255	255	637	1	255	255	255
638	2	255	255	255	638	1	255	255	255
639	2	255	255	255	639	2	255	255	255
640	1	255	255	255	640	354	255	255	255
641	1	255	255	255	641	10	255	255	255
642	157	255	255	255	642	1	255	255	255
643	3	255	255	255	643	2	255	255	255
644	8	255	255	255	644	14	255	255	255
645	6	255	255	255	645	3	255	255	255
646	1	255	255	255	646	1	255	255	255
647	22	255	255	255	647	1	255	255	255
648	15	255	255	255	648	7	255	255	255
649	2	255	255	255	649	5	255	255	255
650	11	255	255	255	650	2	255	255	255
651	3	255	255	255	651	1	255	255	255
652	37	255	255	255	652	6	255	255	255
653	1	255	255	255	653	2	255	255	255
654	2	255	255	255	654	7	255	255	255
655	46	255	255	255	655	1	255	255	255
656	3	255	255	255	656	1	255	255	255
657	1	255	255	255	657	13	255	255	255
658	95	255	255	255	658	4	255	255	255
659	1	255	255	255	659	1	255	255	255
660	6	255	255	255	660	20	255	255	255
661	1	255	255	255	661	17	255	255	255
662	3	255	255	255	662	4	255	255	255
663	1	255	255	255	663	2	255	255	255
664	3	255	255	255	664	40	255	255	255
665	1	255	255	255	665	1	255	255	255
666	1	255	255	255	666	86	255	255	255
667	2	255	255	255	667	16	255	255	255
668	6	255	255	255	668	1	255	255	255
669	27	255	255	255	669	17	255	255	255
670	59	255	255	255	670	13	255	255	255
671	1	255	255	255	671	1	255	255	255

Original data for Figure 2D, Densitometric analysis of Nuclear/Perinuclear B-catenin in control and EoE patient esophageal biopsy tissue.

Values are representative of the mean of a sample size N=18. p<0.05**

672	1	255	255	255	672	1	255	255	255
673	6	255	255	255	673	4	255	255	255
674	1	255	255	255	674	4	255	255	255
675	54	255	255	255	675	3	255	255	255
676	21	255	255	255	676	3	255	255	255
677	2	255	255	255	677	86	255	255	255
678	1	255	255	255	678	7	255	255	255
679	5	255	255	255	679	2	255	255	255
680	1	255	255	255	680	18	255	255	255
681	1	255	255	255	681	2	255	255	255
682	1	255	255	255	682	66	255	255	255
683	1	255	255	255	683	1	255	255	255
684	56	255	255	255	684	1	255	255	255
685	2	255	255	255	685	2	255	255	255
686	3	255	255	255	686	1	255	255	255
687	1	255	255	255	687	3	255	255	255
688	336	255	255	255	688	26	255	255	255
689	2	255	255	255	689	44	255	255	255
690	12	255	255	255	690	1	255	255	255
691	1	255	255	255	691	1	255	255	255
692	8	255	255	255	692	14	255	255	255
693	72	255	255	255	693	9	255	255	255
694	1	255	255	255	694	1	255	255	255
695	144	255	255	255	695	1	255	255	255
696	77	255	255	255	696	17	255	255	255
697	3	255	255	255	697	3	255	255	255
698	2	255	255	255	698	3	255	255	255
699	1	255	255	255	699	6	255	255	255
700	1	255	255	255	700	7	255	255	255
701	1	255	255	255	701	1	255	255	255
702	13	255	255	255	702	2	255	255	255
703	28	255	255	255	703	24	255	255	255
704	1	255	255	255	704	1	255	255	255
705	174	255	255	255	705	2	255	255	255
706	336	255	255	255	706	3	255	255	255
707	1	255	255	255	707	1	255	255	255
708	1	255	255	255	708	19	255	255	255
709	50	255	255	255	709	1	255	255	255
710	1	255	255	255	710	1	255	255	255
711	77	255	255	255	711	2	255	255	255
712	1	255	255	255	712	2	255	255	255
713	41	255	255	255	713	1	255	255	255
714	6	255	255	255	714	9	255	255	255
715	1	255	255	255	715	1	255	255	255
716	1	255	255	255	716	24	255	255	255
717	38	255	255	255	717	60	255	255	255
718	3	255	255	255	718	2	255	255	255
719	1	255	255	255	719	2	255	255	255
720	128	255	255	255	720	1	255	255	255
721	60	255	255	255	721	1	255	255	255
722	1	255	255	255	722	5	255	255	255
723	2	255	255	255	723	255	255	255	255
724	3	255	255	255	724	21	255	255	255
725	9	255	255	255	725	1	255	255	255
726	2	255	255	255	726	1	255	255	255
727	1	255	255	255	727	42	255	255	255
728	46	255	255	255	728	17	255	255	255
729	23	255	255	255	729	1	255	255	255
730	1	255	255	255	730	1	255	255	255
731	2	255	255	255	731	1	255	255	255
732	84	255	255	255	732	1	255	255	255
733	102	255	255	255	733	1	255	255	255
734	19	255	255	255	734	42	255	255	255
735	36	255	255	255	735	112	255	255	255
736	3	255	255	255	736	1	255	255	255
737	40	255	255	255	737	4	255	255	255
738	1	255	255	255	738	2	255	255	255
739	35	255	255	255	739	28	255	255	255
740	1	255	255	255	740	48	255	255	255
741	2	255	255	255	741	1	255	255	255
742	1	255	255	255	742	24	255	255	255
743	6	255	255	255	743	13	255	255	255
744	91	255	255	255	744	25	255	255	255
745	1	255	255	255	745	9	255	255	255
746	1	255	255	255	746	1	255	255	255
747	2	255	255	255	747	1	255	255	255
748	1	255	255	255	748	2	255	255	255
749	40	255	255	255	749	56	255	255	255
750	30	255	255	255	750	25	255	255	255
751	1	255	255	255	751	29	255	255	255
752	1	255	255	255	752	1	255	255	255
753	12	255	255	255	753	3	255	255	255
754	1	255	255	255	754	1	255	255	255
755	2	255	255	255	755	1	255	255	255
756	2	255	255	255	756	3	255	255	255
757	2	255	255	255	757	6	255	255	255
758	20	255	255	255	758	5	255	255	255
759	36	255	255	255	759	1	255	255	255
760	225	255	255	255	760	3	255	255	255
761	5	255	255	255	761	1	255	255	255
762	2	255	255	255	762	3	255	255	255
763	43	255	255	255	763	161	255	255	255
764	7	255	255	255	764	1	255	255	255
765	79	255	255	255	765	1	255	255	255
766	2	255	255	255	766	30	255	255	255
767	4	255	255	255	767	1	255	255	255
768	58	255	255	255	768	1	255	255	255
769	93	255	255	255	769	1	255	255	255
770	1	255	255	255	770	3	255	255	255
771	9	255	255	255	771	1	255	255	255
772	3	255	255	255	772	29	255	255	255
773	8	255	255	255	773	4	255	255	255
774	12	255	255	255	774	1	255	255	255
775	61	255	255	255	775	1	255	255	255
776	2	255	255	255	776	2	255	255	255
777	2	255	255	255	777	1	255	255	255
778	36	255	255	255	778	1	255	255	255
779	5	255	255	255	779	1	255	255	255
780	1	255	255	255	780	18	255	255	255
781	2	255	255	255	781	44	255	255	255
782	1	255	255	255	782	15	255	255	255
783	1	255	255	255	783	3	255	255	255
784	44	255	255	255	784	1	255	255	255
785	1	255	255	255	785	1	255	255	255
786	37	255	255	255	786	2	255	255	255
787	5	255	255	255	787	2	255	255	255
788	2	255	255	255	788	4	255	255	255
789	2	255	255	255	789	21	255	255	255
790	11200	255	255	255	790	31	255	255	255
791	6	255	255	255	791	5	255	255	255
792	1	255	255	255	792	1	255	255	255
793	1	255	255	255	793	32	255	255	255
794	19	255	255	255	794	3	255	255	255
795	1	255	255	255	795	1	255	255	255
796	99	255	255	255	796	60	255	255	255
797	3	255	255	255	797	8	255	255	255
798	1	255	255	255	798	1	255	255	255
799	4	255	255	255	799	1	255	255	255
800	3	255	255	255	800	1	255	255	255
801	6	255	255	255	801	2	255	255	255
802	1	255	255	255	802	1	255	255	255
803	2	255	255	255	803	1	255	255	255
804	37	255	255	255	804	15	255	255	255
805	1	255	255	255	805	12	255	255	255
806	2	255	255	255	806	1	255	255	255
807	211	255	255	255	807	19	255	255	255
808	1	255	255	255	808	2	255	255	255
809	6	255	255	255	809	3	255	255	255

Original data for Figure 2D, Densitometric analysis of Nuclear/Perinuclear B-catenin in control and EoE patient esophageal biopsy tissue.
Values are representative of the mean of a sample size N=18. p<0.05**

810	11	255	255	255	810	1	255	255	255
811	3	255	255	255	811	2	255	255	255
812	15	255	255	255	812	1	255	255	255
813	1	255	255	255	813	15	255	255	255
814	54	255	255	255	814	1	255	255	255
815	68	255	255	255	815	1	255	255	255
816	6	255	255	255	816	106	255	255	255
817	1	255	255	255	817	1	255	255	255
818	19	255	255	255	818	22	255	255	255
819	1	255	255	255	819	44	255	255	255
820	49	255	255	255	820	1	255	255	255
821	1	255	255	255	821	3	255	255	255
822	2	255	255	255	822	17	255	255	255
823	30	255	255	255	823	28	255	255	255
824	2	255	255	255	824	7	255	255	255
825	63	255	255	255	825	4	255	255	255
826	102	255	255	255	826	10	255	255	255
827	14	255	255	255	827	3	255	255	255
828	2	255	255	255	828	4	255	255	255
829	3	255	255	255	829	61	255	255	255
830	1	255	255	255	830	53	255	255	255
831	26	255	255	255	831	1	255	255	255
832	2	255	255	255	832	2	255	255	255
833	53	255	255	255	833	3	255	255	255
834	19	255	255	255	834	1	255	255	255
835	1	255	255	255	835	99	255	255	255
836	26	255	255	255	836	14	255	255	255
837	92	255	255	255	837	22	255	255	255
838	17	255	255	255	838	5	255	255	255
839	1	255	255	255	839	1	255	255	255
840	61	255	255	255	840	6	255	255	255
841	17	255	255	255	841	1	255	255	255
842	2	255	255	255	842	15	255	255	255
843	36	255	255	255	843	1	255	255	255
844	12	255	255	255	844	2	255	255	255
845	6	255	255	255	845	1	255	255	255
846	1	255	255	255	846	3	255	255	255
847	34	255	255	255	847	46	255	255	255
848	2	255	255	255	848	2	255	255	255
849	2	255	255	255	849	2	255	255	255
850	64	255	255	255	850	1	255	255	255
851	1	255	255	255	851	27	255	255	255
852	1	255	255	255	852	1	255	255	255
853	7	255	255	255	853	69	255	255	255
854	1	255	255	255	854	2	255	255	255
855	18	255	255	255	855	34	255	255	255
856	1	255	255	255	856	27	255	255	255
857	5	255	255	255	857	2	255	255	255
858	2	255	255	255	858	1	255	255	255
859	1	255	255	255	859	27	255	255	255
860	1	255	255	255	860	2	255	255	255
861	76	255	255	255	861	1	255	255	255
862	2	255	255	255	862	32	255	255	255
863	1	255	255	255	863	54	255	255	255
864	2	255	255	255	864	1	255	255	255
865	1	255	255	255	865	1	255	255	255
866	108	255	255	255	866	1	255	255	255
867	25	255	255	255	867	2	255	255	255
868	1	255	255	255	868	1	255	255	255
869	49	255	255	255	869	1	255	255	255
870	50	255	255	255	870	1	255	255	255
871	14	255	255	255	871	2	255	255	255
872	2	255	255	255	872	2	255	255	255
873	28	255	255	255	873	60	255	255	255
874	6	255	255	255	874	10	255	255	255
875	60	255	255	255	875	56	255	255	255
876	59	255	255	255	876	9	255	255	255
877	2	255	255	255	877	4	255	255	255
878	3	255	255	255	878	15	255	255	255
879	1	255	255	255	879	1	255	255	255
880	15	255	255	255	880	3	255	255	255
881	2	255	255	255	881	1	255	255	255
882	3	255	255	255	882	1	255	255	255
883	133	255	255	255	883	148	255	255	255
884	2	255	255	255	884	7	255	255	255
885	1	255	255	255	885	2	255	255	255
886	1	255	255	255	886	38	255	255	255
887	20	255	255	255	887	1	255	255	255
888	23	255	255	255	888	2	255	255	255
889	2	255	255	255	889	2	255	255	255
890	2	255	255	255	890	7	255	255	255
891	1	255	255	255	891	4	255	255	255
892	1	255	255	255	892	3	255	255	255
893	1	255	255	255	893	5	255	255	255
894	1	255	255	255	894	4	255	255	255
895	1	255	255	255	895	8	255	255	255
896	1	255	255	255	896	1	255	255	255
897	2	255	255	255	897	5	255	255	255
898	142	255	255	255	898	1	255	255	255
899	1	255	255	255	899	1	255	255	255
900	1	255	255	255	900	3	255	255	255
901	9	255	255	255	901	10	255	255	255
902	1	255	255	255	902	7	255	255	255
903	6	255	255	255	903	2	255	255	255
904	1	255	255	255	904	15	255	255	255
905	10	255	255	255	905	19	255	255	255
906	1	255	255	255	906	1	255	255	255
907	56	255	255	255	907	6	255	255	255
908	2	255	255	255	908	2	255	255	255
909	1	255	255	255	909	1	255	255	255
910	1	255	255	255	910	2	255	255	255
911	1	255	255	255	911	3	255	255	255
912	1	255	255	255	912	1	255	255	255
913	2	255	255	255	913	2	255	255	255
914	2	255	255	255	914	1	255	255	255
915	1	255	255	255	915	15	255	255	255
916	1	255	255	255	916	24	255	255	255
917	42	255	255	255	917	2	255	255	255
918	1	255	255	255	918	2	255	255	255
919	3	255	255	255	919	1	255	255	255
920	1	255	255	255	920	3	255	255	255
921	1	255	255	255	921	1	255	255	255
922	1	255	255	255	922	23	255	255	255
923	2	255	255	255	923	1	255	255	255
924	1	255	255	255	924	11	255	255	255
925	5	255	255	255	925	1	255	255	255
926	1	255	255	255	926	16	255	255	255
927	5	255	255	255	927	1	255	255	255
928	1	255	255	255	928	1	255	255	255
929	1	255	255	255	929	2	255	255	255
930	1	255	255	255	930	2	255	255	255
931	18	255	255	255	931	11	255	255	255
932	1	255	255	255	932	5	255	255	255
933	2	255	255	255	933	10	255	255	255
934	2	255	255	255	934	15	255	255	255
935	19	255	255	255	935	1	255	255	255
936	36	255	255	255	936	1	255	255	255
937	1	255	255	255	937	22	255	255	255
938	5	255	255	255	938	1	255	255	255
939	29	255	255	255	939	2	255	255	255
940	1	255	255	255	940	1	255	255	255
941	20	255	255	255	941	5	255	255	255
942	1	255	255	255	942	7	255	255	255
943	19	255	255	255	943	1	255	255	255
944	12	255	255	255	944	1	255	255	255
945	27	255	255	255	945	73	255	255	255
946	1	255	255	255	946	5	255	255	255
947	2	255	255	255	947	1	255	255	255

Original data for Figure 2D, Densitometric analysis of Nuclear/Perinuclear
B-catenin in control and EoE patient esophageal biopsy tissue.
Values are representative of the mean of a sample size N=18. p<0.05**

948	1	255	255	255	948	1	255	255	255
949	47	255	255	255	949	1	255	255	255
950	8	255	255	255	950	6	255	255	255
951	1	255	255	255	951	16	255	255	255
952	64	255	255	255	952	12	255	255	255
953	5	255	255	255	953	5	255	255	255
954	2	255	255	255	954	5	255	255	255
955	2	255	255	255	955	1	255	255	255
956	1	255	255	255	956	2	255	255	255
957	101	255	255	255	957	129	255	255	255
958	2	255	255	255	958	1	255	255	255
959	4	255	255	255	959	1	255	255	255
960	1	255	255	255	960	18	255	255	255
961	29	255	255	255	961	2	255	255	255
962	92	255	255	255	962	3	255	255	255
963	76	255	255	255	963	2	255	255	255
964	102	255	255	255	964	6	255	255	255
965	7	255	255	255	965	1	255	255	255
966	2	255	255	255	966	3	255	255	255
967	2	255	255	255	967	4	255	255	255
968	19	255	255	255	968	2	255	255	255
969	4	255	255	255	969	46	255	255	255
970	2	255	255	255	970	1	255	255	255
971	5	255	255	255	971	4	255	255	255
972	1	255	255	255	972	9	255	255	255
973	1	255	255	255	973	1	255	255	255
974	7	255	255	255	974	2	255	255	255
975	2	255	255	255	975	35	255	255	255
976	1	255	255	255	976	329	255	255	255
977	11	255	255	255	977	6	255	255	255
978	2	255	255	255	978	13	255	255	255
979	22	255	255	255	979	3	255	255	255
980	13	255	255	255	980	3	255	255	255
981	65	255	255	255	981	3	255	255	255
982	72	255	255	255	982	3	255	255	255
983	101	255	255	255	983	2	255	255	255
984	1	255	255	255	984	1	255	255	255
985	13	255	255	255	985	1	255	255	255
986	1	255	255	255	986	1	255	255	255
987	6	255	255	255	987	12	255	255	255
988	1	255	255	255	988	45	255	255	255
989	3	255	255	255	989	3	255	255	255
990	9	255	255	255	990	1	255	255	255
991	1	255	255	255	991	46	255	255	255
992	4	255	255	255	992	29	255	255	255
993	4	255	255	255	993	64	255	255	255
994	1	255	255	255	994	5	255	255	255
995	78	255	255	255	995	8	255	255	255
996	47	255	255	255	996	6	255	255	255
997	31	255	255	255	997	1	255	255	255
998	8	255	255	255	998	12	255	255	255
999	5	255	255	255	999	12	255	255	255
1000	9	255	255	255	1000	45	255	255	255
1001	88	255	255	255	1001	4	255	255	255
1002	1	255	255	255	1002	2	255	255	255
1003	1	255	255	255	1003	17	255	255	255
1004	4	255	255	255	1004	4	255	255	255
1005	52	255	255	255	1005	44	255	255	255
1006	42	255	255	255	1006	1	255	255	255
1007	1	255	255	255	1007	2	255	255	255
1008	1	255	255	255	1008	194	255	255	255
1009	4	255	255	255	1009	1	255	255	255
1010	18	255	255	255	1010	20	255	255	255
1011	9	255	255	255	1011	14	255	255	255
1012	4	255	255	255	1012	1	255	255	255
1013	1	255	255	255	1013	1	255	255	255
1014	6	255	255	255	1014	1	255	255	255
1015	228	255	255	255	1015	124	255	255	255
1016	2	255	255	255	1016	1	255	255	255
1017	1	255	255	255	1017	14	255	255	255
1018	45	255	255	255	1018	10	255	255	255
1019	1	255	255	255	1019	1	255	255	255
1020	1	255	255	255	1020	1	255	255	255
1021	1	255	255	255	1021	1	255	255	255
1022	2	255	255	255	1022	122	255	255	255
1023	28	255	255	255	1023	49	255	255	255
1024	3	255	255	255	1024	2	255	255	255
1025	3	255	255	255	1025	48	255	255	255
1026	7	255	255	255	1026	1	255	255	255
1027	1	255	255	255	1027	1	255	255	255
1028	1494	255	255	255	1028	3	255	255	255
1029	8	255	255	255	1029	30	255	255	255
1030	1	255	255	255	1030	1	255	255	255
1031	2	255	255	255	1031	1	255	255	255
1032	51	255	255	255	1032	2	255	255	255
1033	109	255	255	255	1033	30	255	255	255
1034	1	255	255	255	1034	27	255	255	255
1035	2	255	255	255	1035	1	255	255	255
1036	32	255	255	255	1036	4	255	255	255
1037	1	255	255	255	1037	2	255	255	255
1038	1	255	255	255	1038	21	255	255	255
1039	114	255	255	255	1039	20	255	255	255
1040	1	255	255	255	1040	3	255	255	255
1041	2	255	255	255	1041	1	255	255	255
1042	25	255	255	255	1042	1	255	255	255
1043	9	255	255	255	1043	6	255	255	255
1044	1	255	255	255	1044	8	255	255	255
1045	2	255	255	255	1045	1	255	255	255
1046	1	255	255	255	1046	27	255	255	255
1047	4	255	255	255	1047	1	255	255	255
1048	1	255	255	255	1048	1	255	255	255
1049	2	255	255	255	1049	1	255	255	255
1050	6	255	255	255	1050	1	255	255	255
1051	3	255	255	255	1051	2	255	255	255
1052	59	255	255	255	1052	11	255	255	255
1053	17	255	255	255	1053	15	255	255	255
1054	37	255	255	255	1054	2	255	255	255
1055	1	255	255	255	1055	3	255	255	255
1056	2	255	255	255	1056	2	255	255	255
1057	9	255	255	255	1057	10	255	255	255
1058	19	255	255	255	1058	28	255	255	255
1059	2	255	255	255	1059	1	255	255	255
1060	4	255	255	255	1060	4	255	255	255
1061	1	255	255	255	1061	44	255	255	255
1062	7	255	255	255	1062	1	255	255	255
1063	4	255	255	255	1063	2	255	255	255
1064	2	255	255	255	1064	13	255	255	255
1065	14	255	255	255	1065	25	255	255	255
1066	4	255	255	255	1066	1	255	255	255
1067	1	255	255	255	1067	1	255	255	255
1068	2	255	255	255	1068	63	255	255	255
1069	17	255	255	255	1069	28	255	255	255
1070	15	255	255	255	1070	8	255	255	255
1071	9	255	255	255	1071	6	255	255	255
1072	1	255	255	255	1072	45	255	255	255
1073	8	255	255	255	1073	2	255	255	255
1074	4	255	255	255	1074	1	255	255	255
1075	28	255	255	255	1075	1	255	255	255
1076	5	255	255	255	1076	3	255	255	255
1077	1	255	255	255	1077	1	255	255	255
1078	11	255	255	255	1078	1	255	255	255
1079	2	255	255	255	1079	37	255	255	255
1080	170	255	255	255	1080	61	255	255	255
1081	1	255	255	255	1081	12	255	255	255
1082	1	255	255	255	1082	23	255	255	255
1083	1	255	255	255	1083	1	255	255	255
1084	118	255	255	255	1084	20	255	255	255
1085	9	255	255	255	1085	34	255	255	255

Original data for Figure 2D, Densitometric analysis of Nuclear/Perinuclear B-catenin in control and EoE patient esophageal biopsy tissue.

Values are representative of the mean of a sample size N=18. p<0.05**

1086	45	255	255	255	1086	1	255	255	255
1087	2	255	255	255	1087	1	255	255	255
1088	2	255	255	255	1088	1	255	255	255
1089	418	255	255	255	1089	14	255	255	255
1090	2	255	255	255	1090	2	255	255	255
1091	2	255	255	255	1091	1	255	255	255
1092	2	255	255	255	1092	1	255	255	255
1093	230	255	255	255	1093	1	255	255	255
1094	2	255	255	255	1094	4	255	255	255
1095	1	255	255	255	1095	1	255	255	255
1096	5	255	255	255	1096	1	255	255	255
1097	6	255	255	255	1097	1	255	255	255
1098	6	255	255	255	1098	30	255	255	255
1099	1	255	255	255	1099	17	255	255	255
1100	1	255	255	255	1100	2	255	255	255
1101	2	255	255	255	1101	29	255	255	255
1102	65	255	255	255	1102	6	255	255	255
1103	287	255	255	255	1103	1	255	255	255
1104	1	255	255	255	1104	1	255	255	255
1105	2	255	255	255	1105	78	255	255	255
1106	2	255	255	255	1106	21	255	255	255
1107	1	255	255	255	1107	133	255	255	255
1108	2	255	255	255	1108	8	255	255	255
1109	6	255	255	255	1109	1	255	255	255
1110	8	255	255	255	1110	7	255	255	255
1111	13	255	255	255	1111	62	255	255	255
1112	119	255	255	255	1112	6	255	255	255
1113	23	255	255	255	1113	3	255	255	255
1114	1	255	255	255	1114	1	255	255	255
1115	41	255	255	255	1115	4	255	255	255
1116	9	255	255	255	1116	2	255	255	255
1117	1	255	255	255	1117	219	255	255	255
1118	6	255	255	255	1118	2	255	255	255
1119	2	255	255	255	1119	1	255	255	255
1120	19	255	255	255	1120	1	255	255	255
1121	1	255	255	255	1121	3	255	255	255
1122	16	255	255	255	1122	13	255	255	255
1123	1	255	255	255	1123	2	255	255	255
1124	7	255	255	255	1124	4	255	255	255
1125	5	255	255	255	1125	60	255	255	255
1126	1	255	255	255	1126	1	255	255	255
1127	1	255	255	255	1127	1	255	255	255
1128	1	255	255	255	1128	37	255	255	255
1129	1	255	255	255	1129	8	255	255	255
1130	3	255	255	255	1130	1	255	255	255
1131	3	255	255	255	1131	1	255	255	255
1132	1	255	255	255	1132	18	255	255	255
1133	1	255	255	255	1133	1	255	255	255
1134	9	255	255	255	1134	186	255	255	255
1135	44	255	255	255	1135	1	255	255	255
1136	85	255	255	255	1136	1	255	255	255
1137	1	255	255	255	1137	1	255	255	255
1138	5	255	255	255	1138	3	255	255	255
1139	4	255	255	255	1139	3	255	255	255
1140	1	255	255	255	1140	1	255	255	255
1141	1	255	255	255	1141	3	255	255	255
1142	2	255	255	255	1142	1	255	255	255
1143	22	255	255	255	1143	7	255	255	255
1144	17	255	255	255	1144	1	255	255	255
1145	18	255	255	255	1145	68	255	255	255
1146	3	255	255	255	1146	41	255	255	255
1147	1	255	255	255	1147	43	255	255	255
1148	100	255	255	255	1148	26	255	255	255
1149	35	255	255	255	1149	1	255	255	255
1150	1	255	255	255	1150	3	255	255	255
1151	1	255	255	255	1151	2	255	255	255
1152	1	255	255	255	1152	117	255	255	255
1153	1	255	255	255	1153	2	255	255	255
1154	7	255	255	255	1154	1	255	255	255
1155	1	255	255	255	1155	2	255	255	255
1156	48	255	255	255	1156	2	255	255	255
1157	41	255	255	255	1157	2	255	255	255
1158	7	255	255	255	1158	6	255	255	255
1159	81	255	255	255	1159	1	255	255	255
1160	83	255	255	255	1160	1	255	255	255
1161	7	255	255	255	1161	1	255	255	255
1162	18	255	255	255	1162	1	255	255	255
1163	2	255	255	255	1163	2	255	255	255
1164	1	255	255	255	1164	1	255	255	255
1165	1	255	255	255	1165	10	255	255	255
1166	2	255	255	255	1166	14	255	255	255
1167	1	255	255	255	1167	2	255	255	255
1168	45	255	255	255	1168	2	255	255	255
1169	47	255	255	255	1169	2	255	255	255
1170	2	255	255	255	1170	1	255	255	255
1171	72	255	255	255	1171	5	255	255	255
1172	71	255	255	255	1172	27	255	255	255
1173	4	255	255	255	1173	1	255	255	255
1174	1	255	255	255	1174	5	255	255	255
1175	1	255	255	255	1175	8	255	255	255
1176	1	255	255	255	1176	11	255	255	255
1177	1	255	255	255	1177	1	255	255	255
1178	157	255	255	255	1178	4	255	255	255
1179	1	255	255	255	1179	2	255	255	255
1180	3	255	255	255	1180	9	255	255	255
1181	7	255	255	255	1181	33	255	255	255
1182	2	255	255	255	1182	4	255	255	255
1183	1	255	255	255	1183	6	255	255	255
1184	11	255	255	255	1184	2	255	255	255
1185	101	255	255	255	1185	1	255	255	255
1186	84	255	255	255	1186	3	255	255	255
1187	1	255	255	255	1187	2	255	255	255
1188	1	255	255	255	1188	39	255	255	255
1189	12	255	255	255	1189	3	255	255	255
1190	2	255	255	255	1190	4	255	255	255
1191	2	255	255	255	1191	5	255	255	255
1192	1	255	255	255	1192	25	255	255	255
1193	73	255	255	255	1193	10	255	255	255
1194	1	255	255	255	1194	1	255	255	255
1195	1	255	255	255	1195	134	255	255	255
1196	16	255	255	255	1196	1	255	255	255
1197	1	255	255	255	1197	17	255	255	255
1198	1	255	255	255	1198	8	255	255	255
1199	73	255	255	255	1199	15	255	255	255
1200	1	255	255	255	1200	50	255	255	255
1201	7	255	255	255	1201	1	255	255	255
1202	2	255	255	255	1202	2	255	255	255
1203	1	255	255	255	1203	9	255	255	255
1204	30	255	255	255	1204	18	255	255	255
1205	1	255	255	255	1205	4	255	255	255
1206	41	255	255	255	1206	42	255	255	255
1207	6	255	255	255	1207	2	255	255	255
1208	9	255	255	255	1208	5	255	255	255
1209	18	255	255	255	1209	1	255	255	255
1210	7	255	255	255	1210	1	255	255	255
1211	24	255	255	255	1211	178	255	255	255
1212	32	255	255	255	1212	1	255	255	255
1213	29	255	255	255	1213	45	255	255	255
1214	2	255	255	255	1214	2	255	255	255
1215	1	255	255	255	1215	1	255	255	255
1216	1	255	255	255	1216	6	255	255	255
1217	2	255	255	255	1217	242	255	255	255
1218	2	255	255	255	1218	2	255	255	255
1219	42	255	255	255	1219	17	255	255	255
1220	7	255	255	255	1220	2	255	255	255
1221	11	255	255	255	1221	1	255	255	255
1222	34	255	255	255	1222	2	255	255	255
1223	21	255	255	255	1223	1	255	255	255

Original data for Figure 2D, Densitometric analysis of Nuclear/Perinuclear
B-catenin in control and EoE patient esophageal biopsy tissue.
Values are representative of the mean of a sample size N=18. p<0.05**

1224	10	255	255	255	1224	3	255	255	255
1225	52	255	255	255	1225	1	255	255	255
1226	8	255	255	255	1226	4	255	255	255
1227	6	255	255	255	1227	1	255	255	255
1228	3	255	255	255	1228	3	255	255	255
1229	9	255	255	255	1229	4	255	255	255
1230	1	255	255	255	1230	2	255	255	255
1231	1	255	255	255	1231	4	255	255	255
1232	93	255	255	255	1232	16	255	255	255
1233	41	255	255	255	1233	2	255	255	255
1234	619	255	255	255	1234	28	255	255	255
1235	1	255	255	255	1235	77	255	255	255
1236	411	255	255	255	1236	1	255	255	255
1237	8	255	255	255	1237	1	255	255	255
1238	29	255	255	255	1238	6	255	255	255
1239	93	255	255	255	1239	5	255	255	255
1240	1103	255	255	255	1240	13	255	255	255
1241	10	255	255	255	1241	1	255	255	255
1242	1	255	255	255	1242	34	255	255	255
1243	1	255	255	255	1243	2	255	255	255
1244	2	255	255	255	1244	2	255	255	255
1245	3	255	255	255	1245	2	255	255	255
1246	1	255	255	255	1246	5	255	255	255
1247	4	255	255	255	1247	1	255	255	255
1248	50	255	255	255	1248	1	255	255	255
1249	1	255	255	255	1249	26	255	255	255
1250	1	255	255	255	1250	2	255	255	255
1251	32	255	255	255	1251	3	255	255	255
1252	4	255	255	255	1252	6	255	255	255
1253	48	255	255	255	1253	12	255	255	255
1254	4	255	255	255	1254	2	255	255	255
1255	27	255	255	255	1255	105	255	255	255
1256	1	255	255	255	1256	46	255	255	255
1257	3	255	255	255	1257	1	255	255	255
1258	4	255	255	255	1258	2	255	255	255
1259	13	255	255	255	1259	1	255	255	255
1260	5	255	255	255	1260	1	255	255	255
1261	2	255	255	255	1261	76	255	255	255
1262	2	255	255	255	1262	3	255	255	255
1263	73	255	255	255	1263	2	255	255	255
1264	43	255	255	255	1264	2	255	255	255
1265	1	255	255	255	1265	1	255	255	255
1266	1	255	255	255	1266	43	255	255	255
1267	1	255	255	255	1267	16	255	255	255
1268	1	255	255	255	1268	1	255	255	255
1269	1	255	255	255	1269	8	255	255	255
1270	6	255	255	255	1270	1	255	255	255
1271	423	255	255	255	1271	8	255	255	255
1272	1	255	255	255	1272	31	255	255	255
1273	45	255	255	255	1273	1	255	255	255
1274	118	255	255	255	1274	54	255	255	255
1275	1	255	255	255	1275	4	255	255	255
1276	40	255	255	255	1276	2	255	255	255
1277	29	255	255	255	1277	1	255	255	255
1278	240	255	255	255	1278	30	255	255	255
1279	28	255	255	255	1279	22	255	255	255
1280	1	255	255	255	1280	1	255	255	255
1281	1	255	255	255	1281	11	255	255	255
1282	7	255	255	255	1282	58	255	255	255
1283	50	255	255	255	1283	1	255	255	255
1284	1	255	255	255	1284	35	255	255	255
1285	2	255	255	255	1285	32	255	255	255
1286	1	255	255	255	1286	16	255	255	255
1287	1	255	255	255	1287	1	255	255	255
1288	4	255	255	255	1288	18	255	255	255
1289	178	255	255	255	1289	1	255	255	255
1290	9	255	255	255	1290	48	255	255	255
1291	293	255	255	255	1291	6	255	255	255
1292	2	255	255	255	1292	1	255	255	255
1293	1	255	255	255	1293	45	255	255	255
1294	1	255	255	255	1294	5	255	255	255
1295	8	255	255	255	1295	38	255	255	255
1296	2	255	255	255	1296	31	255	255	255
1297	6	255	255	255	1297	3	255	255	255
1298	1	255	255	255	1298	5	255	255	255
1299	1	255	255	255	1299	33	255	255	255
1300	88	255	255	255	1300	4	255	255	255
1301	3	255	255	255	1301	6	255	255	255
1302	1	255	255	255	1302	16	255	255	255
1303	1	255	255	255	1303	3	255	255	255
1304	1	255	255	255	1304	1	255	255	255
1305	1	255	255	255	1305	4	255	255	255
1306	1	255	255	255	1306	6	255	255	255
1307	9	255	255	255	1307	1	255	255	255
1308	3	255	255	255	1308	1	255	255	255
1309	1	255	255	255	1309	2	255	255	255
1310	24	255	255	255	1310	71	255	255	255
1311	1	255	255	255	1311	1	255	255	255
1312	1	255	255	255	1312	3	255	255	255
1313	2	255	255	255	1313	1	255	255	255
1314	13	255	255	255	1314	1	255	255	255
1315	2	255	255	255	1315	7	255	255	255
1316	40	255	255	255	1316	21	255	255	255
1317	1	255	255	255	1317	4	255	255	255
1318	36	255	255	255	1318	5	255	255	255
1319	1	255	255	255	1319	15	255	255	255
1320	1	255	255	255	1320	6	255	255	255
1321	1	255	255	255	1321	3	255	255	255
1322	3	255	255	255	1322	1	255	255	255
1323	3	255	255	255	1323	30	255	255	255
1324	53	255	255	255	1324	2	255	255	255
1325	1	255	255	255	1325	13	255	255	255
1326	1	255	255	255	1326	1	255	255	255
1327	2	255	255	255	1327	12	255	255	255
1328	281	255	255	255	1328	2	255	255	255
1329	4	255	255	255	1329	2	255	255	255
1330	1	255	255	255	1330	1	255	255	255
1331	1	255	255	255	1331	1	255	255	255
1332	2	255	255	255	1332	6	255	255	255
1333	1	255	255	255	1333	21	255	255	255
1334	1	255	255	255	1334	16	255	255	255
1335	28	255	255	255	1335	17	255	255	255
1336	4	255	255	255	1336	1	255	255	255
1337	431	255	255	255	1337	32	255	255	255
1338	14	255	255	255	1338	2	255	255	255
1339	2	255	255	255	1339	2	255	255	255
1340	108	255	255	255	1340	39	255	255	255
1341	15	255	255	255	1341	1	255	255	255
1342	2	255	255	255	1342	2	255	255	255
1343	4	255	255	255	1343	2	255	255	255
1344	1	255	255	255	1344	17	255	255	255
1345	2	255	255	255	1345	17	255	255	255
1346	1	255	255	255	1346	122	255	255	255
1347	2	255	255	255	1347	14	255	255	255
1348	70	255	255	255	1348	1	255	255	255
1349	4	255	255	255	1349	3	255	255	255
1350	1	255	255	255	1350	1	255	255	255
1351	18	255	255	255	1351	12	255	255	255
1352	2	255	255	255	1352	2	255	255	255
1353	1	255	255	255	1353	36	255	255	255
1354	24	255	255	255	1354	1	255	255	255
1355	1	255	255	255	1355	1	255	255	255
1356	1	255	255	255	1356	3	255	255	255
1357	1	255	255	255	1357	18	255	255	255
1358	39	255	255	255	1358	1	255	255	255
1359	1	255	255	255	1359	2	255	255	255
1360	3	255	255	255	1360	1	255	255	255
1361	1	255	255	255	1361	17	255	255	255

Original data for Figure 2D, Densitometric analysis of Nuclear/Perinuclear B-catenin in control and EoE patient esophageal biopsy tissue.

Values are representative of the mean of a sample size N=18. p<0.05**

1362	26	255	255	255	1362	2	255	255	255
1363	20	255	255	255	1363	38	255	255	255
1364	1	255	255	255	1364	4	255	255	255
1365	13	255	255	255	1365	30	255	255	255
1366	2	255	255	255	1366	23	255	255	255
1367	109	255	255	255	1367	9	255	255	255
1368	1	255	255	255	1368	17	255	255	255
1369	6	255	255	255	1369	1	255	255	255
1370	4	255	255	255	1370	19	255	255	255
1371	1	255	255	255	1371	11	255	255	255
1372	1	255	255	255	1372	17	255	255	255
1373	2	255	255	255	1373	2	255	255	255
1374	1	255	255	255	1374	10	255	255	255
1375	78	255	255	255	1375	2	255	255	255
1376	2	255	255	255	1376	190	255	255	255
1377	1	255	255	255	1377	1	255	255	255
1378	5	255	255	255	1378	1	255	255	255
1379	5	255	255	255	1379	30	255	255	255
1380	10	255	255	255	1380	4	255	255	255
1381	2	255	255	255	1381	2	255	255	255
1382	3	255	255	255	1382	28	255	255	255
1383	15	255	255	255	1383	17	255	255	255
1384	88	255	255	255	1384	3	255	255	255
1385	60	255	255	255	1385	3	255	255	255
1386	2	255	255	255	1386	4	255	255	255
1387	3	255	255	255	1387	34	255	255	255
1388	2	255	255	255	1388	1	255	255	255
1389	1	255	255	255	1389	8	255	255	255
1390	56	255	255	255	1390	1	255	255	255
1391	3	255	255	255	1391	1	255	255	255
1392	1	255	255	255	1392	30	255	255	255
1393	6	255	255	255	1393	9	255	255	255
1394	3	255	255	255	1394	34	255	255	255
1395	13	255	255	255	1395	1	255	255	255
1396	5	255	255	255	1396	45	255	255	255
1397	2	255	255	255	1397	1	255	255	255
1398	2	255	255	255	1398	8	255	255	255
1399	54	255	255	255	1399	2	255	255	255
1400	315	255	255	255	1400	1	255	255	255
1401	2	255	255	255	1401	2	255	255	255
1402	46	255	255	255	1402	19	255	255	255
1403	1	255	255	255	1403	1	255	255	255
1404	1	255	255	255	1404	3	255	255	255
1405	19	255	255	255	1405	19	255	255	255
1406	44	255	255	255	1406	5	255	255	255
1407	76	255	255	255	1407	46	255	255	255
1408	12	255	255	255	1408	1	255	255	255
1409	4	255	255	255	1409	2	255	255	255
1410	1	255	255	255	1410	21	255	255	255
1411	3	255	255	255	1411	79	255	255	255
1412	43	255	255	255	1412	4	255	255	255
1413	56	255	255	255	1413	34	255	255	255
1414	2	255	255	255	1414	1	255	255	255
1415	2	255	255	255	1415	1	255	255	255
1416	2	255	255	255	1416	3	255	255	255
1417	52	255	255	255	1417	31	255	255	255
1418	11	255	255	255	1418	44	255	255	255
1419	10	255	255	255	1419	1	255	255	255
1420	16	255	255	255	1420	1	255	255	255
1421	12	255	255	255	1421	1	255	255	255
1422	13	255	255	255	1422	29	255	255	255
1423	5	255	255	255	1423	2	255	255	255
1424	3	255	255	255	1424	1	255	255	255
1425	12	255	255	255	1425	2	255	255	255
1426	28	255	255	255	1426	3	255	255	255
1427	94	255	255	255	1427	4	255	255	255
1428	1	255	255	255	1428	17	255	255	255
1429	2	255	255	255	1429	15	255	255	255
1430	3	255	255	255	1430	1	255	255	255
1431	58	255	255	255	1431	3	255	255	255
1432	26	255	255	255	1432	2	255	255	255
1433	3	255	255	255	1433	59	255	255	255
1434	1	255	255	255	1434	6	255	255	255
1435	1	255	255	255	1435	2	255	255	255
1436	1	255	255	255	1436	26	255	255	255
1437	1	255	255	255	1437	1	255	255	255
1438	28	255	255	255	1438	3	255	255	255
1439	1	255	255	255	1439	1	255	255	255
1440	42	255	255	255	1440	15	255	255	255
1441	1	255	255	255	1441	4	255	255	255
1442	1	255	255	255	1442	1	255	255	255
1443	53	255	255	255	1443	1	255	255	255
1444	1	255	255	255	1444	24	255	255	255
1445	1	255	255	255	1445	39	255	255	255
1446	104	255	255	255	1446	72	255	255	255
1447	1	255	255	255	1447	6	255	255	255
1448	4	255	255	255	1448	8	255	255	255
1449	27	255	255	255	1449	2	255	255	255
1450	4	255	255	255	1450	1	255	255	255
1451	1	255	255	255	1451	1	255	255	255
1452	1	255	255	255	1452	14	255	255	255
1453	1	255	255	255	1453	9	255	255	255
1454	13	255	255	255	1454	20	255	255	255
1455	1	255	255	255	1455	4	255	255	255
1456	1	255	255	255	1456	31	255	255	255
1457	196	255	255	255	1457	13	255	255	255
1458	3	255	255	255	1458	61	255	255	255
1459	27	255	255	255	1459	5	255	255	255
1460	256	255	255	255	1460	15	255	255	255
1461	3	255	255	255	1461	1	255	255	255
1462	11	255	255	255	1462	10	255	255	255
1463	174	255	255	255	1463	1	255	255	255
1464	10	255	255	255	1464	9	255	255	255
1465	4	255	255	255	1465	1	255	255	255
1466	1	255	255	255	1466	34	255	255	255
1467	30	255	255	255	1467	24	255	255	255
1468	2	255	255	255	1468	53	255	255	255
1469	1	255	255	255	1469	1	255	255	255
1470	1	255	255	255	1470	10	255	255	255
1471	1	255	255	255	1471	2	255	255	255
1472	35	255	255	255	1472	33	255	255	255
1473	1	255	255	255	1473	53	255	255	255
1474	2	255	255	255	1474	1	255	255	255
1475	9	255	255	255	1475	2	255	255	255
1476	3	255	255	255	1476	4	255	255	255
1477	276	255	255	255	1477	9	255	255	255
1478	1	255	255	255	1478	1	255	255	255
1479	23	255	255	255	1479	7	255	255	255
1480	1	255	255	255	1480	10	255	255	255
1481	3	255	255	255	1481	1	255	255	255
1482	122	255	255	255	1482	1	255	255	255
1483	2	255	255	255	1483	6	255	255	255
1484	1	255	255	255	1484	1	255	255	255
					1485	1	255	255	255
					1486	2	255	255	255
					1487	4	255	255	255
					1488	4	255	255	255
					1489	78	255	255	255
					1490	4	255	255	255
					1491	7	255	255	255
					1492	1	255	255	255
					1493	1	255	255	255
					1494	7	255	255	255
					1495	21	255	255	255
					1496	6	255	255	255
					1497	1	255	255	255
					1498	5	255	255	255
					1499	1	255	255	255

Original data for Figure 2D, Densitometric analysis of Nuclear/Perinuclear B-catenin in control and EoE patient esophageal biopsy tissue.

Values are representative of the mean of a sample size N=18. p<0.05**

1500	16	255	255	255
1501	1	255	255	255
1502	2	255	255	255
1503	1	255	255	255
1504	31	255	255	255
1505	87	255	255	255
1506	1	255	255	255
1507	19	255	255	255
1508	2	255	255	255
1509	5	255	255	255
1510	49	255	255	255
1511	1	255	255	255
1512	63	255	255	255
1513	2	255	255	255
1514	2	255	255	255
1515	1	255	255	255
1516	2	255	255	255
1517	77	255	255	255
1518	1	255	255	255
1519	1	255	255	255
1520	1	255	255	255
1521	1	255	255	255
1522	2	255	255	255
1523	1	255	255	255
1524	3	255	255	255
1525	2	255	255	255
1526	2	255	255	255
1527	3	255	255	255
1528	3	255	255	255
1529	2	255	255	255
1530	1	255	255	255
1531	2	255	255	255
1532	3	255	255	255
1533	1	255	255	255
1534	1	255	255	255
1535	3	255	255	255
1536	4	255	255	255
1537	26	255	255	255
1538	18	255	255	255
1539	6	255	255	255
1540	1	255	255	255
1541	1	255	255	255
1542	2	255	255	255
1543	6	255	255	255
1544	2	255	255	255
1545	4	255	255	255
1546	2	255	255	255
1547	115	255	255	255
1548	11	255	255	255
1549	5	255	255	255
1550	1	255	255	255
1551	1	255	255	255
1552	36	255	255	255
1553	1	255	255	255
1554	3	255	255	255
1555	4	255	255	255
1556	13	255	255	255
1557	50	255	255	255
1558	1	255	255	255
1559	3	255	255	255
1560	3	255	255	255
1561	1	255	255	255
1562	3	255	255	255
1563	119	255	255	255
1564	78	255	255	255
1565	6	255	255	255
1566	1	255	255	255
1567	37	255	255	255
1568	1	255	255	255
1569	8	255	255	255
1570	3	255	255	255
1571	43	255	255	255
1572	41	255	255	255
1573	1	255	255	255
1574	2	255	255	255
1575	1	255	255	255
1576	17	255	255	255
1577	45	255	255	255
1578	1	255	255	255
1579	5	255	255	255
1580	8	255	255	255
1581	37	255	255	255
1582	1	255	255	255
1583	2	255	255	255
1584	2	255	255	255
1585	1	255	255	255
1586	1	255	255	255
1588	65	255	255	255
1589	1	255	255	255
1590	1	255	255	255
1591	3	255	255	255
1592	1	255	255	255
1593	1	255	255	255
1594	2	255	255	255
1595	94	255	255	255
1596	2	255	255	255
1597	1	255	255	255
1598	81	255	255	255
1599	2	255	255	255
1600	21	255	255	255
1601	44	255	255	255
1602	3	255	255	255
1603	1	255	255	255
1604	38	255	255	255
1605	2	255	255	255
1606	83	255	255	255
1607	8	255	255	255
1608	2	255	255	255
1609	2	255	255	255
1610	64	255	255	255
1611	3	255	255	255
1612	1	255	255	255
1613	2	255	255	255

Original data for Figure 3B. Quantification of N-Cadherin analysis of control and EoE esophageal biopsy tissue.

Original data for Figure 3B. Quantification of N-Cadherin analysis of control and EoE esophageal biopsy tissue.

Values are representative of mean of a sample size n=18 each group.*p<0.001**

High Content Analysis (HCA)

Average Cell Intensity/hpf

Negatice Control	Control	EoE (>15 Eos)
0	0	543.293
0	0	0
0	0	205.176
0	0	0
0	0	0
0	0	0
0	0	0
0	0	0
0	0	0
0	0	0
0	0	0
0	0	315.553
0	0	0
0	0	0
0	0	227.137
0	0	0
0	0	0
0	0	0
0	0	0
0	0	0
0	0	0
0	0	0
0	0	0
0	0	120.531
0	0	0
0	0	0
0	0	0
0	0	0
0	0	0
0	0	0
0	0	102.446
0	0	0
0	0	0
0	0	0
0	0	213.431
0	0	0
0	0	0
0	0	219.014
0	0	0
0	0	575.85
0	0	0
0	0	0
0	0	0
0	0	0
0	0	512.349
0	0	0
0	0	0
0	0	0
0	0	0
0	0	0
0	0	0
0	0	0
0	0	0
0	0	0
0	0	0
0	0	339.137
0	0	0
0	0	0
0	0	0
0	0	0
0	0	0
0	0	0
0	0	0
0	0	0
0	0	117.319
0	0	0
0	0	0
0	0	607.563
0	0	0

Original data for Figure 3B. Quantification of N-Cadherin analysis of control and EoE esophageal biopsy tissue.
Values are representative of mean of a sample size n=18 each group.*p<0.001**

0	0	0
0	0	0
0	0	0
0	0	0
0	0	0
0	0	221.327
0	0	210.408
0	0	232.036
0	0	250.222
0	0	220.437
0	0	257.755
0	0	245.225
0	0	295.422
0	0	286.501
0	0	279.296
0	0	277.252
0	0	213.128
0	0	215.203
0	0	191.986
0	0	250.392
0	0	252.801
0	0	127.394
0	0	251.295
0	0	262.859
0	0	151.257
0	0	220.128
0	0	219.538
0	0	278.501
0	0	316.292
0	0	273.196
0	0	253.371
0	0	261.973
0	0	234.416
0	0	247.182
0	0	263.378
0	0	277.333
0	311.6202	265.209
0	0	389.355
0	0	294.352
0	0	270.463
0	338.8188	222.011
0	0	329.756
0	307.0476	266.805
0	0	269.131
0	0	260.591
0	0	240.407
0	0	207.685
0	356.8889	292.19
0	0	277.971
0	0	471.904
0	0	268.923
0	0	203.2
0	0	279.421
0	0	302.732
0	0	235.612
0	0	230.251
0	0	271.159
0	0	181.063
0	347.1351	236.038
0	0	381.272
0	0	219.958
0	0	286.287
0	194.6923	172.723
0	0	219.917
0	0	344.339
0	422.0899	193.206
0	0	179.646
0	0	350.72
0	0	246.597
0	0	262.31
0	0	279.82
0	182.4349	357.554
0	0	240.905
0	0	281.788
0	0	251.404
0	0	319.068

Original data for Figure 3B. Quantification of N-Cadherin analysis of control and EoE esophageal biopsy tissue.

Values are representative of mean of a sample size n=18 each group.*p<0.001**

0	0	269.981
0	0	285.481
0	308.8452	315.434
0	0	256.408
0	0	243.521
0	189.1797	225.296
0	0	190.826
0	0	309.729
0	0	278.747
0	0	310.61
0	286.1571	264.545
0	0	235.594
0	0	327.745
0	229.9194	240.481
0	0	273.436
0	0	263.909
0	236.3735	114.191
0	0	183.722
0	0	254.025
0	0	227.838
0	0	332.802
0	0	370.78
0	0	258.498
0	0	249.62
0	0	292.712
0	0	289.069
0	0	313.999
0	0	245.693
0	0	251.778
0	0	309.587
0	0	212.664
0	0	0
0	0	300.605
0	0	279.688
0	0	228.688
0	0	209.812
0	0	178.111
0	0	343.455
0	0	326.67
0	0	263.22
0	0	250.159
0	0	234.582
0	0	268.813
0	0	156.119
0	0	216.691
0	0	302.722
0	0	238.19
0	0	200.785
0	0	291.633
0	0	275.155
0	0	232.644
0	0	282.524
0	0	314.169
0	0	304.867
0	0	277.984
0	0	267.888
0	0	203.672
0	0	165.811
0	0	177.602
0	0	237.989
0	0	167.064
0	0	193.494
0	0	186.723
0	0	0
0	0	0
0	0	333.754
0	0	0
0	0	0
0	0	0
0	0	0
0	0	0
0	0	0
0	0	0
0	0	0
0	0	0
0	0	0

Original data for Figure 3B. Quantification of N-Cadherin analysis of control and EoE esophageal biopsy tissue.

Values are representative of mean of a sample size n=18 each group.*p<0.001**

0	0	0
0	0	0
0	0	0
0	0	299.564
0	0	0
0	0	0
0	0	0
0	0	0
0	0	0
0	0	735.317
269.68	0	0
0	0	0
0	0	0
0	0	0
0	0	0
0	0	0
0	0	0
0	0	0
0	0	0
0	0	0
0	0	0
0	0	226.22
0	0	0
0	0	489.44
0	0	0
0	0	0
0	0	0
0	0	0
0	0	0
0	674.3178	0
0	0	0
0	0	0
0	417.2586	0
0	0	0
0	0	0
0	0	0
0	0	413.856
0	0	752.428
0	0	0
0	0	0
0	0	0
0	0	0
0	0	1311.82
0	0	1099.95
0	0	0
0	0	1247.51
0	0	906.442
0	0	0
0	0	0
0	0	0
0	0	0
0	0	0
0	0	0
0	0	0
0	0	277.084
0	0	0
0	0	0
0	0	881.231
0	0	0
0	0	249.696
0	0	0
0	0	0
0	0	0
0	0	330.07
0	0	0
0	0	0
201.7547	0	0
0	0	359.263
0	0	263.578
0	0	0
0	0	0
0	0	0
0	0	240.272
0	0	221.882
0	0	0
0	0	0

Original data for Figure 3B. Quantification of N-Cadherin analysis of control and EoE esophageal biopsy tissue.

Values are representative of mean of a sample size n=18 each group.*p<0.001**

0	0	275.012
0	0	428.367
0	0	591.04
0	678.2347	0
0	0	0
0	0	0
0	0	392.767
0	0	0
0	0	0
0	0	0
0	0	345.267
0	375.205	993.294
0	0	238.994
0	0	0
0	0	465.762
0		494.651
0		0
0		706.559
0		259.811
0		0
0		0
0		0
0		880.751
0		0
0		0
0		0
0		1620.04
0		0
0		229.957
0		0
0		328.408
0		986.551
0		0
0		375.282
0		0
0		0
0		0
0		0
255.9048		560.957
0		0
0		285.288
0		
0		
195.2872		
0		

Original data for Figure 3B. Quantification of N-Cadherin analysis of control and EoE esophageal biopsy tissue.
Values are representative of mean of a sample size n=18 each group.*p<0.001**

Statistics:

Table Analy	All data N-cadherine
Column A	
vs	vs
Column C	
Unpaired t test	
P value	P<0.0001
P value sur	***
Are means	Yes
One- or tw	Two-tailed
t, df	t=14.21 df=678
How big is the difference?	
Mean ± SEI	2.698 ± 1.356 N=342
Mean ± SEI	175.6 ± 12.17 N=338
Difference	-172.9 ± 12.17
95% confid	-196.8 to -149.1
R squared	0.2295
F test to compare variances	
F,DFn, Dfd	79.50, 337, 341
P value	P<0.0001
P value sur	***
Are varianc	Yes

Original data for Figure 4B. Quantification of TWIST1 IF analysis of control and EoE esophageal biopsy tissue.

Original data for Figure 4B. Quantification of TWIST1 IF analysis of control and EoE esophageal biopsy tissue.

Values are representative of mean of sample n=18 each group .*p<0.001**

High Content Analysis (HCA)

Average Cell Intensity/hpf

Negatice Control	Control	EoE (>15 Eos)
0.009394	0	428.5533
0	120.704	399.5126
0	0	365.1852
0.008866	0	427.3385
0.011897	0	363.0961
0	0	256.4902
0.007712	157.955	406.0459
0	146.513	422.3068
0.01255	0	419.9322
0.009825	154.795	454.4507
0.015485	0	528.8448
0.017976	177.243	380.1004
0	155.512	395.3482
0	157.596	502.3618
0	168.485	474.2701
0	143.474	410.7964
0	125.254	446.9244
0	143.576	477.4219
0.009252	155.726	372.2559
0	147.809	363.0698
0	147.081	393.6325
0	130.77	418.5338
0	0	350.5869
0.006929	146.412	357.9615
0	150.882	460.915
0	148.154	513.2569
0	173.988	386.2083
0	146.61	453.5069
0	0	443.0008
0.01635	148.262	401.81
0	124.5	488.0079
0	143.074	491.6218
0	161.762	0
0.006581	484.991	0
0.008493	0	398.5579
0	0	333.5373
0	0	410.7735
0.005884	501.271	510.7211
0	499.005	420.2861
0	819.966	0
0	0	435.5699
0.019462	0	390.4828
0.013113	0	412.9364
0.013742	0	445.9398
0	628.594	488.134
0.015775	327.477	430.2755
0.013011	0	434.3298
0	0	463.1194
0	758.052	453.2112
0.010463	0	377.627
0.017525	0	469.9797
0	549.131	512.4368
0	0	407.9128
0.016919	0	395.0429
0.008896	621.266	420.2854
0.006114	0	458.1958
0.009186	514.398	451.322
0.01891	463.403	463.9505
0.038567	0	427.1406
0	450.756	443.9726
0.023058	0	419.8124
0.013436	610.26	398.709
0.011127	0	425.5801
0.027495	548.547	409.8024
0.008398	0	514.6855
0.012679	0	387.1296
0	603.66	408.7748
0.00602	0	492.032
0.013633	0	448.3894
0.007618	0	445.6891
0.010508	421.585	469.4345

Original data for Figure 4B. Quantification of TWIST1 IF analysis of control and EoE esophageal biopsy tissue.

Values are representative of mean of sample n=18 each group .*p<0.001**

0	636.667	394.835
0.009394	467.733	322.3596
0	556.667	273.974
0	572.207	0
0.008866	342.214	399.0208
0.011897	523.255	379.5731
0	596.335	436.0696
0.007712	0	429.1007
0	439.153	324.3448
0.01255	0	289.4075
0.009825	0	208.7886
0.015485	584.603	378.0613
0.017976	510.436	404.7932
0	0	406.2805
0	495.646	282.6832
0	0	421.3837
0	392.185	362.4267
0	0	421.8144
0	340.442	402.3276
0.009252	0	407.4127
0	0	459.6099
0	577.567	317.742
0	0	372.6727
0	530.096	415.798
0.006929	0	457.7176
0	564.47	362.3422
0	332.365	274.7352
0	0	465.5025
0	0	543.249
0	0	403.4412
0.01635	0	0
0	0	335.6435
0	0	289.0969
0	0	393.2506
0.006581	483.177	261.2141
0.008493	379.907	308.9714
0		0
0	0	256.0754
0.005884	430.077	330.7087
0	0	396.6994
0	0	390.3045
0	0	389.6645
0.019462	0	458.4546
0.013113	0	395.6006
0.013742	0	245.3704
0	491.712	289.2642
0.015775	0	399.1054
0.013011	0	394.2724
0	0	247.4006
0	0	419.8389
0.010463	0	319.396
0.017525	0	330.0486
0	0	402.0812
0	0	353.9367
0.016919	797.025	410.9127
0.008896	0	248.1136
0.006114	0	377.2592
0.009186	0	274.657
0.01891	0	382.1479
0.038567	0	327.2429
0	0	299.6746
0.023058	0	300.3447
0.013436	0	271.4353
0.011127	0	395.0117
0.027495	0	404.1135
0.008398	0	464.0332
0.012679	0	576.7894
0	0	696.1354
0.00602	0	474.4615
0.013633	0	889.2126
0.007618	0	711.7128
0.010508	0	479.2695
0	0	743.8438
206.0048	0	670.567
0	603.125	657.7568
0	0	643.5501
194.4374	0	794.2601
260.8902	1336.14	473.1828
0	711.513	652.5811
169.1205	448.623	0
0	447.007	288.8784

Original data for Figure 4B. Quantification of TWIST1 IF analysis of control and EoE esophageal biopsy tissue.

Values are representative of mean of sample n=18 each group .*p<0.001**

275.2133	581.497	612.5035
215.465	0	384.0785
33.95892	563.647	449.7801
0	508.583	631.9047
0	1509.75	551.7755
0	467.822	579.5506
0	0	548.962
0	567.301	842.2667
0	0	610.2292
0	724.415	780.6974
202.894	550.901	818.0764
0	0	765.3838
0	0	803.6832
0	0	805.5368
0	0	614.2905
151.95	0	624.8226
0	0	554.1583
0	0	530.2036
0	0	0
0	0	470.6152
0	0	619.5299
358.5561	0	611.1985
0	0	680.9115
0	0	461.6685
0	0	511.468
144.3189	0	464.0064
186.2523	0	715.7149
0	0	427.0359
0	0	602.2588
129.0291	0	626.5534
0	0	723.8781
0	0	861.8959
0	0	526.4858
426.8118	0	766.8417
287.5718	0	596.8797
301.3726	0	472.5474
0	0	546.3063
345.9517	0	611.8378
285.3296	0	710.4668
0	0	535.9648
0	0	574.3207
229.4578	0	631.2714
384.3321	0	547.7518
0	0	649.0388
0	0	695.4938
371.024	0	727.4675
195.0829	0	728.1971
134.0801	0	505.2568
201.4514	0	832.0758
414.702	0	679.4909
84.7653	0	648.3625
0	0	764.9811
	0	713.0101
	0	542.2271
	638.422	493.9506
	0	687.5706
	0	769.9548
	0	466.2555
	0	776.7684
	0	608.4745
	0	668.4136
	0	685.7325
	0	442.3316
	0	452.324
	0	480.7885
	528.595	601.1234
	0	544.1965
	0	516.1378
	646.912	585.9567
	0	595.5927
	0	422.4172
	0	736.9032
	0	755.0892
	0	722.4993
	0	626.6127
	0	729.8539
	0	696.7099
	0	616.4597
	0	776.1423
	0	464.3917
	568.832	561.3235

Original data for Figure 4B. Quantification of TWIST1 IF analysis of control and EoE esophageal biopsy tissue.

Values are representative of mean of sample n=18 each group .*p<0.001**

0	738.2642
0	542.5781
0	412.5792
570.281	490.6604
0	711.0132
0	451.7904
0	631.8527
0	585.7603
0	604.2292
0	765.8654
0	533.731
0	739.5685
0	524.9454
0	612.9247
555.746	692.8574
0	626.4015
0	570.5869
0	421.5347
0	717.563
0	652.3324
0	535.2424
0	702.1309
0	700.5397
0	567.7314
0	593.8572
0	638.207
0	585.913
0	745.3491
0	761.8944
0	768.5951
0	801.4127
0	518.7601
0	778.3865
0	579.3934
0	579.2189
0	537.2675
	636.48
	445.2082
	703.8
	653.0229
	688.713
	613.8706
	505.1816
	539.3931
	659.9478
	603.6599
	471.9688
	604.4968
	678.9429
	382.8105
	768.593
	539.5955
	527.6211
	640.6511

Original data for Figure 4B. Quantification of TWIST1 IF analysis of control and EoE esophageal biopsy tissue.

Values are representative of mean of sample n=18 each group .*p<0.001**

Statistics

Twist intensity per hpf
(arbitrary units)

Table	Twist final neg ct normal and EoE
Column B	
vs	vs
Column C	
Unpaired t test	
P value	< 0.0001
P value sur	***
Are means	Yes
One- or twc	Two-tailed
t, df	t=20.54 df=553
How big is the difference?	
Mean ± SE	134.1 ± 15.08 N=268
Mean ± SE	500.1 ± 9.910 N=287
Difference	-365.9 ± 17.82
95% confid	-400.8 to -331.0
R squared	0.4327
F test to compare variances	
F,DFn, Dfd	2.162, 267, 286
P value	< 0.0001
P value sur	***
Are varianc	Yes
